# The Risk Correlation between N7-Methylguanosine Modification-Related lncRNAs and Survival Prognosis of Oral Squamous Cell Carcinoma Based on Comprehensive Bioinformatics Analysis

**DOI:** 10.1155/2022/1666792

**Published:** 2022-08-24

**Authors:** Yanglong Xu, Xue Zou, Jie Mei

**Affiliations:** ^1^Department of General Dentistry, Stomatological Hospital Affiliated to Zunyi Medical University, China; ^2^Department of Periodontics, Stomatological Hospital Affiliated to Zunyi Medical University, China; ^3^Stomatological Hospital, Southern Medical University, China

## Abstract

**Objective:**

N7-methylguanosine modification-related lncRNAs (m7G-related lncRNAs) are involved in progression of many diseases. This study was aimed at revealing the risk correlation between N7-methylguanosine modification-related lncRNAs and survival prognosis of oral squamous cell carcinoma.

**Methods:**

In the present study, coexpression network analysis and univariate Cox analysis were used to obtained 31 m7G-related mRNAs and 399 m7G-related lncRNAs. And the prognostic risk score model of OSCC patients was evaluated and optimized through cross-validation.

**Results:**

Through the coexpression analysis and risk assessment analysis of m7G-related prognostic mRNAs and lncRNAs, it was found that six m7G-related prognostic lncRNAs (AC005332.6, AC010894.1, AC068831.5, AL035446.1, AL513550.1, and HHLA3) were high-risk lncRNAs. Three m7G-related prognostic lncRNAs (AC007114.1, HEIH, and LINC02541) were protective lncRNAs. Then, survival curves were drawn by comparing the survival differences between patients with high and low expression of each m7G-related prognostic lncRNA in the prognostic risk score model. Further, risk curves, scatter plots, and heat maps were drawn by comparing the survival differences between high-risk and low-risk OSCC patients in the prognostic model. Finally, forest maps and the ROC curve were generated to verify the predictive power of the prognostic risk score model. Our results will help to find early and accurate prognostic risk markers for OSCC, which could be used for early prediction and early clinical intervention of survival, prognosis, and disease risk of OSCC patients in the future.

## 1. Introduction

Oral squamous cell carcinoma (OSCC) is a malignant tumor occurring in oral cavity with squamous cell as the main cell. Cancer cells can occur in gingival, hard palate, tongue, buccal mucosa, lips, and other organs [1]. It is the most malignant and harmful tumor of the head and neck, accounting for about 50% of the incidence of the head and neck squamous cell carcinoma [2]. Due to the rich blood flow and complex anatomical structure of oral and maxillofacial region, OSCC surgery often cannot completely remove the tumor [3]. At the same time, OSCC is prone to lymph node metastasis and postoperative recurrence, so its prognosis is poor [4]. Therefore, it is particularly urgent to identify new prognostic genes to accurately predict the prognosis of OSCC patients at an early stage.

Long noncoding RNAs (lncRNAs) are noncoding RNAs with a length of more than 200 nucleotides. They are functional RNA molecules that cannot be translated into proteins, but they are involved in a variety of biological regulation processes in cells of the body, such as epigenetic regulation, cell cycle regulation, and cell differentiation regulation [5, 6]. N7-methylguanine (m7G) is a metabolite of RNA methylation, which can be produced by methylation agents and is used as probes for protein-RNA interactions and as a key component of RNA sequencing methods [7, 8].

Some studies have shown that N7-methylguanosine modification-related lncRNAs (m7G-related lncRNAs) are involved in maintaining RNA stability, processing, nucleation, translation, and other functions, affecting the occurrence and progression of many diseases [9–13]. However, as far as we know, no studies on m7G-related lncRNAs have been reported in OSCC. This study was aimed at revealing the risk correlation between N7-methylguanosine modification-related lncRNAs and survival prognosis of oral squamous cell carcinoma.

In this study, we constructed a prognostic risk score model for OSCC based on the new m7G-related lncRNAs; conducted risk assessment, survival analysis, and risk analysis for OSCC patients; and finally verified the accuracy and independent predictive ability of the prognostic risk model. The purpose of this study was to find early and accurate prognostic risk markers for OSCC, which could be used for early prediction and early clinical intervention of survival, prognosis, and disease risk of OSCC patients in the future.

## 2. Materials and Methods

### 2.1. Acquisition and Cleaning of Gene Matrix and Clinical Data

The transcriptome expression matrix of all OSCC samples and clinical data of patients were obtained from The Cancer Genome Atlas (TCGA) database (https://tcga-data.nci.nih.gov/tcga), which provides clinical information and genome variation, mRNA expression, miRNA expression, methylation, and other data on various human cancers, making it an important data source for cancer researchers [14, 15]. A total of 83 samples were collected, including 70 OSCC tissue samples and 13 normal tissue samples. In addition, we used custom Perl scripts to clean and organize all the data for further bioinformatics analysis and statistical analysis.

### 2.2. Identification of m7G-Related mRNAs and lncRNAs

Firstly, the transcriptome expression matrix of all OSCC samples was divided into mRNA and lncRNA expression profiles using customized Perl scripts and human gene profiles. Then from MSigDB database (http://www.gsea-msigdb.org/gsea/login.jsp) [16] to arrange m7G-related mRNAs list, then extracted each relative expression of m7G-related mRNAs via R package limma [17] from OSCC gene matrix. Finally, the lncRNA expression matrix and the list of m7G-related mRNAs were read respectively; the expression values of repeated genes and normal samples were removed; the coexpression of lncRNAs with m7G-related mRNAs was identified through the cyclic calculation of correlation test, named as m7G-related lncRNAs; and the expression matrix was obtained [18]. The cutoff criterion was Pearson correlation coefficient > 0.4 and *P* value < 0.001.

### 2.3. Identification of m7G-Related Prognostic lncRNAs

First, the survival data of all patients (including survival time and survival status) were collated, and the m7G-related lncRNA expression matrix was combined with the survival data of patients (only patients with complete information were included). Then, Kaplan-Meier (KM) analysis and univariate Cox analysis methods [19, 20] were used to test the correlation between m7G-related lncRNA expression levels and survival time and status of patients via R package survival [21], so as to identify the m7G-related prognostic lncRNAs. Only *P* values less than 0.05 for the two analysis methods were considered statistically significant.

### 2.4. Construction of Prognostic Risk Score Model for OSCC

We used the m7G-related prognostic lncRNA expression matrix with survival information as input file to construct Cox model through R package survival and optimized the model by calculating AIC value, so as to obtain the formula of prognostic risk score model [22, 23]. We then calculated each OSCC patient's risk scores based on this formula and compared the risk scores with the median risk score to predict whether each patient was a high- or low-risk patient.

### 2.5. Analysis of Prognostic Gene Coexpression Network

First, we extracted the list of m7G-related prognostic lncRNAs involved in prognostic model construction and the table of coexpression relationship between m7G-related prognostic mRNAs and lncRNAs. Then, by customizing Perl scripts, we sorted out and obtained coexpression relations and node attributes of m7G-related prognostic mRNAs and lncRNAs, which were used as input files to build coexpression networks [24]. Finally, we visualized the prognostic gene coexpression network using Cytoscape software [25]. In the gene coexpression network, hub genes represent a small proportion of nodes with maximal information exchange with other nodes. Cytoscape is an open source software for visualizing complex networks and integrating these with any type of attribute data.

### 2.6. Risk Assessment of m7G-Related Prognostic lncRNAs

In order to identify which m7G-related prognostic lncRNAs in the prognostic model are high-risk lncRNAs (hazard ratio, HR > 1) and which are protective lncRNAs (HR < 1), we analyzed the association between m7G-related prognostic lncRNAs and mRNAs [26] and conducted a risk assessment for these lncRNAs via packages ggalluvial, ggplot2, and dplyr [27, 28]. The result was represented by a Sankey diagram [29].

### 2.7. Survival Analyses of m7G-Related Prognostic lncRNAs

Through R package survival, survival analyses were performed on all m7G-related prognostic lncRNAs involved in the establishment of the prognostic risk score model to explore the relationship between m7G-related prognostic lncRNA expression and survival prognosis of OSCC patients [30]. Prognostic models or risk scores are frequently used to aid individualize risk assessment for diseases with multiple, complex risk factors and diagnostic challenges. According to the median m7G-related prognostic lncRNA expression value, all OSCC patients were divided into high m7G-related prognostic lncRNA expression group and low m7G-related prognostic lncRNA expression group, and then, survival analysis function was defined to compare whether there was statistical difference in survival rate between the two groups [31]. Results were visualized by survival curves [32].

### 2.8. Risk Analysis of the Prognostic Risk Score Model for OSCC

To further elucidate the relationship between patient survival time, survival status, m7G-related prognostic lncRNA expression levels, and risk scores, we conducted a comprehensive risk analysis for OSCC patients with high- and low-risk groups. Results were displayed by a risk curve, a survival scatter plot, and a risk gene heat map [33, 34].

### 2.9. Independent Prognostic Analysis of the Prognostic Risk Score Model for OSCC

We performed independent prognostic analyses of the prognostic risk score model to verify whether risk score could be an independent prognostic factor for OSCC patients [35]. Univariate and multivariate independent prognostic analyses were performed using the R package survival by extracting and combining risk information (including survival time, survival status, risk score, and m7G-related prognostic lncRNA expression matrix) and clinical trait lists (including patients' age, gender, cancer grade, and stage) of all patients. The results were shown in forest maps [36].

### 2.10. ROC Curve Analyses of Several Clinical Traits in the Prognostic Model

Receiver operating characteristic (ROC) curve, also known as sensitivity curve, is a comprehensive index reflecting the continuous variables of sensitivity and specificity [37]. Generally, the relationship between the two is revealed by using the composition method. The larger the area under ROC curve (AUC), the higher the diagnostic accuracy of the test [38]. To test the predictive performance of OSCC prognostic risk score model, R package survival ROC was used to perform KM analysis on patients' survival time, survival status, risk score, and clinical characteristics (age, gender, tumor stage, and grade), and ROC curves of risk score and clinical traits were plotted [39, 40].

## 3. Results

### 3.1. Identification of m7G-Related lncRNAs

We downloaded 60,660 gene transcription expression matrices from TCGA database, including 16,798 lncRNAs and 19,926 mRNAs. According to MSigDB database and OSCC mRNA matrix, 31 m7G-related mRNAs were obtained. Then, 399 m7G-related lncRNAs were identified through coexpression network analysis. For the expression matrix and coexpression relationship of m7G-related lncRNAs, see Supplementary Files (m7G-lncRNAs_exp.xls and co-exp_rel.xls).

### 3.2. Identification of m7G-Related Prognostic lncRNAs

Combined with KM analysis and univariate Cox analysis, we identified 16 m7G-related prognostic lncRNAs (as shown in Table 1). Three lncRNAs, LINC02541, AC007114.1, and HEIH, were low-risk lncRNAs (HR values < 1), and the rest were high-risk lncRNAs (HR values > 1). The *P* values of lncRNAs identified by the two methods were all less than 0.05.

### 3.3. The Prognostic Risk Score Model for OSCC

We performed univariate Cox analysis on 16 m7G-related prognostic lncRNAs and optimized 9 m7G-related prognostic lncRNAs, among which LINC02541, AC007114.1, and HEIH were low-risk lncRNAs (HR value < 1) and HHLA3, AC010894.1, AL513550.1, AL035446.1, AC068831.5, and AC005332.6 were high-risk lncRNAs (HR value > 1). The prognostic risk score model of OSCC patients was evaluated and optimized through cross-validation. lncRNAs and prognostic model formula that comprised the prognostic risk score model are shown in Tables 2 and 3. The raw data related to the prognostic model are detailed in Supplementary Files (risk.xls and uniSigExp.xls).

### 3.4. The Coexpression Network of m7G-Related Prognostic lncRNAs and mRNAs

**Figure 1:**
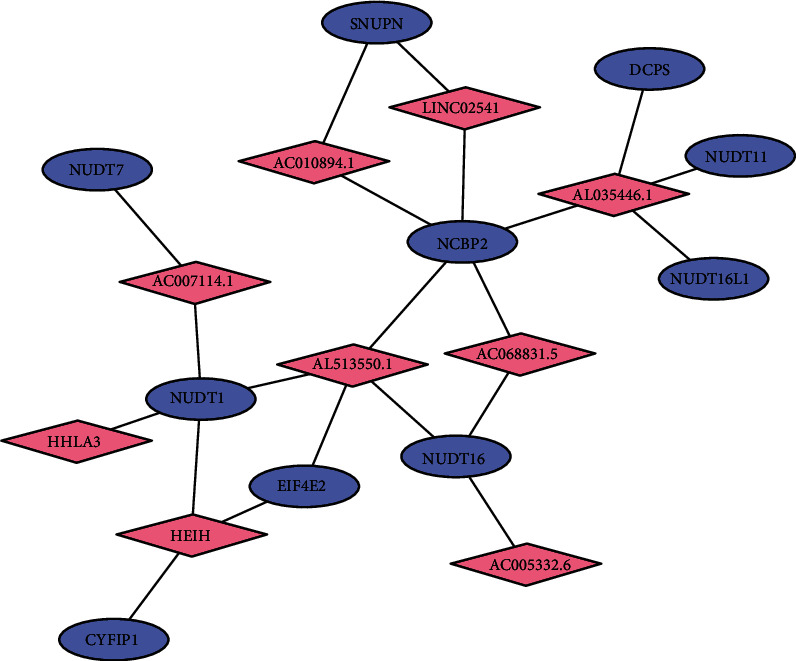
The coexpression network of m7G-related prognostic lncRNAs and mRNA. Pink diamonds represent lncRNAs, and blue ellipses represent mRNAs. Black solid lines represent the coexpression relationships between the mRNAs and lncRNAs.

A total of 10 m7G-related prognostic mRNAs and 9 m7G-related prognostic lncRNAs were identified through coexpression network analysis. For details about the coexpression relationship between the m7G-related prognostic lncRNAs and mRNAs, see Supplementary Files (coexp_network.xls). Coexpression network was used to visualize the correlation between the 10 m7G-related prognostic mRNAs and 9 m7G-related prognostic lncRNAs (Figure 1).

### 3.5. Risk Identification of m7G-Related Prognostic lncRNAs

**Figure 2:**
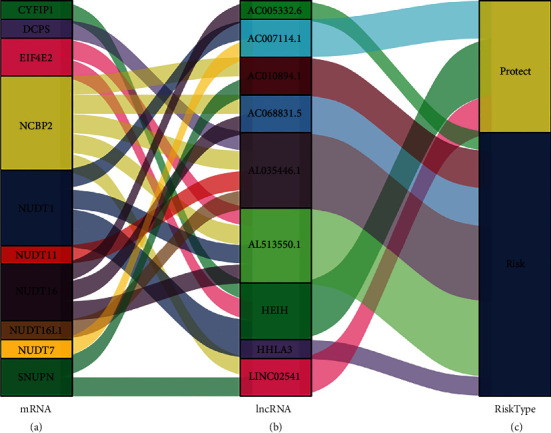
The Sankey diagram showed the risk profile of nine m7G-related prognostic lncRNAs. The blocks represent (a) the m7G-related prognostic mRNAs, (b) the m7G-related prognostic lncRNAs, and (c) the risk types of lncRNAs.

Through the coexpression analysis and risk assessment analysis of m7G-related prognostic mRNAs and lncRNAs, it was found that six m7G-related prognostic lncRNAs (AC005332.6, AC010894.1, AC068831.5, AL035446.1, AL513550.1, and HHLA3) were high-risk lncRNAs. Three m7G-related prognostic lncRNAs (AC007114.1, HEIH, and LINC02541) were protective lncRNAs. The Sankey diagram showed the risk profile of all m7G-related prognostic lncRNAs (Figure 2).

### 3.6. Survival Curves of m7G-Related Prognostic lncRNAs

**Figure 3:**
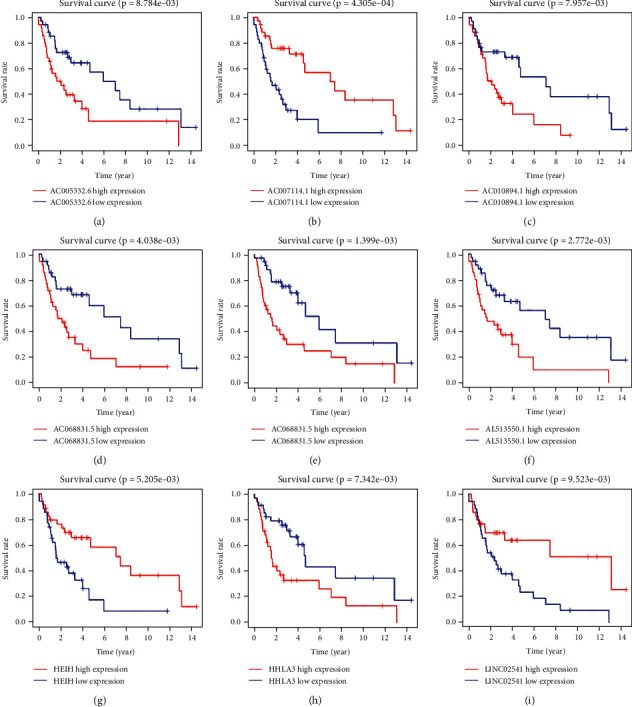
The survival curve of high (red) and low (blue) expression groups of m7G-related prognostic lncRNAs. Abscissa: survival years of patients; ordinate: survival rate of patients.

Survival curves were drawn by comparing the survival differences between patients with high and low expression of each m7G-related prognostic lncRNA in the prognostic risk score model. As shown in Figure 3, the survival of six m7G-related prognostic lncRNAs (AC005332.6, AC010894.1, AC068831.5, AL035446.1, AL513550.1, and HHLA3) with the high expression group was significantly lower than that with the low expression group (*P* < 0.05), while the survival of the other three m7G-related prognostic lncRNAs (AC007114.1, HEIH, and LINC02541) with the high expression group was significantly higher than that with the low expression group (*P* < 0.05).

### 3.7. Risk Assessment of the Prognostic Risk Score Model for OSCC

**Figure 4:**
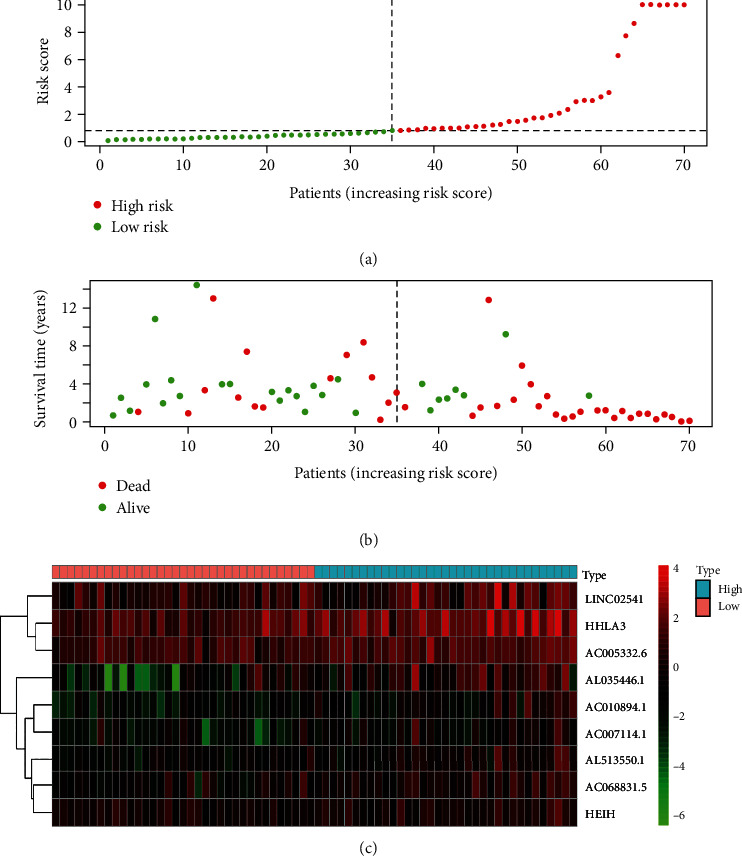
(a) The risk curves of OSCC patients. Abscissa: patients' risk scores ranked from low to high; ordinate: risk score. Red dots: high-risk group; green dots: low-risk group. (b) The risk scatter plots of OSCC patients. Ordinate: survival time. Red dots: dead patients; green dots: alive patients. (c) The risk heat maps of nine m7G-related prognostic lncRNAs of OSCC patients. Red squares: high-expression lncRNAs; green squares: low-expression lncRNAs.

Risk curves, scatter plots, and heat maps were drawn by comparing the survival differences between high-risk and low-risk OSCC patients in the prognostic risk score model. As shown in Figure 4(a), the risk score of OSCC patients in the high-risk group was significantly higher than that in the low-risk group (*P* < 0.05). As shown in Figure 4(b), there were significantly more deaths in the high-risk group than in the low-risk group. In addition, Figure 4(c) shows that AC005332.6, AC010894.1, AC068831.5, AL035446.1, AL513550.1, and HHLA3 were highly expressed in the high-risk OSCC group, while AC007114.1, HEIH, and LINC02541 were highly expressed in the low-risk OSCC group. These results suggested that this prognostic risk score model could accurately predict the prognostic risk outcomes of both groups of OSCC patients.

### 3.8. Validation of the Predictive Power of the Prognostic Risk Score Model

**Figure 5:**
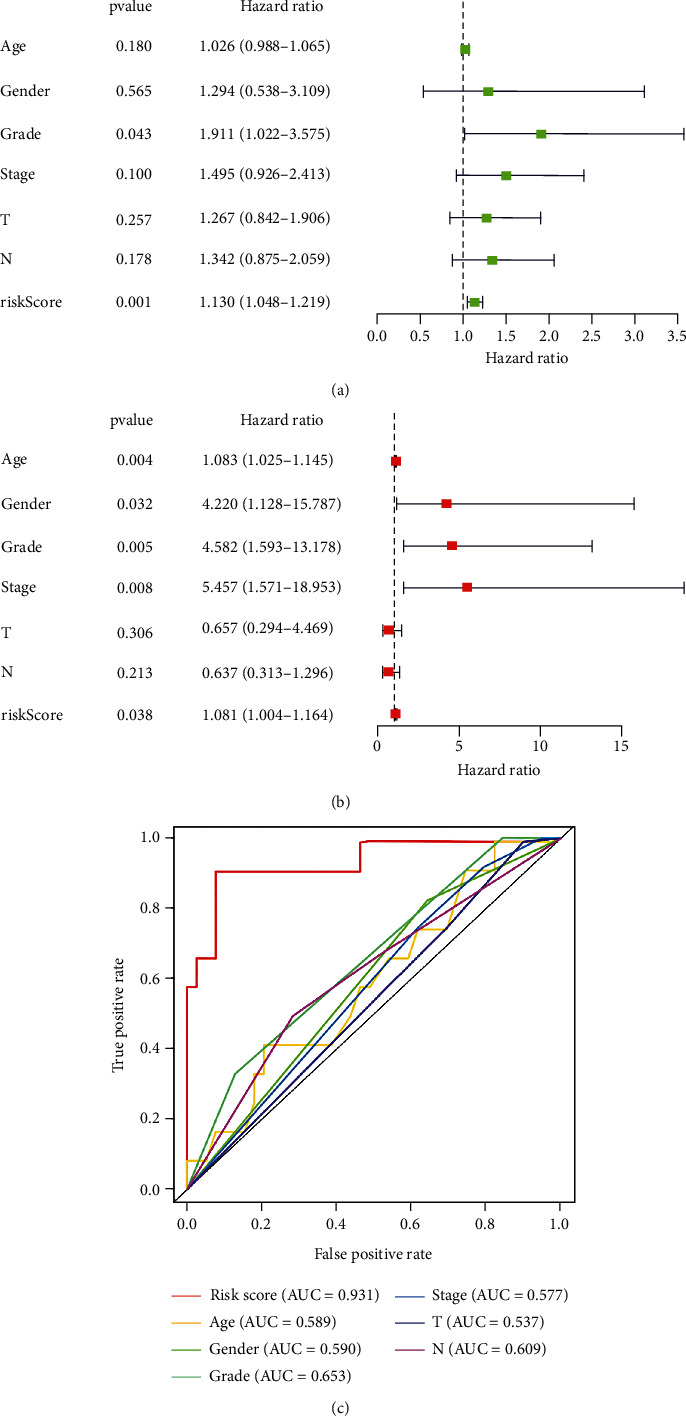
Forest plots of univariate (a) and multivariate (b) independent prognostic analyses of clinical trait parameters in OSCC patients. Green or red squares represent hazard ratio (HR) value, and blue solid lines represent 95% confidence intervals. (c) A ROC curve of the prognostic risk score model. The different colored curves represent different clinical trait parameters. AUC: area under the ROC curve. Abscissa: false positive rate (1 − specificity); ordinate: true positive rate (sensitivity).

Forest maps and the ROC curve were generated to verify the predictive power of the prognostic risk score model. According to Figures 5(a) and 5(b), *P* values of risk score were less than 0.05 and HR values were greater than 1 in both univariate and multivariate independent prognostic analyses, suggesting that risk score in the prognostic risk score model may be a reliable clinical independent prognostic factor. As shown in Figure 5(c), the risk score had the highest AUC value (0.931) compared to other clinical trait parameters. These results suggested that the prognostic risk score model has the ability to predict the prognosis of OSCC patients accurately and independently.

## 4. Discussion

Factors that influence the recurrence of OSCC have been extensively explored in recent years. Cai et al. [40] have analyzed the patient clinicopathologic data, including tumor sites, clinical and pathologic stage, histological grade, invasion mode, and perineural invasion. They have concluded that tongue cancer and poor differentiation contributed to OSCC recurrence after surgery. Xia et al. [41] have reported that the recurrence rate was 44.9% in 118 patients with OSCC. Statistical analysis showed that comorbidities, degree of tumor differentiation, and tumor stage were important prognostic factors for recurrence. In this paper, 31 m7G-related mRNAs and 399 m7G-related lncRNAs were obtained through coexpression network analysis. Afterwards, we performed univariate Cox analysis on 16 m7G-related prognostic lncRNAs and optimized 9 m7G-related prognostic lncRNAs, and the prognostic risk score model of OSCC patients was evaluated and optimized through cross-validation. Further, through the coexpression analysis and risk assessment analysis of m7G-related prognostic mRNAs and lncRNAs, it was found that six m7G-related prognostic lncRNAs (AC005332.6, AC010894.1, AC068831.5, AL035446.1, AL513550.1, and HHLA3) were high-risk lncRNAs. Three m7G-related prognostic lncRNAs (AC007114.1, HEIH, and LINC02541) were protective lncRNAs. Then, survival curves were drawn by comparing the survival differences between patients with high and low expression of each m7G-related prognostic lncRNA in the prognostic risk score model. Further, risk curves, scatter plots, and heat maps were drawn by comparing the survival differences between high-risk and low-risk OSCC patients in the prognostic model. Finally, forest maps and the ROC curve were generated to verify the predictive power of the prognostic risk score model.

So far, no study has reported the risk correlation between m7G-related lncRNAs and survival prognosis of OSCC based on bioinformatics analysis. However, in other areas of cancer, a few studies have found that m7G-modification is involved in gene regulation of tumor cell biology. Chen et al. [10] conducted tRNA modification and expression profile, mRNA translation profile, and rescue analysis in a conditional gene knockout mouse model and found that abnormal translation regulated by METTL1/WDR4-mediated tRNA m7G-modification drives the development and progression of squamous cell carcinoma of the head and neck. Xia et al. [41] verified the high expression of WD repeat Domain 4 (WDR4) in hepatocellular carcinoma (HCC) by cell culture and functional experiments and observed that upregulated WDR4 expression increased m7G-methylation level in HCC. And HCC cell proliferation was promoted by inducing G2/M cell cycle conversion and inhibiting apoptosis. Liu et al. [42] confirmed that methyltransferase-like 1 (METTL1) acts as a tumor suppressor in colon cancer by activating m7G-regulated let-7e miRNA/HMGA2 axis through quantitative PCR, Western blot, CCK-8 assay, transwell assay, and dual-luciferase reporter gene system. To our knowledge, this study has manifested the risk correlation between N7-methylguanosine modification-related lncRNAs and survival prognosis of oral squamous cell carcinoma for the first time. Our data has identified m7G-related lncRNAs through coexpression analysis and the m7G-related prognostic lncRNAs by KM analysis and univariate Cox analysis methods. Then, a prognostic risk score model for OSCC were constructed and obtained the formula of the model. Next, coexpression network analysis, risk assessment, and survival analysis of m7G-related prognostic lncRNAs were carried out. In addition, we conducted a comprehensive risk analysis for OSCC patients with high- and low-risk groups and performed independent prognostic analyses and ROC curve analyses to verify the predictive performance of OSCC prognostic risk score model.

However, there are still some limitations in this study, such as the lack of further studies on the important functions and key pathways of different pathological subtypes of OSCC and m7G-related gene [43]. More experimental validation of tissue samples from patients is needed in the future to further validate our new findings.

## Figures and Tables

**Table 1:** Identification of m7G-related prognostic lncRNAs.

lncRNAs	HR	HR.95L	HR.95H	KM_*P* value	Cox_*P* value
TMEM99	1.177014456	1.094434527	1.265825407	0.000111836	1.13*E* − 05
HHLA3	1.167767969	1.075729404	1.267681281	0.007342318	0.000213266
AC010894.1	2.447756883	1.687562963	3.5503942	0.007956686	2.38*E* − 06
AL513550.1	2.097224234	1.463206544	3.005966249	0.002772334	5.52*E* − 05
LINC02541	0.576058399	0.524831944	0.632284835	0.009523161	0.002882225
AL035446.1	1.270188027	1.072263941	1.504645976	0.001398622	0.005652859
MAPKAPK5-AS1	1.517938814	1.224317121	1.881978292	0.006793084	0.000141708
CASC9	1.15835462	1.074073357	1.249249334	0.001601384	0.000136753
FLJ20021	1.062598463	1.019889443	1.107095972	7.95*E* − 05	0.00372095
AC007114.1	0.560783838	0.386005302	0.814699982	0.000430497	0.006336202
AC010326.3	1.374941078	1.101926411	1.715598201	0.005999112	0.004811556
PCCA-DT	1.191786502	1.071410431	1.325687174	0.002625784	0.001239526
AP001505.1	1.124910675	1.037735455	1.21940907	0.000367312	0.004236502
AC068831.5	2.156967923	1.40121821	3.320332685	0.00403799	0.000478102
AC005332.6	1.342011378	1.076790983	1.672557225	0.008783854	0.008830636
HEIH	0.414925538	0.247038478	0.696908446	0.005205462	0.005809747

**Table 2:** Coefficient profiles of the nine m7G-related prognostic lncRNAs.

lncRNAs (Exp*β*)	Coef (*β*)	HR value
HHLA3	0.177050205	1.19369102
AC010894.1	1.226938632	3.410771908
AL513550.1	1.011070188	2.748540896
LINC02541	-0.319316881	0.726645252
AL035446.1	0.301815135	1.352311208
AC007114.1	-0.737018334	0.478538633
AC068831.5	0.793005914	2.210029611
AC005332.6	0.539498029	1.715145692
HEIH	-0.800902563	0.448923599

Calculation formula: Risk score = Exp*β*1 × *β*1 + Exp*β*2 × *β*2 + Exp*β*3 × *β*3 + Exp*βi* × *βi*.

**Table 3:** Coefficient profiles of the nine m7G-related prognostic lncRNAs.

lncRNAs (Exp*β*)	Coef (*β*)	HR value
HHLA3	0.177050205	1.19369102
AC010894.1	1.226938632	3.410771908
AL513550.1	1.011070188	2.748540896
LINC02541	-0.319316881	0.726645252
AL035446.1	0.301815135	1.352311208
AC007114.1	-0.737018334	0.478538633
AC068831.5	0.793005914	2.210029611
AC005332.6	0.539498029	1.715145692
HEIH	-0.800902563	0.448923599

Calculation formula: Risk score = Exp*β*1 × *β*1 + Exp*β*2 × *β*2 + Exp*β*3 × *β*3 + Exp*βi* × *βi*.

## Data Availability

The dataset used and/or analyzed during this study may be granted by contacting the corresponding author.

## Supplementary Materials

Supplementary Materials Supplementary Materials. File m7G-lncRNAs_exp.xls shows the expression matrix of 399 m7G-related lncRNAs. Rows represent m7G-related lncRNA names, and columns represent samples. File co-exp_rel.xls shows the coexpression relationship of m7G-related lncRNAs and m7G-realated mRNAs. The first column represents m7G-realated mRNAs, the second column represents m7G-realated lncRNAs, the third column represents coexpression correlation coefficients, and the fourth column represents the *P* value of the correlation test. File risk.xls presents univariate Cox regression analysis for 16 significant m7G-related prognostic lncRNAs. The first column represents samples, the second column represents the survival time of patients, the third column represents their survival status, and columns 4 to 19 represent m7G-related prognostic lncRNAs. File risk.xls presents the risk scores of nine m7G-related prognostic lncRNAs that constitute the prognostic model. The first column represents samples, the second column represents the survival time of patients, the third column represents their survival status, columns 4 to 12 represent m7G-related prognostic lncRNAs, and columns 13 and 14 represent the risk score and risk grouping for each patient. File coexp_network.xls shows the coexpression relationship between the m7G-related prognostic lncRNAs and mRNAs. The first column represents prognostic m7G-realated mRNAs, the second column represents prognostic m7G-realated lncRNAs, and the third column represents the correlation type.
