# Supplementary material for: The Risk Correlation between N7-Methylguanosine Modification-Related lncRNAs and Survival Prognosis of Oral Squamous Cell Carcinoma Based on Comprehensive Bioinformatics Analysis
Source: Appl Bionics Biomech. 2022 Aug 24;2022:1666792. doi: 10.1155/2022/1666792 (PMC9433249; doi:10.1155/2022/1666792)
Supplement: Supplementary Materials — File m7G-lncRNAs_exp.xls shows the expression matrix of 399 m7G-related lncRNAs. Rows represent m7G-related lncRNA names, and columns represent samples. File co-exp_rel.xls shows the coexpression relationship of m7G-related lncRNAs and m7G-realated mRNAs. The first column represents m7G-realated mRNAs, the second column represents m7G-realated lncRNAs, the third column represents coexpression correlation coefficients, and the fourth column represents the P value of the correlation test. File risk.xls presents univariate Cox regression analysis for 16 significant m7G-related prognostic lncRNAs. The first column represents samples, the second column represents the survival time of patients, the third column represents their survival status, and columns 4 to 19 represent m7G-related prognostic lncRNAs. File risk.xls presents the risk scores of nine m7G-related prognostic lncRNAs that constitute the prognostic model. The first column represents samples, the second column represents the survival time of patients, the third column represents their survival status, columns 4 to 12 represent m7G-related prognostic lncRNAs, and columns 13 and 14 represent the risk score and risk grouping for each patient. File coexp_network.xls shows the coexpression relationship between the m7G-related prognostic lncRNAs and mRNAs. The first column represents prognostic m7G-realated mRNAs, the second column represents prognostic m7G-realated lncRNAs, and the third column represents the correlation type. [file 1666792.f1.zip › coexp_network.pdf]

mRNA	lncRNA	Type
NUDT1	HHLA3	coexpression
NCBP2	AC010894.1	coexpression
SNUPN	AC010894.1	coexpression
NUDT1	AL513550.1	coexpression
NUDT16	AL513550.1	coexpression
EIF4E2	AL513550.1	coexpression
NCBP2	AL513550.1	coexpression
NCBP2	LINC02541	coexpression
SNUPN	LINC02541	coexpression
DCPS	AL035446.1	coexpression
NUDT11	AL035446.1	coexpression
NUDT16L1	AL035446.1	coexpression
NCBP2	AL035446.1	coexpression
NUDT1	AC007114.1	coexpression
NUDT7	AC007114.1	coexpression
NUDT16	AC068831.5	coexpression
NCBP2	AC068831.5	coexpression
NUDT16	AC005332.6	coexpression
NUDT1	HEIH	coexpression
CYFIP1	HEIH	coexpression
EIF4E2	HEIH	coexpression
