# Supplementary material for: The Risk Correlation between N7-Methylguanosine Modification-Related lncRNAs and Survival Prognosis of Oral Squamous Cell Carcinoma Based on Comprehensive Bioinformatics Analysis
Source: Appl Bionics Biomech. 2022 Aug 24;2022:1666792. doi: 10.1155/2022/1666792 (PMC9433249; doi:10.1155/2022/1666792)
Supplement: Supplementary Materials — File m7G-lncRNAs_exp.xls shows the expression matrix of 399 m7G-related lncRNAs. Rows represent m7G-related lncRNA names, and columns represent samples. File co-exp_rel.xls shows the coexpression relationship of m7G-related lncRNAs and m7G-realated mRNAs. The first column represents m7G-realated mRNAs, the second column represents m7G-realated lncRNAs, the third column represents coexpression correlation coefficients, and the fourth column represents the P value of the correlation test. File risk.xls presents univariate Cox regression analysis for 16 significant m7G-related prognostic lncRNAs. The first column represents samples, the second column represents the survival time of patients, the third column represents their survival status, and columns 4 to 19 represent m7G-related prognostic lncRNAs. File risk.xls presents the risk scores of nine m7G-related prognostic lncRNAs that constitute the prognostic model. The first column represents samples, the second column represents the survival time of patients, the third column represents their survival status, columns 4 to 12 represent m7G-related prognostic lncRNAs, and columns 13 and 14 represent the risk score and risk grouping for each patient. File coexp_network.xls shows the coexpression relationship between the m7G-related prognostic lncRNAs and mRNAs. The first column represents prognostic m7G-realated mRNAs, the second column represents prognostic m7G-realated lncRNAs, and the third column represents the correlation type. [file 1666792.f1.zip › co-exp_rel.pdf]

ARGgene	lncRNA	cor	pvalue
NUDT11	LINC01116	0.4628223	5.48E-05
NUDT4	LINC01116	0.449235	9.58E-05
NCBP2	TMEM99	0.5224754	3.49E-06
LSM1	TMEM99	0.6074331	2.47E-08
NUDT1	SNHG11	0.4413402	0.0001311
NUDT16L1	SNHG11	0.4883225	1.80E-05
NCBP2	SNHG11	0.6124134	1.76E-08
NUDT16	DLGAP1-AS1	0.4483726	9.92E-05
NCBP2	DLGAP1-AS1	0.5006936	1.01E-05
NUDT16L1	NINJ2-AS1	0.4670501	4.58E-05
NUDT7	NINJ2-AS1	0.563963	3.70E-07
NCBP2	NINJ2-AS1	0.4197336	0.0002979
NUDT1	ZFAS1	0.7784308	2.18E-15
NUDT5	ZFAS1	0.4884855	1.79E-05
CYFIP1	ZFAS1	-0.431424	0.0001924
EIF4E2	ZFAS1	0.6315632	4.57E-09
NCBP2	ZFAS1	0.4397295	0.0001397
EIF3D	ZFAS1	0.4917627	1.54E-05
NUDT16	EIF3J-DT	0.5066053	7.65E-06
NCBP2	EIF3J-DT	0.5614896	4.26E-07
SNUPN	EIF3J-DT	0.5977816	4.66E-08
NUDT16	FLJ37453	0.4090603	0.0004381
NUDT16L1	FLJ37453	0.544894	1.08E-06
NCBP2	FLJ37453	0.5688599	2.78E-07
EIF4G3	FLJ37453	0.4427058	0.0001243
NUDT16L1	LINC02870	0.4572048	6.92E-05
NCBP2	LINC02870	0.4204728	0.0002899
NUDT7	TP53TG1	0.4035826	0.0005314
NUDT16L1	C5orf38	0.4952108	1.31E-05
NUDT1	KTN1-AS1	0.667033	2.89E-10
EIF4E	KTN1-AS1	-0.400212	0.0005974
EIF3D	KTN1-AS1	0.4977841	1.16E-05
DCP2	AC091057.1	0.4141004	0.0003658
CYFIP2	AC091057.1	0.4729791	3.55E-05
NUDT16L1	PAX8-AS1	0.4274578	0.0002236
NUDT16L1	LINC01560	0.423532	0.0002589
NCBP2	LINC01560	0.515799	4.88E-06
LSM1	LINC01560	0.4037179	0.0005289
NUDT1	CH17-340M2	0.8941923	1.98E-25
EIF4E2	CH17-340M2	0.7506277	7.34E-14
NUDT1	HHLA3	0.4482391	9.97E-05
NUDT1	SNHG12	0.6059084	2.73E-08
NUDT5	SNHG12	0.4233659	0.0002605
EIF4E2	SNHG12	0.4292132	0.0002092
DCPS	NBR2	0.4086021	0.0004453
NUDT16	NBR2	0.4157675	0.0003443
NUDT7	PDCD4-AS1	0.4388217	0.0001447
NUDT16	AC083799.1	0.4349831	0.0001679
NCBP2	AC083799.1	0.406549	0.0004788
NUDT5	SNHG5	0.4856561	2.03E-05

NUDT11	ARRDC1-AS1	0.4000665	0.0006004
NUDT16	ARRDC1-AS1	0.4539856	7.90E-05
NCBP1	ARRDC1-AS1	0.5999385	4.05E-08
NCBP2	ARRDC1-AS1	0.5408476	1.34E-06
NUDT16	LINC00963	0.619618	1.07E-08
NCBP2	LINC00963	0.5256074	2.98E-06
DCP2	PSMB8-AS1	0.4138886	0.0003686
EIF4E3	PSMB8-AS1	0.4165257	0.000335
IFIT5	PSMB8-AS1	0.5375074	1.60E-06
NUDT7	SNHG32	0.4092023	0.0004359
NUDT1	MIR1915HG	0.8514202	9.85E-21
EIF4E2	MIR1915HG	0.7712556	5.65E-15
LARP1	HCP5	0.5050321	8.25E-06
IFIT5	HCP5	0.6600125	5.13E-10
GEMIN5	LINC02693	0.4358703	0.0001622
NUDT11	AC016747.1	0.4032151	0.0005382
NUDT4	AC016747.1	0.5019841	9.54E-06
NCBP3	AC016747.1	0.4140904	0.0003659
NUDT16	APTR	0.5544606	6.35E-07
NCBP2	APTR	0.6637314	3.79E-10
SNUPN	APTR	0.5268199	2.80E-06
SNUPN	AC087491.1	0.5651007	3.46E-07
NUDT1	AC010168.1	0.6925192	3.11E-11
EIF4E2	AC010168.1	0.5845556	1.08E-07
LARP1	AC010168.1	-0.407033	0.0004707
NCBP2	AC010168.1	0.4259645	0.0002365
NUDT16	CD27-AS1	0.5169722	4.61E-06
NCBP2	CD27-AS1	0.5606321	4.48E-07
SNUPN	CD27-AS1	0.6258542	6.90E-09
NUDT16	DHRS4-AS1	0.461586	5.77E-05
NCBP2	DHRS4-AS1	0.5503844	7.97E-07
SNUPN	DHRS4-AS1	0.5267328	2.81E-06
NUDT16L1	EXOC3-AS1	0.6309249	4.78E-09
NCBP2	EXOC3-AS1	0.4255597	0.0002401
NCBP1	AL441992.1	0.4444094	0.0001162
EIF4E3	HLA-DQB1-A	0.4014024	0.0005733
NUDT11	LINC00205	0.5382667	1.54E-06
NCBP1	LINC00205	0.4930121	1.45E-05
NUDT1	EPB41L4A-A	0.409246	0.0004352
CYFIP1	EPB41L4A-A	-0.432864	0.0001821
NUDT7	HAGLR	0.4215692	0.0002785
NCBP2	HAGLR	0.4989453	1.10E-05
NUDT16	LINC01133	0.4153248	0.0003499
NCBP2	LINC01133	0.4906452	1.62E-05
NUDT16	AC096677.1	0.4446972	0.0001148
NUDT16L1	AC096677.1	0.5293202	2.46E-06
NUDT7	AC096677.1	0.5777973	1.63E-07
NCBP2	AC096677.1	0.5357439	1.76E-06
SNUPN	LINC00885	0.4652334	4.95E-05
NUDT16	MRPL20-AS1	0.4695538	4.12E-05
NUDT16L1	MRPL20-AS1	0.4871828	1.89E-05

NCBP2	MRPL20-AS1	0.4366366	0.0001575
NUDT1	AC073957.1	0.6609271	4.77E-10
EIF4E2	AC073957.1	0.4814994	2.44E-05
NUDT1	AL590617.2	0.7426426	1.85E-13
EIF4E2	AL590617.2	0.6446372	1.72E-09
NCBP2	AL590617.2	0.486117	1.99E-05
NUDT16L1	LINC01770	0.5231181	3.38E-06
NUDT16L1	LINC01980	0.4447583	0.0001146
DCP2	FGD5-AS1	0.5894889	7.91E-08
NCBP3	FGD5-AS1	0.4966767	1.22E-05
DCPS	P3H2-AS1	0.4588563	6.47E-05
LSM1	P3H2-AS1	0.4664544	4.70E-05
NCBP2	LINC00623	0.4199983	0.0002951
IFIT5	LINC00623	0.4009951	0.0005814
NUDT16	BAIAP2-DT	0.4432596	0.0001216
NUDT16L1	BAIAP2-DT	0.496082	1.26E-05
NUDT7	BAIAP2-DT	0.406458	0.0004804
NCBP2	BAIAP2-DT	0.4774208	2.93E-05
LSM1	BAIAP2-DT	0.4081026	0.0004533
NUDT1	NUP50-DT	0.806716	3.46E-17
EIF4E2	NUP50-DT	0.6584202	5.84E-10
NUDT1	AL354836.1	0.571718	2.35E-07
NCBP2	AL354836.1	0.5269846	2.78E-06
EIF3D	AL354836.1	0.4160351	0.000341
NCBP2	HAGLROS	0.4447985	0.0001144
LSM1	HAGLROS	0.4934002	1.43E-05
NUDT1	Z93930.2	0.645822	1.57E-09
EIF4E2	Z93930.2	0.4452107	0.0001125
NCBP2	Z93930.2	0.5231356	3.38E-06
NCBP2	AC010894.1	0.5476182	9.29E-07
SNUPN	AC010894.1	0.432805	0.0001825
NUDT11	DANCR	0.4188543	0.0003077
NUDT16L1	DANCR	0.4935682	1.41E-05
NUDT5	DANCR	0.462497	5.56E-05
NCBP2	DANCR	0.5886866	8.32E-08
NUDT1	AC099066.2	0.5131483	5.56E-06
EIF4E2	AC099066.2	0.481717	2.42E-05
NUDT16L1	SOX21-AS1	0.412248	0.000391
LSM1	SOX21-AS1	0.4077751	0.0004585
DCP2	AL390728.4	0.5450757	1.07E-06
NCBP3	AL390728.4	0.5431577	1.18E-06
NUDT16	MELTF-AS1	0.6253915	7.13E-09
NUDT16L1	MELTF-AS1	0.4981767	1.14E-05
NCBP2	MELTF-AS1	0.6920999	3.23E-11
NUDT7	AL355607.1	0.4568106	7.04E-05
SNUPN	HCG11	0.4115669	0.0004006
NUDT16L1	PCAT6	0.4174765	0.0003236
IFIT5	PCAT6	-0.466011	4.79E-05
NUDT1	LINC00392	0.8713463	1.04E-22
EIF4E2	LINC00392	0.7459232	1.27E-13
DCPS	AC106875.1	0.4297396	0.0002051

LSM1	AC106875.1	0.4479479	0.0001009
NUDT1	AL513550.1	0.5422221	1.25E-06
NUDT16	AL513550.1	0.4112277	0.0004055
EIF4E2	AL513550.1	0.4856506	2.03E-05
NCBP2	AL513550.1	0.6406686	2.33E-09
NCBP2	AC093159.1	0.4176154	0.000322
LSM1	AC093159.1	0.487032	1.91E-05
NUDT7	AC064807.1	0.4449864	0.0001135
NCBP2	LINC02041	0.4184816	0.0003119
NUDT1	ZFAND2A-DT	0.4002151	0.0005973
NUDT16L1	ZFAND2A-DT	0.4282014	0.0002174
NCBP2	ZFAND2A-DT	0.4728488	3.57E-05
NCBP3	ANKRD10-IT	0.457743	6.77E-05
NUDT1	AC046143.1	0.8550043	4.57E-21
EIF4E2	AC046143.1	0.7032911	1.13E-11
NCBP2	AC046143.1	0.4586665	6.52E-05
NUDT1	MYOSLID	0.4034179	0.0005344
NUDT11	MYOSLID	0.6271072	6.30E-09
EIF4E2	MYOSLID	0.5073014	7.40E-06
EIF3D	MYOSLID	0.5422877	1.24E-06
NUDT1	MAST4-AS1	0.4419153	0.0001282
NUDT16L1	TRPM2-AS	0.4570887	6.96E-05
LSM1	TRPM2-AS	0.4374485	0.0001526
NUDT1	AL162231.2	0.4593433	6.34E-05
NUDT1	PRRT3-AS1	0.569323	2.70E-07
NUDT5	PRRT3-AS1	0.4676034	4.48E-05
EIF4E2	PRRT3-AS1	0.4260658	0.0002356
DCPS	NFE4	0.4028547	0.000545
LSM1	NFE4	0.5824066	1.23E-07
CYFIP2	SERPINB9P1	0.4277658	0.000221
NUDT4	AP000695.1	0.404363	0.0005171
DCP2	AC021078.1	0.4601911	6.12E-05
NUDT16L1	AL604028.1	0.4079994	0.0004549
NUDT7	AL604028.1	0.4826027	2.33E-05
NCBP2	LINC02541	0.5058871	7.92E-06
SNUPN	LINC02541	0.520659	3.83E-06
LSM1	AC005392.2	0.4180397	0.000317
LSM1	AC074389.2	0.527431	2.71E-06
NUDT16L1	COA6-AS1	0.5461983	1.00E-06
NCBP2	COA6-AS1	0.6228894	8.51E-09
NUDT1	TMEM44-AS1	0.4559703	7.28E-05
NUDT16	TMEM44-AS1	0.4789404	2.74E-05
NUDT16L1	TMEM44-AS1	0.5150079	5.08E-06
NCBP2	TMEM44-AS1	0.7458816	1.28E-13
NUDT16	PIK3CD-AS2	0.4176311	0.0003218
NUDT16L1	PIK3CD-AS2	0.4352506	0.0001662
NCBP2	PIK3CD-AS2	0.5140657	5.32E-06
NUDT1	DCST1-AS1	0.4093148	0.0004342
LSM1	DCST1-AS1	0.4173284	0.0003253
NUDT16L1	AC012313.1	0.540921	1.34E-06
NCBP2	AC012313.1	0.4153243	0.0003499

NUDT16L1	MHENCR	0.5701221	2.58E-07
NUDT7	MHENCR	0.453056	8.21E-05
NCBP2	MHENCR	0.4582336	6.64E-05
DCPS	EMSLR	0.4744385	3.34E-05
NUDT11	AC093673.1	0.4075774	0.0004618
NCBP2	AC093673.1	0.4703218	3.99E-05
NUDT16L1	AL354892.2	0.4801468	2.60E-05
NUDT7	AL354892.2	0.4708574	3.89E-05
NCBP2	AL354892.2	0.4538099	7.96E-05
NCBP2	LINC00665	0.5073477	7.38E-06
NUDT1	SNHG15	0.7709941	5.85E-15
EIF4E2	SNHG15	0.5778329	1.63E-07
EIF3D	SNHG15	0.4222065	0.000272
NUDT1	SNHG7	0.4874601	1.87E-05
NCBP2	SNHG7	0.4976403	1.17E-05
NUDT1	AC016876.1	0.5111897	6.12E-06
NUDT11	AP000695.2	0.4228265	0.0002658
NUDT4	AP000695.2	0.5096119	6.61E-06
NUDT11	LINC01503	0.4364543	0.0001586
NCBP1	LINC01503	0.437377	0.0001531
NCBP2	LINC01503	0.499798	1.06E-05
EIF3D	LINC01503	0.4215106	0.0002791
NUDT16L1	AC074117.1	0.4433941	0.0001209
NCBP2	AC074117.1	0.4083143	0.0004499
NCBP3	AC074117.1	0.40958	0.0004301
DCPS	AL035446.1	0.4133039	0.0003764
NUDT11	AL035446.1	0.4662744	4.74E-05
NUDT16L1	AL035446.1	0.4073714	0.0004651
NCBP2	AL035446.1	0.4865176	1.95E-05
NUDT1	MAPKAPK5-A	0.4554829	7.43E-05
NUDT5	MAPKAPK5-A	0.413547	0.0003731
NCBP2	MAPKAPK5-A	0.6563164	6.91E-10
SNUPN	MAPKAPK5-A	0.4483103	9.94E-05
NUDT1	AL139289.1	0.4303465	0.0002005
NUDT7	AL139289.1	0.420904	0.0002854
DCPS	GAS5	0.4363498	0.0001593
NCBP2	GAS5	0.4378832	0.0001501
EIF3D	GAS5	0.4194465	0.0003011
NCBP2	AC128709.2	0.4542303	7.82E-05
LSM1	AC128709.2	0.4844728	2.14E-05
SNUPN	AC128709.2	0.4992691	1.08E-05
NCBP2	MCF2L-AS1	0.4699983	4.04E-05
SNUPN	MCF2L-AS1	0.4595892	6.27E-05
DCPS	LINC01278	0.4657683	4.84E-05
NUDT7	LINC01278	0.4001193	0.0005993
AGO2	AC073046.1	0.5149714	5.09E-06
CYFIP1	AC073046.1	0.419522	0.0003003
CYFIP2	LINC01871	0.4417672	0.0001289
EIF4E3	LINC01871	0.5278067	2.66E-06
IFIT5	LINC01871	0.5914593	6.99E-08
NUDT1	ASH1L-AS1	0.4964818	1.24E-05

NUDT16L1	ASH1L-AS1	0.5361231	1.72E-06
NCBP2	ASH1L-AS1	0.5983541	4.49E-08
NUDT16	AL354766.2	0.45268	8.33E-05
DCPS	SLC12A9-AS	0.4447913	0.0001144
NUDT7	SLC12A9-AS	0.4394094	0.0001414
CYFIP2	LINC02195	0.5217048	3.63E-06
EIF4E3	LINC02195	0.4591167	6.40E-05
IFIT5	LINC02195	0.5681445	2.90E-07
NUDT1	GS1-124K5.	0.44829	9.95E-05
NUDT16L1	GS1-124K5.	0.4258591	0.0002374
NCBP2	GS1-124K5.	0.6380528	2.83E-09
NUDT11	FOXD2-AS1	0.4868895	1.92E-05
NCBP2	FOXD2-AS1	0.4841271	2.17E-05
NCBP2	BX293535.1	0.425545	0.0002402
SNUPN	BX293535.1	0.4825509	2.33E-05
NUDT16L1	LINC01637	0.4898465	1.68E-05
NUDT7	LINC01637	0.4622325	5.62E-05
NCBP2	LINC01637	0.4003449	0.0005947
DCPS	LINC00857	0.422432	0.0002697
IFIT5	TTLL11-IT1	0.4945746	1.35E-05
EIF4E3	AC022034.1	0.4824384	2.34E-05
IFIT5	AC022034.1	0.4117737	0.0003977
NUDT1	DGUOK-AS1	0.7832198	1.13E-15
EIF4E2	DGUOK-AS1	0.632216	4.35E-09
NUDT11	AL355574.1	0.4486131	9.82E-05
NUDT16	AL355574.1	0.5440904	1.13E-06
NCBP1	AL355574.1	0.5799441	1.43E-07
NCBP2	AL355574.1	0.591278	7.07E-08
NUDT1	LINC01589	0.5344756	1.88E-06
EIF4E2	LINC01589	0.4417345	0.0001291
CYFIP2	LINC00707	0.4564374	7.15E-05
NCBP2	BX470102.1	0.4776348	2.90E-05
SNUPN	BX470102.1	0.4897927	1.68E-05
LSM1	LINC02031	0.4860893	1.99E-05
NUDT1	KMT2E-AS1	0.6444145	1.75E-09
NUDT16L1	KMT2E-AS1	0.4160728	0.0003406
NUDT5	KMT2E-AS1	0.4247411	0.0002475
EIF4E2	KMT2E-AS1	0.4874572	1.87E-05
NCBP2	KMT2E-AS1	0.4299505	0.0002035
NUDT1	PRR34-AS1	0.7815922	1.41E-15
NUDT5	PRR34-AS1	0.4486194	9.82E-05
CYFIP1	PRR34-AS1	-0.423673	0.0002576
EIF4E2	PRR34-AS1	0.6634327	3.88E-10
EIF3D	PRR34-AS1	0.5411182	1.32E-06
NUDT5	SNHG3	0.431618	0.000191
NUDT1	LASTR	0.4928873	1.46E-05
EIF4E2	LASTR	0.4425388	0.0001251
NUDT16	AL591895.1	0.6310668	4.74E-09
NCBP2	AL591895.1	0.5583284	5.11E-07
LSM1	AL591895.1	0.4546986	7.67E-05
SNUPN	AL591895.1	0.5499533	8.17E-07

NUDT1	AC082651.3	0.4650366	4.99E-05
NUDT7	AC082651.3	0.4477999	0.0001015
NCBP2	AC082651.3	0.4153527	0.0003496
NCBP3	LINC01214	-0.492867	1.46E-05
NCBP3	ATP1B3-AS1	0.478876	2.75E-05
NUDT16L1	AC147067.1	0.5282532	2.60E-06
NUDT1	AC108676.1	0.4921469	1.51E-05
EIF4E2	AC108676.1	0.4230627	0.0002635
DCP2	NEAT1	0.4316188	0.000191
NUDT16	SCAMP1-AS1	0.4129789	0.0003808
SNUPN	SCAMP1-AS1	0.4172338	0.0003265
NCBP3	RAD51-AS1	0.4083027	0.00045
LSM1	RAD51-AS1	0.4006243	0.0005889
NUDT1	SNHG6	0.7338488	4.94E-13
NUDT5	SNHG6	0.5378927	1.57E-06
CYFIP1	SNHG6	-0.490218	1.65E-05
EIF4E2	SNHG6	0.5594949	4.78E-07
NCBP2	SNHG6	0.4422664	0.0001264
EIF3D	SNHG6	0.4894634	1.71E-05
NUDT16L1	AC016065.1	0.4217583	0.0002765
SNUPN	SBF2-AS1	0.4410024	0.0001329
NCBP3	AL049840.2	0.5353634	1.80E-06
LSM1	MIR210HG	0.411168	0.0004064
NUDT7	ZBED5-AS1	0.4113895	0.0004032
NUDT1	GPRC5D-AS1	0.6398533	2.47E-09
NUDT5	GPRC5D-AS1	0.4130133	0.0003804
EIF4E2	GPRC5D-AS1	0.5709228	2.46E-07
NUDT7	NNT-AS1	0.4527504	8.31E-05
NUDT7	AC114956.1	0.4289816	0.0002111
NUDT1	TRIM52-AS1	0.6178005	1.22E-08
NUDT16L1	USP46-DT	0.4630426	5.43E-05
NUDT5	USP46-DT	0.4084984	0.0004469
NUDT1	AC093895.1	0.5206263	3.84E-06
NUDT5	AC093895.1	0.4692395	4.17E-05
EIF4E2	AC093895.1	0.5364	1.70E-06
NCBP2	AC093895.1	0.4403444	0.0001363
NUDT1	IRX4-AS1	0.6965111	2.15E-11
EIF4E2	IRX4-AS1	0.5307747	2.28E-06
NUDT16	CASC9	0.4355241	0.0001644
NUDT16L1	CASC9	0.4025409	0.000551
NCBP2	CASC9	0.525057	3.06E-06
NUDT16L1	AC034231.1	0.4981164	1.14E-05
NUDT1	AL589765.4	0.5343036	1.90E-06
NUDT11	LINC00942	0.5878481	8.77E-08
NCBP1	LINC00942	0.408398	0.0004485
DCPS	LINC02762	0.4258666	0.0002373
NUDT7	LINC02762	0.4201268	0.0002937
NUDT7	SNHG18	0.444881	0.000114
AGO2	AC125807.2	0.4891328	1.73E-05
NCBP2	THAP9-AS1	0.5397033	1.43E-06
CYFIP2	AC020661.1	0.4320779	0.0001877

GEMIN5	LINC00958	0.4717414	3.75E-05
EIF4G3	FAM160A1-I	0.4803832	2.57E-05
LSM1	LINC01932	0.4635339	5.32E-05
NUDT11	AC124067.2	0.4093336	0.0004339
NCBP2	AC124067.2	0.4982463	1.14E-05
LSM1	AC124067.2	0.5039449	8.69E-06
SNUPN	AC124067.2	0.5213131	3.71E-06
NUDT1	GASAL1	0.4238855	0.0002555
NUDT7	MINCR	0.4020395	0.0005607
NCBP2	MINCR	0.4040765	0.0005223
NCBP2	OTUD6B-AS1	0.5001865	1.04E-05
EIF4E3	AC007991.2	0.4902379	1.65E-05
IFIT5	AC007991.2	0.4382963	0.0001477
IFIT5	TNFRSF10A-	0.4848042	2.11E-05
NUDT1	AP001207.3	0.4542942	7.80E-05
EIF4E	AC009902.2	-0.447918	0.000101
NCBP2	CASC19	0.5116407	5.99E-06
NUDT4	AC091563.1	0.4303545	0.0002004
NUDT16	AL354920.1	0.4212939	0.0002813
NCBP2	AL354920.1	0.4116267	0.0003998
LSM1	AL354920.1	0.4718446	3.73E-05
NUDT1	FLJ20021	0.8070393	3.29E-17
EIF4E2	FLJ20021	0.7356827	4.04E-13
EIF3D	FLJ20021	0.4799465	2.62E-05
DCPS	AP003068.2	0.4921869	1.51E-05
NUDT5	WAC-AS1	0.5273121	2.73E-06
NCBP2	WAC-AS1	0.5397057	1.43E-06
NUDT7	AP001372.2	0.4083738	0.0004489
NUDT1	AP003390.1	0.560562	4.50E-07
NCBP2	AP003390.1	0.4054014	0.0004986
EIF3D	AP003390.1	0.4203347	0.0002914
SNUPN	AC136475.3	0.5917772	6.85E-08
NUDT7	AP003119.2	0.406738	0.0004757
NUDT1	AP002360.1	0.5962759	5.14E-08
EIF4E2	AP002360.1	0.4605164	6.04E-05
EIF3D	AP002360.1	0.5342651	1.90E-06
NUDT16L1	TOLLIP-AS1	0.5949842	5.58E-08
NUDT7	TOLLIP-AS1	0.445387	0.0001117
NCBP2	TOLLIP-AS1	0.4768664	3.00E-05
CYFIP2	AC090559.1	0.5039153	8.70E-06
EIF4E3	AC090559.1	0.5067889	7.58E-06
NUDT16L1	SNHG9	0.4721297	3.69E-05
NCBP2	SNHG9	0.4282641	0.0002169
NUDT16L1	AC109322.1	0.5570396	5.49E-07
NCBP2	AC109322.1	0.4582476	6.63E-05
NUDT1	AP006621.3	0.8344115	2.92E-19
NUDT5	AP006621.3	0.4488996	9.71E-05
EIF4E2	AP006621.3	0.6960894	2.24E-11
EIF3D	AP006621.3	0.4167834	0.0003319
SNUPN	AP001830.1	0.4690114	4.22E-05
NUDT1	AC104031.1	0.6346326	3.65E-09

EIF4E2	AC104031.1	0.463856	5.25E-05
LARP1	AC104031.1	-0.414247	0.0003638
DCPS	SNHG1	0.4214267	0.0002799
NCBP2	SNHG1	0.4463567	0.0001075
EIF4E3	LINC02446	0.6258723	6.89E-09
IFIT5	LINC02446	0.5542761	6.42E-07
NUDT1	URB1-AS1	0.5578004	5.26E-07
EIF4E2	URB1-AS1	0.4829664	2.29E-05
NCBP2	URB1-AS1	0.499693	1.06E-05
NUDT16L1	ZBTB11-AS1	0.4938227	1.40E-05
NCBP2	ZBTB11-AS1	0.4731349	3.53E-05
NUDT1	MIR200CHG	0.6030317	3.31E-08
NUDT5	MIR200CHG	0.4581672	6.65E-05
EIF4E2	MIR200CHG	0.5418813	1.27E-06
NUDT16	TMPO-AS1	0.5556011	5.96E-07
NCBP3	AL928654.2	0.4264892	0.0002319
DCP2	PSMA3-AS1	0.4272989	0.0002249
NUDT16	PSMA3-AS1	0.4788005	2.76E-05
NCBP3	PSMA3-AS1	0.4911398	1.58E-05
SNUPN	PSMA3-AS1	0.4334188	0.0001783
NUDT1	G2E3-AS1	0.6585224	5.79E-10
EIF4E2	G2E3-AS1	0.5768507	1.73E-07
NCBP2	G2E3-AS1	0.4064821	0.00048
NCBP2	LBX2-AS1	0.5219832	3.58E-06
LSM1	LBX2-AS1	0.5148997	5.10E-06
NCBP2	CPNE8-AS1	0.4673879	4.52E-05
LSM1	CPNE8-AS1	0.4563327	7.18E-05
NUDT1	AC025575.2	0.4582293	6.64E-05
NUDT5	AC025575.2	0.4460209	0.000109
EIF4E2	AC025575.2	0.4597853	6.22E-05
NUDT16	AC009779.2	0.5626642	3.99E-07
NUDT4	AC009779.2	0.4609006	5.94E-05
NCBP2	AC009779.2	0.6019479	3.55E-08
SNUPN	AC009779.2	0.5041846	8.59E-06
NCBP3	AL512791.1	0.4049126	0.0005072
SNUPN	LINC00640	0.5430967	1.19E-06
LSM1	RHOXF1-AS1	0.6479073	1.34E-09
NUDT1	ARHGAP5-AS	0.5540698	6.49E-07
EIF4E2	ARHGAP5-AS	0.4467107	0.000106
LARP1	ARHGAP5-AS	-0.52153	3.67E-06
NUDT1	LINC02310	0.4314432	0.0001923
EIF4E2	LINC02310	0.4074595	0.0004637
LARP1	LINC02310	-0.401837	0.0005647
EIF3D	LINC02310	0.452504	8.39E-05
NCBP2	LINC02820	0.4928241	1.46E-05
LSM1	LINC02820	0.4868493	1.92E-05
NUDT1	FOXN3-AS1	0.8826608	5.49E-24
EIF4E2	FOXN3-AS1	0.7270698	1.03E-12
SNUPN	AL049870.3	0.401352	0.0005743
NUDT16	LINC00519	0.4112489	0.0004052
NCBP2	LINC00519	0.4410799	0.0001325

SNUPN	LINC00519	0.5959433	5.25E-08
DCPS	AL121820.2	0.4310278	0.0001953
NUDT16L1	AL121820.2	0.4361388	0.0001606
LSM1	AL121820.2	0.48567	2.03E-05
NUDT7	AC023906.2	0.4875874	1.86E-05
NCBP2	AC023906.2	0.4145685	0.0003596
LSM1	AC023906.2	0.5192692	4.11E-06
NUDT7	AC015660.1	0.4351244	0.000167
EIF4G3	AC108449.2	0.5379534	1.57E-06
NUDT1	AC087612.1	0.669875	2.28E-10
NUDT5	AC087612.1	0.4518847	8.61E-05
EIF4E2	AC087612.1	0.4893115	1.72E-05
DCPS	AC023906.5	0.4626679	5.52E-05
NUDT11	AC023906.5	0.69466	2.55E-11
NUDT7	AC012640.2	0.4409483	0.0001332
NUDT16L1	AC009113.1	0.5273748	2.72E-06
NUDT7	AC009113.1	0.4931406	1.44E-05
NUDT16L1	AL050341.2	0.5895479	7.89E-08
NCBP2	AL050341.2	0.4559459	7.29E-05
NUDT16L1	AL133338.1	0.4269611	0.0002278
NUDT1	AC040169.1	0.8210471	3.24E-18
NUDT5	AC040169.1	0.4193798	0.0003018
EIF4E2	AC040169.1	0.6778936	1.15E-10
NUDT16	AC104794.3	0.5366496	1.68E-06
EIF4G3	AC104794.3	0.4630047	5.44E-05
NUDT16L1	LINC02846	0.4953161	1.30E-05
NUDT16	CD2BP2-DT	0.4105104	0.0004161
NUDT16L1	CD2BP2-DT	0.4293457	0.0002082
NCBP2	CD2BP2-DT	0.5834727	1.15E-07
NUDT16L1	AL035071.1	0.4055627	0.0004957
NUDT1	SNHG19	0.6949537	2.49E-11
NUDT16L1	SNHG19	0.4529165	8.25E-05
NUDT5	SNHG19	0.4893497	1.72E-05
EIF4E2	SNHG19	0.5366695	1.68E-06
NCBP2	SNHG19	0.5042516	8.56E-06
NUDT16	LINC02562	0.4316019	0.0001911
NCBP2	LINC02562	0.45523	7.51E-05
LSM1	LINC02562	0.4101891	0.0004209
SNUPN	LINC02562	0.5334588	1.98E-06
EIF4E2	LINC01882	0.4717805	3.74E-05
NUDT16L1	AC132872.1	0.4557881	7.34E-05
NUDT16L1	AC011374.1	0.6351477	3.51E-09
NUDT7	AC011374.1	0.4135914	0.0003725
NUDT1	AL118516.1	0.4082244	0.0004513
NUDT16L1	AL118516.1	0.4649333	5.01E-05
NCBP2	AL118516.1	0.4713582	3.81E-05
IFIT5	AL118516.1	-0.407466	0.0004636
LSM1	DOCK9-DT	0.4199141	0.000296
NUDT16	AC092718.4	0.4475035	0.0001027
NUDT4	AL512274.1	-0.479336	2.69E-05
NCBP2	AL512274.1	-0.437183	0.0001542

CYFIP2	LINC02188	0.4989197	1.10E-05
NCBP2	SPINT1-AS1	0.4125884	0.0003862
LSM1	SPINT1-AS1	0.5997729	4.09E-08
SNUPN	SPINT1-AS1	0.4342051	0.000173
NUDT16	Z95115.1	0.4843108	2.16E-05
NCBP2	Z95115.1	0.7038571	1.07E-11
EIF4E2	AL031058.1	0.4444306	0.0001161
DCPS	VPS9D1-AS1	0.4833813	2.25E-05
GEMIN5	VPS9D1-AS1	0.4639558	5.23E-05
LSM1	LINC01003	0.4589654	6.44E-05
NCBP2	AC026471.4	0.5356704	1.77E-06
IFIT5	AL596244.1	0.6360019	3.30E-09
NUDT1	LINC02582	0.4523941	8.43E-05
NCBP2	LOXL1-AS1	0.5878949	8.75E-08
SNUPN	LOXL1-AS1	0.43288	0.000182
NUDT1	MIR193BHG	0.5627642	3.96E-07
EIF4E2	MIR193BHG	0.5173183	4.53E-06
NUDT1	AC007114.1	0.4580142	6.70E-05
NUDT7	AC007114.1	0.4338152	0.0001756
NCBP2	LINC00667	0.4175343	0.0003229
NUDT16	AC080037.2	0.5515875	7.46E-07
NUDT7	AC080037.2	0.4097186	0.000428
NCBP2	AC080037.2	0.4341904	0.0001731
LSM1	AC080037.2	0.444973	0.0001136
SNUPN	AC080037.2	0.4494006	9.52E-05
NUDT16	AC015922.2	0.4685882	4.29E-05
NUDT16L1	MAFG-DT	0.5103139	6.39E-06
NCBP2	MAFG-DT	0.4954164	1.30E-05
EIF3D	AC004585.1	0.4507171	9.02E-05
NUDT1	SNHG25	0.8450298	3.69E-20
NUDT5	SNHG25	0.4399016	0.0001387
EIF4E2	SNHG25	0.7065016	8.30E-12
EIF3D	SNHG25	0.4579557	6.71E-05
NCBP3	AC093484.4	0.4990711	1.09E-05
NUDT1	ILF3-DT	0.5702276	2.56E-07
NUDT16L1	ILF3-DT	0.4713187	3.82E-05
EIF4E2	ILF3-DT	0.4575816	6.82E-05
NCBP2	ILF3-DT	0.5911264	7.14E-08
NUDT1	LINC01842	0.5670928	3.08E-07
EIF4E2	LINC01842	0.4275458	0.0002228
NUDT1	AC011472.2	0.7909605	3.76E-16
EIF4E2	AC011472.2	0.6782862	1.11E-10
EIF4E	AP005482.2	0.4114855	0.0004018
NCBP2	AP005482.2	0.4837442	2.21E-05
NCBP2	AC022031.2	0.4341235	0.0001735
NUDT1	CEBPA-DT	0.415217	0.0003513
NUDT16L1	CEBPA-DT	0.5043104	8.54E-06
NCBP2	CEBPA-DT	0.4308156	0.0001969
NUDT1	AC027307.2	0.546029	1.01E-06
NUDT16	SNHG30	0.4626922	5.51E-05
NUDT16L1	SNHG30	0.4109861	0.000409

NCBP2	SNHG30	0.6371495	3.03E-09
NUDT1	AC036176.1	0.49802	1.15E-05
GEMIN5	AP001542.3	-0.416664	0.0003333
LARP1	AP001542.3	-0.417317	0.0003255
EIF4G3	CIRBP-AS1	0.4302345	0.0002013
NUDT1	AC020916.1	0.5967609	4.98E-08
EIF4E2	AC020916.1	0.4902451	1.65E-05
NCBP3	AC008735.2	0.453994	7.90E-05
NCBP3	AC024075.1	0.4978597	1.16E-05
EIF4G3	AC024075.1	0.5528723	6.94E-07
CYFIP2	CARD8-AS1	0.4139729	0.0003674
EIF4G3	AC008764.2	0.5306007	2.30E-06
NUDT5	AC005261.1	0.4042233	0.0005196
NUDT4	LINC02560	-0.431968	0.0001885
NUDT1	IGFL2-AS1	0.5905202	7.42E-08
EIF4E2	IGFL2-AS1	0.4177798	0.00032
LARP1	IGFL2-AS1	-0.435168	0.0001667
LSM1	AL121761.1	0.4275086	0.0002231
NUDT16L1	MAN1B1-DT	0.4236694	0.0002576
NCBP2	MAN1B1-DT	0.4663833	4.72E-05
NUDT16	AC024075.2	0.4386039	0.0001459
NCBP2	AC024075.2	0.4096266	0.0004294
NUDT1	RPARP-AS1	0.7945133	2.23E-16
NUDT5	RPARP-AS1	0.5484836	8.86E-07
EIF4E2	RPARP-AS1	0.6245937	7.54E-09
NCBP2	RPARP-AS1	0.4977277	1.17E-05
NUDT1	AC010326.3	0.5176554	4.45E-06
NUDT16L1	AC010326.3	0.4613487	5.83E-05
EIF4E2	AC010326.3	0.4124679	0.0003879
NCBP2	AC010326.3	0.558654	5.01E-07
NUDT1	SNHG8	0.7622885	1.78E-14
NUDT5	SNHG8	0.5577021	5.29E-07
CYFIP1	SNHG8	-0.4063	0.0004831
EIF4E2	SNHG8	0.6525511	9.31E-10
NCBP2	SNHG8	0.4173741	0.0003248
NUDT1	AL049840.5	0.8490545	1.62E-20
NUDT5	AL049840.5	0.4342532	0.0001727
EIF4E2	AL049840.5	0.762915	1.64E-14
EIF3D	AL049840.5	0.4631948	5.40E-05
NUDT1	AC010969.2	0.499037	1.10E-05
NUDT5	AC010969.2	0.4575354	6.83E-05
NCBP2	AC010969.2	0.4123576	0.0003894
NUDT1	AC012065.2	0.6504535	1.10E-09
EIF4E2	AC012065.2	0.5688532	2.78E-07
NCBP3	AC036176.3	0.4620729	5.66E-05
DCP2	MIR222HG	0.4171301	0.0003277
NCBP3	MIR222HG	0.4593473	6.34E-05
NUDT1	AC023509.3	0.4605987	6.01E-05
NUDT16L1	AC023509.3	0.5330512	2.03E-06
NUDT5	AC023509.3	0.4557604	7.35E-05
NCBP2	AC023509.3	0.4506248	9.06E-05

NUDT16L1	AC104695.2	0.433015	0.0001811
NCBP2	AC106900.1	0.4278679	0.0002201
NUDT1	YTHDF3-AS1	0.8334727	3.49E-19
NUDT5	YTHDF3-AS1	0.4395546	0.0001406
EIF4E2	YTHDF3-AS1	0.6939652	2.72E-11
NUDT1	AL355353.1	0.7596115	2.48E-14
NUDT5	AL355353.1	0.4395406	0.0001407
EIF4E2	AL355353.1	0.6596235	5.30E-10
NUDT1	AC004130.2	0.6142706	1.55E-08
NCBP2	AC004130.2	0.4565154	7.12E-05
EIF3D	AC004130.2	0.4542616	7.81E-05
NUDT16	IRF2-DT	0.4728363	3.58E-05
NCBP2	IRF2-DT	0.4435508	0.0001202
NUDT16L1	AC008608.2	0.498283	1.14E-05
NUDT1	AL118558.3	0.7972069	1.50E-16
EIF4E2	AL118558.3	0.6250569	7.30E-09
EIF3D	AL118558.3	0.4223704	0.0002703
NUDT1	AC026979.2	0.6328788	4.15E-09
NUDT16L1	AC026979.2	0.4066696	0.0004768
NUDT5	AC026979.2	0.4333713	0.0001786
EIF4E2	AC026979.2	0.5186709	4.23E-06
NCBP2	AC026979.2	0.5076005	7.29E-06
DCP2	AC024060.2	0.4189958	0.0003061
NUDT5	AC024060.2	0.4083945	0.0004486
NUDT7	AC120053.1	0.493418	1.42E-05
NCBP2	AC120053.1	0.440606	0.0001349
SNUPN	AC120053.1	0.4316716	0.0001906
NUDT16L1	AC025181.2	0.5504186	7.96E-07
NUDT16L1	AL691432.2	0.5551811	6.10E-07
EIF4G3	AL691432.2	0.4034237	0.0005343
NUDT16L1	AL390719.2	0.4424095	0.0001257
NCBP2	AL390719.2	0.4291586	0.0002097
NUDT1	AC138696.2	0.8214517	3.02E-18
NUDT5	AC138696.2	0.4486484	9.81E-05
EIF4E2	AC138696.2	0.6600467	5.12E-10
NCBP2	AC138696.2	0.4083391	0.0004495
EIF3D	AC138696.2	0.4460123	0.000109
DCP2	AL645933.2	0.4038171	0.000527
NUDT16	AL645933.2	0.4989029	1.10E-05
NUDT16L1	AC093297.2	0.5163545	4.75E-06
NCBP2	AC093297.2	0.401318	0.0005749
NUDT1	AL139246.5	0.4415641	0.00013
NUDT11	MRPL20-DT	0.5769434	1.72E-07
NCBP1	MRPL20-DT	0.4688952	4.24E-05
NCBP2	MRPL20-DT	0.4504685	9.11E-05
NUDT16L1	AL645608.7	0.5275111	2.70E-06
NUDT5	AL645608.7	0.4171299	0.0003277
NUDT1	LINC01023	0.7351231	4.30E-13
NUDT5	LINC01023	0.453505	8.06E-05
EIF4E2	LINC01023	0.6047436	2.95E-08
NCBP2	LINC01023	0.4195258	0.0003002

NUDT16	AC090425.2	0.4784601	2.80E-05
NUDT16L1	AC090425.2	0.4703098	3.99E-05
NCBP2	AC090425.2	0.5484005	8.90E-07
NUDT16L1	AC012306.2	0.5430653	1.19E-06
NCBP2	AC012306.2	0.4452367	0.0001124
NUDT1	AL021707.6	0.8591254	1.84E-21
NUDT5	AL021707.6	0.4373623	0.0001531
CYFIP1	AL021707.6	-0.408416	0.0004483
EIF4E2	AL021707.6	0.7448079	1.45E-13
NUDT1	AC046143.2	0.8050522	4.50E-17
EIF4E2	AC046143.2	0.6564297	6.85E-10
NCBP2	AC046143.2	0.5031405	9.03E-06
NUDT1	ADIRF-AS1	0.5778537	1.62E-07
EIF4E2	ADIRF-AS1	0.4921542	1.51E-05
NCBP2	ADIRF-AS1	0.5029759	9.10E-06
NUDT1	AC103702.2	0.7943752	2.28E-16
EIF4E2	AC103702.2	0.6902233	3.84E-11
NUDT11	MAP3K4-AS1	0.4257858	0.0002381
SNUPN	CHASERR	0.5516625	7.43E-07
NUDT16	AC009237.1	0.5871786	9.15E-08
NUDT7	AC009237.1	0.4631517	5.40E-05
NCBP2	AC009237.1	0.4829491	2.29E-05
LSM1	AC009237.1	0.46971	4.09E-05
SNUPN	AC009237.1	0.5626442	3.99E-07
NCBP2	AC084036.1	0.5059673	7.89E-06
NUDT16	AC008124.1	0.4541389	7.85E-05
NCBP2	AC008124.1	0.510454	6.35E-06
SNUPN	AC008124.1	0.501762	9.64E-06
NCBP2	AL359921.2	0.4509485	8.94E-05
NUDT1	AC009309.1	0.7488561	9.04E-14
NUDT5	AC009309.1	0.525293	3.03E-06
EIF4E2	AC009309.1	0.5885343	8.40E-08
NCBP2	AC009309.1	0.4414316	0.0001306
NUDT1	AC092171.5	0.4717657	3.75E-05
NUDT16L1	AC092171.5	0.497881	1.16E-05
NCBP2	AC092171.5	0.5260411	2.91E-06
NUDT1	AL512598.1	0.6659417	3.16E-10
EIF4E2	AL512598.1	0.5333043	2.00E-06
NUDT7	LINC02604	0.4811028	2.49E-05
NUDT7	BX537318.1	0.4359648	0.0001617
NCBP2	BX537318.1	0.4933617	1.43E-05
NUDT16L1	AP000894.4	0.6206707	9.95E-09
NCBP2	AP000894.4	0.5228287	3.43E-06
NUDT1	AC108673.3	0.5813843	1.31E-07
EIF4E2	AC108673.3	0.4277175	0.0002214
NUDT1	AC064836.2	0.8599052	1.54E-21
NUDT5	AC064836.2	0.4125057	0.0003874
EIF4E2	AC064836.2	0.7099026	5.95E-12
NCBP2	AC064836.2	0.4062539	0.0004838
EIF3D	AC064836.2	0.4164565	0.0003358
SNUPN	AC009283.1	0.6161109	1.37E-08

NUDT16L1	AL121832.2	0.6484842	1.28E-09
NCBP2	AL121832.2	0.5326532	2.07E-06
NUDT16	AC091271.1	0.4035893	0.0005312
NUDT16L1	AC091271.1	0.5256475	2.97E-06
NCBP2	AC091271.1	0.5980553	4.58E-08
LSM1	AC091271.1	0.4169877	0.0003294
LARP1	AC245041.1	0.4122762	0.0003906
NUDT16L1	AC020910.5	0.6501121	1.13E-09
NCBP2	AC020910.5	0.4733071	3.50E-05
LSM1	AL035661.1	0.4081604	0.0004523
SNUPN	AL035661.1	0.4345112	0.000171
NUDT1	AC015912.3	0.7020155	1.28E-11
EIF4E2	AC015912.3	0.5849238	1.05E-07
LSM1	AC090970.2	0.4057678	0.0004922
NUDT16L1	PCCA-DT	0.412955	0.0003812
NCBP2	PCCA-DT	0.4939555	1.39E-05
LSM1	PCCA-DT	0.6591597	5.50E-10
SNUPN	PCCA-DT	0.4577022	6.78E-05
NUDT16L1	AC068473.5	0.4877522	1.85E-05
NCBP3	AC026356.1	0.4797343	2.64E-05
NUDT1	AC018695.6	0.4157823	0.0003442
NUDT1	AC133552.5	0.8009087	8.54E-17
NUDT5	AC133552.5	0.4253192	0.0002422
EIF4E2	AC133552.5	0.6442962	1.77E-09
NUDT1	AC242842.1	0.4156252	0.0003461
NUDT16	AC091982.3	0.5363058	1.71E-06
NUDT16L1	AC091982.3	0.4650306	4.99E-05
NUDT7	AC091982.3	0.410952	0.0004095
NCBP2	AC091982.3	0.5572074	5.44E-07
NUDT16L1	AL355001.2	0.4138947	0.0003685
NCBP2	AL355001.2	0.4191423	0.0003045
NUDT1	AC244153.1	0.5743171	2.01E-07
EIF4E2	AC244153.1	0.6575605	6.25E-10
NUDT16	AL133243.2	0.4743601	3.35E-05
NUDT16L1	AL133243.2	0.5883372	8.51E-08
NUDT7	AL133243.2	0.4594164	6.32E-05
NCBP2	AL133243.2	0.6615972	4.51E-10
NUDT1	AP001505.1	0.5639357	3.70E-07
NUDT5	AP001505.1	0.4136581	0.0003716
EIF4E2	AP001505.1	0.5025307	9.29E-06
EIF3D	AP001505.1	0.492604	1.48E-05
IFIT5	AC002401.4	0.4239433	0.000255
NUDT16	AC015922.3	0.4757452	3.15E-05
NUDT1	AC023157.2	0.414038	0.0003666
NUDT1	AC010654.1	0.7788609	2.05E-15
EIF4E2	AC010654.1	0.6633147	3.92E-10
NCBP2	AC010654.1	0.4891366	1.73E-05
NUDT1	AC006449.6	0.4343366	0.0001721
NUDT16L1	AC006449.6	0.443556	0.0001202
NCBP2	AC006449.6	0.4805045	2.56E-05
NUDT16L1	AC135050.6	0.4748232	3.28E-05

NCBP2	AC135050.6	0.4410719	0.0001325
NUDT5	AL359513.1	0.4014963	0.0005714
NUDT1	AC099518.6	0.8798426	1.17E-23
EIF4E2	AC099518.6	0.6201774	1.03E-08
NCBP2	AC099518.6	0.4105656	0.0004152
EIF3D	AC099518.6	0.4559651	7.29E-05
NUDT16	AC068831.5	0.4083106	0.0004499
NCBP2	AC068831.5	0.5287581	2.53E-06
NUDT16	AC005332.6	0.5351377	1.82E-06
NUDT1	AL136295.7	0.5518897	7.34E-07
CYFIP1	AL136295.7	-0.408048	0.0004541
NCBP2	AL136295.7	0.4388789	0.0001444
NUDT1	HEIH	0.5632973	3.84E-07
CYFIP1	HEIH	-0.419377	0.0003019
EIF4E2	HEIH	0.4191732	0.0003041
NUDT16	AC026401.3	0.4316931	0.0001904
NCBP2	AC026401.3	0.4532918	8.13E-05
NUDT7	LINC00997	0.4275527	0.0002228
EIF4E	LINC00997	-0.410838	0.0004112
DCP2	EBLN3P	0.4702161	4.00E-05
IFIT5	AC119427.1	-0.430808	0.000197
IFIT5	BISPR	0.4116164	0.0003999
GEMIN5	LINC02635	-0.487571	1.86E-05
NCBP2	AL132857.1	0.4131471	0.0003785
LARP1	AC103718.1	0.475692	3.16E-05
NUDT1	AC092306.1	0.8901854	6.56E-25
NUDT5	AC092306.1	0.4094566	0.000432
EIF4E2	AC092306.1	0.7654638	1.19E-14
EIF3D	AC092306.1	0.4289952	0.000211
EIF4E3	AC083837.1	0.4862637	1.97E-05
NUDT4	AL157829.1	-0.431461	0.0001921
NUDT16L1	NIPBL-DT	0.5387514	1.50E-06
NCBP2	NIPBL-DT	0.6049465	2.91E-08
NCBP2	CERNA2	0.4324905	0.0001847
NUDT7	AC108925.1	0.4348302	0.0001689
SNUPN	LCAL1	0.4288962	0.0002118
CYFIP2	AL162253.2	0.6215212	9.38E-09
NUDT16L1	AC046129.1	0.4361109	0.0001607
NCBP2	AC046129.1	0.4142599	0.0003637
LSM1	AC046129.1	0.4495595	9.46E-05
LSM1	AC010378.1	0.4001109	0.0005995
NUDT16L1	AC113410.4	0.5500529	8.12E-07
NUDT16	PARTICL	0.5269758	2.78E-06
NUDT16L1	PARTICL	0.4271949	0.0002258
NCBP2	PARTICL	0.6114624	1.88E-08
SNUPN	PARTICL	0.4304087	0.0002
NUDT16	AC090004.2	0.4076039	0.0004613
LSM1	AC090004.2	0.4573042	6.90E-05
LSM1	AL590550.1	0.4475032	0.0001027
NUDT16L1	AC097382.3	0.5122703	5.81E-06
NCBP2	AC097382.3	0.4530053	8.22E-05

NUDT7	AC108488.3	0.4080989	0.0004533
DCPS	AL512625.3	0.4247591	0.0002474
NUDT11	AL512625.3	0.4200198	0.0002948
NUDT7	AC234917.3	0.4504873	9.11E-05
NCBP2	AC234917.3	0.4852465	2.07E-05
SNUPN	AC234917.3	0.4767109	3.02E-05
NUDT16	AL132712.2	0.4036954	0.0005293
NUDT7	AL132712.2	0.488956	1.75E-05
NCBP2	AL132712.2	0.4104803	0.0004165
SNUPN	AL132712.2	0.4368126	0.0001564
NUDT1	AC108477.2	0.4829714	2.29E-05
EIF4E2	AC108477.2	0.4666593	4.66E-05
EIF4E3	AL021978.1	0.5943107	5.83E-08
IFIT5	AL021978.1	0.4342465	0.0001727
NUDT1	AC067852.5	0.730125	7.41E-13
NUDT16L1	AC067852.5	0.41311	0.000379
NUDT5	AC067852.5	0.4750275	3.25E-05
EIF4E2	AC067852.5	0.5747628	1.96E-07
NCBP2	AC067852.5	0.4773136	2.94E-05
LARP1	AC083862.2	0.4397431	0.0001396
IFIT5	AC083862.2	0.5259982	2.92E-06
IFIT5	AC090994.1	0.4087286	0.0004433
GEMIN5	AL353807.5	0.4001507	0.0005987
LARP1	AL353807.5	0.4869943	1.91E-05
IFIT5	AL353807.5	0.4123621	0.0003894
NUDT1	AC127526.5	0.4431389	0.0001222
NUDT1	AL354953.1	0.5247254	3.12E-06
EIF4E2	AL354953.1	0.4514149	8.77E-05
NCBP2	AC245407.2	0.4860992	1.99E-05
LSM1	AC245407.2	0.5324326	2.09E-06
SNUPN	AC245407.2	0.4616175	5.76E-05
SNUPN	AC016394.2	0.4397998	0.0001393
