# Supplementary material for: The Risk Correlation between N7-Methylguanosine Modification-Related lncRNAs and Survival Prognosis of Oral Squamous Cell Carcinoma Based on Comprehensive Bioinformatics Analysis
Source: Appl Bionics Biomech. 2022 Aug 24;2022:1666792. doi: 10.1155/2022/1666792 (PMC9433249; doi:10.1155/2022/1666792)
Supplement: Supplementary Materials — File m7G-lncRNAs_exp.xls shows the expression matrix of 399 m7G-related lncRNAs. Rows represent m7G-related lncRNA names, and columns represent samples. File co-exp_rel.xls shows the coexpression relationship of m7G-related lncRNAs and m7G-realated mRNAs. The first column represents m7G-realated mRNAs, the second column represents m7G-realated lncRNAs, the third column represents coexpression correlation coefficients, and the fourth column represents the P value of the correlation test. File risk.xls presents univariate Cox regression analysis for 16 significant m7G-related prognostic lncRNAs. The first column represents samples, the second column represents the survival time of patients, the third column represents their survival status, and columns 4 to 19 represent m7G-related prognostic lncRNAs. File risk.xls presents the risk scores of nine m7G-related prognostic lncRNAs that constitute the prognostic model. The first column represents samples, the second column represents the survival time of patients, the third column represents their survival status, columns 4 to 12 represent m7G-related prognostic lncRNAs, and columns 13 and 14 represent the risk score and risk grouping for each patient. File coexp_network.xls shows the coexpression relationship between the m7G-related prognostic lncRNAs and mRNAs. The first column represents prognostic m7G-realated mRNAs, the second column represents prognostic m7G-realated lncRNAs, and the third column represents the correlation type. [file 1666792.f1.zip › m7G-lncRNAs_exp.pdf]

ID	TCGA-CV-74	TCGA-CX-70	TCGA-CN-47	TCGA-CR-73	TCGA-CN-60	TCGA-CQ-62	TCGA-CV-74
LINC01116	15.9056	0.6523	1.8911	4.58	3.4327	4.5624	1.4745
TMEM99	4.8177	8.0807	6.6401	2.2233	3.9239	5.2463	1.8687
SNHG11	1.1582	3.3175	1.2055	0.7908	1.1042	1.0625	0.4991
DLGAP1-AS1	2.1275	2.629	3.3897	0.4407	1.6083	4.5642	0.316
NINJ2-AS1	0.6587	1.7139	1.6986	0.9608	1.5173	1.0973	0.2865
ZFAS1	1.6842	2.8273	2.4857	0.5347	0.9297	1.1783	0.5736
EIF3J-DT	0.7564	1.4616	1.0167	0.7097	1.0767	0.9957	0.2797
FLJ37453	0.7504	3.3057	1.3815	0.4034	0.9068	1.0795	0.3845
LINC02870	0.16	0.5375	3.4512	0.7654	0.6268	0.5412	0.284
TP53TG1	0.5356	0.7168	0.4437	0.3251	0.2412	0.5547	0.3113
C5orf38	1.9548	1.826	3.4809	1.0026	1.9722	0.5445	0.5943
KTN1-AS1	0.5528	0.1888	0.4397	1.2029	0.6377	0.4565	0.0861
AC091057.1	0.503	1.619	0.3482	0.9118	0.4502	0.2281	0.1423
PAX8-AS1	0.2052	0.1401	0.0367	0.054	0.1958	3.812	0.0484
LINC01560	2.2215	2.355	0.9942	0.8827	3.4067	0.7139	0.3201
CH17-340M2	1.5803	4.0715	1.4519	0.6528	3.1579	1.7295	0.7702
HHLA3	3.22	2.6258	3.7689	3.5848	4.4912	8.8182	1.5684
SNHG12	1.4178	2.1998	0.3894	0.2288	0.3228	0.498	0.404
NBR2	1.2233	2.7138	0.7184	1.4159	1.9361	0.9446	0.4972
PDCD4-AS1	1.9656	2.114	1.7953	1.2265	3.2111	0.8982	0.7066
AC083799.1	9.3277	10.6315	5.4137	4.2419	9.6843	8.8927	6.8081
SNHG5	8.0528	6.4311	4.2681	3.5891	3.1714	6.7753	3.0848
ARRDC1-AS1	6.2575	3.0702	1.7997	0.8687	2.0476	1.1512	0.6789
LINC00963	2.6936	4.6885	1.256	1.0021	1.0293	1.2212	0.4151
PSMB8-AS1	1.1275	1.3509	1.6359	0.9398	2.4518	1.096	0.3924
SNHG32	1.4979	7.422	4.2132	0.9633	1.6165	1.3658	1.0312
MIR1915HG	0.6061	0.4881	0.4174	0.3996	0.7042	0.7836	0.4928
HCP5	1.3658	5.6956	9.2943	7.1424	16.6826	4.0496	3.0458
LINC02693	1.5298	0.7201	0.304	1.613	1.895	0.6356	0.1936
AC016747.1	7.1405	1.141	0.4371	2.6994	4.4354	2.1264	0.5496
APTR	0.4299	3.5405	0.2872	0.2933	0.3591	0.5599	0.1457
AC087491.1	0	3.6938	0.079	0	0	0.0146	0.0466
AC010168.1	0.68	2.7488	2.7006	0.066	0.0599	0.4893	0.3844
CD27-AS1	2.0366	2.6001	2.2096	0.5775	1.1571	1.2189	0.287
DHRS4-AS1	1.5138	4.0764	3.4979	1.4124	1.6492	2.3137	0.5489
EXOC3-AS1	2.6864	2.6179	5.0395	1.943	3.1464	2.3532	0.7594
AL441992.1	8.9703	4.0311	8.2233	5.4215	3.5799	6.2946	1.0924
HLA-DQB1-A	0.0623	0.1168	1.0942	0.0393	1.5324	0.2279	0.1615
LINC00205	3.9444	0.7174	0.7383	1.3577	1.7066	0.9429	0.1969
EPB41L4A-A	1.9106	2.1813	1.7942	0.8668	1.4062	1.3035	1.0811
HAGLR	2.2485	3.713	7.1446	0.6607	0.8376	0.9554	0.2118
LINC01133	0.0187	39.2626	8.6058	0.0826	0.2462	3.3725	3.0838
AC096677.1	0.272	1.2657	0.7719	0.5579	0.2595	0.59	0.2058
LINC00885	0.0146	0.4321	0.1445	0.2214	0.3096	0.1248	0.2022
MRPL20-AS1	5.5893	5.7619	4.7282	2.3342	4.7485	5.1769	2.1401
AC073957.1	0	1.7782	2.1169	4.0535	0.0368	0.2612	0.6387
AL590617.2	1.745	2.5234	0.9012	0.8711	0.8647	0.8156	0.456
LINC01770	0.101	0.3032	4.2586	0.3313	0.1849	0.2956	0.0873
LINC01980	1.3552	2.2884	0.9752	0.5305	0.2406	0.0029	1.4694
FGD5-AS1	16.4303	16.6273	10.5017	12.3596	14.6981	12.3547	7.7998

P3H2-AS1	0.0204	0.4022	1.8606	0	0.1168	0.6308	0.0529
LINC00623	1.1631	4.0125	0.5372	0.6256	0.7188	1.3063	0.24
BAIAP2-DT	3.4488	2.3682	3.2111	1.8765	2.9344	3.0864	1.6809
NUP50-DT	0.5744	7.7773	2.6141	2.3958	1.7786	4.3652	1.665
AL354836.1	1.5391	3.6227	1.8848	2.1654	0.751	1.0672	0.4363
HAGLROS	2.3758	1.3809	2.9466	1.1414	0.3159	0.8728	0.3976
Z93930.2	0.2338	2.8081	0.4494	0.118	0.1071	0.6941	0.0505
AC010894.1	1.031	1.4512	0.5663	0.8674	0.8114	0.5241	0.13
DANCR	4.448	7.7224	1.7041	0.4495	0.8252	2.1434	1.3705
AC099066.2	0.4844	0.1546	0.3885	0.428	0.965	0.7644	0.8629
SOX21-AS1	0.0779	2.735	1.7	0.6749	0.0689	0.0519	0.5665
AL390728.4	2.0444	3.1353	1.5467	1.255	1.1293	1.2877	0.5227
MELTF-AS1	0.3771	4.6717	0.5303	0.2285	0.3281	0.135	0.1565
AL355607.1	0.8154	0.0547	0	0.8821	0.1334	0.758	0.6044
HCG11	0.368	3.2951	2.3892	3.0382	1.9443	0.8245	0.4017
PCAT6	1.699	7.4462	7.9955	2.1442	3.8458	7.3651	6.1598
LINC00392	0	0.0296	0.0693	0.0199	0	0.0513	0
AC106875.1	0	0.0138	12.863	0	0	0.0119	0.0127
AL513550.1	0.7901	3.2211	1.0503	0.5698	1.0641	0.9235	0.3444
AC093159.1	0.0811	0.3803	0.5787	0	0.0464	0.0659	0.1402
AC064807.1	0.4463	0.804	0.5334	1.9014	1.455	1.3155	0.2377
LINC02041	0.9779	7.2756	3.2993	1.2342	1.1995	0.0568	0.1208
ZFAND2A-DT	0.3347	1.8139	0.6693	0.992	0.6587	0.5231	0.2244
ANKRD10-IT	1.482	1.7132	0.873	0.7319	1.3524	1.4151	0.26
AC046143.1	0.5127	1.7584	2.3691	0.6024	0.425	0.7477	0.1681
MYOSLID	24.0568	0.3756	2.6795	0.7236	1.0588	2.7539	0.3601
MAST4-AS1	0.2875	0.8093	0.8017	0.0837	0.5063	0.6296	0.4207
TRPM2-AS	1.2595	0.8372	1.3424	0.2176	0.0515	0.0183	0.726
AL162231.2	0.6946	0.4574	1.9009	0.4922	0.572	2.944	0.2529
PRRT3-AS1	3.0783	1.7595	1.8138	0.4945	0.8971	0.5918	0.6775
NFE4	0.0962	0.0602	0.0528	0.0202	0.0367	0.013	0.0139
SERPINB9P1	0.544	0.3355	0.3799	0.4708	0.6406	0.4393	0.1613
AP000695.1	4.1946	0.3792	1.2259	0.3886	2.4231	1.5964	0.0998
AC021078.1	0.4357	0.6292	0.365	0.6604	0.5239	0.5001	1.1526
AL604028.1	1.0344	2.6425	1.1994	1.777	0.8224	1.379	0.6211
LINC02541	0.3994	4.846	4.6229	1.9625	4.1563	1.0559	0.7832
AC005392.2	0.685	2.732	4.1388	0.4323	0.3268	4.2255	12.6852
AC074389.2	0	0.0369	14.1241	0	0	0	0.544
COA6-AS1	1.1355	11.0487	1.0833	0.0754	0.4105	0.5834	0.3876
TMEM44-AS1	2.0693	7.93	2.535	0.7384	2.6667	1.2602	0.5246
PIK3CD-AS2	2.6899	2.5586	1.1458	0.5774	0.5028	0.8335	0.3006
DCST1-AS1	0.8988	1.0593	1.217	0.2902	0.359	0.4931	0.8314
AC012313.1	1.2471	2.6662	0.7071	1.777	1.6349	1.3089	0.6218
MHENCR	1.4372	4.2274	1.5317	0.6768	2.5786	1.6925	0.9274
EMSLR	0.3674	0.231	0.2179	0.3988	0.4122	0.4961	0.2859
AC093673.1	23.8913	4.7527	7.5443	3.8486	6.2231	13.4157	3.164
AL354892.2	0.5543	2.0806	1.5925	1.2915	1.562	0.3121	0.553
LINC00665	3.371	4.1282	1.646	0.0642	0.0922	0.1355	0.0379
SNHG15	2.5432	10.0537	3.1243	4.4319	3.2061	3.0373	2.8288
SNHG7	3.4534	9.4452	2.7323	0.9577	1.8099	1.3602	1.5158
AC016876.1	1.905	2.3018	1.6413	1.9486	2.0384	1.9446	1.0443

AP000695.2	2.1296	0.3559	0.6409	0.2761	1.4528	1.4	0.1009
LINC01503	11.1102	2.7369	8.9239	2.0682	1.1804	3.274	1.0985
AC074117.1	1.4806	2.1266	0.4204	1.3919	0.7898	0.7414	0.283
AL035446.1	4.2862	5.5172	5.0001	1.2925	0.6948	0.9564	0
MAPKAPK5-A	3.9905	6.1847	5.1713	1.2781	2.5255	2.4623	0.975
AL139289.1	0.13	0.8538	1.4278	0.3609	1.5773	0.7189	0.2023
GAS5	13.1478	27.0005	29.7476	4.9042	4.412	12.1378	5.7225
AC128709.2	0	0.549	2.0296	0.0389	0.1763	0.0501	0.0799
MCF2L-AS1	0	2.0608	0.2121	0.0305	0.0553	0.0785	0
LINC01278	1.5706	1.3883	0.9367	0.8324	2.4794	1.238	0.5031
AC073046.1	0.7049	3.8864	1.1132	5.8386	3.6316	0.8959	1.9047
LINC01871	1.3435	1.6589	0.6991	0.1785	0.8095	0.4026	0.2446
ASH1L-AS1	1.3928	1.7605	1.0148	0.5022	1.0088	0.4393	0.2458
AL354766.2	0.1219	0.0762	0.357	0	0.093	0	0.0351
SLC12A9-AS	1.3504	1.3983	0.3069	0.8815	1.546	1.3257	0.3221
LINC02195	1.4027	1.2614	0.1284	0.5163	1.0036	0.2852	0.2021
GS1-124K5.	0.3948	2.1082	0.688	0.1375	0.8885	0.7642	0.2472
FOXD2-AS1	3.5269	2.018	0.9329	0.605	1.2152	0.7576	0.2665
BX293535.1	0	0.4558	1.1339	0	0.278	0.3457	0.1575
LINC01637	0.3517	0.6836	0.8279	0.2536	0.6039	0.4495	0.0869
LINC00857	0.541	0.0707	2.8061	0.1469	0.4312	0.6685	0.0474
TTLL11-IT1	0.1307	0.046	1.9916	0.7421	3.4406	1.1293	0.2825
AC022034.1	0.2945	0.0835	0.3837	0.5071	0.7161	0.8486	0.1362
DGUOK-AS1	0.3625	0.9933	1.1946	0.6771	0.996	1.1795	1.2538
AL355574.1	3.4406	2.6906	1.9348	0.3619	1.7585	0.1999	0.3719
LINC01589	0.3449	0.4119	0.6889	0.3562	0.0718	0.153	0.0542
LINC00707	0.9873	0.0115	0.1344	0.3088	0.114	1.8521	0.2493
BX470102.1	0.0971	1.5487	0.6043	0.0408	0.2964	0.2106	0.0839
LINC02031	0.1451	0.2179	7.5565	5.4581	0.3655	1.2985	0.2008
KMT2E-AS1	1.3541	4.1845	1.1797	0.825	1.3631	1.5951	0.7267
PRR34-AS1	2.2009	2.0211	0.9844	0.4762	0.5579	1.2659	2.0389
SNHG3	3.9426	8.8905	1.1682	0.87	1.9681	3.9215	1.5664
LASTR	0.8224	0.082	1.8068	0.094	0.2706	2.7653	0.3667
AL591895.1	1.8763	3.6141	2.5493	0.7478	0.2261	2.4499	1.2381
AC082651.3	0.6972	0.4306	1.2797	0.969	0.2223	0.1292	0.3205
LINC01214	0.0982	0	2.1572	0.4027	0.1124	1.3577	1.422
ATP1B3-AS1	0.1828	0.7719	0.6024	0.5768	0.994	0.1487	0.1185
AC147067.1	1.6916	1.0026	1.5322	0.2622	1.0532	0.7966	0.2309
AC108676.1	0.0289	0.277	3.6619	3.2211	4.0416	1.0498	0.2502
NEAT1	2.0694	4.1495	4.923	1.5717	4.5404	3.6631	3.0051
SCAMP1-AS1	0.7928	0.8979	0.7996	0.4572	1.7488	0.3975	0.133
RAD51-AS1	0.4631	1.4924	0.4953	0.2922	0.5301	0.3668	0.0685
SNHG6	24.582	36.4124	24.0521	7.304	12.1308	25.4424	8.4233
AC016065.1	1.7569	1.1156	1.5118	0.4952	1.951	0.4764	0.3899
SBF2-AS1	0.3452	0.3831	0.5068	0.3602	0.8777	0.4482	0.221
AL049840.2	0.3549	1.4011	0.3227	0.4479	0.6445	0.7665	0.2645
MIR210HG	0.7107	5.134	15.907	2.6162	4.3394	1.096	5.9722
ZBED5-AS1	2.2006	0.9361	0.9973	1.4665	2.1771	2.6743	0.4975
GPRC5D-AS1	1.1907	2.4234	1.0486	0.038	1.8233	0.1471	0.8297
NNT-AS1	1.0058	1.0293	1.9505	0.6983	1.2275	1.2402	0.2552
AC114956.1	0.537	1.1337	0.9954	0.8471	0.73	0.5733	0.4643

TRIM52-AS1	2.522	3.781	2.3313	1.4492	4.2605	1.8096	0.9042
USP46-DT	3.1316	2.831	0.9726	0.7863	1.0699	1.2536	0.6096
AC093895.1	1.5912	5.0891	1.055	0.808	0.4419	0.0077	0.2931
IRX4-AS1	0	0.7209	1.1547	0.5613	0.648	0.6577	0.839
CASC9	3.0902	12.0291	2.6403	0.7222	0.7007	0.0121	3.0936
AC034231.1	1.6959	1.4113	1.2302	0.1212	2.1979	0.8589	0.332
AL589765.4	0.1349	0.2111	0.7412	1.7599	0.309	0.5488	0.1167
LINC00942	26.3337	0.1313	0.1964	0.0589	0.2581	0.2909	0.0269
LINC02762	0.4897	0.3406	2.2973	0.8107	2.6778	1.5628	0.4658
SNHG18	0.9336	0.7603	10.2745	2.0675	2.5207	6.9775	1.5154
AC125807.2	4.4399	1.193	0.4995	3.7892	1.2651	1.229	0.4566
THAP9-AS1	3.5835	9.2375	8.0575	1.81	2.1071	5.1208	1.7503
AC020661.1	0.231	1.2628	0.0221	0.8745	0.138	0.2206	1.2243
LINC00958	14.568	2.9398	12.2248	9.9748	5.5502	5.7456	4.6888
FAM160A1-I	0.096	1.126	1.6872	1.6355	1.5935	0.6637	0.2905
LINC01932	0.09	1.4914	0.56	0.3028	0.412	0.1951	0.4408
AC124067.2	2.4547	1.8037	2.0351	0.2046	0.7953	0.8665	0.0601
GASAL1	1.1754	0.4258	3.9456	2.132	0.8199	0.7871	0.9785
MINCR	0.8198	2.2026	2.4304	0.2977	0.5271	0.9318	0.2719
OTUD6B-AS1	1.2074	2.1791	2.142	1.1644	0.9822	2.021	0.6194
AC007991.2	0	0.3162	0.5552	0	0.7714	0	0
TNFRSF10A-	2.6705	1.291	1.126	0.8171	1.0191	0.8996	0.9563
AP001207.3	0	0.3612	1.038	0.53	0	0.9678	0.2421
AC009902.2	0.2451	0.6211	3.4466	0.7115	0.8418	0.1794	0.6358
CASC19	0.0023	2.1909	3.0209	0.0039	1.6777	0.0031	0.0769
AC091563.1	1.0054	0.922	5.7479	0.5191	0.4447	1.7656	0.1778
AL354920.1	1.3789	3.1282	1.2817	1.1092	1.1102	0.8627	0.5372
FLJ20021	7.5908	6.7151	12.5676	1.4549	5.2789	7.0944	2.8586
AP003068.2	0.0768	1.2964	1.1804	0.9808	0.1867	1.2642	0.2572
WAC-AS1	4.2056	9.0262	5.6565	2.369	3.839	5.1746	4.1408
AP001372.2	0.9029	1.0672	1.7338	0.7481	1.223	2.6518	0.3397
AP003390.1	2.1684	1.819	0.6496	1.5342	2.2567	0.7215	1.0227
AC136475.3	0.4795	0.1312	1.0096	0.2774	0.2974	0.26	0.1037
AP003119.2	1.6503	1.412	1.1197	0.3063	1.7782	0.8291	0.4617
AP002360.1	2.2292	2.2599	3.0565	0.2875	2.4589	1.7206	0.4502
TOLLIP-AS1	0.2013	1.099	0.6634	0.5543	0.9008	0.4465	0.1899
AC090559.1	0.809	0.5391	0.219	0.9766	1.1611	0.453	0.1926
SNHG9	1.7775	9.423	1.1305	1.1807	0.8568	3.5766	2.2649
AC109322.1	0.4486	2.0666	0.3435	0.2574	0.2957	0.3538	0.2351
AP006621.3	1.5002	1.4403	0.8367	0.3641	1.1758	0.5256	0.3592
AP001830.1	0.0745	0.4893	0.9547	0.094	0.8244	0.3837	0.3435
AC104031.1	0.5543	0.0433	4.9218	1.1659	0.899	0.7514	0.6789
SNHG1	7.1717	11.9613	9.7943	2.1686	2.0712	3.1438	2.6196
LINC02446	0.3515	0.5741	0.6149	0.1314	0.7004	0.1906	0.3264
URB1-AS1	4.8798	5.5494	5.2015	2.5574	5.7759	3.9356	1.0191
ZBTB11-AS1	1.3468	1.7576	0.8051	0.7273	0.9897	0.7032	0.5037
MIR200CHG	0	8.9836	5.9323	0.6816	2.8102	6.2295	3.5232
TMPO-AS1	0.6808	1.6763	0.551	0.9238	1.5118	0.3747	0.2257
AL928654.2	0.5406	1.5581	0.2969	0.4873	0.5305	0.4711	0.1002
PSMA3-AS1	2.2426	1.7258	1.2509	1.4898	1.4019	1.5921	0.5555
G2E3-AS1	2.4091	2.5022	0.5542	0.2079	0.3536	0	0

LBX2-AS1	1.8634	1.5474	5.7514	0.9157	2.9356	1.6565	0.1996
CPNE8-AS1	2.4827	2.6363	1.6866	0.3298	0.5236	0.372	0.3389
AC025575.2	2.4634	8.7043	0.0334	2.3029	0	0	0.4733
AC009779.2	2.5839	1.989	2.3676	0.8266	2.3038	1.5594	0.3398
AL512791.1	0.2291	0.7525	0.1888	0.506	0.459	0.6988	0.9409
LINC00640	0.0409	0.4545	0.4871	1.0073	0.0312	0.6325	0.1946
RHOXF1-AS1	0.0069	0.0775	1.6775	0.0087	0.0551	0.0448	0.0059
ARHGAP5-AS	1.7666	2.1464	0.9703	1.9128	1.0964	2.1403	1.3506
LINC02310	1.4419	0.0846	0.429	0.2654	0.2407	1.4661	0.3377
LINC02820	1.225	5.002	2.1094	0	0.3411	0	0
FOXN3-AS1	1.5316	4.8358	3.8557	3.3022	2.5023	2.4008	3.0349
AL049870.3	0.0955	0.1792	0.0524	0.1205	0	0.1553	0
LINC00519	0.6596	12.6972	2.1032	3.1473	1.363	3.1931	0.5147
AL121820.2	0.1848	0.5201	0	0.0777	0.2115	1.5529	0.0533
AC023906.2	3.0125	0.2094	2.4922	1.0796	1.0645	0.6655	1.1898
AC015660.1	0.098	0.0793	0.5435	0.7881	0.1364	0.3214	0.3313
AC108449.2	1.5875	3.9143	1.6071	1.2964	1.2116	0.709	0.8344
AC087612.1	3.4854	0.9085	3.431	2.6457	0.7325	0.945	0.9681
AC023906.5	5.8719	0.4317	1.3673	0.4098	1.1772	0.8364	0.1638
AC012640.2	1.6401	2.3981	1.5921	1.3238	1.8339	0.7911	0.3957
AC009113.1	0.8018	1.0098	0.1854	0.6214	1.6265	0.635	0.2372
AL050341.2	1.6948	4.8028	1.5311	0.8444	1.6722	1.4784	0.3374
AL133338.1	1.0421	1.4881	1.4682	0.7434	1.2448	0.7647	0.1273
AC040169.1	0.4947	1.0832	0.9782	0.7909	1.1327	0.7243	0.5418
AC104794.3	0.634	1.9489	0.6844	0.7863	1.0011	0.6935	0.586
LINC02846	0.6183	0.7978	0.9056	0.3902	0.1032	0.0629	0.3341
CD2BP2-DT	2.5116	3.1696	3.1966	0.7433	2.8555	1.1976	0.5092
AL035071.1	1.1127	2.2397	0.9212	0.7836	0.2585	3.0695	2.7399
SNHG19	11.6058	100.8009	11.9213	4.5021	5.8668	14.5486	14.075
LINC02562	1.863	11.4104	4.1113	1.1966	0.6855	1.1636	1.1218
LINC01882	1.5793	0.4734	2.2731	0.5445	1.2556	0.5828	0.1012
AC132872.1	0.8915	2.6447	0.6002	0.6896	1.2511	1.5555	0.7708
AC011374.1	0.1206	2.2844	19.3692	0.775	0.9541	0.8563	0.7016
AL118516.1	3.8212	4.3319	5.5499	0.6797	1.5561	2.9205	0.7318
DOCK9-DT	0.4471	2.3377	2.0115	1.6392	1.0482	1.5587	0.81
AC092718.4	6.1915	9.4584	4.587	5.9667	9.451	7.9643	2.3447
AL512274.1	0.2119	2.6808	1.0616	6.2908	0.2232	2.0272	11.6173
LINC02188	0.0088	0.0884	0.2327	0.2674	0.0168	0.1244	0.4808
SPINT1-AS1	0.1109	4.7761	3.3363	1.1202	1.3019	0.8686	1.2591
Z95115.1	1.3191	2.7218	0.5388	0.2028	0.9584	0.736	0.1316
AL031058.1	3.8893	2.4332	4.9314	3.4434	2.5918	5.2412	3.5804
VPS9D1-AS1	8.5276	2.2627	3.6825	3.303	6.2503	2.2723	1.2713
LINC01003	0.6916	0.521	0.535	0.8851	0.4349	0.7289	0.2864
AC026471.4	1.1863	3.8066	0.5885	1.1592	1.5334	0.7782	1.1581
AL596244.1	0.3046	0.7083	0.4246	5.0442	2.0613	2.2672	0.3342
LINC02582	2.3985	0.194	0.4089	0	0	0.0336	0
LOXL1-AS1	0.9483	4.516	1.3949	0.3489	0.9201	0.8315	0.2363
MIR193BHG	1.2896	0.2212	0.7645	0.4517	1.8926	1.5365	1.0144
AC007114.1	0.4351	1.3507	0.2206	0.2816	1.2646	0.3721	0.2508
LINC00667	1.2171	4.9344	2.3393	0.2277	1.4382	3.0545	0.7536
AC080037.2	0.0193	0.3809	1.9746	0.2927	0.4204	0.3616	0.0501

AC015922.2	8.5666	0.6041	1.2511	5.5621	1.4172	2.2556	0.8991
MAFG-DT	1.9132	6.203	1.0857	0.5493	1.4429	1.8807	0.6115
AC004585.1	0.8643	0.1211	2.3807	0.4559	1.1224	1.6579	0.0892
SNHG25	0.6181	1.392	0.4074	0.312	0.2123	0.3017	0.5344
AC093484.4	1.413	2.1747	0.1863	0.4994	0.453	0.2759	0.4398
ILF3-DT	7.6165	17.9295	5.873	2.7012	6.6463	6.9706	2.4799
LINC01842	1.4766	0.063	0.3195	2.146	1.6905	0.4186	0.0387
AC011472.2	0.3763	1.0357	0.248	0.5382	0.2297	0.3877	0.7808
AP005482.2	1.2887	2.2619	1.2846	0.8433	0.4645	0.9706	0.5159
AC022031.2	0.4597	3.6028	0.6259	0.4412	0.5114	1.1954	0.3975
CEBPA-DT	0.3518	1.0197	0.3177	0.3552	0.3848	0.3624	0.169
AC027307.2	1.3957	3.8585	2.1341	4.9038	1.5547	3.0377	1.5004
SNHG30	2.1775	11.7342	2.3209	0.6394	3.9244	2.9198	0.755
AC036176.1	0.394	0.6162	1.6351	1.0498	1.1276	0.8546	0.3407
AP001542.3	2.3149	4.3753	1.0029	3.2509	1.4931	14.5865	10.6282
CIRBP-AS1	0.5575	3.4722	0.181	0.7996	0.7979	0.6287	0.6793
AC020916.1	0.2715	1.8648	0.5608	0.255	0.7332	0.7645	0.1482
AC008735.2	0.3982	1.5521	0.1682	0.2319	0.2104	0.299	0.1059
AC024075.1	0.5035	1.7128	0.9334	1.1121	0.5764	0.6144	0.4626
CARD8-AS1	2.2427	0.868	2.0227	1.2666	1.1089	1.2777	0.3094
AC008764.2	0.5484	2.3586	1.4056	1.6149	0.2616	1.1523	1.0669
AC005261.1	3.1839	8.9483	2.478	2.2256	1.5338	4.9171	4.7209
LINC02560	0.1123	4.6722	1.3982	0.9449	0.3857	14.616	22.593
IGFL2-AS1	0.1236	0.6138	1.4711	0.182	0.5189	3.7664	1.5721
AL121761.1	0.1043	0.8806	7.502	0.0658	0.0955	0.1442	0.009
MAN1B1-DT	0.7435	3.8071	1.2503	1.2511	0.9352	0.9258	0.2381
AC024075.2	0.8515	3.1088	1.102	1.0895	0.3739	1.5466	0.9279
RPARP-AS1	0.3487	1.8088	0.8396	0.3908	0.5109	0.4356	0.1833
AC010326.3	2.6065	4.0845	1.0286	1.1817	1.1299	1.5644	0.8315
SNHG8	2.9175	8.8015	3.6991	0.6071	1.3455	2.2864	2.0212
AL049840.5	9.5765	11.5316	3.0489	9.3055	4.4145	11.3742	6.6316
AC010969.2	1.0742	3.4984	0.5141	1.0135	1.0776	1.1117	0.7587
AC012065.2	0.1566	0.1469	0.172	0.1482	0.3585	0.8914	0.0677
AC036176.3	0.1233	1.3501	0.9483	0.5188	1.2236	0.2341	0.9241
MIR222HG	0.6355	1.5516	0.5558	0.1791	0.4874	0.3563	0.192
AC023509.3	1.6407	2.3094	0.4871	0.1399	0.7615	0.9558	0.2876
AC104695.2	0.3326	0.3121	0.5845	2.6863	0.3807	0.8115	0.7285
AC106900.1	1.2467	1.4242	0.4168	1.3682	0.3723	0.3086	0.703
YTHDF3-AS1	0.3829	2.4479	1.4196	0.3926	1.1507	0.5256	0.3932
AL355353.1	0.0521	3.2249	0.3432	0.1971	1.1326	0.5082	0.1351
AC004130.2	0.652	1.0438	1.5378	1.2828	0.7465	1.1231	0.1492
IRF2-DT	1.2425	2.6219	1.1312	0.4938	1.4932	0.7929	0.3027
AC008608.2	0.9087	1.0853	0.2722	0.4692	1.5132	0.7055	0.4643
AL118558.3	0.9714	2.391	1.207	0.6632	1.9507	1.4637	0.3029
AC026979.2	0.938	9.0825	0.7026	0.1076	3.124	0.763	0.8111
AC024060.2	0.8696	1.6973	0.2675	0.1317	0.1991	0.5376	0.3007
AC120053.1	0.6537	1.183	0.4873	0.7955	0.294	0.9495	0.2826
AC025181.2	0.9213	1.9703	5.6014	1.0275	1.3245	1.307	0.4261
AL691432.2	1.1551	3.0856	1.4263	1.2131	0.6467	1.2001	1.1154
AL390719.2	0	5.0317	0.5737	0.1758	0.0598	0.2266	0.2107
AC138696.2	1.3869	1.2292	1.4389	0.5835	0.9703	0.6267	0.1999

AL645933.2	0.9538	1.3493	0.2346	0.3414	1.4342	0.7874	0.2831
AC093297.2	0.6144	1.5567	3.3821	1.3613	0.5862	1.4271	0.7496
AL139246.5	0.202	0.3686	0.3822	0.3116	0.2827	1.1685	0.3688
MRPL20-DT	5.3139	1.6093	0.4462	2.3779	0.2971	0.4956	0.4975
AL645608.7	0.2471	2.0407	0.371	0.1248	0.2358	0.0737	0.0712
LINC01023	1.3399	4.4379	1.9481	0.4476	3.1582	0.9937	0.6815
AC090425.2	0.1227	3.4552	0.1348	0	0	0.2995	0.3184
AC012306.2	3.0001	2.8922	1.3782	1.4113	1.5612	1.5529	0.6603
AL021707.6	0.9099	2.1674	0.4998	0.2208	0.601	0.5408	0.2723
AC046143.2	2.6848	3.7288	0.4719	0.6777	0.7377	0.6115	0.1857
ADIRF-AS1	0.293	3.111	1.5007	0.4754	0.9343	0.8875	0.5195
AC103702.2	0.0538	0.3132	5.3693	0.1631	8.4921	0	0
MAP3K4-AS1	5.2609	1.7836	0.4008	0.846	1.9525	1.7876	0.5943
CHASERR	1.9875	6.1792	2.4799	1.2153	0.9377	1.5307	1.3225
AC009237.1	0.3557	0.6474	0.5447	0.6258	1.2341	0.6313	0.2796
AC084036.1	0.575	3.428	0.6688	0.2561	0.2711	1.6233	0.351
AC008124.1	0.6075	1.9223	0.6111	0.5037	0.469	0.5573	0.2886
AL359921.2	1.329	4.573	0.4171	0.5192	0.2536	0.4633	0.1642
AC009309.1	1.4941	1.332	0.6565	0.8486	1.1546	0.2127	0.3876
AC092171.5	0.2554	2.6874	0.521	1.1511	0.6682	0.3413	0.2366
AL512598.1	0.5665	0.5316	0.1383	0.1589	0.4323	1.4335	0.0544
LINC02604	0.6741	1.4808	0.2743	0.3844	0.8289	0.4833	0.3972
BX537318.1	1.025	1.0286	1.6264	0.6827	1.369	0.3937	0.357
AP000894.4	0.4625	2.3703	0.7425	0.1347	1.3033	0.4919	0.0615
AC108673.3	0.359	2.0697	1.0142	0.2589	0.1174	0.8344	0.7983
AC064836.2	0.9702	3.5459	1.4865	0.3545	0.3507	1.1837	0.1987
AC009283.1	1.5451	4.5748	0.439	0.6724	1.2199	0.7367	0.7371
AL121832.2	2.135	3.7897	1.0172	0.422	2.5914	1.2972	0.5783
AC091271.1	0.6211	1.7207	1.0071	0.2986	1.1681	0.7938	0.1023
AC245041.1	19.4709	5.2707	7.1982	13.7835	61.7761	9.8817	8.0041
AC020910.5	1.4955	2.3589	3.2857	0.241	0.6193	0.8541	0.3302
AL035661.1	0.03	7.7239	2.3924	3.1657	0.6534	6.3652	1.2338
AC015912.3	0.5925	3.9384	1.5187	0.1246	0.3392	0.5623	0.2135
AC090970.2	1.627	0.54	1.8091	1.5776	1.272	0.3228	0.223
PCCA-DT	5.427	3.7652	3.7623	2.1926	1.2881	1.7765	1.0157
AC068473.5	1.2273	1.0117	1.6671	0.7431	0.7215	0.6476	0.3729
AC026356.1	1.6967	1.818	1.0293	0.6073	0.8988	0.9888	0.5036
AC018695.6	0.5148	0.4227	0.1767	0.2437	0.442	1.518	0.1391
AC133552.5	0.547	1.8117	1.4139	0.2843	0.6999	0.5496	0.4452
AC242842.1	0.8072	2.5251	2.5336	0.5822	2.2884	1.4696	0.2327
AC091982.3	0.568	2.332	2.5479	1.374	2.3844	0.7701	0.4707
AL355001.2	0.9089	5.7133	2.9319	3.5324	1.3213	1.15	1.0977
AC244153.1	3.029	0.7494	6.6245	2.7976	1.5447	4.3901	4.0238
AL133243.2	1.3992	1.7408	1.0829	0.1605	0.4126	0.2673	0.3116
AP001505.1	7.9513	4.1494	2.2582	2.4966	1.4209	2.4609	1.0061
AC002401.4	7.1619	0.0277	0.9389	4.3893	1.4509	2.4693	0.5097
AC015922.3	5.877	0.6543	1.1216	2.3258	0.9693	2.7548	0.8397
AC023157.2	1.8351	4.7724	3.8329	0.7893	0.9044	5.4755	1.8287
AC010654.1	0.6167	2.1752	0.8877	0.0805	0.7547	0.4843	0.2942
AC006449.6	0.4041	1.4046	1.7675	0.3967	0.7025	0.7244	0.2782
AC135050.6	4.6366	7.1237	3.2458	2.6677	2.8622	4.6773	2.8692

AL359513.1	1.2031	0.7816	1.4233	0.4283	1.2714	1.1292	0.4534
AC099518.6	0.2673	1.7558	0.4614	0.241	0.1749	0.3727	0.099
AC068831.5	0.869	2.5206	1.5187	1.346	0.3165	0.6747	0.6148
AC005332.6	3.8295	3.8255	2.4232	2.2198	2.9025	3.0288	2.5228
AL136295.7	0.6443	0.9943	0.6764	0.3253	0.541	0.7454	0.1981
HEIH	0.8026	1.426	1.0015	0.8065	1.017	1.0078	0.4851
AC026401.3	24.0596	21.4443	20.994	6.8353	27.4549	11.0449	14.3011
LINC00997	0.6104	0.8105	0.8275	1.3769	0.2899	0.4701	0.6569
EBLN3P	8.3424	3.0813	5.2801	3.1024	3.6673	4.8356	2.2472
AC119427.1	0.2163	3.3931	3.5985	3.6273	1.5567	3.0919	2.3247
BISPR	0.5022	0.2224	2.0952	0.8333	1.699	0.8813	0.4196
LINC02635	0.0143	3.6518	1.6817	2.8891	0.0819	1.862	7.4842
AL132857.1	0.1824	1.6023	3.0139	0.1151	0.1139	0.0405	0.2222
AC103718.1	0.0128	0.1172	0.1126	0.5337	0.2127	0.9471	0.5055
AC092306.1	0.2771	2.9128	1.5222	0.6996	0.3173	2.3444	1.4378
AC083837.1	0.0412	0.149	1.273	0.2376	0.862	0.1196	0.1424
AL157829.1	0.8281	0.3264	0.3275	0.9406	0.5972	1.3067	2.7638
NIPBL-DT	1.0796	1.8009	0.7644	0.3563	0.6464	0.4915	0.1413
CERNA2	0.0367	24.72	14.927	5.4736	13.3731	2.8863	6.1679
AC108925.1	0.0598	0.0806	1.6287	0.4808	0.5216	1.1422	0.465
LCAL1	1.5166	0.2402	0.4616	0.3616	0.6779	0.174	0.1784
AL162253.2	0.798	0.3623	0.4171	0.4792	1.5324	0.3612	0.6677
AC046129.1	0.0322	6.9277	1.7353	0	0	0.0262	0.0279
AC010378.1	0	0.5795	0.5788	0.2078	0.6598	0.7635	1.4522
AC113410.4	0.6633	0.7391	0.296	0.2093	0.9492	0.3878	0.1971
PARTICL	1.1835	3.2516	1.0965	1.3318	1.3549	0.6959	0.3699
AC090004.2	0.0103	11.7264	2.1235	0.11	0.0411	0.2544	0.164
AL590550.1	0.7479	0.036	1.0744	0	0.0659	0	0.0497
AC097382.3	3.2324	2.7285	2.5824	0.9955	0.8676	2.7047	0.4547
AC108488.3	0.548	3.0941	0.4336	1.6997	0.8666	1.0161	0.2048
AL512625.3	5.0734	1.391	3.8301	0.8959	5.2106	1.4605	0.3249
AC234917.3	0.6297	1.3869	1.2635	0.8678	1.0299	0.5488	0.2
AL132712.2	2.4021	1.3909	0.5951	1.432	1.6968	0.449	0.1149
AC108477.2	0.643	1.5085	0.3532	0.2705	0.1636	0.3196	0.2162
AL021978.1	1.2634	0.1976	0.0771	0.6555	0.8196	0.2626	0.2306
AC067852.5	0.1585	2.0825	0.2438	0.16	0.871	0.361	0.1645
AC083862.2	0.4895	0.4134	0.8425	0.7208	1.0648	0.7698	0.1411
AC090994.1	0.0156	0.0876	0.2051	1.3842	3.7401	0.696	0.0471
AL353807.5	0.7506	0.4995	1.5442	1.1368	1.7031	0.9325	0.1416
AC127526.5	0.0424	1.3249	0.1551	1.0157	0.5657	0.5972	1.2452
AL354953.1	1.0693	0.6401	1.0936	2.7689	0.5277	0.7948	0.1275
AC245407.2	0.3858	2.1724	1.2715	0.2597	0.2356	1.0043	0.645
AC016394.2	0.9929	1.1388	0.3939	0.905	0.221	1.2114	0.4054

TCGA-IQ-76	TCGA-CV-74	TCGA-CN-69	TCGA-CQ-53	TCGA-BA-55	TCGA-CR-73	TCGA-DQ-75	TCGA-CQ-70
1.4937	1.3978	4.342	2.1045	1.958	1.4639	0.2105	5.496
2.1694	1.6274	3.6648	2.1865	2.4374	2.7096	5.3404	2.1639
1.2236	0.8893	2.5463	0.8619	0.4757	1.1902	1.3012	1.8625
0.2172	0.7388	1.7871	0.8141	0.5929	1.0059	0.9473	1.5027
1.0264	1.1157	2.5481	0.7745	0.7723	1.2808	1.0518	1.0489
1.2689	1.9303	3.1031	1.246	0.4749	0.7835	1.737	3.3636
0.4962	0.3451	1.7135	0.395	0.3385	1.1908	0.4406	1.3723
0.6167	0.2566	1.5201	0.5805	0.905	2.8169	0.6875	1.2046
0.2356	0.4097	0.1963	0.1525	0.2096	0.5691	0.1842	0.7037
0.3808	0.1714	1.023	0.4483	0.1797	0.3983	0.317	0.7624
0.8791	1.7367	5.0727	0.3232	1.2293	1.7926	4.5742	1.3631
0.3013	1.1466	1.9823	0.1504	0.3426	0.5108	0.7081	0.489
0.3706	0.9885	0.7418	0.5433	0.1619	1.2301	0.9303	0.9858
0.8601	1.5019	1.6865	3.1162	0.1322	0.5303	0.1886	2.0571
0.6336	0.5806	1.1894	0.993	0.603	1.6087	0.5142	1.9217
1.2578	0.2071	1.5623	0.6002	0.9774	1.4292	0.8679	1.3422
3.8298	3.7364	4.5325	1.7817	2.7765	2.1641	15.6269	4.5598
0.2743	0.664	1.9032	0.3477	0.2907	0.8845	1.1957	4.7
1.1036	0.5941	1.9485	0.8556	0.2198	0.984	0.8866	2.7802
1.8261	0.4178	3.2022	0.369	1.4842	0.6011	0.4601	0.8333
17.1734	11.5488	10.4374	3.2077	5.6239	17.224	5.2708	7.8024
0.8371	5.9312	5.7321	4.256	2.0302	3.9341	8.6517	11.0205
0.8546	3.6937	2.6667	1.2486	1.0898	2.2152	1.4237	3.6313
0.9699	1.6241	3.6356	0.5324	0.7956	2.2848	1.1502	2.1947
0.5777	0.8176	1.322	0.8591	0.6648	0.945	0.3472	4.5994
0.6496	2.7071	4.7056	1.6341	1.1794	1.6116	1.3713	3.3425
0.4345	0.1222	1.1593	0.296	0.4465	0.4245	0.7679	0.4226
2.5677	10.7902	4.1398	5.3357	2.6015	4.0554	4.6612	16.6315
0.57	1.1665	0.2861	0.2245	0.4162	0.1242	0.5246	0.2092
1.801	1.4636	2.8547	0.9081	0.6007	1.5767	0.2187	2.666
0.2578	0.6279	0.7369	0.3074	0.2434	1.1121	0.5718	0.7071
0	0.1133	0	0.0751	0.0149	0.5229	0.0374	0.0323
0.7632	0.1885	0.4401	0.2913	0.1736	0.6872	3.4323	0
0.4953	0.5707	4.5698	0.8122	0.3338	0.9108	0.3876	1.6488
0.4379	0.7093	0.7006	1.0653	0.579	1.4607	1.8713	1.3269
1.7086	1.1448	2.1802	0.5899	1.363	2.3175	2.1524	2.8866
2.9957	4.6661	6.7876	1.9352	1.913	3.4681	9.6394	3.3481
0	0.0841	0.8059	0.0867	0.5682	1.0517	0	0.755
0.266	1.7511	0.7562	0.8573	0.3424	0.7916	0.6013	1.0231
1.4025	1.9392	3.3739	1.9433	1.0008	1.2229	1.8433	3.9745
0.71	0.4716	3.0846	0.5561	0.2756	1.244	0.1923	1.0582
1.8813	0.661	0.4538	0.0521	0.7368	3.2286	3.2717	0.0672
0.7304	0.3573	0.719	0.3157	0.094	0.5637	1.0035	0.9571
0.311	0.1448	3.7089	0.8956	0.3214	1.1935	0.0457	1.4575
2.6788	5.7697	6.0384	3.6453	1.6021	4.9391	3.8457	6.7128
1.6402	0	2.0788	0	0	0.0603	1.0705	3.4044
0.3404	0.4164	0.9693	0.6709	0.5348	0.6355	0.4544	0.9475
0.2701	0.0909	0.9671	0.0562	0.4188	0.9095	0.1262	0.7981
1.031	0.0098	0.516	0.55	0	0	1.1322	1.2044
14.3967	8.0807	13.6728	18.4754	14.2252	15.9004	7.5166	21.9449

0.2233	2.0404	0.3698	0	0.254	1.1491	2.5506	0.0367
0.5625	0.5179	0.5818	0.7178	0.2796	0.7318	0.5576	1.3595
3.2742	1.2744	1.6451	1.1981	0.9348	1.7433	2.0398	3.1272
2.3813	4.5729	3.5054	2.5061	4.0983	3.4191	4.9571	4.747
0.7977	1.5228	4.3769	0.3162	0.2303	1.8943	3.3113	2.3346
0.3541	0.1795	3.7304	0.064	0.1526	1.3234	0.0319	0.6885
0.2985	0.2106	0.4086	0.179	0.2618	0.3949	0.6696	0.2625
0.4962	0.1741	1.0566	0.1495	0.0891	0.5644	0.3803	0.4824
1.1043	0.3148	1.5362	0.9073	1.0714	1.4273	1.7811	0.8082
0.5949	0.9687	0.8027	0.0675	0.2894	0.7456	0.5348	0.0609
0.1249	0.067	2.2051	1.5977	0.1616	2.795	0.0959	0.5885
1.1123	0.5589	3.007	0.9083	0.7788	2.8518	1.044	3.4801
0.1651	0.1631	0.9374	0.1891	0.0876	0.6088	0.11	0.7995
0.2125	0.2623	10.4809	0	2.7061	1.2026	0	1.2558
1.3938	0.5163	1.234	0.8149	0.4805	2.9924	0.5718	1.13
7.7468	1.6347	13.2462	1.8012	22.6167	8.1533	3.7403	3.0524
0.0767	0.0142	0.0613	0	0.0262	0.0296	0.0164	0.0142
0.0535	0	0	0	0	0.0275	0	0.0132
0.1857	1.7463	0.4212	0.2126	0.3856	1.7072	0.1798	0.9602
0.1478	0.4015	0.0525	0.1129	0.1009	0.8747	0.0844	0.0728
0.5947	1.1255	1.0029	0.5916	0.3939	0.8912	0.8962	3.7265
1.1041	0.755	7.9571	0.4863	0.4056	0.0656	0.2183	0
0.5807	0.3862	0.3554	0.309	0.1529	0.9882	0.5053	2.5269
0.9924	0.2902	2.6692	1.0169	0.3386	1.3303	2.1029	3.8588
0.2794	0.414	0.5492	0.2708	0.3667	0.9124	0.313	0.6987
0.6116	8.6085	1.8414	0.3389	1.0051	0.8629	8.4612	1.3312
0.4571	0.259	1.4314	0.2771	1.376	0.5396	0.7601	1.033
0.711	0	1.2131	0	0.0498	0.0352	0.3201	1.0102
0.2964	2.6126	0.8203	0.2715	0.5864	0.5261	0.9647	3.0214
1.7014	0.0756	0.1087	1.0911	0.2786	0.2889	2.8274	1.1061
0.0195	0	0	0	0.3192	0	0	0.0432
0.0992	0.0945	0.9809	1.0821	0.7705	0.8059	0.1255	0.3421
0.0468	0.5026	4.7825	0.3215	1.6283	1.9322	1.8238	1.2272
0.9919	0.6076	2.2512	0.4987	0.8996	0.9112	0.5782	1.2994
1.2228	1.0094	1.4507	1.1203	0.6913	3.128	1.4665	2.3747
1.3356	3.9192	4.9215	0.7449	1.3966	3.869	2.5631	1.7812
6.0388	0.2057	0.0739	0.159	1.6576	0.9107	2.7351	0.5128
2.7252	0	0	0	0.0326	0	0	0.0353
0.5087	0.1346	1.2766	0.4577	0.4215	0.7572	0.3735	0.6443
1.3437	0.6246	3.1136	0.528	1.6808	1.6369	1.3203	1.343
0.445	0.5934	2.0372	0.6115	0.3492	0.3263	1.2198	1.1178
0.6354	0.2071	1.2989	0.7568	0.1214	1.7263	0.8274	2.1594
0.9966	1.1007	2.5713	1.7855	0.6592	1.6611	1.2832	1.5404
2.0867	1.3913	3.499	0.7468	1.3704	1.1878	1.4076	4.066
0.5495	1.3006	0.0809	0.486	0.0884	0.4655	1.3163	1.0033
7.9315	5.5175	5.4579	6.4616	8.1618	4.1558	3.6181	12.7198
1.659	0.2304	0.8831	0.9499	0.5306	1.6005	0.3997	1.0341
2.7904	0.0267	1.1243	0.0216	0.1254	0.0981	2.1711	0.0457
3.1772	9.1672	2.5468	2.1078	2.3886	6.2676	8.2511	18.6132
1.2388	6.6075	4.6752	2.4166	1.0901	2.8848	2.3528	10.4396
0.9538	1.6936	4.0619	2.4793	2.3036	8.0276	2.516	2.2929

0.0887	0.2759	1.926	0.0812	1.0649	0.6434	1.1698	0.5372
1.3657	1.0748	7.1317	2.6203	0.9593	1.9215	1.2427	9.2393
1.0961	0.3085	1.5385	0.3927	0.2883	0.9263	0.1836	1.1536
1.1069	2.1867	0.2455	0	0.3462	0.9611	0.4741	0.1363
2.0766	1.8293	2.9975	1.6845	1.0763	2.584	3.5121	2.3652
0.3793	0.2342	2.1537	0.0724	0.6039	0.927	2.7888	2.4052
7.7108	10.0628	16.4737	13.495	5.1611	11.3364	13.9088	41.9481
0.4119	0.0832	0.2392	0	0.2555	1.1848	0.4811	0
0	0.6522	0.4686	0	0.02	0.0453	0.0503	0.0217
0.5287	0.5919	1.5913	0.4719	0.4434	0.8367	0.749	0.826
1.4464	2.3812	5.7605	3.1902	2.1932	4.7549	0.7342	1.781
0.9458	1.7197	1.19	1.1815	0.4107	2.0571	0.1473	15.8164
1.2442	0.9986	0.9199	0.2969	0.6485	1.1871	0.2665	0.8426
0	0	0.9465	0	0.337	0	0	2.2624
0.3963	0.6711	3.014	0.7781	0.2704	0.437	1.164	1.1712
0	0.4738	0.6053	1.3835	0.3394	0.3291	0	8.4001
0.149	0.0859	0.5464	0.2085	0.2485	0.4472	0.3404	0.2813
0.3164	1.8507	0.8334	2.2505	0.7386	1.2983	2.3397	1.1443
0.1477	0	0.8646	0.3382	0.2519	0.057	0.1897	0.3818
0.0916	0.0679	2.7317	0.6296	0.3543	0.7073	0	0.519
0.9326	0.1419	0.9752	0.515	0.2614	0.0964	0.1498	0.0738
0.2383	2.2069	0.1269	0.0682	0.2846	0.1686	0.2212	0.6456
0.1083	0.5079	2.3021	1.3096	0.0777	0.2185	0.3828	6.1786
0.6347	0.5225	2.6655	0.2221	1.3474	1.9731	1.7066	0.8727
0.3735	1.0884	0.8749	0.2281	0.5267	1.3454	0.3413	1.0854
0.2669	0.226	2.0701	2.0521	0.8585	0.4414	0.3919	3.3237
0.5716	0.6155	0.0882	0.1484	0.7147	0.0492	2.9631	0.1083
0.4328	0.0583	1.508	0.2704	0.1879	1.2755	0.1011	1.5406
1.5881	0.183	2.029	0.0404	0.0482	1.1713	1.8139	0.0782
0.6813	1.6403	3.8382	1.1378	3.4864	2.1033	1.0456	4.0476
1.1278	0.5523	2.4218	0.7224	2.4129	1.5343	2.2924	1.4546
1.7785	1.8741	4.5271	0.8721	1.8447	2.9505	3.2878	5.8751
1.9645	0.1604	3.3154	3.9674	1.3888	0.237	0.9679	2.4867
1.2608	2.0014	1.9815	1.8564	0.9012	2.5949	1.44	3.504
0.2146	0.2226	1.291	0.2458	0.8786	0.8448	0.2482	0.8007
0.0597	0.4201	0.7309	0	4.3174	0.1152	0.0256	0.0662
0.2778	0.3293	5.206	0.4454	0.1137	0.772	0.3332	0.6979
0.5773	0.802	0.7683	0.6199	0.714	0.2228	0.7109	1.1463
3.5537	0.0156	1.6258	0.1531	0.8835	1.1297	1.3683	2.4122
4.4911	1.3522	3.654	1.5028	1.6599	13.492	3.6805	12.4766
0.2535	1.1667	1.5882	0.5901	0.3799	0.3881	0.2741	2.5142
0.3853	0.2195	1.6012	0.1612	0.1062	0.6462	0.3998	1.3574
11.1585	30.1735	25.0085	20.1656	8.6082	19.7332	24.3698	47.754
1.0254	0.2057	1.865	1.0357	0.7482	0.9783	1.7812	1.7834
0.4197	0.3618	1.0956	0.1941	0.2946	0.7382	0.2558	0.5397
0.2679	0.3528	2.5349	0.4431	0.264	1.2864	0.3824	0.7586
1.6653	3.0409	14.3276	2.6493	6.5842	3.842	4.0791	4.07
1.4719	1.1274	4.0144	0.4786	1.1314	0.9011	2.4166	2.4064
0.6964	0.0769	2.4975	0.4058	0.6127	1.0656	1.7112	1.2953
0.3618	0.2791	1.7255	0.3322	0.1673	1.3686	0.4355	0.5302
0.4489	0.6953	2.5198	0.2804	0.3063	1.4173	0.804	1.7184

1.0596	1.4684	3.1454	1.0421	2.8494	1.8954	4.5383	3.9785
1.4753	0.3988	1.9102	0.8219	0.7073	1.7541	0.3757	1.6496
0.7556	0.4495	1.1579	1.1275	0.1094	0.0177	0.6865	2.4527
0.4424	0.9105	1.2038	0	0.5702	4.3254	1.7265	1.598
2.2327	4.2224	6.532	4.0328	0	2.7132	2.7729	0.0045
0.3502	0.49	0.9113	0.4901	0.9557	1.5315	0.6666	1.006
0.6563	0.1216	0.4658	0.0626	0.1866	0.5066	0.1406	1.6567
0	0.1681	0.2013	5.8462	0.0839	0.1532	2.7777	0.2724
0.5044	0.5267	0.9546	0.3971	0.1577	0.2838	0.8311	0.3105
1.7027	0.4046	3.3519	1.9377	2.9812	3.5044	1.1833	1.3608
0.9929	3.2286	1.7977	0.9532	0.929	2.969	5.256	1.3265
1.1879	6.3748	2.7722	1.812	0.8851	7.7711	5.8856	5.2446
1.5019	0.5337	0.845	0.3496	0.4999	1.3194	0.8682	0.4601
5.3481	22.613	16.6615	4.9428	5.8925	9.1107	16.3027	12.7093
0.4085	0.562	1.2427	0.4678	0.5575	1.4864	1.2498	0.3018
1.3126	0.5402	1.1258	0.4593	0.7961	0.591	0.0625	0.3771
0.1126	0.1252	0.4796	0.5482	0.1921	0.1956	0.8683	0.1664
0.6382	0.4826	3.6306	0.1294	0.7257	1.6161	0.6036	0.7808
0.3824	0.4856	2.4279	0.6412	0.4193	1.1804	1.8482	1.8361
1.2684	1.3	1.8842	0.8102	0.6987	2.9024	1.1975	1.218
0	0.1517	0.4362	0.7037	0	0	0	9.3822
1.9679	1.0934	2.1999	2.2537	0.3804	1.4429	2.2758	5.5732
1.234	0.2522	1.042	0.8771	0.2032	0.9196	0	0.8173
0.3577	0.7065	1.4914	0.3413	0.4677	0.9201	0.3574	0.6385
0.0639	0.5719	0.1542	0.0442	0.0058	0.0631	0.1603	1.0837
0.9446	0.4322	0.5028	0.0955	0.0379	0.4503	0.0714	0.0616
0.5896	2.2793	1.0788	0.3798	0.6411	1.4646	1.6571	1.9192
2.3009	3.8009	10.3334	5.9967	4.8902	2.674	8.7446	11.1955
1.1899	0.6049	0.8942	0.2271	0.6925	0.1441	0.4896	0.586
5.49	2.5179	5.1554	2.9387	2.74	4.9038	3.2972	5.9563
0.4777	0.5153	2.4894	0.7678	0.3831	1.1449	2.9003	2.4085
0.4394	1.3021	1.2334	0.7777	1.908	0.7708	1.1293	1.21
0.1458	0.072	1.1639	0.2226	0.1658	0.4875	0.0832	2.1538
0.7673	0.7433	7.9801	0.5407	0.4027	0.8655	1.8706	3.1394
0.6529	0.2199	1.9379	0.5664	1.242	1.1147	8.3214	1.4324
0.2225	0.2143	0.5448	0.1784	0.2885	0.2919	0.2859	1.5616
0.0784	0.4119	1.0245	0.3837	2.2325	0.5612	0.0305	0.7267
3.0714	1.4888	4.9655	1.5198	7.5288	7.0237	1.4941	1.7368
0.4463	0.5265	0.7215	0.2839	0.8007	0.6124	0.1982	0.8914
0.3788	0.8524	1.464	0.3214	0.8138	0.9205	0.9976	1.1817
0.1812	0.1566	1.4143	0.1383	0.2266	0.0699	0.181	0.9591
1.1794	0.7489	4.0063	0.4502	0.3832	1.6472	1.732	0.1245
3.8312	4.8889	5.3956	2.7964	1.2999	5.4509	8.4797	13.118
0.095	0.3635	0.0843	0.7432	0.3564	0.5253	0.0407	5.3088
3.9473	2.8311	3.6939	2.3322	4.2201	2.7361	2.3906	6.501
2.3079	1.326	2.0841	0.6629	0.421	2.9335	0.659	0.8497
3.4622	3.3167	12.9013	5.1271	4.8876	2.2115	1.1761	13.0979
0.8402	0.6916	0.8283	0.5844	0.2548	1.4026	1.2315	1.0943
0.3287	0.5913	2.3493	0.2688	0.0961	1.0871	0.5228	1.2487
1.6102	2.0473	3.4226	0.8913	1.0005	3.2124	1	3.0166
0.701	1.0108	2.0791	1.0608	0	0	1.1796	0

1.3133	0.5496	1.8999	1.2055	1.1013	2.7082	0.4981	2.3108
0.6355	0.7062	1.8607	0.3184	0.2168	1.8394	0.4763	1.9367
1.8859	0.137	2.4409	0.0847	0.0252	0	2.2173	2.6499
0.7529	0.665	3.4067	0.315	0.899	1.1622	0.7318	1.797
0.9054	0.1548	1.9278	0.319	0.095	1.0213	0.2983	0.7718
0.282	0.3072	1.4658	0.152	0.2377	0.5185	0.1989	0.5515
0.2258	0.0062	3.2413	0.067	0	0.9554	0.0215	0.0371
1.2549	0.3434	1.312	0.5697	2.2834	1.815	1.5883	0.6598
0.0365	0.0271	0.5055	0.0418	3.4891	0	1.0638	0
0.8856	0	1.1143	0.7376	0	0.0311	0.2069	0.0297
2.7353	0.6898	7.6624	0.5776	2.6076	1.3924	2.1769	1.6338
0.1161	0.043	0.6179	0	0.0792	3.1803	0.0497	0.1286
0.1369	2.5796	10.4762	1.1425	0.9344	5.7073	1.4076	3.1073
0.1498	0.2219	0.0797	0	0	0.0578	0.0641	0
0.7236	1.1726	2.0222	0.1036	1.3577	1.815	1.2396	0.3007
0.4255	0.0666	1.5503	0.0966	0.6782	1.2088	1.271	0.2263
1.0598	0.701	2.4984	1.3437	0.8264	1.4606	0.6809	0.9787
1.8938	0.0076	3.3131	0.0234	0.014	0.0237	3.7092	2.2007
0.658	0.5119	2.2069	0.2638	0.3143	0.8126	0.6482	0.1458
0.9971	0.4123	1.1603	0.5311	0.9177	0.877	2.0459	1.3705
0.5131	0.4372	1.0289	0.2644	0.2684	0.6799	0.2198	1.3647
1.0575	1.145	2.0208	0.6055	1.0546	2.6684	0.8943	3.4056
0.7162	0.8774	0.4252	0.3628	0.141	1.148	0.1416	0.5393
1.7645	0.6239	0.8112	0.6429	1.1492	1.1452	0.2405	1.4221
0.412	0.3249	1.0754	0.3196	0.2993	1.8362	0.4099	1.1194
0.094	0.116	0.8671	0.0359	0.2778	1.6561	0.0671	1.4463
0.695	0.8893	3.7446	0.8201	0.3305	2.7959	1.0464	2.2406
3.9511	1.2057	1.2735	0.8084	0.6422	2.5501	2.8471	1.7601
8.4304	2.9417	23.0892	6.1565	19.6327	10.9388	12.5593	17.376
1.2135	0.2547	1.2059	0.8107	0.7452	2.2791	0.4505	3.0333
0.2756	0.0922	0.3218	0.0204	0.0849	0.3225	1.5384	0.046
0.7693	0.2979	1.1913	0.7208	1.3242	2.0916	0.4643	2.5575
3.1199	0.8099	10.2204	1.2673	1.5011	4.5999	0.7538	1.2017
3.0248	3.3728	4.3823	0.8571	6.9363	3.5861	0.9616	2.0734
2.3559	1.7837	5.9261	1.4824	3.6743	3.6367	0.621	5.5085
8.1412	11.1111	4.5047	4.5112	2.3295	13.7156	6.6489	8.7308
11.1834	3.375	2.2167	5.5596	4.8456	7.8041	5.377	2.0332
0.5187	0.0027	0.2361	0.1188	0.227	0.359	0	0.4017
2.5968	0.862	3.4829	4.1712	1.6223	1.9002	1.0978	3.5007
0.329	0.5484	1.2589	0.4003	0.3017	0.9364	0.5637	1.1546
12.7757	1.377	10.6709	1.7244	12.1847	5.9381	3.0635	1.912
3.3729	4.3352	2.3982	3.1529	0.9189	1.9501	3.4508	4.5874
0.8528	0.6142	2.8876	0.4205	1.5031	0.9964	0.345	1.8288
1.6286	0.2068	1.6348	0.6395	0.2857	1.6161	0.7175	2.5095
0.4644	3.8582	0.3767	0.4242	0.7017	0.4054	0.2273	2.0953
2.4555	0	0	0	0.0114	0.2005	48.4073	0
0.8912	0.1969	0.4869	0.0716	0.1974	1.3124	0.6663	0.2801
1.5163	0.9237	1.1547	0.5261	0.9662	0.7624	2.2871	0.837
0.5291	0.1608	2.0366	0.1709	0.3425	0.356	0.3603	0.9522
1.6083	0.5581	0.8947	0.7585	0.8996	2.1151	2.2979	0.1881
0.2585	0.6094	2.377	0.0538	0.1924	1.9771	0.2416	0.3125

0.5419	0.446	11.859	0.8274	2.4649	1.7194	0	0.1779
1.4443	0.2205	0.763	1.1363	0.4815	0.485	2.7011	1.0752
0.4235	1.6849	0.2004	0.1976	1.4234	0.4358	2.1632	1.3906
0.3006	0.3897	0.4001	0.5164	0.0513	0.174	0.1288	1.1105
0.2062	0.2546	1.1707	0.1574	0.0938	0.6896	0.471	0.8632
4.3303	5.69	6.147	2.7751	4.8669	6.6599	3.231	8.9124
0.9249	1.1487	0.7241	0	0.4084	0.063	0.6758	0.6632
1.403	0.1356	0.4546	0.2096	0.5411	0.7533	1.2018	0.2704
1.2187	1.9992	0.6179	0.7643	0.396	2.5532	1.392	1.1148
0.1732	0.3266	0.6789	0.2434	0.0645	0.0061	0.8766	1.0934
0.5608	0.1479	0.8197	0.6096	0.2983	0.5062	0.6107	0.8778
2.7062	4.7402	2.637	2.0748	0.4851	3.3808	2.9272	5.3202
2.6345	2.6411	2.6095	1.3059	2.6562	3.3283	2.7059	3.2052
0.1863	0.4338	0.5667	0.8534	0.5811	0.4725	0.5472	1.9859
3.1716	6.9018	7.8507	0.7718	1.7853	4.1002	8.3202	2.226
0.8628	0.4337	0.3773	0.4235	0.3785	3.1157	0.4619	0.8765
0.6131	0.6468	1.8321	0.1796	0.666	0.9193	0.528	1.0858
0.149	0.3587	0.9516	0.2559	0.0762	1.236	0.1914	1.2656
0.5357	0.5669	0.9777	1.0079	0.3655	2.2741	0.4917	0.9329
0.9762	1.1634	0.9828	0.5468	0.8688	0.778	0.2363	0.9956
1.0002	0.6998	1.9522	0.9545	0.2654	3.3023	1.2376	2.0526
6.0353	1.8466	3.3904	7.1419	2.4741	6.0858	3.6483	6.6446
5.2346	0.1012	7.8022	13.4488	15.6842	4.4266	1.1307	1.2779
1.1774	0.0046	5.5609	0.6885	6.7517	2.2912	11.3573	0.5552
0.2662	0.0094	0.2295	4.9943	0.1557	0.5088	0.1195	0.1218
1.1942	2.1414	1.8893	0.2684	0.7615	2.3516	0.4971	4.1062
1.1914	0.7565	1.3742	0.8446	0.1839	2.8463	1.859	2.232
0.3629	0.3015	0.7041	0.334	0.1435	0.2435	0.3951	1.303
1.3539	2.3707	1.4414	1.3743	0.3149	2.1136	1.0543	2.3642
1.4449	2.703	5.3556	2.7735	2.5241	2.2353	2.5946	5.5243
10.5623	10.0004	6.994	7.3461	5.6073	11.592	19.2211	13.2275
1.9011	0.4263	0.8881	0.7998	0.3574	2.0113	0.7478	1.0032
0.0476	0.1763	0.1013	0	0.2598	0.2571	1.753	0.2813
1.2496	0.2222	1.1707	0.229	0.1705	1.8131	0.3425	0.8862
0.8552	0.1445	0.607	0.1804	0.3327	0.6659	0.3406	0.9588
0.9435	0.1398	1.1481	0.3396	0.3863	0.5618	0.5311	0.9361
0.7818	0.0399	1.2055	0.4631	0.4599	0.541	0.4388	0.7369
0.3954	0.5859	0.421	0.9057	0.045	0.8139	0.2823	1.5094
0.5529	0.582	1.2702	0.2999	1.1913	0.9208	0.5733	0.9674
0.6331	0.0469	0.6741	0.3625	1.0368	1.0262	0.1627	0.8887
1.0027	0.3455	2.1102	0.454	0.6841	1.4938	0.4394	1.7571
0.793	0.5813	1.102	0.5257	0.3189	0.9277	0.3361	2.0473
0.904	1.0046	1.4972	0.5752	1.5078	0.4652	0.4732	2.7828
1.6265	0.5594	2.412	0.4435	0.8456	1.1507	0.8791	1.4734
1.0887	0.3457	1.3799	0.2969	1.3796	0.7202	0.3553	0.8809
0.3806	0.094	0.4052	0.2906	0.1154	0.7834	0.7608	1.8122
0.4258	0.2734	1.2996	0.5526	0.1937	0.6573	0.6565	1.6148
1.9799	1.0807	1.6641	0.716	1.2443	2.8953	0.2678	1.3087
1.8248	0.9569	1.2224	1.3149	1.336	1.3926	0.1317	1.5375
0.4657	0.3764	1.848	0.2909	0.1011	0.9149	0.6165	2.2989
0.6558	0.2776	0.5985	0.5365	0.3836	1.0124	0.8828	2.2149

0.2943	0.6796	0.4976	0.337	0.2362	1.1757	0.5486	0.5755
0.9795	0.7625	1.4847	1.0171	0.5494	2.6139	0.967	1.2449
0.6004	1.2131	1.5546	0.1094	3.082	0.2738	0.0818	0.252
0.8231	1.0976	0.5112	0.864	0.2434	1.2494	0.5758	1.4087
0.0901	1.1352	1.1836	0.1262	0.1025	0.5411	0.5748	0.5698
1.9168	1.9525	3.2141	1.5366	1.8637	1.2205	0.6157	2.5489
0.4477	1.6583	0.1589	0	0.3055	0.9214	0.6392	0.7718
1.8571	1.2284	1.4123	0.8355	2.1044	1.459	0.4545	0.9065
0.5532	2.112	1.7215	0.6335	1.5388	1.2808	0.2552	3.5837
0.9141	2.9021	1.1122	0.2991	0.5346	1.1086	1.1186	0.6753
0.7233	1.034	2.0665	0.512	0.8904	1.3774	1.1302	1.5333
2.5265	6.8176	3.1219	1.5741	1.2058	1.1518	1.8278	0.6286
2.4762	0.5809	0.3076	1.4887	2.7244	6.0998	0.1414	1.6007
2.1025	1.7612	1.7938	1.4062	0.928	4.072	2.541	4.0058
0.1311	0.1359	1.3257	0.1801	0.1341	1.0824	0.4154	0.0387
2.0153	0.5484	0.3065	0.2355	0.1684	1.4602	0.9512	0.1519
0.5261	0.3038	1.0745	0.2533	0.3604	1.054	0.1067	0.4569
0.6926	1.2827	0.6964	0.4406	0.2625	0.7127	0.9229	1.5919
0.4542	0.1346	0.2901	0.2601	0.5578	0.631	0.5447	0.6375
0.9537	0.2136	1.9363	0.4572	0.4237	0.8217	0.19	2.1629
0.0765	0.0567	1.7925	0.0876	0.2089	0.6497	0.7867	0.3958
0.3522	0.5263	1.2476	0.445	0.1367	0.9982	0.8218	2.0769
0.5712	0.8079	3.8332	0.2973	0.5905	0.8417	0.2966	1.0103
0.4326	0.1602	0.3684	0.2972	0.4132	0.4006	0.4446	0.3196
2.3699	0.3234	0.8632	0.1428	0.0851	0.8664	1.2287	1.3823
0.9624	0.115	1.0576	0.32	0.7202	1.9408	0.585	0.8715
0.8422	1.1038	1.9312	0.5935	0.2873	1.8	0.9989	5.7912
2.2519	1.089	3.6962	0.9313	2.1554	1.4242	0.9109	1.8254
0.5574	0.1332	1.0146	0.3089	0.503	1.0685	0.4929	0.8767
7.31	31.8174	15.2959	4.6346	9.5958	8.0041	4.3117	5.1015
1.0446	1.0318	1.0298	0.6646	0.396	0.5375	0.1657	1.4292
5.7894	0.2571	7.0386	5.4378	6.6044	6.6672	1.8304	0.7826
1.0807	1.1119	1.9176	0.4125	8.7254	0.4634	0.1543	1.375
1.7612	0.4289	1.31	0.1105	1.1688	0.9124	1.5913	0.3921
3.0982	0.5962	3.6091	1.8316	0.6109	3.6645	3.5587	2.6085
0.6655	1.2998	0.8481	0.3118	0.6054	1.1518	0.2937	0.7151
0.2925	0.365	1.1474	1.2871	0.5148	1.0101	0.5935	1.4332
0.8608	0.3478	1.333	0.4033	0.1602	0.7549	0.2011	0.318
0.8608	0.4638	1.4996	0.3136	1.7886	0.4228	0.3016	1.0406
0.4674	1.4197	1.891	0.9099	0.1914	0.974	0.6006	2.3826
0.518	0.2345	1.5626	0.8239	0.5498	0.8662	0.5916	1.7009
1.9998	1.9883	2.8201	1.4866	1.568	2.058	0.3757	4.199
6.5457	2.8401	1.3902	4.8504	9.8236	9.3677	10.2391	0.2474
1.3404	0.1623	0.8645	0.4428	0.1231	0.9152	0.3864	0.5428
3.6786	4.3673	2.4101	2.2684	1.0297	3.3488	8.6051	4.5644
1.4693	0.9026	0.3052	1.2312	1.3449	0.3319	1.8726	2.3034
0.4845	0.2467	4.8678	0.4508	1.0743	1.3555	0.1816	0.2237
2.4315	0.8673	12.3682	0.126	4.7655	1.3669	1.5	1.0571
0.6464	0.249	0.6332	0.1184	0.5469	0.6586	0.598	0.3056
0.4641	0.209	1.802	0.4168	0.385	1.1167	0.8652	1.0555
3.7032	2.1906	5.914	1.9622	2.3759	5.7804	2.462	6.4109

0.8252	0.4446	1.9967	0.2148	1.3309	1.5344	0.3534	2.1338
0.4643	0.2752	0.1483	0.5849	0.2218	1.505	1.1534	1.1662
1.1527	0.8895	0.2557	0.33	0.2949	0.4078	0.4937	0.5323
3.2974	3.0309	4.9498	2.7195	0.9712	2.6388	3.7007	2.6157
0.4179	0.4514	0.76	0.4386	0.3326	1.2766	1.0589	1.1962
1.0517	0.6458	1.4224	0.9983	1.015	0.841	1.0714	2.114
15.0182	23.9712	8.4486	12.56	6.8207	13.4195	18.4246	12.4952
1.0975	0.3276	1.6897	0.7234	0.2317	0.4327	0.7711	0.8633
1.8898	4.0996	6.5934	3.1265	4.2392	4.4709	1.467	10.1421
5.1481	2.2827	4.2807	1.9795	3.769	4.1183	2.5107	2.6651
0.3362	1.1691	0.8474	0.6993	0.3886	0.9214	0.1352	3.3861
4.6448	0.3995	7.4639	1.8129	4.6173	3.3432	0.6408	1.5551
0.0101	0.0523	1.2554	0.3116	0.0275	0.5912	0.1209	0
0.2162	1.4947	0.6884	0.2007	0.1781	0.9124	0.5222	2.8301
4.3134	1.4479	2.0091	4.0134	0.9657	3.7973	6.5237	3.7343
0.279	0.71	0.495	0.3276	0.1659	0.6293	0.0368	2.6051
1.0572	0.7608	0.343	0.2306	1.2023	1.904	0.4744	2.7151
0.6835	0.2788	0.7597	0.3655	0.3903	0.9319	0.2063	0.634
6.5332	8.9777	10.0417	3.8686	3.8872	8.5532	4.4504	4.3324
0.1907	1.6214	1.1845	0.0988	0.5484	0.2313	0.7702	0.6374
1.426	0.7398	0.9841	0.0851	0.187	1.4914	0.3501	0.0789
0.493	1.9883	1.4079	0.1434	1.1426	0.8032	0.2748	1.4799
0.0784	0	0.1669	0	0	0.0303	0.638	0.029
1.3315	0	3.3467	0.5847	6.1271	2.3721	0.0343	0.0222
0.6049	0.168	0.5635	0.0289	1.0146	0.214	0.4965	0.9123
3.0516	0.9377	1.1629	0.695	0.8991	2.4623	0.7278	2.8565
0.823	0.0092	1.1351	19.9279	0.034	4.0325	0.0694	0.6679
0	0	0.0248	0.0534	0.0318	0.144	0.02	0.0345
1.448	0.8916	3.0833	0.3876	0.6416	0.8418	0.2739	1.5005
2.3095	0.64	1.7374	0.2845	0.5433	1.2813	0.2999	0.4339
1.5486	0.7711	3.3514	0.6977	0.7275	0.6662	0.4567	3.2258
0.3907	1.0361	0.6239	0.5279	0.3305	1.3629	1.1043	1.2006
0.8203	0.6537	2.9376	0.3274	0.0763	0.7866	1.0647	1.4049
0.8687	0.9332	1.5723	0.6467	0.2075	4.6264	0.186	0.1604
0.0853	0.1012	0.6542	0.1173	1.8052	0.1713	0	1.3367
0.9251	0.3141	0.4925	0.309	0.7628	0.3868	0.2972	0.6835
0.496	1.6316	0.6337	0.4772	0.8258	0.4747	0.153	2.7704
0.0662	0.4414	0.2819	0.639	0.0581	0.3285	0.6481	1.7119
0.5144	1.8563	0.5477	0.6081	0.2944	0.7429	1.3789	2.9424
1.5451	0.8012	1.1149	0.3342	1.1362	1.7888	0.4559	3.0186
3.5643	0.2325	0.3103	0.3081	0.6118	0.6056	2.1123	0.4803
1.0009	0.3939	0.2997	0.1791	0.5335	0.6035	0.0804	0.6008
0.4024	1.4409	0.7855	1.3058	0.1373	0.6989	0.2873	0.5203

TCGA-CV-71	TCGA-CV-71	TCGA-CV-74	TCGA-CQ-62	TCGA-CV-72	TCGA-CV-74	TCGA-CQ-62	TCGA-CV-70
4.6457	6.9244	0.1561	0.348	1.2906	5.9475	1.2011	1.2788
3.0517	6.4422	1.8083	19.1488	2.95	6.1045	3.7305	3.6051
1.5704	1.5767	1.5907	2.3098	2.3261	1.636	1.2182	1.3108
0.9498	2.8885	1.0733	3.1244	0.7619	1.9122	1.2738	1.2537
1.7005	1.3049	1.5361	3.4185	1.1957	1.703	1.0421	0.469
2.0528	1.7218	1.5737	2.5784	2.4944	1.1741	0.914	1.8072
0.7525	1.8527	1.0524	3.264	1.8227	1.1097	0.716	0.6728
0.7256	2.3657	0.6782	2.406	3.761	1.4998	0.8031	0.4064
0.6967	0.9676	3.2715	3.017	0.5927	0.3907	0.2484	0.2908
1.9488	0.8345	0.5327	0.9089	0.5782	1.2165	0.3728	0.2357
3.2962	0.0124	0.953	0.1227	1.6428	0.1831	0.9159	2.7903
2.4277	0.2814	0.7709	0.7858	0.2503	0.9483	0.5595	0.6763
1.3803	1.4049	1.373	1.0946	0.8195	0.6417	0.5932	1.0698
1.3047	0.0366	3.0871	3.052	2.9376	0.1609	1.7712	1.1561
0.7562	2.4675	1.5338	4.7612	3.6373	0.9419	0.6955	1.0388
2.0645	3.6588	1.1978	4.4748	2.8515	3.2037	2.9822	0.6613
7.5772	9.5558	2.7736	3.9803	1.6618	6.8481	9.7016	1.227
1.1105	1.1317	3.2927	1.0922	0.9411	1.006	1.0309	1.0428
1.2622	2.3252	1.2094	1.384	3.0515	1.2018	1.6223	1.0433
1.716	1.987	0.9432	3.7228	0.797	2.7186	1.068	0.3389
6.2313	10.7152	6.2012	24.8912	10.306	7.9328	12.5379	6.1199
2.9499	6.9419	2.0556	9.665	2.5902	3.8791	1.0483	3.7623
1.2837	5.9104	3.026	3.197	1.7797	2.5724	1.3949	0.99
2.3678	3.7484	1.3365	2.2728	3.9156	1.1016	0.7045	0.7216
2.3462	4.6426	2.4371	0.4742	2.5271	0.8286	1.4804	0.8568
13.7977	3.4201	3.0266	1.5907	1.7731	3.2338	0.9452	2.2963
0.4912	0.4507	0.3127	0.7872	0.759	0.6697	0.2347	0.2906
13.974	20.0338	12.0248	1.5317	3.3602	4.2897	11.8848	3.877
0.6295	0.4161	0.0789	0.0274	0.4202	0.934	0.1679	1.173
3.9618	7.573	1.9162	0.0304	2.3734	2.1198	0.5215	1.627
3.2735	2.0824	0.9066	2.3547	2.1932	0.6995	0.3186	0.4345
0	0.0995	0.0365	12.1349	0.0811	0.0802	0	0.0172
1.8136	1.0498	0.0798	2.4677	2.1943	1.8429	0.0308	0.0251
2.6258	2.5961	1.3191	2.7271	2.1821	1.0227	0.9015	1.1187
2.4869	3.7934	1.3902	3.6812	4.8129	2.0181	0.7535	0.956
1.8991	1.9592	2.3848	4.5717	3.3278	4.2487	2.1448	0.9412
1.4514	16.9625	5.6202	3.7616	4.0179	5.9447	8.7917	1.4235
1.9245	1.9388	0.5064	0.1711	0.2527	0.5275	2.4231	0.1791
1.464	2.5284	1.3712	1.002	1.0682	0.7584	0.5479	1.3754
4.0974	2.0413	2.4037	3.1619	3.2926	1.5943	0.9917	1.9588
1.2926	4.9702	0.1263	6.3394	0.2483	0.7663	0.8827	0.4924
1.0232	0.2006	0.5228	16.7685	5.9122	1.2216	2.0894	0.5648
1.9791	1.6943	0.7491	2.44	0.7055	0.364	0.1203	0.1739
0.7083	1.7399	0.1709	3.7762	1.477	0.4955	0.0431	0.3644
4.6697	9.7878	9.6791	6.1107	9.2139	6.4048	3.9858	3.7687
0.1342	0.0445	1.3389	8.2382	7.7073	0	0.303	0
0.5385	1.7483	0.993	1.9027	1.0691	0.8363	0.6604	0.1061
0.9109	0.3074	1.5603	1.7757	1.2387	0.1981	0.1667	0.0581
0.4269	0.005	0.0074	1.6332	0.948	0.0129	0.0043	0.4899
10.5734	21.7691	10.5021	13.6825	16.6187	19.5873	11.8288	17.2523

0.2728	0.5084	1.3072	11.198	0	0.7281	0.2648	0.1174
0.9276	1.9483	1.4313	0.2058	0.4114	0.6366	0.5846	0.6084
1.5309	3.4331	3.0198	6.9223	3.631	2.9317	2.883	1.764
3.766	5.285	5.4064	10.1531	5.0784	3.3181	6.11	0.4406
3.162	3.8764	3.6173	4.6229	3.1185	2.2956	1.3094	1.7904
3.3424	3.4581	0.0779	6.6839	0.1659	0.1231	0.7774	0.7055
0.6836	0.6795	0.7012	1.7882	0.3269	0.3128	0.3722	0.1457
1.0049	1.3675	0.2839	2.3218	0.4844	0.5747	0.6333	0.7826
0.4414	0.9813	0.7123	3.1547	5.1337	1.5464	0.4599	0.5665
0.3885	0.1475	0.4334	0.0621	0.2185	1.6679	0.24	0.9058
0.216	0.5196	1.9012	4.0552	3.524	0.0474	0.4427	0.0611
3.0373	3.4793	1.7471	4.0737	1.8516	1.9655	1.3604	2.441
0.2395	4.698	0.4141	0.6219	0.3811	0.2288	0.3381	0.1591
2.1412	0	0.6515	1.7075	0.0525	18.34	0.2061	0.1117
3.8031	3.2901	0.2024	1.7963	3.4617	1.5714	1.3235	0.998
4.9841	1.3628	3.5334	39.5112	4.7724	4.6324	3.871	4.4061
0.0527	0	0	0.5204	0.4413	0.1407	0.0558	0
0	0	0.0149	15.5392	0.6487	0.0916	0	0
1.1871	1.1395	0.7763	1.9463	0.575	0.379	0.9162	0.6876
0	0.0561	0.1236	5.0489	2.1571	0.4699	0.1912	0
5.6701	1.9073	2.3849	1.0433	1.2658	1.2515	0.8674	0.3903
0	3.577	0.2131	1.7916	0.5671	0	0.5767	1.0047
1.3353	1.9571	0.8948	1.4849	1.5878	0.4461	0.8383	0.7321
3.23	1.7837	0.9827	2.8137	0.9689	14.1771	0.8613	5.8698
0.4135	1.5415	0.8448	2.3644	0.7177	0.5992	0.7089	0.356
1.1522	1.6918	0.9129	1.0454	0.1952	4.3177	2.4622	0.9231
0.1848	0.9488	0.8994	2.5931	0.8579	0.2367	1.4606	0.3181
0.0313	0.0311	0.8995	2.1839	0.886	0.1271	0.0619	0.0216
1.0487	1.4843	1.1152	0.48	1.0994	0.7174	0.7761	1.0751
1.169	0.0775	1.3373	5.2555	0.7322	0.624	0.5611	0.2415
0.067	0.0887	0.0652	24.4629	0.2314	0	0	0.0154
1.2629	0.1882	1.4618	0.7511	1.9105	0.1456	0.2154	0.7974
1.8809	2.4235	1.8195	0.2115	0.6423	3.1237	1.2709	0.9963
0.8893	0.9831	0.9367	1.3634	0.3841	1.15	1.0724	1.3241
1.4884	1.9885	3.2137	1.7899	2.3589	1.871	0.9489	1.5981
2.4611	4.0279	2.1829	9.1674	1.7117	5.4333	2.6331	4.1311
0.2385	0.079	0.7546	68.8203	1.854	0.3055	1.4139	1.2043
0	0	6.0768	21.9363	0	0	0	0
0.5742	0.9099	0.9723	3.5314	5.3649	1.1462	0.3524	0.3152
1.6745	4.8734	1.2558	4.5059	2.8442	2.039	3.1561	0.6649
1.6206	3.0899	0.7442	3.118	1.4197	1.4853	0.7122	0.0702
1.1526	0.868	1.4028	5.8214	2.0179	1.0069	0.641	0.1804
0.9033	1.8096	1.3651	2.658	4.7272	1.9601	1.5479	0.9403
2.079	2.3456	2.0503	5.1094	4.5673	4.1713	1.3915	1.625
2.652	0.5136	1.1841	0.5789	0.2884	0.5768	0.156	0.5214
6.3803	26.535	4.5564	15.7894	12.2482	19.7969	7.3181	9.8148
1.1755	1.2393	1.0838	3.5544	0.4231	0.6465	0.6034	0.368
0.0909	0.0782	0.0244	2.462	0.0994	1.3453	0.025	0.1246
9.1586	3.4837	8.3739	8.5517	4.6084	5.2879	6.3745	5.2789
3.8479	3.5107	5.3616	6.7649	6.7033	1.6013	1.9308	1.5362
1.6332	4.5152	0.5444	1.6311	2.2129	1.7104	1.9384	2.3999

0.5728	1.4536	0.5339	0.4677	0.3027	1.4052	0.5333	1.021
3.4705	8.7515	2.8794	7.6106	2.608	1.465	1.2335	1.3807
1.2454	0.8295	0.6657	2.136	1.313	0.6918	0.8078	1.0914
0.6338	1.89	1.6969	2.0849	1.1635	1.4548	0.85	0.5092
2.7897	3.4852	3.9294	6.6443	4.7754	3.6815	2.5437	2.3831
0.5864	0.6117	2.9867	4.2388	1.1491	2.5738	1.349	1.8446
62.4746	17.245	19.7295	50.9837	30.1997	7.8057	4.989	8.0035
0.3859	0.341	0.6262	12.5249	4.1115	0.1923	0.2179	0
0.0403	0.2338	0.0491	2.255	0.8926	0.0215	0.0285	0.0463
1.5963	1.7518	0.7052	1.6279	0.7638	1.7996	0.8352	1.018
3.276	1.2196	3.6734	0.5651	3.2593	3.8906	2.754	1.5209
2.304	4.3063	6.6864	0.1296	1.4672	0	2.8355	1.3561
1.4368	1.672	0.5925	2.9164	2.1541	0.9128	0.5029	0.4225
0.0679	14.4494	0	0.1861	0.2199	0.4348	0	0.039
3.5403	1.4179	1.373	2.9861	2.3525	1.0799	0.7689	0.3126
0.6348	0.5662	2.6741	0	1.2127	0	0.7582	1.1769
0.7849	0.603	0.4153	2.7565	0.8598	1.2023	0.3051	0.235
0.4806	2.1707	1.4135	1.8194	1.075	1.2095	0.7916	0.7683
0.1522	0.5883	0.1235	4.5609	1.5337	0.3791	0.0716	0.0582
1.0494	1.2169	0.4853	1.0356	1.3598	1.4789	0.2667	0.3613
0.3319	2.4361	0.578	1.7002	0.2348	2.2724	0.9693	0.1313
0.191	1.2434	1.7936	0.0599	0.0147	0.8013	0.8669	0.1096
1.1539	3.7315	0.687	0.1297	0.0783	1.5211	0.4658	0.3218
1.163	0.4415	1.9166	4.0913	0.8765	1.4227	1.505	0.4171
0.3422	4.0535	1.7492	1.8011	1.0162	0.548	0.7005	0.3731
1.3622	1.5623	0.0956	2.183	0.3112	0.2517	0.3328	0.1804
0.784	0.3821	0.4655	0.0368	0.0441	0.2619	1.4715	1.188
0.4866	0.2239	0.7567	1.8083	1.051	0.6927	0.8778	0.2793
2.1582	1.2855	1.0624	37.1185	14.8994	5.6957	9.6535	0.0557
1.97	1.2602	2.4212	9.4317	5.2023	2.4364	1.5695	1.5447
0.7486	1.1097	0.8632	1.0225	1.3615	1.0377	1.0569	1.2964
2.0332	2.1292	5.0729	2.3565	6.8028	2.3957	2.4787	1.846
0.2435	0.8873	0.7703	0.3381	0.111	2.9602	0.8325	0.2825
4.4139	9.3626	2.6608	10.3129	3.2072	1.3653	1.6888	0.9469
1.047	1.2948	0.2602	2.6356	0.4618	2.0939	1.2282	0.2285
0	0	0.2246	0.1349	0	0.0438	1.3896	0
0.7636	0.9488	0.697	2.6793	0.6184	0.9375	0.8085	0.3944
0.8182	0.6572	0.513	0.1903	1.5261	1.2177	0.245	0.7399
0.7446	0.1842	0.0059	0.4719	0.0522	0.8311	4.7098	0.0111
11.667	5.5176	10.1401	18.8209	2.9189	12.0959	4.4693	3.5245
0.8537	1.7312	1.2787	0.9486	1.113	1.3199	0.7376	0.3146
0.7127	1.1048	0.5204	2.8467	0.4453	0.4185	0.4312	0.1636
30.6223	25.804	32.1256	34.5616	36.0254	11.0125	7.4107	24.497
0.3963	1.0456	1.0897	1.2769	4.3536	1.0604	1.0844	0.5897
0.6267	0.4548	0.9787	2.0372	1.572	0.4145	0.7673	0.3628
1.0941	0.9316	0.5351	1.592	0.276	1.703	0.8372	0.4812
2.3689	3.9142	6.8423	19.5658	6.1178	2.9583	6.8367	1.8315
1.3141	1.0756	0.6534	2.2898	1.2101	2.2028	2.1548	0.4335
0.1133	1.1335	1.2567	1.8134	1.3145	1.4027	1.4814	0.5059
2.5909	2.2942	0.7051	0.8944	0.6745	3.8563	0.6381	0.5612
0.3645	0.6968	0.4436	2.7669	0.6964	6.1666	0.5146	0.2574

4.3769	3.0367	1.574	5.4849	1.8868	3.9597	0.8222	2.4479
1.2739	1.2254	0.9836	1.9676	1.6271	1.6818	0.7155	0.8018
0.409	0.8731	0.3063	1.518	0	0.21	0.6996	0.4604
1.5876	0	0.9455	1.5926	1.8237	4.1111	0.6676	1.4344
5.2384	7.342	1.1182	10.6261	4.4803	4.6916	2.2537	0.0048
0.0267	0.93	0.7483	1.6415	0.9237	0.4852	1.2076	0.0614
5.4491	0.5603	1.555	0.5769	0.0406	0.7623	0.3714	0.1725
0.1039	0.0323	3.9052	0.0427	0.2806	0.0485	0.0275	0.6263
1.0998	0.5183	0.3192	0.4407	0.1819	8.6641	0.9785	0.3259
3.1198	1.3409	1.0925	9.5849	1.6368	15.1145	1.1842	0.7178
4.1833	0.6023	2.4113	1.2587	2.3645	1.3477	0.8708	2.8743
5.4164	8.6703	3.5999	8.9139	8.7961	1.7568	1.5163	2.773
0.8055	0.6255	0.6841	1.104	0.6614	0.3941	0.4027	0.1926
12.3404	6.1826	17.6131	7.6985	6.789	4.0614	9.3636	13.1899
0.2807	0.7307	1.0736	0.4837	1.9485	1.7125	0.9623	0.6443
0.4009	0.332	0.6708	4.1206	0.3787	1.2572	0.7074	1.0927
1.1608	0.2244	0.1884	3.8397	1.2535	1.8384	0.1912	0.3775
2.608	0.5221	5.3919	0.5709	0.3847	3.8182	1.354	0.7863
1.4356	0.8394	2.3751	3.5185	2.5836	3.3959	0.4371	1.0338
2.4846	1.7872	1.7943	2.1737	2.2951	2.1165	1.0118	0.9433
0	4.1969	1.3703	0	0.152	0.1503	0.3974	0.1615
1.9832	1.6427	1.3439	0.6178	1.8982	1.2512	1.368	1.0088
1.1987	0.8719	0.7828	5.8667	6.6932	0.2809	0.6604	0
1.6178	0.5428	0.4237	0.6287	0.3096	0.962	0.5782	0.3056
0.0271	2.8878	0.0071	2.4172	0.644	1.7072	1.0726	0.1691
3.818	0.1898	0.999	0.4605	2.721	0.2242	0.0808	1.9062
0.9368	2.6635	0.6933	2.096	0.7382	1.2705	0.4646	0.8573
3.6343	7.2551	6.7229	8.0099	4.003	4.1324	3.6427	3.3162
0.1122	1.1154	0.3219	4.7196	2.0513	2.5074	1.8783	0.1472
3.6281	6.0246	3.5437	6.7341	5.9361	6.5795	5.0978	2.3934
3.2823	2.156	1.4296	1.6732	1.853	4.8814	0.9592	2.8486
1.9214	0.3638	0.2338	8.6379	1.7487	1.055	0.6975	0.5671
3.3552	1.2721	5.1803	1.7388	0.7931	0.3743	0.1414	0.3065
0.5677	1.8811	1.678	4.9801	3.9849	2.7709	1.7747	0.1862
1.1283	3.4458	2.2997	7.14	3.391	2.7286	0.8828	1.217
0.7797	0.4812	0.5954	2.3133	1.6178	0.4407	0.4101	0.3334
1.6212	0.8114	0.6557	0.1128	1.3434	0.8105	0.3526	1.0793
2.2667	1.4675	2.4098	5.6564	6.6114	1.057	1.508	0.8074
0.5452	0.1693	0.8155	0.7721	0.9075	0.7639	0.1924	0.0391
0.5303	1.0062	0.9269	2.1566	1.697	1.2867	0.4491	0.3762
0.6846	1.306	0.303	1.5013	0.224	1.0631	0.4393	0.1191
0.7718	0.0639	0.2348	4.2737	0.8334	3.9965	1.9067	0.2658
8.5736	10.4415	9.4615	18.3537	6.841	8.7713	4.2675	5.4203
0.7069	0.7747	0.6353	0.0477	0.2936	0	0.5373	0.674
3.6623	4.436	3.6161	8.6754	7.0699	3.6149	4.8286	3.2715
0.6071	2.3236	1.0955	4.149	2.6392	1.3242	2.0982	0.6187
1.8867	2.5141	4.5923	3.9127	7.7508	6.9189	2.7793	1.6947
0.6842	3.5351	0.6193	1.9553	0.845	1.2921	0.8693	0.3828
2.0646	0.6948	1.0993	1.3442	0.209	0.6544	0.4099	0.5554
3.2474	4.0477	2.0657	3.9538	2.7032	2.0852	1.8764	1.6901
0.3096	0.0142	0	1.509	0.065	0.5326	0.0607	0

2.7485	5.0669	2.7246	10.7708	4.2399	2.6255	2.2269	0.9883
0.8461	2.9841	0.4982	2.1846	0.6484	1.3403	0.4623	0.5638
1.2958	0	0	2.1452	0	0	0	1.079
1.4226	3.5764	1.1865	2.8798	1.6866	2.7828	1.264	2.1352
1.0049	0.4756	0.5824	1.2593	0.465	0.6642	0.608	0.6591
1.2766	0.0944	1.0334	3.2744	0.3015	1.15	0.3701	0.085
0.1379	0.1047	0.035	20.6496	0.2668	0.0552	0	0.0264
1.6313	0.489	0.5672	2.4531	0.9143	2.6125	1.1953	0.8648
0.0753	0.0832	0.2749	1.7062	0	0.3215	0.4606	1.1522
0.5532	0	0.6059	3.5182	0	0.4725	0	0.0317
5.2384	1.612	1.5086	5.4223	1.2378	3.3869	1.6182	0.6884
0.0798	0.3303	1.6015	8.0453	0.0431	0.1703	0.1689	0.0458
1.9894	2.0487	3.7952	42.6535	0.3484	8.0938	2.0114	0.2777
0.5145	0.2557	0.5636	4.175	1.8335	0.1648	0	0.0591
2.983	0.5662	1.5883	10.9368	1.0737	3.1516	1.7546	0.7133
3.3734	0.6889	0.9217	0.3219	0.0313	0.7575	0.2704	0.201
1.3783	0.8186	0.9495	0.7414	2.1343	2.6377	0.8444	0.5671
1.8006	0.0117	2.003	2.8073	0.4709	3.597	0.5461	0
1.266	0.5243	0.3302	4.2141	0.0976	0.7482	0.383	0.5189
2.1988	1.0294	0.7174	1.8868	1.0667	7.3656	1.2596	0.9692
0.5347	1.0222	1.0298	1.6559	1.3896	1.1481	0.7549	0.4451
1.3601	3.0249	2.4602	3.0644	4.2652	1.7704	1.6439	1.5611
0.3123	1.5677	0.7831	0.5914	0.4905	0.4445	0.8149	0.4888
0.7991	0.913	0.9389	1.3596	2.3505	1.3826	0.7391	0.0949
1.5525	2.6173	1.1558	1.4719	2.2878	0.8482	0.3481	0.4821
0.1507	0.2852	1.4537	0.826	1.255	0.2757	0.2734	0.1359
1.8958	2.1097	1.497	1.9676	2.1721	1.4832	1.5526	1.1128
2.1288	0.6138	2.1316	3.0876	2.4369	1.2371	4.4316	1.4304
6.884	4.8677	20.7402	36.8206	69.576	12.1896	12.029	5.7813
2.6404	4.7427	2.1648	5.8514	2.1159	2.4778	0.3531	0.7018
0.0305	0.5364	2.2675	0.576	0.2705	0.013	0.9141	0.0421
1.6578	1.0548	0.9942	5.0977	2.1793	1.2184	1.1701	1.0617
2.7942	1.4874	1.4717	45.4444	14.4827	3.5407	3.5048	1.6088
2.2283	1.5973	3.103	10.6897	13.4656	1.098	2.7827	2.8773
1.5298	1.6798	1.9701	11.4301	4.322	2.412	2.8377	0.4288
5.9949	25.3104	8.8818	10.9702	11.3448	8.5195	7.1326	6.7656
1.9405	2.3231	2.2496	5.2185	8.2441	0.9073	4.3191	2.9913
3.4537	0.281	0.4637	0.6711	0.3796	0.0026	0.0694	0.031
1.205	3.3207	2.524	9.5537	2.0769	2.0534	1.1777	1.383
0.5299	1.7439	0.7222	2.9282	1.6783	0.9655	1.127	1.0622
1.9723	1.8747	1.8494	5.6724	7.0875	6.1444	6.0705	3.5069
5.4867	5.6844	5.0429	12.8584	3.2719	3.2262	0.9595	9.3615
0.708	1.7527	1.0201	7.3439	4.9915	1.9289	1.3901	0.7472
1.9823	1.9598	0.7393	4.0316	2.762	1.3654	0.7673	0.4037
1.7133	4.4179	3.0374	0.1666	0.521	2.9769	0.8902	0.654
0	0.0095	0.014	7.5515	0.3793	0.0061	0.0488	0.0066
1.3805	1.4817	1.3271	1.178	1.685	0.4014	0.2161	0.2651
0.375	0.4246	2.4405	1.2335	0.3562	0.9578	1.0888	0.6592
0.5686	1.0038	0.2042	1.7376	0.5838	1.6123	0.658	0.2889
1.6185	1.8142	1.9402	3.6756	2.1193	2.9882	0.1004	1.4691
2.5191	4.3071	1.356	3.7183	0.2616	0.0345	1.5045	0.1112

3.1028	18.1624	0.7552	0.0907	1.7423	1.7226	2.7449	1.5195
0.7803	0.5146	1.0971	7.5008	3.4521	1.0757	0.5668	0.426
0.6359	0.7678	0.9837	1.4417	0.2444	1.6454	0.5781	0.9401
0.6713	0.5133	0.6284	0.9626	0.5576	0.2757	0.5103	0.4149
0.5194	0.5476	0.3448	1.398	0.204	0.4033	0.4666	0.3794
6.3779	8.0474	12.1934	18.4556	19.8637	5.8122	4.6602	6.6305
0.3738	0.0929	0.091	0.2255	0.0606	0.2594	0.6069	0.472
0.6916	0.2083	0.3826	0.8732	2.1499	0.4251	0.5325	0.1684
0.6978	0.8918	0.7765	2.0769	3.3587	0.2129	0.4222	1.6934
0.86	1.4965	0.7109	0.7295	0.0058	1.386	0.2673	0.0062
0.3396	0.3138	0.3815	4.1465	0.7546	0.3556	0.4979	0.2474
6.1614	3.2893	2.9917	4.0605	4.237	3.247	0.9343	0.5607
1.531	6.0127	2.773	10.0429	8.5677	3.5672	2.2122	1.9536
0.5486	1.9995	1.0015	0.5815	0.5727	0.6834	0.4905	0.5247
1.6072	0.9026	4.8401	6.3023	9.3245	6.8344	10.1136	0.7816
1.3657	0.5612	0.219	0.1161	1.006	0.4069	0.4633	0.1944
0.5547	0.2647	1.3968	0.6297	0.638	0.7362	0.5192	0.3607
0.5374	0.8055	0.436	1.459	0.5803	0.601	0.289	0.558
1.3409	2.4828	1.8559	0.2883	2.0442	1.6281	0.3712	0.513
1.8956	1.3769	1.1446	1.2151	1.9131	0.467	0.6484	1.1715
2.8253	4.3011	2.5556	0.8373	3.5868	1.9973	0.8085	1.0517
3.9581	6.1271	3.8199	8.1292	7.5651	3.0596	2.7938	5.7618
0.3753	2.3316	7.5745	14.6091	26.0049	0.5343	17.5723	1.723
0.8865	0.1069	0.199	16.3212	2.3606	4.337	2.7154	0.1729
0.1481	0.2886	0.8057	17.1544	0.4609	0.3906	0.5288	0.3399
1.365	1.0418	1.4652	2.9934	3.4117	2.1614	0.7578	0.7833
1.9004	4.0525	2.6331	1.9983	3.4308	1.311	0.7154	0.9284
0.3356	0.6641	0.7856	1.5306	0.7751	0.4479	0.3718	0.3236
1.9874	2.3467	2.007	2.5501	1.9634	1.5801	0.7163	1.5288
3.5433	4.1281	2.8871	2.8966	6.0031	2.3681	1.5242	2.1818
7.0805	8.1523	11.0943	13.2759	8.3911	6.8979	11.2948	7.5171
1.3063	1.5899	0.9246	1.5541	1.8136	0.6356	1.3673	1.2647
0.2616	2.2753	1.5919	0.1434	0	0.8379	1.4311	0.2627
1.9576	0.1138	1.2121	1.1297	0.0371	1.1367	0.4848	0.1971
0.067	0.9052	0.1945	0.9721	0.3006	1.8106	0.6403	0.3313
0.5557	0.6444	0.6087	1.6045	1.4602	0.356	0.2092	0.1063
1.2411	0.3069	0.8566	0.9749	0.6001	0.5735	0.1307	0.2976
1.4039	2.1007	1.0471	1.8869	1.8582	1.2571	0.5754	0.6237
0.6798	0.7287	1.2896	1.7539	2.7635	1.3021	0.5081	0.1377
0.3045	1.1531	0.5294	1.3833	0.6107	0.1858	0.6141	0.4494
4.5022	0.4778	1.0334	3.9703	2.1799	1.3684	0.5202	0.3862
1.1585	2.2519	1.4379	1.4024	2.0685	0.4409	1.1821	0.6714
0.6212	1.0862	1.3859	1.6271	1.1179	0.7369	0.682	0.6733
1.4768	1.1021	1.1982	3.049	1.2786	1.4489	0.9767	0.4734
0.3206	0.4131	0.5202	1.4061	4.8851	0.7987	1.0057	1.0221
0.465	0.9629	0.7427	2.0074	0.7845	0.4033	0.5743	1.1674
2.9647	1.7126	1.1869	2.0531	1.8113	0.9371	0.826	0.8283
0.716	1.4234	0.61	2.8263	2.4354	1.9302	0.9349	0.5342
1.1059	2.614	1.6116	1.8768	9.4698	1.1356	1.0839	0.5093
0.1454	0.8915	1.7878	3.7638	1.6648	0.4969	0.5749	0.1335
2.1888	0.9598	2.037	3.1764	1.2513	1.4433	0.727	0.665

1.8078	2.4433	0.2461	0.6781	0.3853	1.5111	1.2257	0.9283
2.1502	2.2488	1.8672	3.5459	3.8985	2.6366	0.6844	2.2912
0.225	0.2175	0.8901	0.7505	0.486	0.3103	1.1913	0.2906
0.9049	2.1862	0.8948	1.3849	1.0687	0.473	0.5855	0.3786
0.523	0.9349	0.2094	0.8074	0.052	0.2719	0.068	0.1027
0.7902	3.3274	2.5645	2.3106	2.8443	1.5116	1.6733	1.0203
0.7178	1.6987	0.7487	1.5741	0.6644	0	0.2895	0.2354
1.1849	1.6987	1.5807	2.8983	2.0669	2.0435	0.9972	0.6538
0.7017	1.889	2.7042	1.6671	0.442	0.3122	1.7335	0.4698
0.8075	2.5269	0.7643	1.9676	1.4533	0.9579	2.1532	1.1328
0.3272	1.1502	2.0425	2.1661	1.0707	0.9412	1.2869	0.708
0.0899	0.0447	6.9839	1.7457	2.2922	0.0192	2.2221	2.6325
2.1197	1.4093	0.5177	0.6296	0.8956	0.9234	1.261	0.716
4.2849	4.3152	2.6894	5.1421	3.4907	4.9256	1.4611	1.4216
1.477	1.9396	0.8439	2.8341	0.6127	1.25	0.4196	0.6099
0.2826	0.4682	0.5159	1.3944	1.5563	0.3922	0.8378	0.746
0.3895	0.8115	0.5871	1.6064	1.7589	0.712	0.4707	0.3065
3.3576	1.1388	1.6731	1.3625	3.5114	0.9032	0.6718	0.5462
0.2809	0.7238	0.3038	1.8821	2.7633	0.7996	0.793	0.4656
1.1887	0.8836	0.7048	3.8433	2.995	0.6345	0.8175	0.1049
0.3155	0.5227	0.32	0	0.0568	0.5614	0.2969	0.0604
3.3501	1.5551	1.2896	2.9414	0.8965	1.1892	0.4182	0.8284
2.1646	1.4975	0.6224	2.217	1.0788	1.1428	0.4533	0.6962
0.1486	0.6401	0.6873	4.8233	0.9949	0.3808	0.4615	0.1706
0.9428	0.9229	0.9387	2.9602	1.2494	0.2745	1.2704	0.5902
0.5119	0.8128	1.272	8.6539	4.4916	1.5714	0.6324	0.0979
2.0254	4.4615	2.4378	3.1238	2.4755	1.1881	1.9794	0.9707
1.7407	1.9937	1.9877	3.8881	2.9704	1.8814	1.6686	0.962
0.3089	1.0438	0.6164	3.5898	1.9344	0.6991	0.8371	0.3545
0.0174	20.8744	43.0848	3.1825	1.3137	32.4531	15.9475	8.358
1.1431	1.2332	1.2618	2.536	1.6937	0.965	0.3753	0.5797
10.3777	1.9334	0.4123	28.8123	4.7968	8.3064	3.7024	2.3188
0.5775	0.3417	1.4559	1.8093	5.6572	0.8368	1.3394	0.5682
0.4476	0.8788	1.0491	3.7444	0.6444	2.1415	1.8721	0.4186
5.3842	1.7176	1.9935	14.4373	0.9703	2.2949	1.5124	1.8087
0.7968	0.9068	1.2395	1.6637	2.2893	0.2071	1.115	0.4533
0.6771	2.3134	0.9141	0.3248	0.3656	1.1859	0.463	0.7407
0.2957	0.8462	0.8834	1.975	0.8709	1.062	1.7457	0.5245
0.2419	0.668	0.3599	1.7981	1.2192	0.8323	0.759	0.3703
1.5415	0.7449	0.9771	1.7959	0.6242	1.7487	0.8161	0.2949
1.2062	2.9482	0.7701	3.469	1.7936	1.1189	0.8374	0.9305
2.0491	1.5575	2.2297	2.4847	2.2647	2.059	2.2631	0.6225
0.5406	7.5082	11.4512	20.4076	3.9872	5.4282	7.6483	0.4357
0.7705	1.4378	0.3126	2.5733	1.2145	0.5862	0.3376	0.6302
1.8147	4.6169	3.6282	5.0101	4.374	2.3872	3.3395	9.5587
0.3201	2.4476	0.1798	0.189	0.1064	2.9181	1.5295	1.9783
1.7476	11.2363	0.6836	0.0456	1.3929	1.466	1.4392	1.4089
0.6119	1.401	1.481	7.9383	3.2147	0.1174	9.599	0.9706
0.4086	0.9419	0.1946	1.4221	0.518	0.4173	0.8528	0.2855
0.3376	0.6008	0.8675	3.4896	3.3353	0.534	0.4237	0.3947
3.7218	4.6873	4.2984	8.495	8.489	4.9264	5.2272	3.073

0.4124	0.7686	0.941	1.8369	1.9203	3.0268	0.4002	0.7987
3.2858	2.0083	1.7083	7.4856	0.2411	0.4768	0.2252	0.1465
0.66	2.7336	0.241	2.7501	1.8887	2.6778	1.1181	0.8333
2.1133	5.0235	3.4152	3.2225	1.6876	2.6445	2.0259	1.6076
1.3159	1.308	1.0628	2.2693	0.9301	1.0345	1.0977	0.357
1.3867	2.2974	1.2994	1.3992	1.6943	0.9453	1.0661	1.1433
4.8666	42.7113	15.3513	29.7148	23.6215	10.0961	25.3664	14.0855
2.5228	1.1325	2.1393	1.6003	0.4335	0.8167	0.4825	0.7099
3.4316	7.5323	6.0662	3.3681	8.556	5.4385	3.6448	4.5212
6.6098	4.2774	2.3881	6.4549	4.21	3.4457	2.515	1.6003
0.7307	1.2653	2.1172	1.1476	1.4509	1.0218	1.1381	0.8658
1.9244	0.3168	0.32	8.5585	5.4469	3.8667	5.7709	0.9878
0.4017	1.1587	0.0927	1.4351	0.0673	0.7024	0.088	0.0079
1.3609	0.2948	6.1005	1.278	0.5173	0.7515	0.8822	0.923
1.5282	1.7645	2.5361	6.2455	3.9504	0.9394	2.811	1.5414
0.3883	1.5144	3.1341	0.1347	0.3078	0.2728	0.4648	0.4061
0.3874	0.0458	3.7889	2.0254	1.0534	1.2778	1.9631	0.5955
0.542	1.0316	0.4129	1.7422	1.5347	1.7626	0.4322	0.3609
7.76	11.2313	12.017	22.6285	5.9393	10.305	6.9859	1.7409
2.2371	0.4704	0.4633	0.6842	0.0943	4.6403	0.7135	0.53
0.3255	0.0529	2.1673	2.2747	7.1891	13.2004	4.8299	0.033
0.6506	0.6859	0.9226	0.1769	0.4761	1.2227	0.5996	0.5616
0.0269	0.0446	2.0323	15.7703	1.3088	0.0575	3.1938	0
0.0757	0.0228	0.3683	9.0432	0.2451	0.022	0.0874	0.0237
0.2598	0.7172	0.4004	1.4051	1.3276	0.6841	0.6356	0.2584
2.0014	2.7039	0.7394	4.7418	2.5472	1.9715	1.7322	0.8614
0.0557	5.046	1.5641	31.0636	4.2281	0.0686	0.2359	0.0197
0.032	0.0265	0.039	3.6365	0.1903	0.9066	0.0905	0.0368
0.9045	1.4342	1.3209	3.8821	2.7069	1.1588	0.7479	0.9788
3.0069	0.8549	0.3825	2.7695	2.4048	3.9932	0.8654	0.3295
0.9245	2.254	1.5921	4.5711	0.3579	1.3595	1.0096	1.3614
2.797	1.5831	2.0319	1.4246	0.9623	1.2266	0.5608	0.4806
1.8446	2.7164	0.1559	1.0019	0.4611	2.5711	0.8801	0.2646
0.6566	0.1483	0.1453	0.9818	4.8986	0.5417	0.5477	0.5138
0.3049	0.5246	1.1704	0.0386	0.2913	1.0143	0.6623	1.4404
0.1059	0.5265	0.4835	2.2653	2.0021	0.4807	1.0095	0.2736
1.8677	1.1067	1.5098	1.6294	0.6036	1.2809	0.2502	0.9858
0.104	1.841	1.5185	0.0784	0.6456	0.9852	1.7247	0.2536
3.3523	7.2348	7.66	3.3007	1.6868	0.9008	1.5453	1.6359
2.4536	0	0.5312	1.1641	0.3567	0.9949	0.8493	0.6092
1.6019	0.1531	0.0375	0.5573	0.3493	2.0885	0.1957	4.0484
0.9026	0.3916	0.4185	5.9617	1.578	1.9502	0.5461	0.222
0.7374	0.8398	1.6545	0.6317	2.4136	0.5658	0.4229	0.6612

TCGA-HL-75	TCGA-CR-73	TCGA-CR-73	TCGA-CV-74	TCGA-CR-73	TCGA-CV-74	TCGA-CV-74	TCGA-CV-70
4.0427	0.7324	1.46	5.0713	1.311	2.9672	4.511	6.3799
2.1717	2.0301	3.8837	6.1709	1.6138	11.5021	1.5736	3.3447
1.2482	0.8029	1.2386	1.3304	0.7311	1.6161	0.7883	1.2331
2.0792	0.7214	1.6129	0.7978	1.0578	1.2148	1.1266	0.3856
1.3483	0.5257	0.5741	3.1039	0.8273	3.6255	0.635	0.4861
1.5209	0.8923	1.4843	1.2392	0.6112	0.5703	1.0024	0.7825
0.5808	0.3183	0.6518	0.8532	0.5911	0.6717	0.6008	0.5046
0.8275	0.5432	0.7317	1.1527	0.7058	1.9594	0.6034	0.4102
0.311	0.6585	0.375	0.1874	0.1311	0.63	0.4282	0.4324
0.6448	0.4056	0.3232	1.0552	0.3716	1.1676	0.341	0.6987
0.0106	1.6019	2.3461	3.6649	0.5923	1.4198	1.6827	1.0999
0.2018	0.3951	0.5023	1.2131	0.3592	0.2549	0.3948	0.6742
3.8262	0.4983	0.3967	0.7232	0.5719	0.5294	0.5101	0.3795
3.3521	0.0787	2.6986	0.3163	1.3176	0.0615	0.7928	0.1766
1.1105	0.7797	1.2562	0.9151	0.91	1.1083	0.9731	0.8273
1.711	1.1743	1.3038	2.3436	2.2363	4.768	1.6737	1.6596
3.3324	1.699	2.0706	12.5632	2.8799	7.9186	2.3232	4.133
2.7246	0.5582	0.3173	1.9636	0.2805	0.1247	2.8112	0.2637
0.6445	0.601	0.8205	1.3376	1.3809	1.1487	0.2985	1.2301
1.0521	0.7064	0.6807	1.9037	1.0314	1.633	0.9122	0.652
17.9	7.5825	9.4084	4.0776	4.4242	8.8315	11.0989	5.4531
6.6047	7.6715	3.7407	7.9021	2.4192	2.1778	1.825	3.3299
2.6092	1.2144	1.7387	0.9838	1.1079	3.0213	1.3501	0.8586
1.321	0.9116	1.4537	1.139	2.0656	2.9408	0.7892	0.5848
1.6976	1.4239	0.6678	2.123	2.0973	1.6462	1.1041	0.619
1.977	1.6105	1.0782	2.3079	0.7113	2.7151	2.7989	1.7389
0.7649	0.3456	0.8132	0.8342	0.6151	0.7661	0.3209	0.598
12.9077	8.6585	2.9254	7.3478	7.2244	4.6425	5.8208	3.953
0.2181	1.0063	0.49	0.6716	0.8962	0.4116	0.7706	0.322
0.8228	1.0606	2.1056	2.8583	2.7167	4.2053	1.4985	3.0362
1.5254	0.3355	0.2263	0.5626	0.3783	1.4733	0.5137	0.4511
0	0	0	0.3665	0.0295	0.1545	0.019	0.0231
1.059	0	0.0224	0.0242	0.0857	1.4841	0.3324	0.2014
1.2397	0.4736	0.6974	1.2258	1.0752	2.46	0.5965	1.0973
0.7706	0.7917	0.6532	0.4564	1.3285	2.3564	1.0128	1.7733
1.3413	1.5789	1.5038	1.312	1.0327	2.2653	1.1381	1.7771
4.6269	4.097	5.7329	2.9568	2.1995	4.7524	2.4086	5.8786
1.1492	1.1062	0.5863	0.6059	1.0201	0.0535	0.3297	0.1598
1.778	0.4328	0.5599	1.8896	1.0975	0.9714	0.5231	0.5297
1.8036	1.8383	0.7538	2.2727	0.8584	1.1961	1.9816	0.7798
3.1015	0.2266	0.6149	1.1602	0.8468	0.8223	0.2595	0.7538
0.9297	0.7309	0.6443	1.6159	0.4021	2.7971	1.0396	0.48
0.9449	0.5639	0.2426	1.0295	0.5014	0.7114	0.4321	0.7856
0.2524	0.039	0.1502	0.5961	0.1437	0.7038	0.1471	0.2721
4.8102	4.0233	3.7956	6.9513	4.1613	7.8806	3.3919	4.0147
3.1747	1.2555	0	4.6735	0	0	0	0.7008
0.9693	0.3586	0.223	0.9293	0.2987	0.2463	0.3517	0.351
0.2164	0.2511	0.3112	0.5988	0.2647	1.1631	0.1283	0.2073
0.0086	0.573	0	0	0	3.2992	0.9786	0.7767
25.5298	18.909	11.2169	10.181	18.5386	14.1865	15.0006	9.2757

0.1944	0.2901	1.0308	0.3405	0.0502	0.2983	0.1081	0.5763
1.9519	0.458	0.3565	0.6431	0.85	0.5852	0.448	0.3308
2.3323	1.6631	3.6862	2.2789	3.1764	4.7728	1.9331	2.4767
2.9839	3.6548	2.0514	1.6732	1.972	0.936	1.9409	1.9099
3.065	0.6725	0.7345	2.2684	0.4548	0.7159	1.2025	1.0364
1.7342	0.2724	0.7611	0.2983	0.1884	0.224	0.4545	0.7673
0.3063	0.3115	0.4403	0.39	0.3064	0.1909	0.0743	0.3001
0.5116	0.1527	0.4597	0.8958	0.1408	2.733	0.3412	1.4336
1.9983	0.4409	0.6634	0.6321	0.325	5.2147	0.969	0.4745
0.3173	1.1706	0.3484	1.2033	0.4961	0.0916	0.3899	0.8145
0.0548	0.6733	0.8823	0.302	0.2321	4.2931	0.1425	0.2091
4.8696	1.7244	1.1305	2.7796	1.8835	0.6976	2.4096	0.7862
1.7787	0.0804	0.3487	0.9787	0.1112	1.8418	0.0959	0.3679
0.6937	0.414	0.5984	2.3213	0.334	0.7512	0.3701	1.0467
1.3113	0.8791	0.7722	3.5732	2.2613	2.8296	0.6166	1.3126
3.242	1.5094	5.0909	6.7997	1.3603	5.7599	3.7419	5.9474
9.5459	0	0.0811	0.0146	0	0	0.0167	0
0.0175	0.0261	0	0	0	0.0252	0	0
0.6642	0.2241	0.3126	0.7323	0.6527	0.9647	0.5344	0.3324
0.0483	0.144	0.0694	0	0.0664	0.2091	0	0
1.1607	0.5519	0.7336	2.2202	1.1816	1.7833	0.4537	1.668
0.3327	0.3723	0	3.2369	0.515	3.2429	0.7398	0.8069
0.8465	0.3943	1.0142	0.4671	0.419	1.0961	0.5696	0.3138
1.6371	0.8778	1.7285	1.712	1.179	0.831	1.0236	0.9373
0.6737	0.1256	0.8021	0.6718	0.3186	0.6383	0.2434	0.658
1.7005	0.5382	1.8707	0.3058	0.3279	0.2651	0.5843	0.5345
0.7374	0.2358	0.1136	0.3484	0.1993	0.6464	1.0305	0.369
0.4286	0.1865	0.0128	2.7793	0.4054	5.0147	0.0079	0.7217
1.64	0.4547	0.5425	2.2251	1.857	0.681	1.0325	2.4245
1.3665	0.1492	0.0958	0.7781	0.1376	0.2165	0.2371	0.8262
0.0191	0	0.0137	0.0891	0	0.0689	0	0
1.2865	0.4005	0.4558	0.4718	1.649	0.2539	0.1934	0.1895
1.2145	0.5812	1.5814	4.5119	1.1981	9.0492	0.6317	1.6547
1.1421	1.0606	0.6217	1.9916	0.4059	0.2173	1.2686	0.6385
1.4372	1.7869	1.3776	3.2757	1.7655	1.5564	1.7955	1.4384
2.6418	0.8491	1.374	3.7153	0.7538	2.8516	2.3267	2.3346
0.068	2.6372	1.9549	1.164	0.5144	0.2454	23.579	1.2456
0	0	0	0.1093	0	0	0	0
0.7474	0.1593	0.4861	0.7201	0.1469	0.8735	0.3798	0.2301
1.9483	0.539	1.0945	0.8736	0.4083	1.7139	0.872	0.9595
0.5448	0.3902	0.5483	1.5773	0.3898	2.1869	0.0388	2.2078
0.8215	0.6314	0.9127	0.5812	0.2911	1.276	0.7306	0.5634
0.8444	0.9203	1.4767	2.5121	0.9764	1.9758	1.177	1.704
1.6606	1.4103	1.506	1.7099	0.7908	1.3464	1.3859	0.4681
0.3763	0.7964	0.6196	0.2213	0.1415	1.0992	0.6926	0.8299
3.4737	5.3241	8.2408	6.6602	6.213	7.7514	3.9036	5.1053
1.371	0.4546	0.7301	0.9879	0.5589	2.2361	0.587	0.6567
1.5827	0.2133	1.0205	1.7343	0.1886	1.604	0.0523	1.2798
5.2159	5.7047	5.0007	3.8894	1.4724	2.7979	8.2144	2.4244
2.9789	1.1111	3.1792	1.9211	0.3857	2.6701	2.8278	1.2265
3.9444	1.1062	1.4498	3.162	1.2445	5.3526	0.989	1.758

0.7817	0.3628	0.6993	2.1225	0.7529	2.7841	0.2317	1.0297
3.8024	0.9815	3.5973	2.0922	1.418	4.4263	3.2527	2.0608
0.8816	0.4921	0.7847	0.5648	0.4992	0.853	1.3334	0.7563
0.4517	0.674	0.2923	2.2851	0.2486	0.1304	0.2009	0.4382
2.7054	1.4492	1.6168	3.2673	1.3768	1.8025	1.4899	2.0095
0.6811	0.3233	1.0015	1.1565	0.2343	0.2011	0.6883	0.6673
28.5103	19.5911	6.5077	11.857	5.2709	4.7658	16.7963	5.3657
0.0367	0.0547	0.1845	0.685	0.0505	0.9002	0	0.3557
0.0862	0.0429	0.0207	0.0224	0.0593	1.9919	0	0.031
0.7392	0.4273	0.8735	1.3161	0.9394	1.8234	0.8452	0.0717
0.9445	1.8788	5.5448	0.8575	2.8515	1.8561	2.3798	1.3007
9.7686	8.795	0.4843	0.6554	5.2717	2.4926	3.7445	0.1815
0.7447	0.7071	0.438	0.382	0.2794	0.6354	0.5569	0.6202
0	0.2887	0.0696	0.3012	0.1997	9.6393	0.086	0
0.9428	0.9101	0.8372	0.7337	0.267	2.5623	1.0357	0.2391
5.4291	2.0769	0.2502	0.5959	4.1657	0.2512	0.7428	0.3001
0.1946	0.0726	0.4313	0.7319	0.1896	1.0653	0.3461	0.5592
1.6231	0.7301	0.3577	1.9804	0.7687	0.5417	0.3119	0.6681
0.2169	0	0.2599	0.3939	0.1492	1.2007	0.0643	0.3117
0.5385	0.2678	0.3871	1.9325	0.5763	1.1231	0.0532	0.1935
0.7259	0.1217	0.2463	0.0762	0.2749	0.1119	0.0218	0.4219
0.8364	0.5224	1.4683	0.3482	0.736	0.014	0.2595	0.1887
0.1713	0.1785	0.1896	0.6432	4.006	2.63	0.2853	0.2901
0.7426	0.8502	1.3035	1.4783	0.3207	2.057	1.4743	0.912
1.1463	0.2911	0.789	0.5315	0.5872	0.5106	0.5639	0.4732
0.112	0.1114	0.4295	0.2034	0.4624	0.647	0.1328	1.3281
8.3078	0.3974	1.0846	0.23	0.0945	0.0015	1.0049	0.0045
0.8093	0.46	0.2216	0.2399	0.3711	0.1669	0.1371	0.1661
0	2.2176	0.8449	6.3487	0.4756	0.2246	0.5533	1.1176
3.3086	1.4934	1.2392	1.4497	0.9755	2.4689	3.0165	0.5993
1.7786	0.8939	0.9018	0.4663	0.4637	0.7839	1.1655	1.9371
4.0847	2.6961	1.6373	2.2166	0.7404	1.7961	3.4945	0.7445
0.7365	0.1668	1.0229	0.3541	0.3767	0.1002	0.3943	0.5444
6.9976	1.7547	1.0145	2.6085	2.1844	3.099	1.8303	1.6478
0.6832	0.3763	0.476	0.499	0.188	0.22	0.4113	0.9402
0	1.3957	1.2399	0.3185	0.2614	0.0211	0.26	0.0945
0.7075	0.3248	0.7042	0.593	0.2246	0.1179	0.3388	0.3519
0.3534	0.8437	0.7114	1.8704	0.7537	1.2758	0.5343	0.5714
2.9224	0.5039	0.2081	0.9709	0.0047	0.403	2.4089	3.2611
5.8397	3.8018	3.484	9.8644	1.8376	0.5593	5.2084	1.7185
0.6797	0.4919	0.2662	0.5448	0.5096	0.7345	0.4343	0.25
0.7547	0.249	0.4121	1.1464	0.2496	0.1205	0.3485	0.1877
35.2163	10.7238	15.2888	16.7429	12.587	14.098	15.5275	15.2456
1.4507	1.1551	0.7671	1.5144	0.5086	0.6143	0.9614	0.654
0.498	0.401	0.6195	0.5414	0.2474	0.8019	0.4781	0.2386
1.2243	0.5871	0.8906	0.9301	0.2807	0.1473	0.4925	0.3613
8.1995	2.1442	3.581	4.5835	0.7824	9.0447	6.1941	3.2213
0.4405	0.8697	2.2327	2.2751	0.8846	0.8127	1.0011	1.4195
0.724	1.1695	0.9119	0.014	0.4569	1.8877	0.3299	1.3607
1.0309	0.284	0.4569	0.8114	0.9691	1.1375	0.3777	0.4956
0.8793	0.9541	1.178	1.9285	0.1925	0.5482	0.8886	1.0338

1.9658	1.4756	0.8251	3.3352	2.8557	3.1556	2.0522	1.2501
1.2887	1.1944	0.8703	1.5652	1.1015	2.3541	1.2676	0.7155
0.8858	0.5855	0.0161	1.0646	0.27	2.5013	0.7379	1.281
1.7815	0.9339	1.2461	1.3115	0.1987	1.0428	0.5995	0.7784
2.1631	0.0088	0.0043	3.1777	0.4729	9.006	2.867	1.0922
0.3811	0.398	0.767	0.7118	0.5505	0.5502	0.305	0.2464
1.7678	0.3197	0.3466	1.1673	0.5896	0.2707	0.3811	1.2124
0.2593	0	0.0666	0.4468	0.1083	0.1003	0.2388	0.1896
0.295	0.3276	0.6757	1.5779	0.2997	1.6073	0.3905	0.8319
0.3881	0.313	2.247	2.7264	1.9842	12.0624	0.3731	0.2487
2.3643	2.1547	1.2433	1.269	1.8027	0.9922	1.0927	1.3808
6.8739	2.0623	3.0646	2.2275	0.8196	4.2704	1.1961	2.2822
3.0618	0.3926	0.4987	2.541	0.3209	0.7944	0.1383	0.4125
3.1848	8.2069	8.0336	9.0143	6.5823	4.8596	20.5888	7.6064
0.2286	0.2558	0.9862	0.4004	0.3539	0.3301	0.5592	0.5545
0.3571	0.1066	0.4878	0.1946	0.0737	0.7993	2.0644	0.1155
0.524	0.288	0.0991	2.0604	0.1518	1.0552	0.0981	0.8025
0.5469	1.9719	0.9782	2.6298	1.4467	0.3525	2.2813	2.6104
0.9362	0.2993	1.0576	0.687	0.3496	3.1864	0.3687	0.5334
1.0841	0.8879	1.1578	1.2275	1.5317	2.2023	0.9734	1.2133
0.4012	0.5986	0.1442	0	1.5181	0	0	0
2.6342	2.5882	1.8706	1.6001	0.5967	0.6494	2.4569	1.1772
0.2501	0.3731	0.9288	1.8164	0.2007	2.4371	0.7783	0.8984
0.4963	0.1307	0.4197	0.6134	0.1607	1.2012	0.3375	0
0.0111	0.3916	0.9951	0.1211	0.0044	0.0113	0.0278	0.2968
0.0816	0.0812	1.1542	0.4447	1.6286	1.3555	0.1936	0.3813
2.2557	0.8346	0.5319	1.5588	1.2414	5.5758	1.6369	1.7504
2.5903	3.8647	3.836	2.7618	2.3699	7.0126	3.248	4.3551
0.2856	1.2274	2.727	0.4001	0.6052	0.5032	1.4733	0.4803
7.667	4.1646	3.0707	5.9809	4.0897	7.4935	3.7557	5.0205
0.5418	0.8695	0.8792	2.2742	1.5133	1.5881	1.438	0.575
1.4477	0.5254	2.2221	1.1571	0.4037	1.1016	1.357	0.7169
3.0695	0.213	0.2737	0.9444	0.2292	5.205	0.1693	0.2821
0.4046	0.8625	0.8727	2.2494	0.7556	0.9598	1.131	0.3115
1.279	0.4337	0.9612	0.9953	0.4532	1.1751	1.0857	0.4386
0.2397	0.3251	0.4543	0.7802	0.2549	1.3688	0.155	0.2584
0.377	1.1562	0.3112	1.0052	4.1545	0.1915	0.7761	0.4891
4.7166	1.6623	0.8009	3.584	1.8395	9.6254	1.6183	0.8806
0.4695	0.1691	0.3608	0.2646	0.1893	0.7714	0.2016	0.1919
1.3331	0.8407	0.6422	1.3583	0.2174	2.282	0.3789	0.3555
0.2661	0.2206	0.3401	0.6444	0.2441	0.4483	0.3156	0.0319
0.4401	1.0669	3.2031	4.3667	0.1514	0.8737	0.2935	2.0751
11.4946	2.9454	2.5123	5.6361	1.5815	1.321	9.3008	2.561
1.3797	1.1102	0.1114	0.1448	2.7301	0.3693	0.4412	0.0334
3.1766	1.2124	2.4252	4.4653	2.3377	13.1555	4.4455	3.3706
0.94	1.0685	1.2764	0.7838	0.7647	1.7612	1.0151	1.0131
6.0806	2.617	3.5726	3.0031	0.8044	14.2667	6.3434	3.0877
1.4203	0.7185	0.6003	0.9773	0.608	1.6679	0.4716	0.6504
1.3336	0.2744	0.4959	0.8231	0.0633	0.1328	0.6952	0.5452
3.3006	1.5	1.5745	2.4246	1.368	2.7955	2.1295	1.277
0	0	0	0.5057	0.0253	1.7261	0	0

2.2683	0.7325	1.2918	2.4454	1.9657	2.552	0.4453	1.9896
0.8948	0.4644	0.3915	0.878	0.4817	4.3814	0.1384	0.5451
6.8091	2.5938	0	3.918	0	16.682	0.6764	3.1618
1.9003	0.6559	0.9685	1.9314	1.834	3.7266	0.5801	1.0546
0.955	0.2035	0.6375	0.6901	0.1408	0.1477	0.6673	0.2205
0.065	0.1212	0.5198	0.6954	0.2962	1.2668	0.4046	0.4378
0.0082	0.1589	0.0177	0.051	0.0507	6.1565	0.0364	0.0177
1.1406	1.6521	0.6926	0.7239	0.3809	1.407	1.8021	1.6112
0	0.3736	0.5144	0.2227	0.0246	0	0.6363	0.1928
1.104	0	0	1.4115	0	4.2982	0	0
1.52	2.6647	2.0215	4.2586	1.2548	5.6651	2.6359	2.9693
3.0127	0.0848	0	0	0.0782	0.041	0.1517	0
0.5748	0.8576	3.113	4.8313	2.4121	17.4014	1.2609	2.9737
0	0	0.1582	0.3425	0.3532	1.5885	0	0.1581
0.3543	0.5948	1.9742	4.0331	0.2438	6.1395	0.1969	0.5251
0.7131	0.1796	1.1893	1.2162	0.1396	0.3229	0.1709	0.2594
1.2234	0.9403	1.599	0.6347	1.8106	0.8297	1.2198	0.5594
3.6896	0.015	0	6.4919	0.4138	0.0434	0.0178	1.2967
0.3545	0.2885	0.834	0.9279	0.1108	0.1629	0.5445	0.9725
0.7041	0.6438	1.5021	0.9014	0.375	1.8692	0.9493	1.126
0.9382	0.4749	0.548	0.9387	0.7203	0.3447	0.2533	0.2528
1.5138	0.6142	1.1265	1.5192	1.1419	2.0036	1.736	0.6584
0.9443	0.322	0.2521	0.7139	0.5197	0.9252	0.6358	0.4362
0.7855	0.4688	0.5929	1.1616	0.2702	1.0492	0.8034	1.3123
1.8745	0.5827	0.8141	0.8814	1.2447	4.3042	0.8219	0.6173
0.3988	0.2518	0.1323	0.5969	0.3482	1.185	1.2414	0.3141
0.4951	0.9233	1.4533	1.3646	1.3197	4.0358	0.6788	1.4007
2.5735	2.8511	1.3254	1.7562	0.7597	2.1838	1.0931	2.2665
15.6767	6.0705	8.3017	11.2224	2.7166	35.4212	16.7615	8.1255
1.4263	0.4138	1.0253	1.7113	0.1772	22.0526	1.6384	1.281
1.0449	0.026	0	1.457	0.7308	0.3395	0.7047	0.2909
1.1473	0.8176	0.9479	1.6125	0.6126	0.7541	2.132	0.406
0.4701	0.4092	2.2061	1.6261	0.2785	10.8325	3.0427	0.6756
2.6572	1.4126	1.6248	0.9745	1.2816	3.3074	2.1186	1.8105
1.5215	1.0972	1.75	2.6247	0.7675	1.611	2.8188	1.6671
22.2355	6.4388	5.4241	9.9201	6.2718	9.1817	5.5408	4.8669
4.9771	14.4597	4.5939	4.7769	1.4445	6.5886	25.3805	5.3097
2.5474	0.0993	0.1486	0.3	0.5857	0.0051	0.1527	0.1888
2.4609	0.8871	0.8192	4.6787	0.6817	7.2614	2.0857	2.3319
0.5337	0.1653	1.1149	0.243	0.3325	0.6034	0.3582	0.5427
2.1662	2.6818	2.3855	7.8372	0.7451	5.6231	17.2555	2.6823
0.5487	2.0377	4.07	1.4899	1.4133	2.2754	2.9482	3.6108
0.5104	0.6231	0.8756	0.4514	0.5666	0.7537	2.022	1.0877
0.5925	1.36	0.7536	1.4188	0.7525	1.4806	0.8917	0.1474
2.1498	1.0099	1.2029	1.8753	2.8646	0.1525	1.1413	1.5583
0.0082	0.0122	0.0236	0.0256	0	0.0119	0	0
0.4288	0.5655	0.7211	1.3795	0.5188	2.3908	0.0409	0.3425
2.3478	0.6713	1.0895	0.5871	0.4096	0.3419	0.5866	0.8605
0.6777	0.2181	0.1528	1.1065	0.2377	0.5084	0.1773	0.2005
1.2914	0.7804	3.196	0.9845	1.601	2.3285	0.9357	0.5331
1.3121	0.103	0.4964	0.9315	0.1108	3.7224	0.0205	0.5954

6.546	1.7597	1.7382	2.9373	5.7201	3.2782	0.3671	0.572
0.6803	0.5478	1.6457	1.4707	1.1143	3.477	0.2785	0.5121
0.5684	0.6876	1.6677	0.7174	0.9617	0.0444	0.6968	0.5961
0.7361	0.2196	0.2117	0.2864	0	0.0531	1.5056	0
1.4809	0.703	0.4355	0.3143	0.1852	0.0486	0.7782	0.1451
9.7725	3.6028	3.687	5.7261	4.018	6.4219	5.442	2.7582
0.0533	0.0795	0.8236	0.5806	0.2933	0.1731	0.782	0.4595
0.717	0.4011	1.0738	1.1159	0.1439	0.6254	0.7704	0.483
1.4211	0.8481	1.042	1.2165	0.7234	1.1285	1.011	0.8577
0.9175	0.5637	0.327	0.324	0.4243	1.2023	0.3908	0.6066
0.2328	0.3334	0.435	0.7898	0.3843	0.5243	0.3974	0.5017
5.5034	2.5135	2.406	3.8983	1.8388	2.5784	0.8989	2.1062
2.85	0.9768	2.3903	1.109	1.1657	2.5116	1.918	1.0377
0.4953	1.0112	0.4685	1.3998	0.4304	1.5808	0.5564	0.9833
3.4559	5.9672	18.9258	6.4065	2.4308	7.2044	9.7034	2.218
1.0715	0.5854	0.2604	0.5285	0.2699	4.0528	0.3758	0.1952
1.3289	0.4327	0.4402	0.2751	0.5166	0.138	0.3286	0.2304
1.2037	0.2721	0.4196	0.6529	0.2007	0.1053	0.2595	0.2752
0.7496	0.7269	0.8352	0.4958	1.186	0.8929	1.1999	0.3635
1.2783	1.7367	0.4931	0.8978	2.3664	0.9679	1.3401	0.4368
1.3062	1.5428	1.3694	0.593	2.3961	2.3967	1.9844	0.7625
8.343	5.2128	3.289	6.4293	2.3671	7.9175	8.0221	9.7066
1.7387	8.3146	4.8715	3.7472	0.8281	4.4738	35.6034	8.2165
0.6564	0.0275	0.2602	6.3731	0.1224	0.2967	0.0546	3.5766
0.0621	0	0.4285	0.058	0.0427	0.0179	0.011	0.2409
0.47	0.6687	1.0059	0.7997	0.5264	1.4679	0.3694	0.6244
2.0144	1.9484	0.9288	1.6758	1.3953	2.6177	1.6061	1.0181
0.9302	0.3321	0.2508	0.773	0.1691	0.3861	0.7799	0.2327
1.6576	1.3489	0.78	1.7356	1.2439	1.9582	1.3401	2.2737
3.2021	2.5412	1.5227	2.2751	1.5104	3.1612	4.4118	1.5253
13.0704	10.7292	6.7296	17.0645	6.3356	2.8417	19.2304	10.2524
1.7987	1.2095	0.7649	3.1348	0.8801	1.2391	1.3011	1.1195
0.2797	0.0695	1.7425	0.5804	0.0641	0.0337	0.2902	0.7536
0.1469	0.3652	0.5983	0.1143	0.6735	0.1414	0.7401	0.897
0.6172	0.4495	0.5124	0.5432	0.2325	0.191	1.6336	0.2693
0.3169	0.3546	0.4556	1.274	0.3451	1.3534	0.5401	0.1707
0.2376	0.197	0.1898	1.0069	0.6721	1.0865	0.8453	0.4553
1.162	0.3853	0.2784	2.6626	0.1332	2.7496	0.4593	2.1568
0.513	0.3827	0.4098	0.6211	0.4509	0.7201	0.8111	0.3993
0.6821	0.2776	0.3566	0.8204	0.128	1.343	2.1507	0.2673
0.6395	0.2726	0.8373	1.6174	0.4242	1.0717	0.6702	0.3446
0.5723	0.9636	0.8111	0.5281	1.0011	0.4781	0.9524	0.5992
0.5903	0.6605	0.4243	1.1868	0.4399	0.9944	0.9187	0.6362
0.6069	0.4244	0.5181	1.0479	0.574	1.4787	0.6915	0.7563
0.6601	0.5303	0.292	0.5532	0.3842	0.6232	0.587	0.1642
2.9411	0.6798	0.2382	0.5803	0.6839	0.8074	0.3684	0.2232
1.14	0.5393	0.4597	2.1854	0.4973	0.6022	0.544	1.0788
1.4034	0.9137	0.8071	1.0326	1.1409	1.2342	0.7488	0.66
1.0443	0.9438	0.6641	1.0706	0.8427	2.1674	1.1787	0.9307
1.9282	0.6496	0.2534	1.3715	0.0998	0.464	0.1291	0.1564
1.1012	1.0953	0.7916	1.1426	0.2525	2.5174	0.1632	0.7912

2.7974	0.4047	0.3413	1.1875	0.5365	0.7833	0.4825	0.4568
1.3415	1.2858	1.1748	1.9614	1.0682	4.5546	1.2508	1.8489
0.2005	1.7549	0.9705	0.4057	0.1931	0.0868	0.9747	0.3457
1.0212	0.8019	0.3767	1.6103	0.4806	2.5802	0.3943	1.9694
2.8351	0.2488	0.0564	0.4199	0.189	0.5666	0.0349	0.3066
2.159	0.7003	0.8435	1.9724	0.7426	2.9818	1.5026	1.1634
0.7308	0	0.8406	0.6825	0.5028	1.0552	0	0.3151
1.4292	1.4539	1.2376	2.8059	1.0725	2.345	1.3865	1.3653
5.0846	1.3058	0.3595	1.265	0.4587	0.3611	1.2602	0.2246
0.6395	1.1449	0.7355	2.2893	0.6159	0.7387	0.1137	0.9649
1.5237	1.0517	1.0672	1.244	0.3796	0.3418	1.0321	0.3108
7.8982	0.0383	1.0416	2.7241	1.6758	0.1296	3.4891	4.4911
2.9306	0.4222	0.6829	0.9123	1.5155	1.9333	0.3325	1.6773
3.4368	1.4554	1.8147	3.6488	1.4599	2.2615	1.0447	1.4093
1.1426	0.2298	0.6737	0.9092	0.574	2.4838	0.0685	0.5258
1.3696	0.3005	0.2896	2.0064	0.4434	0.9015	0.1433	0.3473
0.6246	0.3546	0.4445	0.5123	0.4716	0.4783	0.346	0.2357
2.3365	0.506	0.4335	0.8212	0.7	1.578	0.5027	0.2437
1.646	0.2655	0.4477	1.1079	0.306	0.4496	0.3561	0.0479
0.6734	0.7779	0.3436	0.2536	0.2092	0.4548	0.8307	0.1405
0.4497	0	0.4311	0.2333	0.3094	0.8117	0.0667	0.4039
1.7722	0.4348	0.5942	1.6013	0.4868	0.8329	0.7827	0.5512
0.746	0.3288	0.5363	0.818	0.5248	1.5422	0.2412	0.5847
0.1695	0.3793	1.066	0.4286	0.1457	0.8564	0.7159	0.0457
0.733	1.3671	1.0978	1.6163	0.3362	1.4112	1.0865	1.4484
1.1859	0.5444	1.4208	0.5443	0.502	2.0415	0.8923	1.9007
2.6016	0.568	0.5246	2.8641	0.9821	10.9711	2.3982	0.9232
0.6433	0.9598	1.1011	1.1443	0.4636	2.2337	0.9807	0.9575
0.4227	0.2102	0.3925	0.5756	0.3634	0.7628	0.3759	0.3416
11.3714	9.6842	6.0731	0.0578	10.9072	7.1183	19.8505	17.9429
0.379	0.735	0.8173	1.0027	0.8342	1.149	0.2359	0.4901
3.7385	0.8807	2.4818	2.7007	0.9844	18.2341	4.2314	4.6269
1.1173	0.7896	0.2536	0.4576	0.2023	0.467	1.4119	0.6338
0.3544	0.6699	1.1721	0.9379	0.2926	0.9894	0.6514	1.2734
1.3793	1.0878	0.6162	2.814	0.9353	8.8412	1.4807	2.7184
0.8889	0.781	0.639	0.3689	0.6794	1.1622	1.1506	0.9154
2.0207	0.72	0.9973	0.3873	0.6743	0.9363	0.456	0.8776
1.5712	0.4574	0.7163	0.9245	0.0527	0.1937	0.6134	0.2478
0.6898	0.2859	0.6887	0.835	0.3163	0.8853	0.1022	0.413
0.5494	0.4781	0.2304	1.1757	0.7243	2.1152	0.9364	0.296
1.212	0.6308	0.4863	1.8207	0.5817	2.5843	0.8773	1.3367
1.2199	0.769	1.5069	3.4097	1.0283	3.1506	1.2377	1.1851
0.6559	0.318	8.6874	8.1542	1.6243	13.8736	4.2579	4.0646
0.4545	0.1695	0.9801	1.0119	0.2084	0.966	0.4266	0.2857
6.8828	3.2392	2.0588	4.5656	2.1608	3.5016	1.7664	4.9785
2.1762	0.9426	0.9588	3.9336	0.6278	0.152	1.748	1.551
6.228	1.062	1.2366	3.162	2.6114	2.6549	0.3165	0.4155
0.3724	0.2047	1.6275	0.7703	0.5461	2.7452	1.194	1.088
0.2786	0.5668	0.3459	0.5519	0.3484	0.8959	0.4279	0.1365
0.6952	0.3058	0.4549	0.8948	0.4598	2.355	0.3963	0.1153
2.1113	4.6443	3.8334	4.171	3.1096	5.8429	5.9698	2.1588

0.845	0.4385	0.9508	0.772	0.2275	1.6975	0.8168	0.3564
1.2278	0.1357	0.3596	0.4601	0.1251	1.7728	0.0809	0.4411
0.1882	0.6317	1.0484	2.5993	0.1942	1.4264	1.3806	0.5577
3.9372	1.6724	1.7627	3.5439	3.4169	5.6544	2.5357	1.9329
1.1937	0.7633	0.3555	0.5309	0.3754	0.7387	0.8644	0.5146
0.8724	1.26	0.5738	1.4662	1.0789	1.0787	0.6437	1.0902
32.0409	11.4924	16.8457	19.284	6.1927	9.6981	18.2807	12.9779
0.4331	0.4269	0.7895	1.2399	0.6171	0.8096	0.4539	0.5585
3.3293	5.785	3.337	1.9398	5.6284	3.5286	17.572	3.9561
1.877	1.9219	3.3337	2.4919	0.7849	5.5798	3.8622	3.927
1.2164	0.752	1.029	0.8577	0.9431	2.0717	1.4523	0.5143
0.5282	2.8472	1.2249	2.4266	0.4806	6.544	9.94	3.9849
0.3258	0.0884	0.1987	0.0691	0.4618	0.8481	0.0088	0.0638
2.7544	1.4343	0.2427	1.9046	0.4343	0.0262	1.5131	0.3536
3.8947	2.2652	2.6574	3.031	0.2271	1.9539	3.17	2.2766
0.3363	0.5958	0.0856	0.9705	1.3494	0.3743	0.5732	0.0453
0.7988	1.1182	1.5739	0.5986	0.5699	0.5269	6.6303	0.7653
0.6842	0.5368	0.4388	0.4751	0.8723	0.5259	0.3934	0.4354
7.5128	6.7937	6.3035	6.0762	3.1174	8.9475	4.7972	4.1662
0.3469	0.2654	1.2565	0.4015	0.2142	0.0642	1.3092	0.8868
0.5141	0.6652	0.0654	0.2054	0.1033	0.693	0.6352	0.2059
18.6164	1.0291	0.3581	0.2744	0.4534	1.1232	2.4809	0.4213
0	0	0.1656	0.5079	0.0264	0	0	0.1655
1.4021	0	0.2256	0.3663	0.0742	3.8083	2.0579	0.0317
0.395	0.4051	0.2662	0.2882	0.1019	0.4455	0.7683	0.2128
1.0359	1.3175	0.94	1.7842	0.4907	2.121	1.5857	0.9151
0	0.2733	0.5751	0.1046	0.1512	4.9685	0.0597	0.2633
0.0457	0.1363	0.0164	2.044	0.0786	28.5407	0.0406	0.0246
0.5342	0.4672	0.9666	0.9031	0.8489	1.7286	2.3751	0.9529
0.9955	0.1073	1.0695	1.868	0.5288	0.6149	0.2853	1.1981
0.6713	0.5194	0.5899	0.7353	1.1289	2.89	3.6927	1.1255
0.9336	0.3768	0.6327	0.7266	0.458	1.0884	0.4968	1.0062
0.5843	0.3088	0.7351	3.0316	0.3768	2.5662	0.2057	0.8266
1.2763	0.6347	0.0612	0.4304	0.5268	0.0921	1.3242	0.2293
0.5517	1.4218	0.1562	1.4833	2.0472	0.0724	0.9664	1.5496
0.7174	0.507	0.19	0.4407	0.2597	0.4906	0.9066	0.2441
0.4858	0.7249	0.5588	1.1645	2.0187	0.4489	0.242	0.2304
0.1019	1.5065	1.272	1.0814	2.4853	0.107	0.346	0.0399
2.3243	1.6248	1.1451	2.4793	1.174	2.0183	1.6623	0.6131
0.8912	0.7275	0.8341	1.2301	0.3354	5.0013	1.2262	0.6705
9.3096	0.5897	0.2683	0.4955	0.6193	0.2061	0.2538	3.8336
0.1838	0.4571	0.2202	1.9311	0.7376	2.9193	0.6266	1.1556
2.5292	1.4212	0.5431	0.5624	0.9038	0.5217	0.4966	0.4602

TCGA-CQ-53	TCGA-CV-71	TCGA-CR-73	TCGA-CQ-53	TCGA-CQ-62	TCGA-CV-74	TCGA-CR-64	TCGA-H7-77
6.3967	1.579	3.0773	4.5609	0.7572	1.5427	1.4409	0.8652
2.9924	2.8796	2.6576	2.7552	1.6075	5.3062	1.1714	1.2822
1.0197	0.5779	0.9966	0.5349	2.1104	0.5235	0.677	0.7545
1.1999	0.2454	0.5086	1.5604	1.0119	1.4717	0.9039	0.459
1.6235	0.1788	2.5567	0.5145	2.9816	0.4069	1.253	0.7736
1.1113	0.6201	1.2819	0.7511	2.2631	1.508	0.5031	0.8141
0.601	0.3008	0.3957	0.4933	2.6569	0.4653	0.5944	0.4603
0.722	0.4725	1.2433	0.731	1.9781	0.6899	0.7357	0.3868
0.2671	0.2546	0.4354	0.3515	0.6436	0.3654	0.2897	0.1443
0.1698	0.2211	0.1455	0.2799	0.8439	0.4317	0.2092	0.3309
1.325	1.2236	1.1181	2.0335	1.1691	2.4179	1.9548	0.5504
0.4687	0.24	0.4274	1.4567	0.551	0.5451	0.2089	0.1288
0.362	0.125	0.4818	0.4797	1.0397	0.3556	0.9875	0.2793
0.0462	1.1445	2.1413	0.2839	3.7007	0.0764	0.0394	0.0413
1.2954	0.4293	2.5493	0.9301	1.4594	0.3966	1.2162	0.4463
0.9306	0.5893	0.9712	0.8495	2.6136	0.7202	0.8581	0.443
2.5237	1.9579	2.8795	4.7194	3.7986	9.0412	2.0105	2.3724
0.9947	0.1696	0.5486	0.5113	0.9049	0.8931	0.4119	0.3036
0.8787	0.4381	1.1825	0.6814	1.2153	0.7826	0.954	0.2173
1.6839	0.6913	1.6686	0.2112	1.6875	0.3832	0.8394	0.8701
6.3192	6.3386	6.6637	4.6957	12.7781	5.9704	6.7325	8.5709
5.5632	0.7122	3.9668	5.1031	6.8734	17.0517	2.6685	0.9259
1.4848	0.6592	1.9515	1.3487	1.6489	1.9344	0.9694	0.9008
0.8349	0.476	1.6273	1.5402	2.4456	0.504	0.9529	0.8317
1.4115	0.199	0.6697	0.8756	0.8321	1.5176	0.9819	0.6256
3.2637	0.3913	1.8495	4.2972	2.6728	3.2558	2.5643	0.6047
0.3632	0.4784	2.0548	0.3893	0.6624	1.0072	0.4298	0.2673
26.1051	1.9708	3.9792	10.0605	3.5872	9.3501	5.8905	7.3674
0.5776	0.1483	1.3566	0.804	0.4853	0.2178	0.3864	0.1066
2.0845	0.6145	2.6372	1.0188	2.4029	1.1988	0.6402	0.6769
0.5311	0.301	0.3494	0.4723	2.8558	0.6524	0.3759	0.3159
0	0	0.0179	0.0156	27.0404	0.0347	0.0163	0
0	0.3105	0.884	0.8414	2.3313	0	0.6154	2.1279
0.6039	0.1411	0.8848	0.5203	8.009	0.6011	0.4005	0.5728
0.7784	0.8455	1.1792	1.456	4.3232	0.3884	0.5726	0.6151
2.5097	1.5502	3.6567	0.8355	1.4755	0.6375	0.8228	0.9646
3.7509	2.1479	3.8308	5.8225	2.2984	4.9936	2.0009	2.2453
2.2683	0	0.0619	0.0271	0.2831	0.03	0.5634	0.1333
0.56	0.3335	1.2846	1.4551	1.6431	0.7566	0.4408	0.255
0.9	0.7556	1.2779	1.0604	3.8522	2.2663	1.259	0.9879
0.6204	0.3369	0.8708	2.6467	0.3144	0.2912	1.7888	0.1667
0.1482	2.4571	0.7156	3.4547	12.4361	1.122	0.7656	2.6422
0.1453	0.3181	0.4845	0.8968	0.4845	0.437	0.2667	0.2718
0.2367	0.0947	0.0581	0.0763	2.812	0.0986	0.2513	0.3318
5.2486	2.011	3.3122	5.8238	5.3128	4.123	3.6305	2.5216
0	0.2773	2.8097	0.8378	0.0584	0.0619	0	1.0453
0.3954	0.5412	0.5178	0.4926	2.4341	0.7595	0.2534	0.8253
0.1771	0.1308	0.1807	0.3687	2.9936	0.0973	0.1096	0.1902
0.4083	0	0.9258	0.7751	1.5194	0.014	0.1804	0.3196
14.9963	12.7947	14.656	10.0377	12.0171	7.4799	21.737	9.6758

0.0275	0.022	5.1326	4.7555	0.0371	0.118	0.0554	0.0524
0.9409	0.1641	0.6964	0.793	0.1202	1.2551	0.4725	0.4358
1.404	1.6865	1.4959	4.83	3.5921	1.6172	1.2966	0.9805
1.9784	2.3501	2.3711	3.8767	5.6078	2.3151	1.4654	3.0969
0.664	0.5719	0.6522	1.097	2.295	1.2405	0.6165	0.7131
1.3755	0.3474	0.64	1.0529	0.2649	0.0739	1.4287	0.407
0.2682	0.2145	0.2092	0.4879	1.0526	0.2817	0.2645	0.1402
0.8116	0.0927	0.6619	0.1681	2.5593	0.1656	0.2721	0.1104
1.0638	1.4704	2.0523	0.5886	1.9961	2.9145	0.4491	0.4164
0.5197	0.3294	0.6549	0.2317	0.0925	1.1675	0.1797	0.1245
0.184	0.5352	0.1021	0.0154	2.5061	0.0068	0.5096	1.0646
1.7766	0.3778	0.8224	1.003	2.8798	1.25	2.0445	0.7767
0.1527	0.0163	0.5249	0.1443	1.4545	0.2181	0.1229	0.0388
0	0.1257	0.579	3.2414	3.0199	0.1123	0.3163	0.2494
1.8868	0.1335	3.1908	1.1856	4.2393	0.6107	1.8591	0.6097
1.7716	6.9187	13.4157	2.4174	14.9091	2.8289	1.7821	2.596
0.0106	0	5.9605	0.2607	0	0	0.0143	0
0	0	0.2917	0	1.0411	0	0	0
0.3942	0.2795	0.4554	0.5542	3.6718	0.3968	0.1322	0.2786
0.0547	0.175	0.1611	0.0705	0.1106	0.1172	0.1467	0.1388
0.7901	0.316	3.2687	0.7761	0.7566	0.3579	0.4685	0.5932
0.0471	0.0754	0.5555	1.5184	0.1271	0.1347	0.5689	0.0598
0.406	0.1664	1.159	0.2518	0.4994	0.1196	0.3369	0.3801
1.0724	0.3478	0.6406	1.0645	2.1295	0.9317	0.6025	0.3495
0.2266	0.2481	0.826	0.3382	0.9969	0.5964	0.224	0.1211
0.5364	0.4962	3.2529	9.3539	0.4821	2.2576	0.2741	0.1251
0.6416	0.2864	0.4397	0.4807	1.3477	0.7888	0.6604	0.8523
0.0101	0.0405	0.559	0.2216	0.0682	0	0.0068	0.3596
0.1727	0.1842	0.7996	0.2543	0.654	0.6814	0.6065	0.0939
0.5476	0.5739	4.5903	0.7057	0.8145	1.7535	0.2279	0.3116
0.0216	0	0.2071	0.1672	0.0146	0	0	0
0.3068	0.0126	0.9309	0.2872	0.2721	0.8765	0.9881	0.1697
0.1428	0.3739	1.5111	1.6396	1.0676	0.1484	0.9403	0.0989
0.8364	0.945	0.4589	0.4325	1.2976	0.6387	2.0148	0.9747
1.3183	0.8684	0.4285	1.1493	1.3591	0.8587	0.9361	0.7629
0.8615	1.1164	1.0069	0.4962	15.5383	4.5313	1.1231	0.4643
2.1184	19.533	7.4909	1.6877	0.5192	0.6053	0.2583	8.9959
0	0	0.0391	0	2.8969	0.0379	0	0
0.6452	0.3226	1.0992	0.1819	3.0171	0.9218	0.2434	0.4351
1.1477	0.7601	2.1002	1.4978	2.6804	1.1071	0.2647	0.3804
0.6173	0.237	0.4548	1.3206	1.1984	0.5116	0.149	0.1567
0.4372	1.1282	1.2468	1.6177	0.6465	0.2015	0.3594	1.3785
2.4056	0.8847	2.6095	0.6078	2.4836	1.9681	0.9602	1.0293
2.3593	0.8336	3.1348	0.8767	1.639	0.9513	0.66	0.6614
1.4848	0.5393	0.6318	0.3801	1.711	0.7721	0.0499	0.1573
2.5764	3.7209	10.5178	4.2424	3.6179	5.7259	3.3595	2.6798
1.0357	0.3222	0.551	0.5561	2.7923	0.4521	0.6945	0.2556
0.7347	0.1006	1.6749	0.0282	2.0887	0.0231	0.1048	0.023
6.3369	1.5209	8.1136	4.362	5.8406	3.8435	2.4673	2.2379
1.6095	1.2893	1.4598	2.3216	1.5434	2.8787	0.5786	2.2355
1.2602	0.8602	2.7108	1.2451	1.461	1.1643	3.1888	0.7038

0.0492	0.1732	0.928	0.6595	1.8973	0.1828	0.4356	0.0125
1.6349	0.954	2.5077	2.6481	6.6556	4.512	0.9531	1.7315
1.0839	0.4892	2.2629	0.4116	1.2047	0.6845	0.401	0.5478
0.0512	0.7779	2.0739	1.9789	3.5881	1.097	0.0343	0.065
1.6029	0.8863	3.2922	2.0372	6.9109	2.4538	1.3127	1.0783
0.3683	0.7295	1.1112	1.3562	0.3547	1.3532	0.1411	0.1558
10.7436	6.5118	10.7562	8.1585	22.7562	18.9828	5.1896	6.191
0.0208	0	0.1224	0.241	7.5065	0.3265	0.0279	0.1319
0	0	0.024	0.021	2.5463	0	0.0218	0.0413
0.5803	0.4379	1.0952	1.2157	0.6927	0.0749	0.4144	0.4005
3.0025	1.3316	2.5403	2.9882	3.9674	1.6989	2.791	1.7735
7.3474	0.3816	0.1406	0.8608	0.1286	0.2727	5.0552	1.8168
0.9014	0.2148	1.7802	0.2348	2.3657	0.7535	0.4502	0.5234
0	0.0877	0.0404	0.0706	0.2586	0	0.0735	0
1.5395	0.7037	1.2499	1.0122	0.8895	0.9876	0.0421	0.5583
2.2477	0.1893	0.2324	0.2541	0.319	0.2817	1.9569	0.4004
0.4777	0.2939	0.6903	0.2249	0.6935	0.0656	0.0986	0.07
0.4806	0.2439	1.8995	1.9651	1.576	1.7495	0.6817	0.1525
0	0	0.0604	0.4751	2.927	0.3512	0.1099	0.26
0.5424	0.2169	0.1998	0.4587	0.5255	0.3875	0.6138	0.043
0.1895	0.3918	0.8987	4.5019	1.3455	0.3301	0.0744	0.0235
0.4739	0.1234	0.0974	2.2013	0.0149	0.5196	0.34	0.2238
0.5838	0.0764	0.0363	0.8932	0.1703	1.1201	0.2624	1.134
1.1936	0.9547	0.6343	2.0173	3.8908	0.643	1.076	0.7327
0.7186	0.199	1.2012	1.6561	0.503	1.0859	0.2965	0.2806
0.0423	0.1015	0.7481	1.7722	3.565	0.0605	0.2838	0.2685
1.0352	1.5373	0.4447	1.6273	0.0175	1.4504	2.1902	0.1377
0.1747	0.1048	0.2252	0.3376	3.7086	0.1872	0.3221	0.2494
0.4896	0.5013	0.3462	0.1767	8.105	0.056	0.0525	0.6215
4.1428	1.1844	2.0424	1.015	6.413	1.5529	1.0141	0.8798
0.9653	1.1369	1.3596	2.4742	1.0153	1.0911	1.4938	0.552
3.3919	1.5743	6.0462	1.9151	2.3245	4.0235	0.9281	1.0044
0.2338	0.36	2.4365	2.393	0.2621	0.7054	0.4219	0.1081
1.4325	0.4264	0.5399	1.1163	5.2995	1.1424	0.6702	1.1841
0.393	0.6478	0.4036	0.5986	0.9392	0.3573	0.3674	0.3175
0.8944	6.2	0.1464	0.8324	0.0223	0.0237	0.0222	0.5886
0.2158	0.0987	0.0909	0.3974	1.0392	0	0.3308	0.2348
0.6808	0.4164	3.0977	0.9032	0.5399	0.6866	0.6714	0.0762
2.4054	0.5685	2.6465	0.0302	0.179	1.0154	0.9061	0.3024
2.4412	2.1067	1.322	3.5273	16.1982	2.6272	3.0564	1.923
0.6987	0.2619	0.6625	0.4754	1.1563	0.448	0.4113	0.4273
0.4399	0.1907	0.3392	0.2172	0.9089	0.0999	0.3364	0.214
9.9759	10.0693	21.2869	7.5363	25.9191	28.8644	19.2033	11.9428
1.4423	0.5373	2.7945	0.3565	1.14	1.1573	0.3445	0.4414
0.2396	0.24	0.4058	0.5714	1.4008	0.4703	0.2673	0.307
0.3551	0.0793	0.3163	0.7662	0.6123	0.177	0.3544	0.1887
2.7073	5.6257	2.7598	1.6222	14.6729	2.3408	0.8374	6.0678
0.6605	1.0566	1.4262	0.5458	0.6933	0.4322	0.8554	0.3744
0.5661	1.0467	1.2137	0.4456	2.0885	1.3561	0.6638	1.3942
0.3551	0.2177	4.3981	0.3272	1.0491	0.2608	0.5363	0.2738
0.4529	0.6883	1.2678	0.6712	0.7326	1.0353	0.8807	0.3449

1.0307	1.1398	2.5582	1.2659	4.215	1.8481	2.8297	0.6849
1.2169	1.0265	2.0211	0.8411	1.1632	1.4067	1.0831	1.3902
1.2388	0.1728	1.4695	0.6058	2.3123	1.5067	0.1363	1.2499
0.7365	0.9601	1.4871	0.6679	0.3677	1.7539	0.9879	0.5886
2.4947	1.7618	5.5603	2.9942	17.6931	0.0096	3.0307	2.1906
0.0864	0.2418	1.9086	0.2226	0.6403	0.2468	0.5212	0.3014
0.3035	0.2428	0.313	0.4302	0.0818	0.4336	0.4071	0.3467
0.0787	0.042	0.0386	0.1014	0.1273	15.0502	0.0141	1.2585
0.6764	0.3949	0.146	1.9762	0.1467	0.6831	0.3232	0.2837
0.8616	1.6636	2.3814	4.5871	2.988	0.866	0.7412	0.2037
1.8872	1.0292	6.6401	2.9125	0.3024	2.6491	1.8408	0.959
1.6796	1.3329	2.0784	4.6076	7.628	6.9843	2.6311	2.2759
0.3117	0.6396	1.4576	0.1921	0.4202	0.0194	0.3544	0.3354
0.0201	3.6869	17.8157	18.8015	6.7609	31.6126	4.1825	3.5834
0.4858	0.7253	0.7634	1.5024	0.4802	0.6015	0.3475	0.9866
1.0319	0.2266	0.477	0.3911	1.9366	0.6649	1.1669	0.2825
0.1719	0.225	0.2532	0.6041	2.4855	0.2902	0.0838	0.1587
2.4198	1.5048	1.5109	1.1789	0.2586	0.3584	2.0037	0.3558
0.8563	0.2061	0.882	0.7031	2.7785	1.0285	0.2338	0.1924
1.1176	0.8053	1.9819	1.2352	2.5413	1.1117	1.7251	0.8889
1.1366	0	0	0	0	0	0.4573	0.1443
2.0384	0.495	1.9307	1.3369	0.4171	3.0424	1.0008	1.2706
1.0862	0.9821	0.5566	0.2434	1.7825	0.1349	0.3484	1.3188
0.9923	0.1323	0.8772	0.3197	0.3344	0.0709	1.0869	0.126
0.0214	0.0117	0.153	0.0188	1.3914	0.0067	0.0168	0.4252
0.2467	0.074	0.4316	0.1391	1.0184	0.0441	0.4549	0.0783
1.6665	0.4416	0.5272	0.5138	1.2404	1.0663	0.6445	0.9344
3.5368	1.9956	8.6921	6.5437	4.6492	7.1918	1.9486	1.602
0.1877	0.7767	0.4578	2.0438	1.2304	1.1098	0.5904	0.6984
5.3775	3.8277	4.5843	3.1055	4.1991	4.7829	4.2467	5.9655
0.6697	0.6101	1.1991	3.2902	0.8777	2.0597	0.5738	0.6552
0.7537	1.4894	2.2536	1.8855	2.6596	0.285	0.5946	0.619
0.2022	0.0863	1.5095	0.0869	12.7759	0.1541	0.1446	0.2567
0.2293	0.3668	2.9434	1.3084	0.7947	3.1818	0.3075	1.3303
1.2186	0.5796	3.6072	6.098	1.7316	3.6077	0.4565	0.3763
0.8643	0.158	0.6731	0.1909	1.2483	0.1764	0.265	0.1567
1.2855	0.1455	0.6119	0.8411	0.2773	0.1865	0.8754	0.5121
3.8084	2.8612	2.6661	0.5423	5.1908	1.924	1.2417	1.8161
0.6512	0.1321	0.9189	0.2009	1.6569	0.1835	0.2214	0.163
0.5995	0.7847	1.3996	0.5719	1.5745	0.2559	0.2611	0.4349
0.2513	0.2144	0.2222	0.2159	15.6743	3.6384	0.1124	0.2765
1.122	1.4957	0.4133	0.6828	1.8066	0.6234	0.0418	0.6725
5.8027	2.406	2.7451	5.1898	6.7595	7.6479	2.4049	2.7875
1.7654	0.0703	0.0388	0.5999	0.2013	0.0753	0.7775	1.3936
4.7853	1.4507	5.5294	0.881	10.4006	1.2956	1.6841	1.8417
1.1708	0.6424	1.3451	0.5556	1.8062	0.9662	1.3208	0.794
3.8756	6.412	8.1991	0.9818	4.912	6.957	0.9774	5.0035
0.6078	0.2652	0.809	0.6364	0.8939	0.6069	1.297	0.5436
0.6253	0.0834	0.499	0.3357	0.8078	0.3722	0.1398	0.2646
1.4709	0.7961	1.3948	1.6193	2.9723	0.9748	1.7399	0.8322
0.0069	0	2.2414	1.1369	1.7699	0.0198	0	0

0.9235	0.5429	1.5655	0.6381	2.6249	0.9856	1.6415	0.1624
0.3747	0.3173	1.851	0.3124	0.5646	0.8817	0.1774	0.2238
0.554	0	4.928	2.6973	0.8299	0	0	1.6149
0.932	0.3856	1.2666	1.2218	3.4009	1.5726	0.8405	0.7139
0.4251	0.2473	0.3416	0.3984	0.6773	0.0552	0.4665	0.3434
0.1703	0.1767	0.0678	0.2076	5.1249	0.2828	0.2778	0.2337
0.0139	0.0074	0.0889	0.0179	1.6392	0.0066	0.0062	0.053
0.5897	1.4452	6.3963	0.5821	0.7781	0.3765	0.7068	0.8123
0.1216	0.6809	3.7329	2.5859	1.0656	0.2317	0.0272	0.1801
2.1222	0.679	0.3949	0.6909	2.1983	0	0	0.0284
2.8096	2.6176	1.9984	1.6231	2.9459	0.446	1.155	1.6397
0	0	0.1423	0.083	10.376	0	0.3887	0
0.3799	0.1737	0.7677	0.8813	48.424	0.6669	0.2766	1.433
0	0.133	0.9184	0.1071	0.2241	0.0594	0	0
0.6023	0.8029	1.331	0.6144	2.4695	0.4302	1.5819	0.4778
0.4152	0.1775	0.3914	0.32	1.6943	0.1643	1.1602	0.3062
1.3232	0.672	0.8974	0.7308	0.7078	1.2903	0.5916	0.8798
0.0057	0.0091	2.8957	2.2913	0	0.0081	0.4724	0.822
0.2191	0	0.4842	2.9176	2.4367	0.2869	0.6857	0.3013
1.068	0.3294	1.5925	0.7296	1.6826	0.4412	0.5177	0.392
0.6169	0.2961	0.9509	0.2079	0.934	0.2644	0.6174	0.1867
1.4671	0.7823	3.5249	0.8629	1.6937	1.0534	1.0393	0.487
0.9935	0.379	1.2724	0.5318	0.3606	0.5678	0.6663	0.194
1.6467	0.2492	2.6557	0.4588	0.6	0.4133	0.4775	0.5084
0.4278	0.2124	0.6085	0.7698	2.754	0.2213	2.2157	0.2902
0.1651	0.2086	0.3457	0.0784	1.5817	0.1863	0.373	0.1875
0.9817	0.3926	1.0332	0.482	1.6228	0.9518	1.0346	0.4747
0.8052	2.2368	1.0501	0.3926	1.1441	1.7178	2.0066	1.1602
12.9157	11.8767	26.4714	8.6062	19.2394	7.5072	2.5007	4.7332
0.9764	0.808	1.3394	2.5311	17.1119	2.8382	0.8279	0.6981
0.9719	0.0316	1.2211	0.089	1.9087	0.5498	0.3772	0.0877
0.456	0.8381	1.4151	0.4626	1.6479	0.6515	0.6116	0.2586
0.8286	0.8877	1.537	0.9535	13.1257	0.4123	1.7564	2.6202
2.6648	1.0796	5.8638	1.6502	8.5844	1.5575	0.4874	0.3734
1.4366	1.4939	1.2065	0.5739	1.2201	0.3695	0.6937	1.9694
5.9911	3.1085	5.2485	7.4286	7.3562	11.9836	11.3365	5.0766
8.4936	21.9326	9.6574	2.1449	2.3671	3.6201	15.0587	25.657
0.1072	0.2255	0.5616	0.0972	0.0321	0.0028	0.0772	0.1814
3.1347	1.2724	1.3097	0.6873	4.8439	1.1096	1.7319	1.7697
0.445	0.1734	0.7145	0.4338	1.3537	0.4728	0.352	0.3042
13.8784	6.9134	12.9458	4.1558	7.8322	2.8353	1.8562	3.1984
4.6295	1.841	8.0097	2.9063	8.2202	6.3876	0.4435	1.9812
1.3933	0.4101	0.1743	0.3981	1.2404	0.6949	0.6435	0.6674
0.4906	0.5783	1.4076	0.5324	3.4809	0.332	0.7965	1.0488
0.5154	0.3043	0.1672	4.5111	0.062	0.6575	1.6089	0.6464
0.014	0.0074	11.3123	0.4675	0.1317	0.0199	0	0
0.1171	0.1423	0.8341	0.7743	1.962	0.1519	0.1862	0.0798
0.667	1.0456	1.8383	1.6401	0.2842	2.0428	0.419	0.8199
0.2861	0.1325	0.3106	0.65	0.6698	0.086	0.2625	0.2102
2.4154	0.9628	1.1473	0.9843	3.1571	0.5645	0.4051	0.6983
0.013	0.0417	0.269	0.7227	2.2152	1.2856	0.2274	0.3807

2.0047	0.6948	5.5134	0.6028	0.8107	0.0955	2.1062	0.1696
0.2264	1.057	1.0637	0.7884	2.3093	1.9143	0.3036	0.963
0.5832	0.0835	0.6925	3.1856	0.0587	0.3358	0.8055	0.1105
0.3753	0.1334	0.7988	0.1612	1.2931	0.2979	0.3915	0.1059
0.6483	0.122	0.3933	0.1474	0.5141	0.4359	0.2046	0.1452
5.8173	3.498	10.957	1.7706	17.1976	4.4773	2.7445	2.8126
0.4529	0.2415	3.2918	0.4474	0	0.0431	0.3644	0.2108
0.1185	0.6769	0.2244	0.7852	0.7986	0.3627	0.2043	0.7734
0.8051	0.8243	0.949	0.913	2.2141	0.2531	1.339	0.7562
0.0044	0.2306	0.7337	0.8332	1.3839	0.6678	0.1406	0.7929
0.2901	0.3375	0.4352	0.4146	2.0834	0.2035	0.6438	0.3348
1.3235	1.8934	2.6438	1.066	4.5635	2.7637	3.4138	1.0338
2.4584	1.1054	5.2402	2.2589	5.2653	2.3589	1.1999	0.9509
0.9452	0.3544	0.6746	0.5139	0.438	0.2954	0.7329	0.15
4.2239	8.8336	4.1164	2.4098	3.3512	2.263	4.2194	3.8256
1.0516	0.2872	1.3857	0.1432	0.5302	0.3665	0.4014	0.5752
0.2885	0.1003	0.6645	0.3326	1.8223	0.7078	0.4392	0.1375
0.4133	0.0331	0.274	0.1864	0.8358	0.1181	0.3603	0.2623
1.3802	0.7813	0.8134	0.9577	0.3149	0.7281	1.4524	0.5121
2.0549	0.3674	0.9196	0.8878	0.8969	0.4711	1.0582	0.5531
1.4492	0.9865	1.1357	1.1523	0.6235	1.4097	2.3159	1.1742
4.4708	3.4193	3.6987	1.9895	6.8896	3.1977	4.8132	5.0701
15.4329	33.0939	19.7982	2.0507	2.3495	3.3201	7.5208	69.156
0.2363	0.3058	6.6923	0.7703	2.6517	5.3873	0.1025	0.4941
0.0774	0.09	9.8364	0.136	1.2708	0.0603	0.0755	0.1429
1.1395	0.2378	1.1771	0.4549	0.8599	0.5132	0.299	0.904
1.1964	1.0955	0.9277	0.6492	0.6578	1.0346	1.6257	1.059
0.6324	0.1716	0.4381	0.2086	0.8373	0.3092	0.2625	0.3249
1.2635	0.6829	0.6792	0.7041	3.4297	1.7808	0.8931	0.6068
2.7195	1.99	2.7349	1.6912	4.9551	3.369	1.3931	2.521
9.678	8.8276	8.2564	8.7323	3.2376	10.4369	13.2872	8.6736
0.8576	0.8438	0.8776	0.911	1.0739	0.7023	1.1406	0.5648
0.132	0.3802	0.7782	1.3954	0.1068	0.7546	0.0708	0.0335
0.2774	0.0887	0.5312	0.3932	0.3365	0.0396	0.2604	0.3873
0.1499	0.0932	0.2331	0.3058	0.6622	0.1784	0.5081	0.148
0.2693	0.5265	1.4988	0.1157	0.4235	0.1069	0.2608	0.3228
0.0898	1.4598	0.4849	0.2699	1.4318	0.342	0.2809	1.2912
0.9509	0.117	0.8083	0.9898	2.7117	2.6126	0.4415	0.6035
0.9688	0.3616	1.7605	0.1665	0.7183	0.346	0.5631	0.2869
3.6186	0.6744	0.88	0.3169	0.8525	0.2008	0.5183	0.2676
0.2717	0.3933	1.0295	0.2168	2.8956	0.3328	0.4339	0.5748
1.3571	0.2964	1.2625	0.388	0.6119	0.6287	0.7455	0.5527
1.3656	0.535	0.9855	0.5028	1.5404	0.3982	0.5233	0.4245
0.9241	0.2235	1.4723	0.5262	0.7967	0.3224	0.4179	0.6547
1.0932	0.8744	2.0769	0.0741	1.5125	0.6987	0.1929	0.4017
1.3612	0.3754	0.4149	0.0605	0.8859	0.3688	0.4092	0.3575
0.2048	0.2772	0.3713	0.2436	1.5714	0.7427	0.4225	0.3399
1.6624	0.4163	2.2577	0.6148	1.3252	0.3718	1.997	0.7523
2.8216	2.8849	2.4055	0.5938	2.9985	0.894	1.0834	2.4118
0.3876	0.0752	0.45	0.1514	1.7577	0.3692	0.2206	0.4026
0.9358	0	1.4554	0.67	1.7521	0.52	0.4184	0.198

0.1921	0.0922	0.4812	1.1388	0.6992	0.6999	0.0644	0.2804
0.7554	1.0979	0.8347	0.558	2.403	1.4412	1.0749	0.801
1.7114	1.5749	1.1938	0.6929	0.7963	0.4652	0.3961	0.2788
1.2484	0.7185	1.6263	0.3139	1.6214	0.2284	0.6739	0.5701
0.2445	0.0711	2.2109	0.1647	1.0714	0.4447	0.1938	0.0988
1.5955	0.8933	2.3115	0.7196	3.7281	0.5699	1.2126	1.2488
0.3312	0.1325	0.244	0	0.3349	0.4733	0.6664	0.4205
3.8461	0.8832	2.4673	1.0435	0.6946	0.6573	1.5303	0.6774
0.9681	0.2644	0.4175	0.8824	1.6234	0.4386	1.0134	0.9591
0.942	0.1159	0.8541	0.6537	0.5861	0.6212	0.3887	0.3679
0.7201	0.6002	1.4948	1.1671	4.0568	0.7176	0.8927	0.4122
0.0363	0	6.5394	6.684	0.5288	1.2766	1.6855	1.4476
0.5267	0.3389	3.8549	0.4206	1.2966	0.4581	1.3515	0.8721
1.2479	0.8334	1.3366	0.9802	43.5629	0.8066	1.9208	1.5136
0.1309	0.256	0.5144	0.3187	2.6081	0.4676	0.3024	0.3878
0.1141	0.2556	0.2018	0.2941	0.523	0.2609	0.3979	0.3766
0.5636	0.3088	0.8462	0.3117	4.5859	0.3047	0.5561	0.5384
0.79	0.1366	0.5977	0.6604	0.9211	1.3117	0.3436	0.2168
0.4789	0.3226	1.114	0.4547	1.0533	0.3601	0.4056	0.2239
0.8246	0.2363	0.8341	0.1586	2.0573	0.3341	0.4292	0.9061
0.1274	0.1359	1.5017	0.3831	0.458	0.5461	0.1709	0.1078
0.7884	0.3396	0.2681	0.3213	1.1128	0.3899	0.5061	0.9195
0.4322	0.1844	1.1746	0.9531	2.4603	0.398	0.2963	0.3414
0.9121	0.3072	0.7427	0.0619	1.1973	0.0686	0.2254	0.0609
0.4499	0.7197	0.3059	0.1784	1.1197	0.2967	0.3249	0.615
1.3092	0.7992	2.5127	0.3996	2.8332	0.2953	0.5083	0.2843
0.8808	0.1725	0.3443	0.5791	30.2163	1.0529	0.6991	0.9126
2.4124	1.1661	2.506	0.5815	2.2227	1.0663	0.3492	0.5948
0.449	0.1277	0.6174	0.3214	1.3719	0.3564	0.4416	0.3799
0.014	13.7868	15.0549	27.512	2.4976	29.6591	12.6882	5.0063
0.5582	0.4465	1.0755	0.3874	1.4182	0.4294	0.6335	0.1908
1.8444	4.1342	1.5977	1.4366	33.7174	4.4306	2.5961	13.0695
1.5658	1.0126	2.0616	0.9016	3.5031	0.714	0.3575	1.4801
0.5757	0.7924	1.716	0.1208	1.3897	0.1339	0.9517	0.6457
1.7583	1.0626	1.8258	1.9638	5.2147	2.0097	0.9283	1.5729
2.1654	1.0384	1.5416	0.3461	0.7392	0.7275	0.9306	0.2344
0.4699	0.2734	0.8434	0.5835	1.4051	0.9033	1.2376	0.835
0.1303	0	0.8317	1.0072	0.7024	0.1241	0.1747	0.4134
1.5198	0.4168	0.7357	0.1399	0.9073	0.1861	0.4368	0.3583
0.7781	0.249	2.4457	0.5348	0.4196	0.5558	0.5914	0.1975
0.7665	0.2299	1.0352	0.391	2.2389	0.4563	1.5635	0.7905
2.5309	1.2147	1.1904	0.6021	2.7558	0.4172	1.6059	1.5198
5.193	4.83	8.7324	2.0472	79.135	8.6006	1.6322	0.9905
0.093	0.0458	0.6112	0.4424	1.0702	0.2861	0.259	0.2451
2.7741	1.6747	4.2029	1.6863	3.8455	6.8425	1.5795	1.0962
0.9943	0.8908	0.4102	0.8713	0	1.3922	0.6668	0.1515
0.8401	0.4838	2.7974	0.4547	0.7475	0.2881	1.5775	0.128
1.5547	1.0482	14.7909	1.0018	6.1379	1.5549	0.4319	0.5638
0.3587	0.2066	0.7611	0.3883	0.7157	0.123	0.5773	0.4735
0.3939	0.2262	0.7588	0.4035	3.4716	0.3824	0.9075	0.1474
2.9291	1.9872	3.8637	1.0079	2.6876	3.1551	3.5583	3.6401

1.2905	0.333	0.4293	0.7511	2.0483	0.5948	0.7538	0.1849
0.2061	0.0412	0.3796	0.5644	0.6252	0.3313	0.4492	0.5886
0.613	0.469	0.7854	0.4122	1.6168	1.3708	0.9294	0.3721
2.179	1.9747	1.4386	2.4552	2.8305	1.5027	2.9864	1.7935
0.4734	0.3091	0.4128	0.498	1.1071	0.3589	0.5572	0.5151
1.0095	0.8748	0.4106	0.8334	2.3391	0.7362	1.2341	1.0011
8.2286	8.3063	12.2786	31.5924	11.6067	21.2592	12.7818	7.3414
0.2585	0.4486	1.3557	0.3388	0.2422	0.5759	0.6171	0.3281
3.8973	2.6463	3.8927	2.0691	4.089	3.4322	3.0708	3.0422
2.5856	1.4678	7.1583	2.5528	3.7386	1.579	3.9154	2.3556
0.7996	0.0365	0.8302	1.158	0.1899	0.5168	0.4136	0.8603
3.6779	4.741	0.3982	0.5349	15.2908	0.7034	2.706	8.2832
0.0056	0.2595	0.0577	0.0793	2.2769	0.04	0.2775	0.2698
2.2122	0.1729	0.1656	0.2312	0.37	0.3103	0.7059	0.2894
2.0195	4.0684	2.8102	1.5905	3.1762	3.0457	1.254	1.3767
0.4048	0	0.0643	0.4194	0.1498	0.6805	0.5217	0.2015
2.1455	1.7251	0.9467	1.1449	0.6252	0.8142	1.6262	3.5744
0.5029	0.302	1.2185	0.2132	1.4708	0.2029	0.7978	0.2418
6.4279	6.3242	4.9677	3.0057	36.7376	4.0836	2.6635	10.1379
0.9374	0.2298	0.7982	1.4223	0.3567	0.7128	0.3954	0.2207
0.964	0.1691	6.9002	1.3519	21.9287	1.5798	0.1003	0.2127
0.5775	0.4098	0.6845	0.3917	0.878	0.8747	0.4834	0.4409
0.0218	0	5.4802	0.028	4.2812	0.0311	0.0583	0.0276
0.0833	1.8485	0.4338	0	8.5223	0	4.3736	0.2962
0.6993	0.5817	0.5357	0.1982	0.6787	0.2398	0.1313	0.426
1.7222	0.5387	1.7436	0.5952	3.3204	0.4124	0.9162	0.8549
0.3702	0.1052	0.5556	0.0089	6.7255	0.0099	0.0371	13.6797
0.0647	0	1.773	0.0333	0.157	0	0.1215	0.0164
1.1479	1.1352	2.214	0.6321	1.9976	0.8349	0.8678	0.5563
0.282	1.0577	1.3111	0.42	1.4869	1.4729	0.5589	0.4494
1.2819	0.0901	3.2586	0.2723	0.8546	0.5434	0.5857	0.5722
1.279	0.4439	1.7567	0.5085	2.1509	0.4026	0.221	0.2862
0.538	0.1545	0.8028	1.6798	1.655	0.1084	0.2035	0.2364
0.7713	0.2314	1.0654	0.3106	0.3574	0.8609	3.6207	1.5603
1.2314	0.0152	0.2233	0.5493	0.1405	0.1624	0.3938	0.4208
1.0054	0.4791	0.6303	0.2205	0.7209	0.2751	0.2582	0.2444
0.6056	0.1761	0.3893	0.4682	0.8312	0.4247	0.7679	0.4472
0.7348	0.1931	0.4794	0.7102	0.0071	0.9748	0.0352	0.2332
3.1493	0.8839	1.9944	2.8007	1.5642	2.6576	0.5311	0.8533
2.0577	1.6764	0.5053	0.2824	2.0291	0.9936	1.4949	1.5478
0.1493	0.3184	1.063	2.4848	0.1509	0.9064	0.2169	0.1105
0.0174	0.2499	0.8694	0.1566	2.1525	0.0496	0.4423	0.5728
0.9677	0.5359	0.7403	0.4317	7.0243	0.4254	0.5741	0.8504

TCGA-CV-69	TCGA-CV-74	TCGA-CR-73	TCGA-CV-74	TCGA-CQ-62	TCGA-CV-69	TCGA-CR-73	TCGA-CV-74
0.957	4.7376	4.3968	0.6961	0.9256	0.2158	0.2153	2.9282
5.3316	3.6515	2.1943	2.387	2.3959	2.1288	4.1565	1.3116
2.7841	1.2645	1.1672	0.647	1.1366	0.6548	1.2689	0.8382
2.0285	0.7958	0.9056	2.2559	0.2886	0.3884	1.1208	0.7998
5.1316	1.2993	0.676	1.0995	0.9222	0.4257	1.4072	0.6105
3.1509	0.7707	0.8113	0.6468	0.8552	0.9494	0.6538	1.1158
1.3561	0.5057	0.7625	0.6544	0.5306	0.4097	1.0804	0.4638
3.3361	0.662	0.7371	0.9651	0.9439	0.7498	4.0209	0.3483
1.8171	0.2828	0.1833	2.691	0.1597	0.2624	0.1428	0.1852
1.0349	0.3718	0.7404	0.3821	0.6195	0.5274	0.4961	0.332
46.13	4.0659	0.5746	1.5457	1.2267	0.5561	1.7074	1.0911
0.615	0.615	0.8238	0.2334	0.5487	0.1225	0.188	0.2887
0.2818	1.0981	0.6771	0.4375	0.5609	0.2984	1.7665	0.6728
6.6764	4.0141	0.4172	1.9903	2.0315	0.038	0.1355	0.0144
3.6258	1.431	0.8579	1.5112	1.6354	0.6255	2.4461	1.1577
5.0803	0.9386	1.9018	1.8094	1.407	1.3745	1.2523	0.8591
3.5581	3.4832	3.6335	1.3021	2.3432	3.238	7.9556	3.623
1.6595	0.4093	0.2849	0.3991	0.9416	0.4257	1.2354	0.967
2.0849	0.8901	0.9609	0.9971	0.9973	0.6961	2.2841	1.8987
2.8302	0.4008	1.1815	1.2067	0.7326	2.7027	0.6417	0.7574
4.9312	8.6695	8.3889	8.0609	11.0803	9.7256	17.8919	6.509
2.4789	2.1252	4.4956	5.5384	1.9327	4.3193	1.9531	5.0902
2.2722	1.0688	1.2525	0.7271	1.3705	1.1917	2.5194	1.1764
0.7787	0.675	2.0834	1.1335	1.0343	0.6278	3.371	1.2955
2.3121	0.6563	2.4369	1.425	0.7505	1.1518	1.9689	0.9229
11.0498	0.5714	3.8322	1.7317	2.0797	2.0803	5.321	1.183
0.8511	1.0917	0.3445	0.3853	0.3787	0.1409	0.1862	0.4375
10.1073	4.3328	8.2166	6.1947	5.8367	6.8022	7.3744	14.8245
0.0807	0.6476	0.5947	0.5143	0.2516	0.0886	0.388	0.5313
3.5501	1.9558	2.0596	0.7268	0.9385	1.1067	0.4639	1.1761
1.093	0.5113	0.366	0.8096	0.7019	0.4944	0.9307	0.3984
0.1458	0.0163	0	0.1664	0	0	0.087	0
1.7818	1.3764	0.3557	1.0414	0.5164	1.7457	2.4486	0.069
2.39	0.3285	0.8526	1.0384	1.2908	0.6209	1.0999	0.4112
2.1222	0.4893	2.2968	1.4113	0.8067	0.7832	1.2727	0.739
14.1287	3.5627	1.0961	1.2056	1.2077	0.7471	1.868	0.6451
1.5883	1.7196	2.4343	1.287	2.3699	2.4245	11.1152	2.6943
0.5554	0	0.3951	0.0865	0.1229	0.7272	0.402	3.6407
1.6718	1.0621	1.231	0.5117	0.2957	0.2484	1.6491	0.4442
5.3559	0.6711	1.7164	1.0681	0.911	1.4889	0.8599	1.204
3.239	1.1785	0.6633	0.4786	0.1749	0.2464	1.0185	0.6286
0.2654	1.722	0.6018	5.2203	1.9529	3.4996	15.404	0.2014
2.5188	0.6068	0.3905	0.4618	0.4834	0.3026	0.9057	0.3887
2.2762	0.2918	0.3976	0.4332	1.4085	0.7318	0.7551	0.0643
13.2136	4.1956	3.7361	4.1622	4.409	3.8881	8.0456	3.0854
0.1042	1.5446	0.6989	1.4276	0.279	0.0715	0.0259	0.6213
1.0878	0.3249	0.3129	0.6813	0.3908	0.9923	0.5203	0.6069
4.225	0.4214	0.2929	0.6544	0.3189	0.6739	0.9452	0.0888
0.7582	0.9568	0.4402	0.0134	0.7584	0.9755	0.038	0.803
13.4285	9.457	17.3752	17.6813	10.2162	10.3686	16.6975	15.3384

0.1986	0.3703	0.037	1.5685	0.1289	0	0.1153	0.0179
0.536	0.8216	0.9408	0.3305	0.3862	0.3382	0.4214	0.6741
6.1023	2.6329	2.3436	3.8771	1.9533	1.5899	2.1879	1.3993
2.4136	4.1448	1.5128	2.6444	5.0593	3.0063	4.0135	5.8208
4.0519	1.7858	0.7321	0.3038	1.2953	0.5895	3.4009	0.8431
0.5719	0.4729	0.0834	0.3407	0.0726	0.648	1.3732	0.1348
0.493	0.7106	0.1908	0.4871	0.3785	0.3641	0.434	0.1645
1.0104	0.5847	0.2337	0.179	0.3053	0.3106	0.3467	0.1511
4.3948	0.9206	0.4301	0.9989	0.6466	0.7699	2.0546	0.2999
2.5853	0.6989	0.3075	0.4396	0.4247	0.1401	0.1251	0.1491
0.9995	0.0289	0.1477	1.6955	0.3664	2.6199	2.7297	0.0374
3.4066	2.6996	1.2767	1.4512	1.7278	0.7168	4.3018	2.0706
3.4748	0.3422	0.4377	2.0396	0.1668	0.1175	0.9497	0.0929
10.2985	0.6871	0.4225	1.0248	0.138	0.1296	0	0.0512
0.3425	1.344	0.9436	3.8858	0.162	0.8725	5.7826	0.2228
29.7197	5.3256	1.2779	4.0763	4.6507	5.0473	3.8645	2.4108
2.8154	0.1718	0.0143	0.0438	0.0374	0	0.0382	0
0.0238	0.0133	0	2.6497	0	0	0	0
1.5724	0.6025	0.9933	0.8485	0.5191	0.2437	0.852	0.4729
0.6574	0	0.9921	0.5629	0.224	0.0902	1.7336	0
3.0576	1.393	1.6023	0.3306	0.3863	0.5004	0.9797	0.6247
1.8128	1.6479	0.5066	5.8214	7.2267	1.0879	1.4094	1.167
1.1773	1.1193	0.6001	0.4562	0.4604	0.4728	1.8698	0.5093
1.9859	0.838	0.8763	1.9691	0.5598	0.4301	1.6122	0.5855
0.8316	0.9464	0.4488	1.8662	0.2792	0.177	0.9845	0.5441
2.3775	2.6501	0.6816	0.5109	1.1875	0.1264	0.5849	0.409
0.7174	0.5619	0.4612	0.4915	0.6812	0.738	1.0888	0.2723
2.043	1.5238	0.1224	0.0208	0.5921	1.1843	1.4764	0.7647
0.1779	0.5197	0.4088	0.2821	0.2406	0.0678	0.4721	0.2143
0.227	0.9142	0.4821	0.8293	0.0221	0.9962	0.3614	0.4184
0	0.1309	0.0291	0.1039	0	0	0.0129	0.0141
0.3025	0.1728	0.451	0.8096	0.3591	1.0677	0.3575	0.246
0.5306	1.2047	1.9191	1.7997	0.0456	0.1498	0.7765	0.2538
2.0655	1.5238	0.5683	0.3243	2.6384	0.8721	1.3425	1.398
6.2922	1.6165	1.485	1.7562	1.0666	1.3745	2.296	1.9203
3.3669	2.6665	0.8601	2.4175	2.1181	0.5158	1.9919	1.3611
0.463	3.4187	4.7618	2.326	0.1353	24.1321	0.5529	29.166
0	0	0.1426	0	0	0	0	0
6.1561	0.461	0.2439	0.6088	0.5664	1.2965	0.6512	0.2628
5.2546	1.7695	0.6483	1.6654	2.0964	0.6629	3.2791	0.6478
2.6121	0.4317	1.2775	0.7287	0.3758	0.0611	0.2363	0.0966
0.9155	1.0243	0.4359	0.1936	0.908	0.3721	1.8221	0.4596
4.4781	1.2457	0.8617	2.6124	1.672	1.2587	1.6884	0.7164
10.4042	1.09	0.7975	0.9734	0.9318	0.8114	1.5409	0.6414
0.0715	0.1133	0.4364	0.6328	0.148	0.0777	0.3618	0.5395
16.0436	7.0499	6.7078	5.4513	6.1781	2.4484	3.4671	3.1479
4.7721	0.3095	0.6572	0.9871	1.0102	0.5692	1.2045	0.3
0.2955	0.6279	0.7657	0.3936	0.0625	0.0251	3.1334	0.4632
2.8728	4.3145	2.9347	3.7447	3.5314	1.8101	4.6087	4.6838
3.7383	1.0934	2.0227	0.7971	1.9256	1.5359	2.3932	1.1462
4.5242	2.6664	1.7386	1.1415	1.6324	0.748	8.6829	0.8759

0.5915	0.5162	0.8332	0.5538	0.0691	0.0487	0.3414	0.1154
1.2007	0.8487	0.4675	0.5013	1.7451	1.4175	3.3259	1.7811
2.8673	0.8774	0.4246	1.0027	1.1097	0.7283	1.1056	0.6288
2.4611	1.6521	0.8942	2.0022	0.0899	0	0.3061	0.0334
2.9923	2.3196	2.0548	1.9922	1.7336	1.2179	2.1737	1.3911
4.6805	3.7976	0.4007	0.337	0.5543	0.1157	0.2728	0.2057
26.8071	3.658	6.9306	5.7805	7.9065	7.9745	6.9371	9.1341
0.3496	0	0	3.1938	0.4621	0.0685	0.0746	0.0271
1.9964	0	0.3063	0.067	0.0953	0.0268	1.6556	0
1.3477	0.7881	1.0077	1	0.8874	0.4324	1.6549	0.4625
0.6432	2.9184	3.1159	4.2024	2.3314	1.5684	5.5118	2.2859
0.2294	0.1283	3.0133	0.3929	1.6196	4.0905	3.4814	5.472
2.0518	0.9801	0.3222	1.2766	0.8643	0.5534	1.0897	0.4249
11.2015	0.0369	0	0.0752	0.0321	0	0.3606	0
1.5107	0.9718	0.5911	0.2587	0.6252	0.6216	0.6389	0.3685
0.1896	0.2652	1.8017	0.6495	0.7385	0.2601	1.5095	1.6446
1.2368	0.6177	0.0988	0.5926	0.3549	0.3787	0.4506	0.1916
3.8881	1.5784	0.7203	0.9893	1.1304	0.1905	1.299	0.3674
0.8864	0.1102	0.0551	0.8996	0.0959	0.4728	0.5391	0.1068
5.7048	0.4331	0.4555	0.8375	0.4563	0.4751	0.365	0.3093
0.6554	0.7643	0.8321	0.6722	1.5361	0.0076	0.304	0.265
0.053	0.4743	0.3702	0.2571	0.4386	0.0909	0.2109	1.264
1.9922	0.1491	0.9107	0.1184	0.7861	2.7351	1.5219	1.8107
1.9757	2.2499	1.5514	1.1951	1.2712	1.3227	1.7321	0.204
0.9966	0.8177	1.2813	0.3983	0.7279	0.319	2.215	0.5763
0.2034	0.1138	0.2559	0.1161	2.625	0.2442	0.3037	0.2206
0.3316	0.7532	0.2804	0.0324	0.0662	0.0136	0.0437	0.1967
1.0498	0.9396	0.2934	0.3596	0.7923	0.144	0.2612	0.2276
0.0942	3.5818	0.3158	2.2309	0.4355	0.5166	0.3514	0.1787
7.1952	1.1016	0.889	2.0322	2.0467	1.2726	1.922	0.8623
2.448	1.3124	0.784	0.6988	0.8443	1.2942	1.0024	0.9954
3.9681	1.6896	0.6631	2.2381	1.7495	1.8891	2.9074	2.1179
6.171	2.4326	0.9071	0.4797	0.4961	0.1801	0.1568	0.8342
2.7231	1.5681	3.4919	1.5546	2.2618	1.0436	3.3476	2.4315
2.0328	1.2572	0.5361	0.6701	1.0734	0.2749	0.3918	0.4656
0.0796	4.0092	0.3784	0	1.0469	2.4577	0.0198	0.9282
0.7413	0.622	0.2072	0.6348	0.9384	0.2034	3.0245	0.1206
9.5314	1.1579	0.7534	0.9619	0.4453	0.1321	0.527	0.2871
2.253	2.9723	1.3853	1.0879	1.7003	0.0966	0.7007	3.3946
4.9447	7.1558	0.9464	1.5711	4.6112	5.7798	8.3429	4.4639
1.3359	0.3814	0.5113	0.8196	0.4696	0.3916	0.6537	0.6341
1.1366	0.2433	0.1934	0.378	0.486	0.2305	1.0377	0.359
38.9135	12.0406	19.4275	9.5191	13.8211	15.145	12.178	12.7836
2.9639	0.7865	0.5098	1.627	1.0546	0.7036	0.7847	0.412
0.8883	0.1385	0.6139	0.6024	0.3198	0.2215	0.592	0.3065
0.9926	0.3998	0.233	0.476	0.8409	0.2723	1.0174	0.2906
5.1037	1.2159	1.2485	1.378	2.1283	5.1391	3.0772	2.6366
3.7635	1.1323	1.1382	0.7819	1.2541	0.7648	1.1278	1.5048
1.6544	1.0531	0.5011	0.7863	0.4682	0.3968	0.6529	0.486
9.8292	0.6003	1.019	0.6172	0.4118	0.342	1.5581	0.4087
2.9941	1.7358	0.3043	0.5283	0.6096	0.1867	0.921	0.8854

5.7873	2.4094	4.6199	2.6868	0.9959	0.7413	1.9197	0.8114
5.6382	2.7077	1.1001	0.8958	2.0848	1.7692	2.5805	1.0525
0.6567	1.2644	0.4524	0.9678	1.1972	1.833	0.4256	0.7617
3.4102	2.1278	0.2933	1.1232	0.2874	0.1349	2.1863	0.3555
10.784	3.4271	3.0139	1.6175	4.5865	4.556	4.2373	0.0131
5.0865	2.6421	0.5512	0.6519	0.556	0.356	1.3171	0.3939
1.897	1.1836	0.3263	0.5832	0.3908	1.0008	0.0726	0.5933
0.4414	0.1058	0.2185	0.216	0.0246	12.1611	0.0941	0.0547
0.4532	0.3084	0.3395	0.144	0.2525	0.3012	0.1023	0.5776
6.2287	1.902	2.4755	2.1205	0.7443	2.518	5.0612	0.2478
3.8865	2.0898	2.0173	2.9071	1.5602	0.7344	2.0523	2.0554
2.5863	0.3404	2.3881	3.6369	2.7747	2.0824	6.5794	1.9622
1.238	1.1117	0.8104	0.1116	0.9121	0.458	0.5188	0.7683
13.7621	7.6363	5.4407	9.3183	5.9481	3.7336	7.5471	9.2834
0.3114	0.7404	0.4787	0.1333	0.9477	0.4272	1.8595	0.3377
0.2432	0.7891	0.1088	1.5829	0.971	0.6338	0.0726	0.4747
1.0142	0.4833	0.9029	1.2652	0.1829	0.1803	0.1869	0.4276
3.1215	1.0765	0.7237	1.3872	0.7945	0.6143	0.2162	0.6298
4.7729	0.3567	0.4379	0.5096	0.1153	0.2624	1.2871	0.1876
2.8059	2.1436	1.3815	0.8867	1.0957	0.9155	2.0703	0.9665
0	0	0	0.156	0	0.5622	0.4079	3.1109
0.6563	1.1993	1.5408	2.398	2.2156	1.5304	0.8491	1.3284
0.1703	1.3019	0.2856	0.0648	0.8015	3.5038	0.6214	1.3848
1.9085	0.6673	0.4	0.0681	0.4259	0.2454	1.2859	0.3018
1.6333	0.0175	0.1444	2.606	0.0165	0.0138	0.0044	0.0136
0.6301	0.1037	1.6781	0.5925	0.1263	0.2034	0.6086	0.1005
1.3272	1.1549	0.5221	0.463	0.7061	1.0114	2.8128	1.3991
4.5513	2.9209	2.0904	3.1622	2.5596	2.8552	2.6508	3.022
0.3424	0.6616	0.5219	0.9862	0.394	0.7471	1.2235	0.9618
9.4635	4.0187	6.8721	6.0979	4.0188	3.4652	6.9281	3.4485
1.5652	2.3265	2.3435	0.5234	0.773	1.8785	1.6355	1.188
1.1191	1.3117	0.7149	1.0344	0.986	0.6579	3.129	0.7512
2.1066	0.1088	0.2536	0.1665	0.0473	0.0222	0.6289	0.2108
0.9448	1.1892	0.7042	2.4722	1.1501	1.8899	1.8805	0.1707
3.0881	1.2698	1.4458	0.4822	0.5783	1.1947	1.484	0.2862
3.4729	0.166	0.4977	0.6609	0.3902	0.3054	0.7385	0.1448
0.5706	0.2607	1.5787	1.0749	0.3936	0.8413	0.7902	1.1445
9.7628	0.9904	2.2054	2.1081	1.6009	4.4405	3.5741	0.8775
4.5424	0.2837	0.1356	0.8183	0.2255	0.4083	0.395	0.3945
1.7783	0.4084	0.4499	0.6839	0.4922	0.4622	1.3971	0.4972
1.4903	0.0676	0.2026	0.2299	0.5099	0.3315	0.6012	0.1965
2.3976	0.3772	0.335	0.8983	1.2039	0.7194	0.1118	0.2843
5.9886	3.9823	2.1662	1.7836	4.3755	2.0704	7.6189	4.4835
0.0845	0	1.4281	0.229	0.2159	0.5648	0.3467	1.8315
7.8484	2.7205	2.9247	3.1977	2.3516	2.9213	4.5227	1.4728
2.5708	2.069	1.0678	0.9572	2.9437	1.0035	3.6352	0.9034
10.671	1.2028	1.2018	3.5461	2.2489	4.0961	2.7341	1.5542
0.822	0.5852	0.5987	0.4171	0.3517	0.4897	2.772	0.6256
0.8142	0.1752	0.14	0.2861	0.305	0.1289	0.9661	0.4075
2.5205	1.2858	1.6233	1.4948	2.1742	1.2539	3.7942	1.4398
1.002	1.1771	1.4655	0	1.4392	0.0229	0	0

3.7983	2.4689	2.0259	2.2829	1.3219	0.532	1.5705	0.8773
1.2187	0.2075	0.5331	0.3025	0.9546	0.5815	2.2411	0.1436
8.6823	3.3667	0.386	0	2.3299	5.853	2.4301	1.7116
2.9166	1.1885	1.0365	0.9263	0.7242	0.5299	1.4897	0.8482
0.6503	0.3638	0.1039	0.3713	1.3571	0.3823	0.8783	0.4533
0.2102	0.3899	0.8226	0.4359	0.5872	0.3946	0.8754	0.2519
1.2831	0	0.0312	0.4332	0.0163	5.4101	3.2534	0.0181
1.3876	1.1812	0.7334	1.0849	0.705	1.2516	1.2232	0.3434
0.1462	1.0358	0.0272	0.1391	0.0949	0.2339	0.7031	0.0792
0.9667	0.9012	0.6904	2.1155	2.2226	1.1418	0.0534	0
1.3716	2.6203	1.3164	0.6205	3.3643	2.2718	1.6096	1.3609
1.0839	0	0.0866	0.2652	0	0.1593	0.5393	0
2.4532	0.6861	1.1232	5.9893	1.0547	1.0381	16.1409	0.481
0.0999	0	0.2234	0.6274	0	0.0685	0.4474	0
3.6801	1.7886	0.1686	1.1021	1.0868	0.4551	1.3208	0.2944
2.1579	0.7322	0.2161	0.0824	0.3133	0.5957	1.0543	0.7693
0.4544	0.6214	0.762	1.2683	1.2047	0.5887	3.9445	0.8759
1.2837	1.841	0.0076	1.7853	0.8311	0.5526	0	0.0074
0.6145	0.5156	0.1227	0.3007	0.5984	0.2709	0.7644	0.119
5.0114	1.592	1.2104	1.0773	1.1899	0.5516	1.1236	0.4863
5.3394	0.8552	0.6696	0.5992	0.6388	0.3912	0.5961	0.4268
11.3767	2.6104	1.0009	2.251	1.4707	0.8683	3.4469	1.1668
3.3623	0.8633	0.3697	0.944	0.7872	0.3528	1.0422	0.5379
2.5679	0.2993	0.6878	0.9468	1.1461	1.0273	0.905	0.406
1.4362	0.8728	0.8523	0.5365	0.4834	0.3405	3.3348	0.769
2.0476	0.1753	0.1635	0.1551	0.234	0.4872	1.4243	0.3511
3.0064	1.6189	0.958	1.3313	0.8071	0.5973	2.3487	0.655
7.0236	4.4777	1.2721	0.8811	1.8404	1.6953	1.0608	1.8294
44.0084	4.6045	5.102	9.817	8.571	17.1589	9.0023	3.2694
0.7285	1.5999	1.4931	4.0511	1.5239	2.165	1.799	0.6144
0.3795	0.909	0.0729	0.2302	0.1155	0.0244	0.2302	2.6684
8.7931	1.1743	0.4042	0.7323	2.9413	0.5119	2.1818	1.1506
82.1041	1.393	0.4176	2.234	2.1131	2.0982	1.4869	0.4725
6.6976	0.8145	1.2092	1.6624	2.2078	1.5121	2.8565	1.1953
2.5558	1.2945	1.7375	1.3802	3.3128	1.2317	2.1997	2.5277
10.3936	10.4959	8.0508	5.9559	6.6717	4.1915	11.6797	7.6395
2.9013	16.1299	2.9668	2.928	11.7832	15.7863	7.7587	13.7385
1.1502	0.5446	0.0667	0.0245	0.0744	0	0.4085	0.0078
2.2046	1.6107	0.7796	1.7081	2.1687	4.3507	3.1453	1.7439
1.7283	0.9208	0.6594	1.1668	0.5276	0.1975	1.0238	0.3123
3.0132	7.8835	2.4211	2.2219	8.9555	3.272	6.8874	1.7356
2.9096	2.8728	0.7376	3.7579	1.1767	1.8864	0.7357	1.4653
2.1015	0.4508	0.7507	1.0915	1.554	0.9102	1.3287	0.6681
3.2279	1.5626	0.5552	1.3821	2.3877	0.596	1.4208	1.0432
0.5605	1.0356	3.5867	0.2526	0.808	0.2428	0.2606	2.475
0.0671	0.1814	0	0.0702	0.0109	0	0.1279	0.0242
1.3975	0.315	0.8861	1.5302	0.0863	0.0966	0.3788	0.13
0.8893	1.1831	0.649	0.6629	1.0455	0.3918	0.6351	1.259
1.3212	0.1215	0.2428	0.248	0.2291	0.4965	1.1797	0.2551
1.1459	0.7414	2.2025	3.9141	0.0968	0.4393	1.2365	0.6781
0.7211	0.4735	0.5958	0.0716	0.5953	0.172	1.0452	0.238

1.7671	1.303	5.5673	4.8607	4.1064	0.3305	1.5187	0.5661
2.8093	2.131	0.9537	2.8801	0.4655	1.0692	1.0099	0.2472
0.1046	0.5501	1.5322	0.2389	0.1223	0.2009	0.2395	0.3063
1.6041	0.0561	0.056	0.0572	0.6346	0.5501	0	0.1087
1.3753	0.2564	0.1537	0.1047	0.625	0.3144	0.2737	0.6462
16.4168	4.0256	9.8442	9.1636	3.7845	2.9208	9.3645	3.9775
0.4718	0.2639	1.0346	0	0.4064	0.2738	0.0181	0.3935
0.0814	0.3869	0.6823	0.0929	0.317	0.3349	0.162	0.7059
0.7355	0.4114	1.1252	1.4144	0.5278	0.7965	1.3483	1.1333
0.6616	0.4582	0.4755	0.7614	0.2966	0.4177	0.2613	1.0988
5.2625	0.5462	0.5316	0.3982	1.5559	0.4609	0.3849	0.2681
5.3847	2.0195	2.0009	2.5501	1.296	1.6996	1.8268	3.5163
4.3539	1.79	1.9743	4.7117	1.5069	1.1272	4.8809	0.8152
0.4971	0.2383	1.0519	0.5473	0.4495	0.7305	1.431	0.5197
2.7502	2.7514	4.5526	4.8611	4.6867	3.6997	4.9737	6.4798
0.37	1.6097	0.2528	0.2347	0.6305	1.0573	2.2807	0.7466
0.8091	0.5598	0.1652	0.3609	0.2843	0.1907	1.5973	0.3601
3.2791	0.3335	0.1666	0.3971	0.387	0.2385	1.7552	0.1616
0.7657	0.8852	0.3424	1.2531	0.1491	0.6652	2.8448	1.0793
0.9484	1.1086	1.4005	1.8668	0.7581	1.1067	2.0426	1.5348
2.298	1.7001	1.1187	1.6928	0.3609	0.6101	3.1352	1.1654
10.362	4.5442	2.5356	7.6346	3.5218	4.7541	3.7814	3.758
7.894	3.6686	5.9738	5.824	7.155	22.8633	1.4201	13.3653
5.3973	0.4533	0.4577	7.0259	0.7282	1.3236	0.158	0.1857
4.262	0.0568	0.0851	0.5504	0.0247	0.0232	0.2272	0.1375
2.2631	0.4914	0.774	0.5695	1.1671	0.5208	1.8225	0.3874
3.5384	1.1008	0.8144	1.2531	0.4791	0.6878	2.1655	1.2617
1.9004	0.1746	0.2554	0.6122	0.3018	0.2824	0.4188	0.493
6.732	1.4926	1.0096	1.617	1.6389	0.9572	1.9608	1.2462
7.993	1.5171	1.424	1.7169	2.257	2.8204	2.2981	1.8613
9.2105	9.2665	4.5528	5.0228	11.3453	8.2023	8.516	7.229
2.8899	0.8927	1.1331	0.8224	1.2013	0.5975	1.3177	1.3001
0.254	1.3141	1.6325	0.1812	0.0309	0.0871	0.1264	0.3098
0.4001	1.0444	0	0	0.1623	0.2287	1.1612	0.4699
0.3903	0.3135	0.0839	0.2	0.9599	0.1853	0.9561	0.8735
5.4305	0.3822	0.5227	0.349	0.5779	0.518	0.859	0.4094
11.2567	1.0663	0.5227	0.5749	0.823	0.9866	0.1611	1.0723
2.0223	0.4427	0.6881	1.6564	0.8134	0.7236	0.7	0.286
6.4051	0.4343	0.3038	0.3103	0.6993	0.6922	1.101	0.3367
6.3345	0.4252	0.1416	1.1572	0.3701	1.0426	1.0086	0.1374
1.9909	0.4002	0.6955	0.7991	0.515	0.5547	0.7894	0.4047
1.8485	0.9095	0.971	1.0235	1.9844	0.7026	1.2023	0.652
8.844	0.6372	0.7865	1.1857	0.8156	0.6433	0.5334	0.3632
2.8673	0.3901	0.5631	0.6636	1.4212	0.6555	0.8483	0.3781
4.9105	0.3869	0.5799	0.7107	0.2694	0.5218	0.7915	0.15
1.8619	0.3787	0.3784	0.5154	0.4121	0.1935	0.3369	0.7647
2.0828	0.8474	0.5292	0.5189	0.9219	0.5973	0.8291	0.5338
5.5953	1.9052	0.8547	1.0714	0.8122	1.9068	3.9427	0.9797
5.6072	0.9257	1.2155	2.3345	0.839	3.3297	3.5862	2.0241
4.8289	0.4107	0.0158	0.4514	1.3337	0.7553	3.836	0.7348
5.1244	0.4894	0.4192	0.999	0.7911	0.5143	1.4926	0.271

1.0391	0.5296	1.3037	0.8964	0.3823	0.2534	1.781	0.0376
5.581	1.0159	1.0584	1.0937	0.6416	0.7746	2.2646	1.1106
1.8933	0.1426	0.3867	0.4573	0.7091	0.2372	0.2899	0.2566
2.1412	0.9316	0.757	0.3552	0.6772	0.8033	1.8303	1.508
9.0992	0.1271	0.1792	0.6255	0.3643	0.0183	0.831	0.1666
7.1598	0.751	1.0005	2.4817	1.8364	2.499	1.495	0.7971
1.5927	0.2227	1.0015	0.7956	0.2908	0.5461	0.6934	0.2159
3.6278	1.2869	1.7311	1.97	1.2709	1.3654	1.651	0.9594
1.1921	0.5081	0.5711	0.4213	1.5202	0.4282	1.6382	1.3233
1.2194	0.6821	0.9737	0.895	1.5267	0.5973	0.7801	0.6611
2.1481	1.8038	0.9134	1.7855	1.74	0.2727	1.5442	0.276
6.5663	2.1394	0.1562	2.991	2.6019	0.1916	0.0348	3.8722
2.0508	1.1087	3.7929	4.416	0.9985	0.6041	1.0615	0.4029
2.4715	1.2653	2.0709	1.7725	2.569	1.7668	4.9261	2.7726
1.5912	0.4402	0.2248	0.6788	0.4257	0.1079	0.6264	0
1.4265	0.7059	0.1533	0.4072	0.3473	0.0753	0.9282	0.1487
1.4294	0.5176	0.4403	0.5626	0.6569	0.4322	1.3469	0.4302
0.8213	0.603	0.9755	0.7326	0.7248	0.352	1.3536	0.167
2.1207	0.5762	0.1693	1.1761	0.531	0.7064	0.9346	0.427
1.7753	0.6786	0.3473	0.9628	1.1381	0.487	1.1631	0.5454
0.1021	0.2284	0.2283	0.9326	0.2983	0.4901	1.4732	0.1107
1.7901	0.7476	0.412	0.4994	0.5679	0.811	1.2776	0.9397
2.3785	0.8009	0.5163	0.8569	0.416	0.1742	0.7124	0.3255
5.0204	0.3874	0.0968	0.7248	0.3934	0.3166	0.201	0.3754
2.1632	0.7447	0.2325	0.3325	1.0533	0.6277	0.621	0.4059
2.7334	1.2974	0.7408	1.3477	0.7461	0.3976	1.2776	0.9879
1.9446	0.3626	0.8454	0.6661	1.8094	0.4445	2.0641	1.2885
7.4687	1.2137	0.6297	1.8342	1.2596	1.2591	0.7682	0.6786
2.1588	0.2818	0.1877	0.671	0.4788	0.2632	1.3009	0.1951
9.1107	33.3726	20.2005	9.1503	10.1863	23.3501	16.2359	14.8174
7.8971	1.0683	0.4616	0.7661	0.3016	0.1062	1.5666	0.4757
5.896	2.7394	2.356	1.836	4.2231	16.6589	5.1886	0.951
2.7231	0.4032	0.4029	0.2286	1.2479	1.8676	1.076	0.6513
0.7402	0.8282	0.4498	0.2389	1.5044	0.309	2.6264	1.1167
4.0795	1.7791	0.6676	1.785	2.0451	3.2488	4.3802	0.9385
3.7665	0.2784	0.6015	0.4915	0.3406	0.7196	1.7403	0.7657
1.1913	0.1609	1.0907	3.7288	0.11	0.1127	0.2862	0.824
2.5053	1.1679	0.2334	0.447	0.8132	0.179	0.9609	0.1415
4.1234	0.7007	0.5835	0.596	0.6608	0.5727	0.935	0.3396
0.3118	0.3837	1.0107	1.4951	0.334	0.4276	1.6754	0.9127
3.7623	0.6872	0.9442	1.315	0.8786	1.0795	1.2035	0.6868
4.8209	1.2437	2.1191	1.1891	1.8117	1.4445	3.7379	1.4844
10.9657	7.396	1.8725	6.12	1.2505	3.1552	2.7337	3.7291
2.1321	0.7021	0.2114	0.6675	0.5442	0.2241	0.5048	0.1585
2.8942	2.6396	2.919	1.2931	0.7352	0.6041	2.4419	2.7628
1.3864	6.7661	2.0844	0.1092	0.3026	0.0984	1.2608	3.162
1.737	0.949	2.7321	3.1824	2.6748	0.8865	0.7638	0.4599
3.3238	1.7473	0.3955	4.801	14.6172	1.2542	2.7365	1.2736
1.1728	0.849	0.1928	0.2954	0.8902	0.5441	0.7209	0.3366
1.6024	0.3667	0.3325	0.8039	0.5497	0.383	1.3228	0.2698
12.8668	4.9291	2.4935	4.4621	3.6979	2.756	6.7324	3.4377

5.6545	0.3919	0.6153	0.3999	1.0964	0.4805	2.2409	0.4612
1.8581	0.1039	0.4501	0.1768	1.6888	0.0425	1.4793	0.235
1.0252	0.5735	0.573	0.3292	0.6551	0.5713	0.5739	0.6947
3.4637	1.5853	4.0855	4.0583	1.719	2.5325	3.1811	1.9192
2.4156	0.5847	0.6102	0.4906	0.6333	0.7009	1.1557	0.4281
2.1615	0.4525	1.081	0.4979	0.9786	1.4564	0.6919	0.7469
7.7422	11.3312	7.1362	12.5563	22.1256	5.5482	12.0602	10.0391
2.8441	0.8191	0.6889	1.3831	0.5437	0.3612	0.76	0.3426
3.9396	2.5827	4.0864	2.3264	2.5351	1.412	2.7044	6.6982
4.3111	4.7952	1.9895	4.2355	5.5406	1.6502	4.2653	2.31
1.2723	0.978	0.9158	1.4161	0.5749	0.7345	1.7808	1.0718
2.3671	3.0768	1.9718	4.8888	10.0566	10.6956	3.5335	1.5601
0.0269	0.0301	0.4133	0.0077	0.4124	0.71	1.251	0.0656
1.403	1.6147	0.0828	0.1231	1.9645	0.5809	0.2585	1.9803
3.6863	2.0621	1.759	2.6178	1.5322	1.4182	1.2079	4.0943
0.124	0.1441	0.4	0.2833	2.7365	0.9685	1.6475	2.633
0.5776	1.5028	0.5256	0.4908	2.8515	1.8885	1.564	0.9539
2.9483	0.8718	0.7903	1.0113	0.6454	0.2507	2.1145	0.3593
44.1877	3.4028	2.1199	12.2778	11.0145	5.6917	10.9379	5.6441
0.9814	0.488	0.1795	0.2144	0.7728	0.1828	0.0904	0.4171
0.2107	1.2791	0.3637	0.0991	1.7077	1.3174	1.1285	0.2284
0.3027	0.8175	0.3851	0.2145	0.4778	0.3866	0.3792	0.9394
0.0523	0.1755	0	0.3582	0.0255	0	0	0
1.2954	0.9637	0.0224	0.0839	0.7803	0.055	0.0598	0.2389
5.3122	0.583	0.1879	0.4031	0.753	0.4381	0.435	0.3463
1.7116	0.9445	1.4739	1.2016	1.3177	1.0311	2.2788	0.8527
1.7883	0.0186	0.4231	9.1977	0.2268	1.2721	3.8862	0.0316
6.9058	0.0522	1.8605	0.0533	0.0303	0.1493	0.0774	0.1518
8.053	1.3192	1.1639	0.487	0.5497	0.4129	0.7739	1.0608
0.8286	0.4256	0.5937	1.273	1.4194	0.6768	1.1207	0.4451
0.9821	1.0609	1.0601	1.0247	1.1378	0.5807	0.9605	0.6059
2.3557	0.7522	0.4486	0.6189	0.3756	0.3217	0.7417	0.5086
0.3316	0.8719	0.8527	0.2461	0.8719	0.1933	0.792	0.2876
0.3477	1.0697	0.4211	1.9518	0.5643	0.8743	1.557	0.4712
0.4327	0.0127	1.6292	0.598	0.377	0.1406	0.6005	1.4937
1.2342	0.5466	0.4312	0.3523	0.3505	0.5996	0.5118	0.4739
1.2176	0.4886	1.6719	0.7253	1.1083	0.4175	0.619	0.4735
0.0505	0.1623	0.134	1.2678	0.0614	0.1298	0.1318	0.4651
1.284	0.6316	0.5816	0.7457	0.7222	0.6832	1.6633	1.5122
1.397	0.7431	0.7809	0.1177	1.4496	1.2094	2.507	0.5587
4.6354	12.5301	0.9528	0.3415	0.5679	0.082	0.2678	0.2918
0.8762	0.3968	0.4431	0.5955	1.7471	0.4578	0.955	0.3393
1.5211	0.4755	0.9253	0.7151	0.3703	0.6136	1.3356	1.2854

TCGA-CV-7(	TCGA-CV-59	TCGA-CQ-53	TCGA-CR-64	TCGA-CR-73	TCGA-CR-73	TCGA-CV-7(	TCGA-CV-69
4.0289	6.2902	0.5564	0.5803	2.1899	3.4582	0.3347	3.6427
2.4526	3.9878	1.7482	1.8437	2.9375	1.4245	2.0471	2.9021
0.8212	1.8145	1.8298	2.407	1.3962	0.903	0.9094	0.8485
0.9132	2.04	1.8269	1.0568	0.662	0.7833	0.9285	0.977
0.7251	0.4935	1.0663	1.0046	1.4566	0.5387	1.9959	0.4143
1.7228	2.5046	0.9749	1.4768	1.2404	1.8473	0.7647	2.139
0.5004	0.7195	0.9357	0.6141	0.7955	0.5133	0.5466	0.5132
0.5409	0.4204	2.1582	0.912	0.8497	0.2721	1.7516	0.3185
0.6284	1.0364	0.297	0.0945	0.5049	0.4585	2.8665	0.4182
0.6678	0.7833	0.2162	0.4367	0.1801	0.4305	0.2688	0.7322
2.4516	6.0226	1.0191	1.9027	1.3342	3.1543	3.8735	1.1421
0.4099	0.3084	0.4338	0.2257	1.0225	0.4126	0.4939	0.271
1.1536	1.2503	1.6889	1.4671	0.6018	0.6405	0.5571	0.4366
0.2127	1.4666	1.6092	1.2628	2.8487	1.9043	1.1009	1.4949
1.0232	0.6328	1.2894	1.0154	0.9646	1.349	2.8273	0.9166
0.9321	1.7383	2.0745	1.644	1.0565	0.9331	1.2159	2.0304
4.1052	8.6654	2.0067	3.5809	1.2812	5.1032	2.8484	3.3835
1.7639	2.9613	2.536	1.1839	1.3876	0.9002	1.4143	1.2615
1.9202	1.4105	1.8268	0.5882	0.9778	1.6422	0.5126	1.2381
1.2657	1.155	2.0395	1.3875	1.0062	0.9281	2.2072	2.1089
8.5958	9.4679	17.4999	8.2192	3.5618	6.7086	16.269	8.3299
4.7172	3.1145	3.5982	2.7423	1.0319	3.7551	3.2587	7.191
1.803	7.1769	3.2959	1.6531	1.1971	2.3424	1.2088	1.9624
1.7079	1.0209	3.9215	2.005	1.2156	1.1655	1.1636	1.0475
1.756	2.9668	2.0598	2.0447	0.8956	1.9574	1.7366	1.8825
2.9505	2.6023	4.4211	3.6285	1.7616	1.8406	1.6578	1.8403
1.1591	0.1855	0.1215	0.3958	0.6977	0.73	1.1361	0.1209
6.4605	22.3912	8.7634	6.7098	2.5933	8.8435	6.8397	13.8346
0.31	1.387	0.4195	0.622	0.7391	0.9573	0.8504	0.7927
1.3506	2.4482	0.631	2.1267	1.4534	2.2328	1.229	0.4855
0.4899	0.9858	1.0777	0.945	0.3738	0.4605	0.4445	0.4327
0.0777	0.1106	1.8106	0	0.0144	0.103	0.0485	0.0343
0.6222	0.0536	2.1077	0.6455	0.0418	0.0999	1.4599	1.5483
1.0984	1.0467	1.5028	0.9985	1.1133	1.1815	1.3039	0.9507
0.9527	0.6363	1.7115	0.4725	0.9189	0.9444	1.3208	0.8743
1.6535	2.0342	1.8042	1.0317	1.47	0.7993	7.5908	1.4997
5.6696	16.0969	8.8563	2.6931	1.7167	5.5507	4.9481	5.1273
0.5722	1.4683	0.2613	0.6286	0.4727	0.5054	0.1121	0.6539
1.5095	1.1609	0.4203	0.6553	0.8707	1.0554	0.3066	0.4565
2.5944	2.6589	2.5353	2.7165	1.9248	1.5767	1.3714	2.8838
1.7455	1.1253	2.6596	0.4443	0.3273	0.4872	0.3414	0.3525
1.9308	0.1725	16.5437	5.5482	0.2765	0.1786	3.6529	0.2589
0.3922	0.5579	0.8563	0.2797	0.5073	0.2815	0.6939	0.4113
0.2529	0.3298	1.6321	0.287	0.4089	0.1396	0.5856	0.3071
3.6836	7.7463	8.7108	4.6457	4.2284	3.2341	4.5424	5.454
0.4515	0	3.0737	0	1.6943	0	1.8217	0.92
0.4788	0.5341	0.8636	1.5851	0.7598	0.2176	0.4455	0.2363
0.655	0.1242	0.7797	1.9481	0.5971	0.0579	0.7271	0.0386
0.5015	1.241	0.0061	0.6098	0.6169	1.104	0.8613	1.0103
12.3927	14.6906	29.4698	13.2982	11.9434	16.0643	11.8917	17.1322

0.2869	0.3557	0.0685	0.1145	0.1631	0.4093	1.1575	0
1.2148	2.731	0.4771	0.6821	0.861	0.8395	0.476	1.0728
1.1756	1.0779	1.3841	2.1769	2.2559	1.2859	1.9155	1.2925
3.7621	1.7838	4.8679	3.4368	1.3511	2.1003	2.3395	3.2073
2.2372	5.5108	3.6432	3.1138	1.3714	1.229	2.1126	1.542
1.6243	0.613	2.625	0.2064	0.098	0.4099	0.5244	0.1756
0.2907	0.2637	0.2355	0.236	0.1962	0.1228	0.642	0.2009
0.209	0.5506	0.4327	0.4578	0.2403	0.0821	0.6768	0.4512
0.679	0.8239	2.001	0.4072	1.3641	0.4546	2.9712	0.332
0.4452	0.1639	0.2359	0.2391	0.604	0.1897	1.3084	0.222
0.1379	0.0291	2.3308	0.6595	1.6248	0.0507	1.0267	0.1556
1.4193	1.9658	3.7531	3.8019	2.8295	3.5329	2.3133	1.7927
0.3915	1.0674	1.0638	0.1862	0.3979	0.3746	3.7351	0.1729
0.8188	0.1792	0.1956	0.1307	0.9777	0.5563	0.8391	0.6674
0.8546	1.3054	1.9601	1.3165	2.2231	1.4867	2.974	0.2535
2.359	0.8315	2.8849	2.2526	6.3578	0.4979	13.5232	1.1799
0	0.0162	0	0.0177	0.0252	0.0151	0.0284	0
0.0317	0.015	0	0.3457	0	0	5.5354	0
0.2226	0.6263	1.1482	0.9609	0.8968	0.4249	0.5679	0.6086
0.0438	0	0.1361	0.1364	0.1296	0.1548	1.3865	0
0.7721	0.9445	0.9351	1.7262	0.7191	1.057	1.2471	0.9341
0	0.1432	1.1727	0.2351	0.2791	0	4.0876	0
0.3335	1.0643	1.0418	1.1434	0.508	0.4597	1.0733	0.2729
1.3006	2.2024	1.2982	2.2891	2.7811	2.1744	1.1022	0.9844
0.4588	0.9063	0.5046	0.8925	0.3109	0.4052	0.2387	0.4389
0.5848	1.6305	0.1816	0.5764	2.1226	0.6147	0.8229	0.8366
0.8848	0.6122	0.9654	0.3721	1.361	0.0211	0.2588	0.3801
0.3729	0.4382	0.3902	0.4289	0.1019	0.2076	0.27	0.7731
0.7379	0.4249	0.4706	0.2051	0.2045	0.7798	0.3621	0.4887
1.1802	0.1148	0.7518	0.4395	0.9395	0.5613	1.0834	0.2138
0.0347	0.0164	0.0538	0.2338	0.0512	0	8.37	0.0612
2.2521	0.6893	1.4353	2.0663	1.1771	0.4491	0.1294	0.1855
0.7698	0.6314	0.2261	0.2806	0.7997	1.2313	1.2473	0.6614
1.0671	0.4182	1.4665	1.6161	2.2885	1.391	0.781	0.7334
1.7399	1.3553	1.4473	0.3224	1.0794	1.3996	2.2507	1.2072
1.4037	1.3238	0.8622	1.2483	1.6533	0.5859	3.1338	0.6266
3.0247	0.4097	19.84	8.1335	9.0799	1.8537	1.4391	0.8722
0.1276	0	0	0	0	0.0376	2.0178	0
1.131	0.7967	2.0571	0.5029	0.1911	0.2283	2.9595	0.1712
0.8201	1.5775	2.6739	1.3856	0.7187	0.3932	2.4971	0.4345
0.3166	0.1501	0.6759	0.3695	0.4388	0.1573	0.7414	0.2097
0.9719	1.1575	3.7904	0.9615	1.2865	0.579	1.2421	0.519
1.0929	1.3046	1.6464	1.6191	2.8336	0.6868	1.7747	1.0468
1.8093	2.3096	1.6928	2.4307	2.6405	1.3932	3.8627	1.1471
1.2911	0.6932	0.6785	0.474	0.3524	1.5544	0.7475	1.2629
8.4298	11.583	6.7671	5.7712	4.0905	9.7756	9.9553	6.0262
0.876	0.2186	1.4317	0.7175	1.1587	0.5294	1.8426	0.4886
0.0366	1.8232	0.7184	3.1165	0.1106	1.3717	0.9167	0.0512
8.448	7.8054	8.3201	8.5741	5.2335	8.1742	6.9672	4.4986
2.9365	7.1867	4.2646	1.3519	2.7322	3.1339	1.5056	2.7508
0.7001	1.4427	15.5954	1.1454	7.4741	1.2368	2.9483	1.2841

0.2524	0.1047	0.0735	0.2782	0.2915	0.5015	0.7092	0.3621
4.3284	6.7894	3.1966	3.7265	3.0994	1.6003	1.4489	3.8118
1.0945	0.7488	1.9363	0.6949	1.4934	0.9427	1.7819	0.5771
1.1484	0.2723	0.1274	0.6809	0.091	0.3623	1.127	2.8251
2.2907	3.3117	3.0053	2.6407	1.8901	2.1226	2.1537	2.2371
0.4779	2.9054	1.0038	0.7582	0.6441	1.3158	1.7319	0.3972
13.0352	15.7197	14.4068	10.813	12.3753	11.8184	9.0609	18.9624
0.0333	0	0.3102	0.6564	0.2707	0.0294	1.9684	0
0	0	1.094	0.0541	0.135	0	0.0435	0
1.3279	0.8582	0.7383	1.4172	0.4951	1.1276	1.1198	0.7771
1.5722	0.0452	5.6219	3.2623	6.7261	2.2723	3.57	0.5048
5.0464	1.7402	3.4429	4.3631	0.7913	4.9975	1.2733	5.4016
1.4446	0.9035	1.2647	0.6856	0.7497	0.8687	0.7422	0.6786
0.6589	0.0417	0.0682	0.319	0.0974	0.0388	0.1829	0.1552
0.8056	1.3847	1.4855	0.6791	1.3771	0.4892	2.0124	0.8893
1.2007	1.4982	0.9813	1.7048	0.327	3.9073	1.105	1.5626
0.2945	0.5305	0.6858	0.2749	0.1632	0.052	1.3116	0.182
0.8516	0.8918	1.2993	0.7068	0.3394	1.0531	0.7276	0.9155
0.2626	0.1868	0.1019	0.2725	0.1456	0.058	0.328	0.116
0.2988	1.0046	0.2109	1.1273	0.1205	0.3119	1.4475	0.1919
0.4147	2.0084	0.1495	0.7453	0.8868	0.5429	2.0532	0.0392
0.4415	3.0649	0.3839	0.2016	0.1436	3.0107	0.5294	1.0606
2.5079	1.0417	0.092	2.1513	0.427	0.9332	0.1911	2.2844
1.1132	1.0557	1.8503	0.4555	0.8808	1.5095	3.094	0.1938
1.0851	1.848	1.7537	0.1608	0.4092	0.9389	0.6085	0.5085
0.5086	0.3858	1.0003	0.3166	0.2506	0.0599	0.367	0.4192
1.5967	0.8314	0.1408	0.1686	0.2277	2.8556	0.0441	0.3404
0.5598	0.0332	0.652	0.9076	0.2069	0.2163	1.4858	0.4326
0.3452	0.8631	0.0487	0.8792	0.1856	0.2772	8.4667	1.164
1.9691	0.9336	5.3309	1.3096	2.034	1.7615	3.321	1.0701
1.1729	2.1923	1.2669	1.2698	0.7539	0.7958	0.5945	1.4711
4.3768	2.9825	3.9802	2.1125	3.4839	2.0915	3.3642	2.0228
0.1961	2.085	0.1522	0.5993	0.3416	0.4267	1.1514	1.552
3.417	1.8733	1.2435	3.656	1.3418	1.6034	1.6893	3.3004
0.229	0.0362	0.489	0.1584	0.2187	0.1686	1.0647	0.2528
0.3451	0	0.1236	0	0.8829	0	0.1989	0.0469
0.5436	0.0469	1.2276	2.3584	0.7305	0.3492	0.7406	0.2618
0.3851	1.6737	0.1495	0.9988	0.7353	0.4535	0.668	0.9068
1.5457	0.546	1.2632	0.9025	0.0046	1.4205	0.4637	2.4758
3.4562	2.4138	5.2536	9.4493	11.122	5.3895	4.1218	1.8233
1.2047	0.9987	1.3025	1.2	0.519	0.7376	0.5969	0.62
0.4151	0.3249	1.0435	0.7041	0.5796	0.3201	0.4334	0.3956
21.3365	49.1698	13.6497	16.4017	10.7713	20.3983	11.7607	20.1837
0.6396	0.5044	1.1799	0.5125	0.4447	0.5705	1.4922	0.7829
0.2548	0.1736	1.343	0.2271	0.7879	0.6498	0.2988	0.3676
0.5823	0.3137	0.8836	1.016	1.839	0.7013	0.595	0.5609
1.553	0.3416	3.356	0.6977	6.58	2.3121	1.9996	2.149
1.3938	2.0688	0.7214	0.6204	0.875	0.7779	1.1975	1.3557
0.6519	1.3189	1.2485	0.5749	0.9357	1.5019	0.9635	1.4776
0.7233	0.6254	1.3378	0.7891	0.7057	0.7422	0.7041	0.4227
0.7258	1.0324	1.6904	0.9789	1.2071	0.7693	1.3295	1.0255

1.6518	3.0592	3.1845	1.9668	1.9339	1.9681	2.6214	1.6447
1.1701	0.8742	1.3213	0.7909	1.0877	0.6733	1.8009	1.409
2.3009	1.6123	0.1897	0.4014	1.475	0.6384	2.916	2.769
1.1804	3.5238	1.697	0.6804	1.131	1.6218	4.55	1.3126
3.363	0.0102	1.6793	5.9065	1.754	5.2707	1.2231	5.8255
0.4152	0.7546	0.8596	0.5385	0.9719	0.3973	1.7861	0.0611
0.3891	0.369	0.6042	0.656	0.6472	0.2148	0.8504	0.6013
6.6417	0.1754	0.0392	0.0087	0.0932	0.2153	1.085	0.1039
0.8566	0.4283	0.0629	0.2779	0.3154	1.1555	0.555	0.2145
1.638	1.0656	1.1682	1.0177	2.851	1.0598	7.8976	1.8333
2.0515	3.585	2.9391	2.7429	4.5611	3.1196	1.805	1.0926
3.0002	8.1745	3.3134	2.4463	2.5987	2.7134	1.6712	3.8957
1.1294	0.4222	0.8093	2.4448	0.5057	0.1247	0.6329	0.6521
8.163	14.703	4.3299	2.4789	8.4594	11.8232	4.7402	13.8198
0.2595	0.4922	1.2088	0.1615	0.7673	0.4584	0.5618	0.275
0.1946	0	0.9566	0.7401	0.3835	0.5728	0.8369	0.3436
0.2504	0.1425	0.1166	0.4936	0.2406	0.2654	0.2919	0.7518
0.5734	0.426	0.7802	1.9013	2.4252	0.9607	3.9672	0.6264
0.3886	1.808	0.9994	0.6174	1.544	0.6436	1.6178	0.4933
1.3037	1.5802	1.015	1.6858	1.2164	1.1298	1.7178	1.0545
5.4647	0.1727	0.2828	7.1816	0	1.1262	0.3033	0.9651
2.7418	1.8532	1.6757	2.2091	0.9055	2.4217	1.4571	6.0787
1.665	0.0718	0.4701	0.5104	0.6993	0.1003	0.8507	0.9357
0.5831	0.2011	0.8642	2.9149	1.6457	0.1639	0.971	0.3277
0.0937	3.2147	0.3739	0.0338	0.0433	1.0318	0.0411	0.2688
0.3706	0.2812	0.3453	1.1023	0.7488	0.0873	0.5966	0.3491
0.9831	1.4294	1.1194	1.4959	0.4966	0.8972	0.7776	2.0256
7.6437	3.5461	3.4327	2.5133	2.6425	2.3058	3.4857	4.2369
1.4213	0.423	1.6752	0.7319	0.4141	1.2095	0.7342	0.1649
4.3008	4.8743	5.5109	4.2629	2.5303	3.445	7.5957	5.6484
0.9987	0.7845	0.8216	0.9743	1.201	1.4021	0.6578	1.4479
1.0658	1.078	1.2687	2.4695	2.521	0.5648	0.7691	1.0039
0.4105	0.1024	1.3754	0.7173	0.8304	0.5343	1.6549	0.1526
2.4668	0.9457	0.8965	1.2525	0.194	0.9272	2.2287	0.2781
1.214	3.4538	2.1582	0.4929	1.8598	0.7149	2.6223	0.4661
0.0989	0.3002	0.7066	0.3695	0.585	0.2097	0.7579	0.5591
0.6783	0.7634	0.6988	2.4006	1.0215	1.1534	0.4328	1.4555
1.3151	4.9244	5.0788	0.8397	4.1626	1.102	8.0298	0.6551
0.588	0.7248	0.9814	0.3508	0.239	0.1818	0.6854	0.5192
0.9234	1.7868	1.8988	0.4402	1.6049	0.9368	1.0701	0.6832
0.349	0.2801	0.1668	0.5571	0.2977	0.1423	0.1118	0
1.0988	0.2842	0.3878	0.3109	0.3692	0.397	1.7466	0.9703
6.2011	13.585	10.5733	5.3995	7.206	8.1599	7.1271	4.6619
1.2668	0.5606	0.6994	0.9492	0.6867	0.9822	0.5274	1.5662
2.5489	2.3323	4.652	1.7398	1.9006	2.2316	6.4784	1.4216
1.1447	1.4452	1.0202	1.4828	0.6008	0.5863	2.3405	1.2322
3.5037	2.9198	4.3686	0.7711	5.4151	2.3913	12.0666	4.0315
0.952	0.9552	2.1227	1.1542	0.4828	0.6648	0.5392	0.7429
0.5428	0.8711	1.4263	0.4332	2.3147	1.2907	0.4172	0.2212
2.1366	1.4625	2.7413	1.9592	2.5692	1.7964	1.5326	1.8824
0	0.3273	0	0.5313	0	1.7896	0.4078	0

1.4087	1.7839	0.8029	1.5077	1.8584	2.5435	2.6575	1.5746
0.5652	0.469	1.2615	0.623	1.2272	0.1872	1.0588	0.3119
7.4649	0.343	0	2.0812	0.4619	0.0581	3.0392	7.8407
1.1073	2.8083	0.7797	1.1622	1.3515	1.5581	1.5116	1.6146
0.8671	0	1.3463	0.7068	3.754	0.7658	0.6188	0.2188
0.4352	0.1259	0.2176	0.2678	0.5016	0.0977	0.7124	0.2345
3.0421	0.0141	0.0693	8.481	0.0275	0	0.2415	0.0131
0.7038	0.1049	1.0616	0.2816	0.5574	0.3641	2.2853	0.6215
0.2923	0.154	0.1513	0.0337	0.6963	0.1435	0.0811	0.1721
0	0	0	0.78	0.0265	0.253	0.9538	0
2.0702	0.8016	2.0894	1.7541	2.3209	1.3866	2.4993	1.5539
0.1032	0	0.1603	0	0.0381	0	0.1289	0
2.6965	0.6764	3.4576	0.7581	1.2602	0.2612	6.0113	1.6285
0.0666	0.1263	0.2068	0.4146	0.3446	0	1.2753	0.1176
0.925	0.1907	0.6244	1.0014	1.1891	0.1776	6.5296	0.1421
0.2414	0.0572	1.2814	1.3605	0.223	0.242	0.8433	0.0864
1.38	0.5745	1.8815	1.6763	2.5378	0.5946	1.1771	0.2972
1.939	2.4689	0.834	0	1.366	0	1.0536	2.5081
0.7315	0.5272	0.4543	0.5464	0.8219	0.1809	0.4872	0.1034
0.8455	1.5644	1.2488	0.4921	0.7926	0.51	3.7087	0.5463
0.502	0.1875	1.2636	0.3077	0.5847	0.2553	1.1968	0.5104
1.1093	0.8004	3.0141	1.2009	1.2388	1.459	2.8209	1.1286
0.5634	0.4646	0.8938	0.3939	0.3531	0.357	0.4895	0.3245
1.0699	0.4735	1.4951	0.925	0.4481	0.4725	5.8198	0.5669
0.4728	0.3699	2.6427	0.7726	0.9173	0.6994	0.492	0.2296
0.4457	0.2245	0.8002	0.4191	0.3295	0.1722	0.7886	0.369
0.9365	1.5275	1.8613	1.2437	1.0107	1.0091	2.9318	1.2239
2.2233	2.6375	1.3944	3.0214	0.999	1.186	1.4955	2.7872
7.5515	8.449	25.9813	3.551	19.1151	5.9981	49.486	3.2623
0.7195	1.0063	0.6982	0.1866	2.1538	0.4289	4.5527	0.54
0.1739	0.075	0.9084	0.8531	0.2747	0.1047	0.6122	2.4576
0.6219	0.6487	1.6899	0.6775	1.8044	1.6891	0.9968	0.6316
1.1976	0.3373	1.031	7.3565	0.6836	0.2723	3.0802	1.2774
2.1076	1.1045	5.7699	1.381	1.9269	1.2982	4.4332	1.1755
1.2663	0.4367	3.1459	1.1944	2.5698	0.8541	2.1087	1.6265
16.9711	18.7758	14.9485	6.7039	2.2634	14.3251	12.7693	9.212
11.1828	1.4864	9.422	11.4502	6.0912	1.344	5.8843	3.3025
1.473	0.006	0.0049	2.1456	1.3758	0.045	0.1642	1.6407
2.7143	2.4601	3.2365	3.3606	1.4188	0.6225	3.5335	1.7215
0.7498	1.3006	1.1074	0.4459	0.7367	0.5007	0.571	0.3876
3.7034	1.6072	4.7108	2.7352	4.0676	0.9425	5.418	1.0347
8.723	9.1262	2.5179	0.9127	5.7584	7.3395	5.3744	1.5911
0.7689	0.4694	1.9789	1.6173	1.3703	1.1722	1.6487	0.6325
1.1587	0.3139	2.2488	0.7299	2.3553	1.1696	1.7572	0.5847
1.2242	7.6323	0.9772	0.8672	0.8795	11.1475	0.3029	2.5877
0.0149	0	0.2777	11.2894	0.7933	0.0395	0.0186	0.0132
0.1425	0.4714	1.0471	1.3957	0.4034	0.1811	0.5731	0.1166
1.0843	0.3757	0.7297	0.4398	1.2783	2.5882	1.6291	0.2767
0.3378	0.2631	0.7306	0.4131	0.2497	0.1385	0.2913	0.277
2.3285	1.667	1.7406	1.5985	0.664	0.9706	1.2828	0.2731
0.4807	0.5351	0.6166	0.4337	0.9269	0.7753	0.2958	0.1107

0.2142	2.2849	3.9907	1.6666	3.1663	0.6621	1.5158	0.9457
0.7059	1.5434	1.157	1.3123	2.6598	0.381	1.0776	0.2251
0.2511	2.5794	0.3465	1.0275	0.3299	0.5298	0.4878	0.5666
0.6015	0.5704	0.6226	0.4161	0.6916	0.4723	0.3339	0.2361
0.9779	0.8114	0.6643	0.7609	1.4457	1.6735	0.6107	0.2159
5.425	3.1453	13.9373	5.5216	5.0697	2.5572	9.2602	4.468
0.2419	0.1147	3.1554	0.4518	1.1803	0.3419	0.4834	0.3845
0.3526	0.2058	0.5475	0.8442	0.3408	0.1677	0.2936	0.9581
0.8516	2.1535	0.8414	0.9638	0.1717	0.6382	1.4611	1.4584
0.8611	0.1261	0.25	0.3486	0.8952	0.4761	0.2914	2.1267
0.4311	0.2325	0.7613	0.3684	1.1435	0.1643	0.4786	0.3882
2.5902	2.2048	2.1851	2.9836	2.2312	0.8467	2.5812	1.9091
2.9374	3.1944	7.7282	1.6689	1.4044	1.7193	4.445	0.9676
1.0888	0.606	0.9555	1.4734	0.9448	0.0418	1.1824	0.7106
2.5032	2.3403	7.8829	4.3529	1.1206	2.3044	3.3758	1.868
1.151	0.8706	0.6808	1.0662	1.0736	0.4236	0.4792	0.605
0.7003	0.4356	0.9284	1.6606	0.5407	1.213	0.4174	0.3893
0.3974	0.2198	0.9771	0.8247	1.934	1.4041	0.5791	0.117
0.6466	0.2904	3.1703	0.9533	1.6853	0.7214	1.36	0.7212
0.5756	1.4228	1.5531	2.2909	0.7394	1.9003	0.8564	1.4166
0.8401	0.4217	4.4502	2.1533	2.3742	0.611	2.1395	0.5236
6.3882	6.0124	2.6424	4.654	5.3446	3.7192	5.2354	5.5719
5.1409	0.8445	16.2789	1.9319	30.3704	1.8592	3.5052	2.0018
0.5737	0.0634	0.9686	0.3294	0.3623	0.0394	1.1732	0.1672
1.1725	0.0321	1.5055	0.0936	0.2583	0.0996	0.1126	0.5177
0.7146	0.7059	2.9743	0.9268	0.8144	0.4471	1.4131	0.4733
1.2109	0.7536	3.1728	2.0546	1.3147	0.6573	1.9324	0.8688
0.4494	0.4319	0.9554	0.4913	0.5596	0.4769	0.6504	0.7138
2.2165	2.3613	1.0197	2.8106	2.3868	1.4743	2.4834	1.7398
4.0967	4.0208	1.9559	1.0239	2.943	2.1482	3.4765	2.9349
12.4859	11.0799	4.6956	7.2972	8.3318	4.4315	4.5018	9.5491
1.6677	1.7941	1.4108	1.2589	1.0117	1.1174	1.0773	2.0617
0.5502	3.8128	0.0657	0.0878	0.2503	1.7196	1.2334	0.299
2.1336	0.0843	0.4141	0.7379	2.4971	0.5496	0.3701	0.471
0.5604	0.7402	0.3626	0.173	0.2959	0.9605	0.7499	0.3299
0.3356	0.6138	0.7445	0.4726	0.1772	0.1694	0.2994	0.4234
0.6473	0.1591	0.8934	1.6168	1.1696	0.2965	0.5988	0.7198
0.8792	0.5558	0.455	0.4257	0.7798	0.4659	1.3664	1.3457
0.3105	0.2945	0.8036	0.8054	0.593	0.2286	1.2281	0.2742
0.563	0.267	0.9616	0.3505	0.2497	0.1492	1.547	0
0.5599	0.8849	1.2879	1.3769	0.7051	0.4029	0.9496	0.4761
1.1432	0.4012	1.8211	0.5545	0.7023	0.5376	1.3906	0.8653
1.1612	0.8047	1.8724	0.5561	0.4952	0.355	1.2644	0.3155
0.7404	0.5879	0.9625	0.8575	0.7128	0.4107	0.9463	0.517
0.6916	0.306	1.2886	0.5262	0.2726	0.2443	2.6871	0.4886
0.5642	1.2127	1.2264	1.0146	0.6672	0.7973	0.6263	1.3285
0.7068	0.7421	0.5488	0.8905	0.4665	0.6912	0.7987	0.9809
1.3437	1.5378	1.4389	0.9134	1.3015	0.7776	2.6041	1.3912
1.9419	0.4841	2.3074	0.9175	1.085	0.3382	2.7251	0.6602
0.9412	0.6605	2.806	1.6211	1.2663	0.1663	0.7523	0.532
0.8332	0.7111	0.5175	1.1238	0.4927	0.368	1.8037	1.0301

0.0924	1.4014	1.1951	0.8784	0.9558	0.7886	0.2307	1.3594
1.1147	1.932	1.7651	1.3939	1.7788	1.3953	2.7105	1.5514
0.9102	0.4718	1.2814	0.7806	0.5651	0.2144	0.9195	0.3001
1.6713	0.9255	1.5533	1.2278	1.2354	0.5603	1.2595	1.0989
0.9709	1.0051	0.4702	1.1829	0.2502	0.3147	0.9641	0.6292
2.429	1.8589	3.3084	2.3875	1.008	0.9033	6.1383	0.4516
0.2654	1.1327	2.6789	0.6885	0.3924	0.1172	0.7735	0.2344
1.1502	1.5382	1.6485	1.8053	1.3298	0.4689	2.0872	0.9376
1.7407	1.6148	2.2915	0.9815	1.0909	1.2701	0.4096	2.005
1.2774	2.753	0.9015	1.6867	0.8584	0.9231	1.3536	1.4357
0.6594	2.0169	1.4756	0.1979	1.1947	1.3647	3.2377	0.5138
7.113	5.2328	2.9102	3.0075	2.9602	7.5885	0.4847	0.0822
1.78	1.1311	0.5556	0.476	1.1055	1.2723	1.2529	1.183
2.6189	1.8513	3.0116	3.1947	1.3633	2.344	1.8158	2.3996
0.1399	0.4421	0.7601	0.2781	0.7582	0.0412	0.4561	0.4529
0.4023	0.8324	0.284	1.6317	0.4866	0.2907	0.3045	0.0646
0.3743	0.2173	0.7175	0.7251	0.6705	0.3019	1.2847	0.3373
1.1292	1.8495	1.3814	0.8875	0.607	1.1787	0.7407	0.8461
0.3231	0.3064	2.3832	0.5448	0.5673	0.3568	1.0425	0.2853
0.8284	0.7295	1.225	0.3888	0.5832	0.5575	1.0675	0.627
0.2722	0.1291	1.1624	0.4237	0.3018	0.1803	0.68	0
1.2959	1.2494	1.3498	0.7675	0.447	0.8632	1.6814	0.4864
0.5388	0.4379	0.9561	0.9742	0.7055	0.4759	1.6021	0.4622
0.4231	0.073	0.5973	0.2794	0.2843	0.068	0.7046	0.0679
0.9983	1.1571	1.464	0.5754	0.8199	0.4409	0.8312	1.4693
0.5522	0.2094	1.4146	0.401	0.3061	0.0732	2.2297	0.3413
1.1811	0.7102	2.0575	1.2851	1.0221	2.3408	0.6716	1.4245
1.4463	1.9782	1.5979	1.4717	1.1925	0.7616	2.8717	0.8842
0.3838	0.2578	0.8938	0.1327	0.4964	0.1412	0.7056	0.1694
19.9746	85.0883	14.2151	13.2559	7.2162	14.3247	17.4178	7.6674
1.2731	1.1094	1.763	0.9282	0.3815	0.8509	1.3465	0.6077
6.1392	0.6623	5.6236	2.8983	1.7167	0.459	1.3388	0.9466
1.6551	0.8607	2.8186	0.4985	0.7104	0.4716	1.3336	0.5658
0.4935	0.1221	1.3658	0.4007	0.9515	0.1706	2.0543	0.1137
2.1559	3.4695	1.8196	5.5873	1.7523	1.0035	2.9421	3.3178
0.7263	0.2041	1.5873	0.7164	0.7159	0.103	1.1349	0.6968
0.4245	0.5973	0.4039	0.3836	0.6781	0.653	1.5504	1.1366
0.4871	2.5406	1.6208	0.6137	0.3343	3.1346	0.6663	0.6145
0.6611	0.5279	2.107	0.6137	0.2829	0.0922	1.7961	0.0615
1.3717	0.9066	0.9681	1.1644	0.4301	0.4038	1.1075	1.248
0.7166	1.626	2.5432	1.5666	0.7378	0.2486	1.2998	0.678
0.8892	0.7545	3.803	2.0394	1.2223	0.8681	2.8316	0.6888
7.2057	12.5226	7.3279	10.071	5.1062	3.3926	5.7021	6.3369
0.4585	0.25	0.2136	0.4281	0.466	0.4657	1.0593	0.2227
5.1588	18.1362	2.7352	0.9138	2.9452	5.0751	2.1302	7.2593
0.3824	6.8905	0.3464	0.7274	0.7303	3.2934	0.4246	29.1
0.2423	2.6556	1.7561	1.1454	2.2094	0.5232	1.2555	0.7609
2.3314	0.3038	1.5613	0.5632	1.059	0.5109	2.8895	0.0471
0.207	0.4579	0.7141	0.6203	0.17	0.1625	0.651	0.4061
0.3965	0.2072	1.5329	0.848	0.3649	0.293	0.95	0.4859
3.7357	3.0064	4.6556	3.3913	4.4508	2.9522	4.7065	2.9516

0.2669	0.5694	1.2949	0.8306	0.8383	0.4714	2.2775	0.4124
1.2387	0.4698	1.2181	0.6854	1.7091	0.6564	3.3003	0.1458
1.2814	0.4455	0.5969	0.7977	0.2842	1.6599	1.6359	0.9052
3.2313	2.4429	3.4667	2.2372	2.3697	1.9158	2.4097	3.2017
0.5264	0.646	1.154	0.9478	0.515	0.7385	0.8122	0.5743
0.8258	1.2305	1.0598	1.39	0.6976	0.908	0.6174	1.2799
12.1971	43.8882	18.4152	7.0595	9.2961	34.9799	23.2226	12.2033
0.8005	0.7724	1.57	1.5226	0.955	0.6946	0.6957	1.0293
4.691	4.7675	5.2161	2.8368	4.0723	4.0386	3.0919	4.7946
2.1387	0.7922	3.5283	1.8722	3.804	1.1511	4.4796	1.7706
1.3424	2.1467	2.217	1.8043	0.6134	0.8731	0.95	1.6812
3.9914	0.1907	2.6423	2.8249	3.3506	1.0931	2.0738	1.4208
0.8156	0.0085	0.5149	0	0.0861	0	0.2761	0
2.0263	0.8787	1.4925	1.4447	2.4221	1.903	0.9243	1.2113
4.5551	3.9217	4.0017	3.7932	4.6075	3.9174	2.3455	6.3513
0.7442	1.6588	0.5728	2.5738	0.268	1.0394	0.2807	1.3256
1.9072	0.5944	2.155	1.1613	1.2179	1.4078	1.0512	0.8065
0.3079	0.5078	0.9064	0.8529	0.7501	0.3216	2.2651	0.3216
5.7108	2.3599	10.653	3.014	5.262	7.9777	9.0476	6.4614
0.424	0.6701	0.2508	1.9315	0.7104	0.5636	0.2589	1.6475
1.2103	0.2429	0.1668	0.3857	0.1832	0.1788	1.4617	0.1605
0.7168	1.9071	0.4862	0.6714	1.1728	0.461	0.8865	0.467
0.1394	0.6942	0.0541	0	0.0773	0	0.0871	0
0.0267	0	0.0553	1.1638	0.0066	0.0079	1.6901	0.2044
0.2465	0.2763	0.6264	0.5116	0.1822	0.1188	0.5972	0.2771
1.434	0.8481	2.8251	1.9037	1.664	0.9942	3.1839	1.3344
1.0646	0.0105	12.0787	0.9837	0.5738	0.1371	1.9482	1.7725
0.0207	0	0.0644	0.0645	0.092	0.0366	2.3309	0
0.7024	1.4273	0.7271	0.7634	1.0137	0.5465	0.8772	0.7384
1.4211	0.2762	0.6394	1.2973	1.1544	0.856	1.6892	0.2661
1.8061	2.933	0.9465	0.8198	0.7509	1.1765	0.9775	0.638
0.8338	1.2058	0.766	0.6416	0.6934	0.7426	0.5149	0.8345
0.5305	0.9433	0.7208	0.5848	0.4494	0.9567	0.7914	0.5466
1.3133	0	5.1581	0.1202	0.2284	0.6482	0.997	1.0233
0.4402	0.8061	0.3535	3.4018	0.864	1.5285	0.2907	5.3619
0.5142	0.4226	1.5436	0.2845	0.2787	0.2119	1.1132	0.8476
0.6176	1.7402	0.7671	1.4279	0.8608	2.4624	0.4701	1.2465
0.9841	4.1236	0.2612	0.0349	0.2052	4.0488	0.5673	0.7428
3.3057	7.2212	1.3061	3.69	0.9927	4.497	3.0965	2.8671
0.7634	0.2027	2.2283	0.3168	1.6363	0.2697	0.9152	1.9684
0.9569	1.9283	0.9596	0.2275	0.2505	4.6486	5.4776	1.0563
0.7232	0.211	1.7707	0.4329	0.5346	0.0246	1.0653	0.3439
1.9683	1.103	1.806	0.6498	1.0802	0.5795	0.8691	1.8435

TCGA-CN-47	TCGA-CV-72	TCGA-CV-75	TCGA-CR-73	TCGA-CN-47	TCGA-BA-55	TCGA-CV-72	TCGA-CQ-53
0.6182	2.4502	8.1636	3.1395	1.4833	0.2721	0.6336	0.6819
5.7538	2.9475	5.8594	1.5566	2.2934	2.2357	4.3749	7.0828
0.5731	0.7311	1.6367	0.6582	1.7381	1.0592	1.322	1.6454
2.7365	2.1503	1.5108	0.9683	1.9339	0.4842	2.1688	3.2167
0.734	2.2201	0.5718	1.3577	0.4309	0.3718	1.2946	1.8785
1.0681	2.1462	1.8935	0.5588	2.3771	1.1747	1.4938	1.8736
0.7407	1.1048	1.3347	0.8441	0.685	0.5552	1.6134	1.9854
0.6967	1.2352	1.0341	0.632	1.2752	0.3676	1.3202	2.3052
0.6182	3.0754	0.333	0.1346	0.1932	0.0972	1.5669	2.1922
0.4356	0.5635	0.8644	0.644	0.454	0.1492	0.9528	0.4646
2.3805	0.5794	1.2455	0.9985	0.7606	4.0437	2.7398	3.1912
0.3109	0.2125	0.7963	0.3567	0.2499	0.6152	0.4306	0.5179
0.8192	0.3013	1.2547	0.1686	0.6893	0.8062	0.4739	0.51
0.1543	1.4191	3.2021	2.2426	1.1557	0.2736	1.0742	4.1475
0.5245	1.4161	1.6583	1.3654	0.779	0.3957	1.1115	1.3924
0.6237	1.6744	2.1829	1.1303	3.5156	0.8844	5.3453	1.4793
1.9041	4.7192	1.4279	4.2002	2.4117	2.6372	3.4565	3.0364
1.3448	0.4308	2.6696	0.1386	1.4785	1.6077	0.9473	1.5091
1.2412	1.5718	1.7742	1.1088	1.3857	0.7211	1.5281	1.8623
0.5936	1.4173	1.3525	1.1946	1.1581	1.1258	2.3478	2.5267
4.0208	7.1172	7.8163	8.0774	3.7955	4.849	9.5091	8.4248
7.1433	1.0146	5.5292	2.2724	4.383	1.1893	13.2039	6.9931
1.4781	1.4873	1.3714	1.1261	1.6891	1.0862	2.0674	2.4676
0.7198	1.6327	1.0265	0.952	1.8656	0.8025	2.0095	1.85
0.7844	0.7532	2.0945	1.2117	1.4332	0.7366	2.9494	0.3549
1.9231	1.9142	2.1226	2.1697	3.6062	3.0374	6.4654	2.797
0.4161	0.5405	0.5119	0.4503	0.3755	0.4133	0.3305	0.3299
6.0025	5.2277	5.5855	7.6614	5.6035	4.8303	6.3491	1.0866
0.6492	0.4589	1.6635	0.5481	0.7481	0.7956	0.5714	0.5665
0.4113	0.0204	3.0847	1.4878	1.1773	1.6	3.5764	5.2875
0.5014	0.8205	1.6706	0.307	0.8123	0.5168	2.416	0.947
0.2601	0	0	0	0.3142	0.0173	0.5254	1.388
2.5683	2.6043	0.0203	0.2893	1.2857	0.1006	1.3206	2.9173
0.473	1.7137	1.8087	0.9581	1.9392	0.2757	4.4301	3.3367
0.448	1.0851	3.2002	1.043	1.3092	0.5108	1.852	1.6149
1.6234	1.8215	1.0412	1.3082	1.0723	1.8882	2.2104	1.9209
2.9712	6.1974	6.0478	1.2813	3.14	2.4304	4.239	4.3363
0.3861	0	0.5067	1.8367	0.9862	0.1497	1.9027	0.7122
1.4127	1.1462	0.3671	1.2946	0.9929	0.3449	1.2137	0.8353
1.0649	3.7929	4.6208	1.1689	2.0969	2.3416	4.0656	2.1595
1.398	2.2898	2.3492	0.3548	0.8469	0.2937	0.6466	6.3831
1.7443	12.2538	3.022	0.7355	3.0077	0.6519	1.1428	3.7701
0.4921	1.2541	0.5272	0.2508	0.5324	0.1853	0.9789	0.6808
0.1133	0.0863	0.102	0.3144	1.1258	0.0844	4.4091	4.3898
5.3841	4.7799	4.2221	4.1876	4.8729	4.5911	5.918	4.016
3.2204	3.8142	0.0249	0	1.6841	0	0.7682	0.8267
0.5452	0.4178	0.7521	0.5843	0.313	0.4572	1.0209	2.0764
0.6261	0.9085	0.0626	0.2234	0.6617	0.0583	0.8585	0.5197
0.4233	0.8606	0.0281	0.1425	0.5621	0.4531	0.6933	0.7203
12.0224	11.0294	35.991	13.7034	16.6838	7.0595	20.6513	14.1998

0.1266	2.8149	0.348	0.1254	0.2006	2.532	0.1085	0.9047
0.3142	0.3022	0.4252	0.8043	0.5848	0.6992	0.8957	0.4442
1.5369	5.9031	1.9032	1.7701	2.8001	1.2707	2.4827	2.605
4.0149	4.2853	4.8937	3.1202	3.8785	1.489	3.6054	6.1646
3.182	1.9914	1.1931	0.1861	2.0122	1.0193	1.3411	1.2268
0.6813	2.5326	0.7605	0.1884	0.5861	0.1327	0.9776	1.381
0.5075	0.7243	0.7521	0.4454	0.4597	0.1349	0.9164	3.1092
0.222	0.1901	0.5827	0.0792	0.4927	0.1446	0.5708	3.6244
2.4359	3.0095	1.1489	0.1681	1.3112	0.9271	5.6502	5.096
0.2904	0.4181	1.8438	1.1374	0.7091	0.1724	0.4055	0.2633
1.2337	2.9962	0.5847	0.4179	2.5108	0.0102	1.1435	1.1597
4.8763	1.2968	5.2355	0.9876	3.1807	2.9228	1.353	3.6972
0.2807	0.434	0.3157	0.0371	0.0989	0.1596	2.0046	1.4023
0.1806	0.0859	0.0903	2.3628	1.5908	0.112	0.3096	1.2494
1.7369	1.2261	2.0555	0.8121	0.9755	0.1799	1.7877	0.8844
2.774	2.9621	1.3919	2.397	2.552	2.5068	2.2575	14.4674
0.0163	0.0233	0.0489	0	13.3069	0.0455	0.2306	6.1149
0	0.736	0.0114	0	0	0	0.0195	0
0.1373	0.7052	0.3448	0.9794	0.6745	0.3704	0.8558	0.8925
0.2932	0.9865	0.0314	0	0.0885	0.039	0.2692	0.8693
1.0075	0.74	0.8635	1.2902	0.546	0.2747	1.8264	1.4448
0.1444	11.9531	0.1083	0.9445	0.3052	0.0672	0.4641	1.4983
1.0107	0.552	0.9271	0.527	1.023	0.2134	0.7196	2.361
3.9076	0.1109	7.0429	0.7129	6.1944	0.5991	1.1131	2.3958
0.402	1.7473	0.2193	0.3477	0.56	0.8841	1.0805	1.5168
1.0622	1.1609	0.3103	0.5385	0.3309	0.2861	0.1437	1.0132
0.2972	0.6362	0.24	0.5437	0.7489	0.7019	0.6171	0.7906
0.5115	1.7253	0.0349	0.4423	0.1638	0.0937	0.6077	1.1471
0.7936	1.8697	0.3968	1.1385	1.1715	0.2227	3.4977	1.4465
0.6943	1.2385	2.2127	0.2064	0.3974	0.6729	0.5578	1.881
0.0663	0.1773	0.0124	0	0.0175	0.0617	0.1278	0.0229
0.3254	0.2035	0.0602	1.0843	0.8405	0.213	0.4027	0.0722
0.4575	1.3909	3.4157	1.5611	2.6906	0.1666	0.5625	0.3302
2.7642	0.5372	3.7202	0.79	1.3563	1.1291	0.2432	0.6516
0.7425	1.5684	1.5815	0.6711	1.0986	0.912	1.6799	1.356
0.9511	3.1883	2.0292	1.7273	1.2467	0.5077	4.3408	5.2633
6.255	3.4107	1.6373	0.7719	0.9978	3.6238	0.4551	30.4502
0.0406	0	0	0	0.043	0	0.1567	0
1.1429	0.9919	0.695	0.1469	0.555	0.4886	2.8589	5.5982
2.2734	5.3143	0.8146	1.1187	1.8937	0.7919	3.7573	4.2917
1.5889	7.0054	1.5746	0.8548	0.5397	0.264	2.164	4.7365
0.3241	0.6013	0.6482	0.0771	0.7307	0.5026	1.1666	2.1822
1.3463	2.0067	1.111	0.8592	1.4558	1.6106	0.7885	1.6971
1.2418	1.345	2.4112	1.1603	2.1091	1.1348	2.4514	5.0924
0.6798	0.6585	0.262	0.0632	0.1525	1.4912	2.3676	1.3818
4.3582	13.6915	9.1471	6.5843	8.2558	5.0243	5.4966	4.8159
0.4407	0.6919	0.8263	0.7862	0.885	0.6972	1.3597	4.3901
0.0978	3.596	0.0898	0.6961	1.0383	0.0435	1.9422	0.6644
4.1349	4.5241	8.2645	1.2923	6.319	7.7577	4.0864	7.6481
1.9973	2.6873	3.8236	0.8248	3.2891	1.6253	5.3077	4.5769
2.7415	0.6337	1.5829	0.949	3.7406	2.2874	2.9284	5.5199

0.0754	1.1082	2.8607	0.7889	1.1951	0.1122	0.1744	0.1669
2.6875	1.6923	0.6908	0.8646	2.3856	1.5053	1.4239	5.0714
0.6143	1.0624	1.1946	0.5448	0.5281	0.5764	0.6692	3.8746
0.9018	2.8259	0.2352	0.1399	0.5801	3.4294	0.4536	1.9528
2.1728	3.0485	4.5616	1.8437	2.8584	2.4008	4.226	5.965
0.8061	0.2684	1.0076	0.1278	1.0791	0.5251	1.1399	1.6729
13.2204	23.493	18.1477	4.432	22.4888	11.8403	24.421	33.0069
0.3501	8.9039	0	0.3785	0.6055	0.0296	0.491	2.378
1.6465	0.178	0.0561	0	0.7118	0	2.854	2.3813
2.0868	0.5949	0.9717	0.9347	1.2075	0.1717	0.5827	0.4899
3.5524	4.6473	2.4249	1.4623	2.3103	3.6443	1.0538	4.7885
1.0233	0.3651	1.4252	2.0861	0.8497	1.6323	4.6979	0.4045
0.4407	0.975	0.7382	0.4542	0.7763	0.9432	1.5108	3.2316
0.042	0	0.0315	0	0.1331	0	0.2699	0.0581
0.6739	2.0609	1.5883	0.229	0.2035	1.3436	3.4031	2.4639
0.9666	0.0431	0.6796	0.8621	1.2131	0.8994	1.5532	1.2537
0.2533	0.3816	0.4116	0.1841	0.6545	0.3536	1.1579	1.9276
1.1964	0.7527	0.791	1.2375	0.7631	0.4874	2.8574	1.6747
0.251	3.5381	0.0471	0	0.2653	0	4.3566	6.1648
0.5193	0.7412	0.2532	0.3706	0.6037	0.4107	3.8387	1.4729
1.522	2.7582	0.1433	0.3788	0.1796	0.6323	0.2275	0.0686
0.0507	0.2651	1.0129	0.783	0.0892	0.1414	0.0217	0
0.446	0.0842	0.3274	1.1786	0.2569	0.1647	0.6309	0.049
1.0492	1.337	1.5624	0.5169	0.8712	0.5439	1.4643	3.4837
0.7621	1.6919	0.4922	0.8056	0.537	0.6894	0.5987	0.7615
1.1344	0.2082	0	0.0771	1.0619	0.1206	0.7083	1.5246
0.8526	0.0644	0.1924	0.0408	0.0248	0.4135	0.0186	0.0025
0.5017	0.6206	0.4515	0.1591	0.7424	0.0934	0.86	1.9899
10.201	4.9242	0.855	0.6065	0.1585	0.5304	0.5014	0.166
1.6652	1.171	2.8956	0.7175	1.6578	1.1901	3.9397	8.6137
0.9913	1.2524	1.5721	0.4058	0.8072	0.4234	2.1725	0.7194
6.4217	1.2588	6.6827	0.5528	2.2132	2.1184	2.9992	9.5873
0.5288	0.7069	2.7332	0.3423	0.2334	0.1744	0.1463	0.7871
1.4801	2.2945	2.3348	1.0925	3.7758	0.6174	4.2646	1.7653
0.2189	0.8983	0.472	0.4014	0.2314	0.3735	0.3049	0.8961
0.1522	1.2131	0.019	1.207	0	2.3373	0.0326	0.0351
1.6061	0.1348	7.4393	0.1685	2.6459	0.4395	0.1822	0.8496
0.3068	0.591	0.3451	1.1674	2.01	0.2854	2.2083	0.8063
0.1675	4.0043	0.4576	1.3375	0.0063	2.5884	1.9687	0.7283
11.7788	2.6037	44.0772	2.2108	8.2547	9.007	3.0206	3.8671
0.3745	1.394	1.3802	0.6847	0.5339	0.6903	2.8563	0.9188
1.0393	0.2607	2.5837	0.1798	0.4393	0.1875	0.421	0.3921
23.5589	17.8073	18.5325	10.9759	18.8463	15.9435	58.5536	26.8188
0.4117	1.2182	0.7309	0.6407	0.7933	0.3267	1.1751	2.01
0.5695	0.852	2.6861	0.51	0.388	0.1085	2.7785	0.8781
2.4796	0.1444	2.4477	0.2407	1.7247	0.8122	0.5204	0.9801
1.7831	4.2922	4.8838	1.9384	1.7954	3.368	1.9773	3.3137
0.784	0.3969	2.01	0.9874	0.7347	0.2731	2.8	1.8166
0.9089	0.1371	0.444	0.9449	0.5434	0.8892	1.8359	1.1208
0.544	0.7945	2.1904	0.8283	0.7818	0.3241	1.245	0.6155
2.2896	0.8417	5.125	0.3713	0.6233	0.6456	0.5797	1.0558

2.819	2.4653	4.4982	2.1424	4.144	1.4495	6.0349	4.4864
0.8983	2.0197	1.8938	1.1891	0.6448	0.8043	1.4815	2.5792
0.2336	0.1597	0.4598	0.5092	1.1725	0.3713	0.638	1.8985
2.7165	0.835	0.7522	0.1491	0.9717	2.8776	0.5909	1.2142
0.4115	2.8594	0.3395	7.01	3.1638	0	2.5192	4.2626
1.1577	0.9205	0.7689	0.1967	0.6292	0.8002	0.8078	0.4576
0.4185	0.365	0.2441	0.5529	0.1474	0.4326	0.2391	0.5146
0.1045	1.038	0.0542	0.1338	0.2463	0.0224	11.4366	3.1911
0.0804	1.0858	0.4578	0.3293	0.2014	0.1967	0.6354	0.7127
0.5007	1.2733	2.1301	2.1436	4.089	1.4231	2.7033	2.1789
12.4039	0.7538	0.8908	0.3005	2.1848	3.2686	2.0662	3.3845
1.3856	2.2138	6.2676	3.5788	8.4485	3.5406	4.0035	7.8965
1.09	0.2963	0.1246	0.2716	0.4388	0.3477	0.3336	0.359
3.657	4.7139	7.7518	6.9076	1.5095	18.9526	0.1781	3.5986
0.5954	1.8764	1.4138	1.593	0.6816	0.3693	0.1276	1.1668
0.124	0.8627	0.279	0.3318	0.5897	0.2307	0.5977	0.5575
0.4548	0.4612	0.1795	0.4555	0.8348	0.0891	2.9232	0.0993
2.4809	0.8388	0.8942	1.6801	0.4838	0.5363	0.3632	0.1798
1.5788	0.3811	0.9402	0.3037	1.2391	0.7345	2.0892	8.3354
1.808	1.7722	2.2728	1.6044	1.3285	1.0016	2.7164	1.3818
0.1741	0	0	0	0.5521	0.162	0	0.2409
0.6971	3.6014	1.7565	0.0995	0.943	0.5708	1.2545	1.543
0.4341	0.3356	0.1085	0.4302	1.2616	0.5722	2.511	3.5032
0.5828	0.3254	0.171	0.3314	0.4284	0.9902	0.456	0.4907
1.088	5.4876	0.0132	0.0247	0.8076	0.9335	1.8157	1.5627
0.1653	0.2528	1.913	0.4213	1.5476	0.2637	3.1576	0.6535
1.7073	0.8606	1.6679	0.8754	0.8939	0.2332	1.7718	0.4984
2.2034	6.7864	2.4111	3.9035	3.3503	2.4895	5.3467	7.3406
1.0611	2.3495	1.3982	0.2712	0.5764	1.8271	2.3839	1.5229
3.4296	6.5561	9.0294	3.1617	3.1922	3.279	8.4735	8.6043
0.4916	0.3203	3.1683	1.8385	0.7605	0.5237	3.7458	1.1729
1.3585	1.7691	0.6112	0.2019	0.2871	1.5168	0.8295	4.5574
0.7642	0.2505	0.7434	0.5894	0.5457	0.6149	1.1947	0.6857
0.2007	1.2173	1.919	0.537	0.2121	1.0271	2.1284	1.3189
0.8915	3.1686	0.5676	0.44	1.0132	0.8138	6.5296	3.4439
0.1324	0.3104	0.3688	0.3599	0.3798	0.1936	1.7021	1.3608
0.4181	0.0476	0.5635	2.0322	3.3559	1.1277	1.7605	0.6958
3.0624	1.7482	0.8703	0.4217	2.4869	2.2195	6.7128	4.1028
0.2108	0.2106	0.137	0.3175	0.5495	0.2746	0.9573	1.458
0.8946	0.4426	0.814	0.5107	0.769	0.1998	1.4874	0.6436
0.7444	0.2015	0.3272	0.5189	0.1356	0.1672	0.3629	0.5681
0.382	0.8518	1.2532	0.3975	0.1514	0.8885	0.1841	0.6605
3.7537	4.5207	19.0236	1.0375	6.577	8.4548	7.2061	13.3694
0.1076	0.4609	0.4238	1.4561	0.1422	0.6886	1.3838	0.1675
2.2443	2.4251	3.4301	4.3718	2.1685	1.591	7.7205	8.6639
1.4051	2.1902	0.8498	0.6314	0.6638	1.452	0.8324	0.851
5.3793	18.8306	4.7952	0.7242	1.4481	3.5414	5.1535	9.5482
0.8571	0.7287	0.4682	0.2391	0.3075	0.3397	0.4693	0.5197
1.5966	0.1139	1.0776	0.0475	0.5905	1.1884	0.2565	0.8283
2.0042	1.3925	4.7552	1.3684	2.1966	1.4231	2.1695	1.4891
0.0532	0.4936	0.1277	0.2784	0.4724	0.6733	0.9302	0.692

1.5682	0.9123	1.8216	1.7534	1.9906	1.0864	1.742	3.3958
0.1351	0.6747	0.4305	0.2811	0.9992	0.4399	0.8681	1.4482
0	0.6954	0	0.0374	4.4519	0.819	1.8589	1.8266
1.0093	1.1506	1.2923	1.6833	2.5757	0.3894	3.8607	1.4474
2.9011	0	1.6428	0.352	1.8145	0.7162	0.2283	0.6553
0.4583	0.5635	0.2961	0.4947	0.3279	0.1575	1.6134	1.0633
0.064	0.2638	0.128	0.0085	0.1503	0.0198	0.064	0.1672
0.5959	0.9328	3.7913	0.5943	0.4266	1.1	0.6548	1.2366
0.3416	1.1301	0.0931	0.3693	0.0328	0.1156	0	1.8902
0	0.1221	0.1283	1.0988	0.434	0	1.7597	1.7518
1.4678	1.8241	1.0636	1.4709	0.9063	1.3811	2.2471	1.6884
0.0493	11.3366	0.037	0.0587	0.1043	0	0.1268	0.2048
1.6132	2.6702	2.9558	2.5512	3.1109	0.3559	18.0004	26.6892
0.3183	2.0897	0	0	0.1346	0.1777	0.8183	0.5284
1.3071	7.901	1.2109	0.5029	0.2844	0.465	0.1977	0.4787
0.7687	0.1724	2.0251	0.1632	0.29	0.1161	0.0762	0.0841
1.1262	2.2043	2.0511	0.4592	1.5643	0.7485	0.5791	0.6232
1.9842	3.3037	0.8353	0.0103	0	0.7045	0.0336	2.5042
1.2028	0.479	2.664	0.3659	0.7981	0.4164	0.0719	0.1935
0.9265	0.619	1.0496	0.5861	0.5	1.3207	1.2163	1.8
0.2181	0.716	0.9105	1.1067	0.7299	0.3112	1.1682	1.7703
1.0489	1.8917	2.8956	2.0286	1.1572	0.8794	2.3113	4.4489
0.6557	0.2423	1.159	2.3809	0.1609	0.4031	0.4365	0.8099
0.375	1.0217	0.6136	0.6892	0.6125	0.793	2.4979	2.4525
0.7909	0.4999	0.9151	0.9271	1.6001	0.6413	1.4088	1.7194
0.253	0.4751	0.0799	0.2692	0.2111	0.1239	0.154	0.6447
0.8415	3.2964	1.7455	0.6813	1.4003	0.833	2.0024	1.9569
3.0423	0.7435	1.1939	0.3866	3.0566	1.1013	1.6607	0.9686
15.2169	14.5274	10.5157	2.3469	7.245	8.4566	40.9906	58.4853
1.1864	2.3312	0.851	0.4907	1.3992	0.336	4.1774	4.2342
0.2948	0.2589	0	1.0606	0.0559	0.0281	0.1457	0.4287
1.308	0.8061	1.8169	0.7954	1.7279	1.0787	2.7513	3.4134
1.6889	34.2623	1.3005	2.1297	1.0661	0.7172	1.3113	4.6887
3.6584	2.4781	2.5646	0.5044	3.39	1.036	6.9177	11.6994
1.2766	1.7276	1.4855	0.9422	1.3724	1.6383	2.0088	3.4408
4.5149	6.7621	8.39	2.891	7.2358	6.1629	9.9491	7.5159
19.8385	2.0259	0.2563	0.6252	3.7875	12.344	0.4731	1.091
0.0122	0.0456	0.0114	0.2134	0.0964	0.0651	1.9196	0.0042
2.351	5.7081	1.4191	1.0058	0.8787	1.4673	2.7642	3.907
0.3933	0.7298	1.9337	0.6549	0.6466	0.2359	1.4158	1.6446
6.9807	2.7119	3.63	2.8781	1.2895	5.6764	0.8228	4.2061
1.6515	2.7691	1.3373	0.7109	1.3706	8.5886	6.4862	11.7185
0.2517	1.552	1.4875	0.6825	0.4256	0.9275	2.0968	2.4656
1.345	1.0162	1.6316	0.6586	2.3412	0.6257	1.1696	2.2985
0.329	0.1945	2.1747	1.1793	0.8891	0.328	0.2719	0.1691
0.6198	0.0508	0.1496	0	0.0151	0.9679	0.0458	6.4363
1.0434	0.1209	0.8448	0.4544	1.2011	0.136	2.029	2.6067
0.9778	4.4022	1.5172	3.5512	0.5741	0.7595	0.1661	1.0602
0.2422	1.1934	0.8736	0.3155	0.6948	0.0322	1.1564	1.1329
1.8177	2.3681	1.631	1.8791	3.0702	0.4748	1.4612	0.9348
0.4395	1.7536	0.0599	0.4277	0.1689	0.6321	0.1027	0.0276

6.859	0.0365	1.7274	5.3565	3.0834	1.0954	7.3694	1.2747
1.2653	0.8762	1.1879	0.9251	2.2288	0.7064	2.6989	6.5094
0.6667	0.7327	0.42	0.4916	0.606	0.1365	1.2856	0.3321
1.0223	0.3647	0.4312	0	0.2026	0.2378	0.7392	0.9723
1.1101	0.1251	1.7088	0	0.741	0.7067	0.1502	2.2633
1.5406	3.004	3.9026	7.6158	5.2915	2.9086	11.5253	17.7075
0.3469	0.3796	1.058	0.0275	0.22	0.0646	0.9216	0.3839
0.5705	0.111	0.1945	0.185	0.5755	0.4343	0.2666	1.3631
0.7647	1.285	1.1655	0.7041	1.1471	0.1148	1.1416	2.389
0.3681	0.4871	0.01	0.3581	0.877	0.2179	0.5764	1.074
0.1939	0.1615	0.709	0.2883	0.4953	0.188	1.7867	1.3192
1.5793	0.9739	0.8919	1.4607	1.9781	4.0273	4.3609	3.9381
2.8303	4.0234	3.7854	0.9011	4.1687	2.0218	10.9862	9.6193
0.7919	0.5167	1.1199	0.8072	0.5978	0.3579	0.5526	1.0956
9.3359	6.0606	9.2759	2.2444	2.992	1.1603	3.3793	8.3929
0.4846	0.1215	0.2063	0.109	0.706	1.0481	0.4546	0.453
1.7222	0.8031	2.1208	0.1364	2.8991	0.6186	0.4854	0.5637
1.4247	0.113	1.4958	0.0377	1.0707	1.0605	0.1221	0.7008
1.2687	0.6732	1.7321	0.1161	2.0284	0.454	0.7109	1.3951
0.9742	1.2166	1.0959	1.0084	1.3823	0.7302	2.0524	0.6739
1.5589	1.1461	1.9484	0.0562	2.3464	0.4395	0.7894	1.1763
5.7523	5.1942	5.4274	2.5646	5.6692	3.3496	3.5873	2.4238
7.778	10.2997	1.509	0.8283	1.8812	5.8689	0.3979	4.4434
5.8035	5.8585	0.3114	7.3255	1.0072	0.1635	0.0342	5.1856
0.1832	1.3767	0.0808	0.2307	0.262	0.0903	0.0277	7.0228
0.2752	0.8328	0.7044	0.8575	0.9827	0.6356	1.1831	3.4653
1.0852	0.9981	1.6548	0.4015	1.7968	0.5385	1.6275	1.2345
0.692	0.6564	0.3092	0.1989	0.2011	0.3434	0.8303	0.9773
1.5172	1.68	2.0009	0.4355	1.0505	2.1906	0.9079	1.8456
2.5858	2.4091	5.3699	0.9749	2.5367	2.3634	5.1759	5.9237
9.5051	7.2046	4.5659	2.6826	5.5781	8.5792	7.8265	6.8173
1.1654	0.5492	1.1873	0.7452	1.5221	0.8593	1.2013	1.7188
0.6474	0.1732	0.2427	0.0481	0.0855	0.1506	0.052	0.056
1.6572	0.1213	5.449	0.0505	2.2902	0.2372	0.0546	0.5291
1.2373	0.5325	4.2758	0.0758	0.5459	0.3382	0.0574	0.0794
0.3438	0.3434	0.0859	0.4906	0.5571	0.1279	1.09	0.4439
1.0313	0.3761	0.4125	0.5178	0.654	0.2985	0.5008	0.3805
0.1681	0.4798	1.0505	0.6663	1.2435	0.3649	0.3601	5.4261
0.3711	0.4237	0.3896	0.3824	0.3399	0.5064	2.2577	1.8138
0.1076	1.6131	0.5247	0.32	0.1706	0.7011	1.107	1.4148
0.2775	1.0326	0.9514	0.6601	0.9427	0.5349	3.491	2.5779
0.2484	0.9368	0.6918	1.4683	0.6225	0.482	0.8027	1.1192
0.1281	1.0664	0.7364	0.8124	0.5415	1.1918	2.0307	1.4767
0.2963	0.6695	0.4321	0.5677	0.6959	0.3982	1.6928	1.4575
0.2644	0.7863	0.2644	0.2096	0.8384	0.164	1.8696	2.1951
0.6112	0.1796	2.5346	0.5558	1.0259	0.2007	0.6471	0.2985
0.7964	0.5338	0.9049	0.287	0.7396	0.2021	0.9927	0.601
0.8859	2.0703	0.5315	1.1851	1.1	2.6789	2.2206	1.3481
0.9674	3.5946	0.9215	0.912	1.0592	0.9245	2.2518	2.0859
2.7713	0.1798	0.2159	0.0214	0.8748	0.7702	0.3933	0.1743
0.4779	0.3979	0.4778	0.3789	0.9259	1.4081	1.9453	1.8732

0.5003	0.252	1.3243	0.77	0.7931	0.2602	1.8538	0.9976
1.1996	1.556	1.9686	0.8811	1.6109	1.2278	0.898	0.7712
0.1044	0.3311	0.0957	1.6417	0.4291	0.6585	1.208	0.979
1.3178	1.2566	0.4198	0.2635	0.7025	0.6511	0.5697	0.5002
1.2091	0.2734	0.0894	0.0911	0.6209	0.515	0.6239	1.0837
0.5703	4.4768	2.1385	1.5502	0.7319	1.7057	2.2518	2.5361
0.3806	0.6337	0.7611	0.1509	1.0726	0.4722	0.4893	0.7021
0.9586	2.0319	1.5223	1.9446	1.311	1.5215	1.9208	1.8332
1.8811	0.2065	0.7867	0.3011	1.6057	0.8078	0.93	0.3503
1.7762	0.8714	0.666	1.1881	1.7598	1.3428	0.7135	1.2286
1.6254	1.1422	2.8874	0.2756	1.0809	0.8999	0.7666	1.5475
0.0556	0.8974	1.0015	1.9587	1.1644	0.0414	0.0572	3.0177
0.6578	0.1502	1.8483	2.2217	4.2362	1.1507	1.0937	0.4611
2.015	1.819	7.1117	1.9018	3.8421	1.8466	5.4846	2.4434
0.1783	0.7395	1.0696	0.5433	1.0363	0.394	0.9883	0.632
1.3986	1.1227	0.5244	0.2495	0.9977	0.4229	1.3034	0.3386
0.4892	0.917	0.8939	0.5753	0.7254	0.3091	0.8987	0.894
0.8832	0.9337	0.6133	0.2723	2.0741	1.2783	0.5466	0.8146
0.2703	0.5511	0.1448	0.7806	0.1632	0.2874	1.9854	0.9081
0.132	0.888	0.7353	0.3588	0.1594	0.2632	0.6544	1.1478
0	0.1393	0.8294	0.3868	0.6188	0.3632	0.5854	2.3402
0.8982	0.5304	1.5755	0.2946	1.031	0.658	0.6635	0.7927
0.309	0.651	0.3973	0.665	0.7309	0.2875	1.0593	1.364
0.1103	0.5773	0.4688	0.3498	0.3109	0.0684	1.04	2.2385
2.1208	0.1513	1.3121	0.1892	1.681	0.1973	0.9542	0.6602
0.5542	0.6215	1.5833	0.408	0.7252	0.3438	3.1211	2.5558
1.8175	1.3166	4.8943	0.6877	1.4551	1.1274	1.8053	1.8286
0.2925	1.1384	0.6181	0.6324	0.562	0.9896	1.8457	3.936
0.2751	0.6763	0.4929	0.4726	0.4523	0.2276	1.1396	1.586
3.9996	24.7198	23.1082	37.3084	6.1131	18.6274	1.3268	1.7254
1.0525	1.1736	0.518	0.5867	0.9038	1.0712	1.2684	1.7292
3.1052	8.5641	5.1114	3.2495	2.1003	0.4767	5.9275	12.7803
1.327	0.8741	0.9186	0.2428	0.863	1.8046	4.1334	4.7309
0.2256	0.922	1.3228	0.3415	0.4335	0.458	0.3955	0.6242
3.3608	3.3807	0.6862	0.722	1.8346	1.7664	4.2322	3.4546
0.4458	0.2019	1.0478	0.7645	0.9241	0.1914	1.2672	0.8064
0.3141	0.6912	2.1693	0.716	0.913	0.1827	1.0095	2.1911
1.8627	2.3972	0.9728	0.1582	0.703	0.8975	0.5131	0.4602
0.1996	0.2611	0.5488	0.4351	0.6679	0.6809	1.411	1.0123
0.2781	0.8506	0.5364	0.8978	0.4199	0.8504	2.4517	1.979
0.5872	2.06	0.899	0.7564	0.8532	0.4098	1.9498	1.76
0.4176	1.9687	0.9394	1.2414	1.6706	1.013	2.9906	2.8678
5.6075	2.8843	0.0427	1.8617	0.9175	7.482	0.6586	8.939
0.3506	2.1658	0.7231	0.443	0.9148	0.2855	0.6197	1.8494
3.0472	3.4619	1.6236	1.6688	2.161	1.828	3.1954	1.9969
1.5538	3.348	0.5712	1.3767	0.0644	1.1339	0	0.0421
3.5266	0.0735	1.525	3.0917	2.5028	0.479	6.5187	0.8547
2.4924	8.6499	1.1546	1.75	1.3845	2.2795	0.4374	6.5315
0.3517	0.6745	0.511	0.4444	0.2091	0.1227	0.3956	0.4562
0.557	0.6624	0.4235	0.276	1.0873	0.4535	1.1436	2.0655
3.2187	4.3925	6.0961	1.3969	3.4265	2.3172	5.255	4.7455

1.3393	1.0467	4.0892	0.6826	0.8088	0.5934	1.3527	2.5587
0.0395	0.3943	0.148	0.1877	0.1251	0.6611	1.167	0.6553
0.4491	2.9137	2.0209	0.4856	0.0863	0.9118	1.0497	0.5649
3.2532	2.9028	2.9823	2.379	4.0485	0.7582	7.1911	2.6078
0.5625	0.4647	1.5096	0.264	0.9386	0.3718	0.9323	0.2457
0.6203	0.7932	1.9874	1.3123	1.5323	0.8694	1.7913	0.9472
10.9551	30.6802	26.7002	10.7046	10.6199	10.9241	11.2347	21.285
1.309	0.4694	0.6746	0.5428	0.8939	0.7183	0.8456	0.6222
2.4157	5.308	11.1388	5.6046	4.2276	3.259	7.2487	3.6372
2.5237	7.9557	3.3539	1.9374	1.1816	2.6454	0.9855	5.5686
0.385	1.2029	1.8985	1.0752	0.863	0.2985	0.9073	0.0726
2.3071	4.7912	2.6618	1.3718	1.4223	1.7201	2.7185	2.2711
0.3856	0.0061	0.4883	0	0.0543	0.008	0.5397	0.1422
2.0215	0.2457	0.8766	0.5178	0.8801	4.258	0.1128	0.2199
4.9275	6.0101	2.2343	0.2725	0.9083	4.2116	3.0197	2.1402
0.2858	0.1172	0.1915	1.0847	0.6105	0.3734	0.7895	0.1346
1.0357	0.0061	0.5007	0.8245	0.7327	0.7009	0.033	0.4618
0.8805	0.9973	1.0884	0.6788	0.6195	0.3763	1.2997	0.7861
5.6204	13.2426	22.8798	4.3506	2.5743	4.2243	3.2408	6.5693
0.4401	1.7437	0.6514	0.5922	0.0449	0.1185	0.1935	0.5341
1.623	1.0229	0.2547	0.9016	0.0292	0.474	1.3198	1.6717
0.3459	0.8829	0.6036	0.6566	0.4781	0.6066	1.163	0.3221
0.0333	11.6044	0	0.1585	0.3874	0.155	0	1.2448
0.0596	3.6438	2.0998	0.0304	0.018	0.1029	0.0656	0.2708
0.4285	0.5657	0.5945	0.3567	0.2264	0.299	0.7987	1.5413
0.5454	1.178	1.4038	0.8414	1.1683	0.7955	2.0084	1.2439
1.1131	0.5863	0.0556	0.1576	8.0439	0.0148	5.1442	9.0121
0	0.198	0.0446	0	0.1466	0	0.1529	0.1097
0.5116	1.0838	1.2469	1.8441	2.0613	0.8777	1.3769	3.0964
0.9937	1.2469	0.2844	1.4272	1.1212	1.8849	1.4008	4.1972
0.5827	0.9086	0.5017	0.9496	1.0948	0.3615	1.7755	1.5526
0.8237	0.8058	0.533	0.4818	0.5826	0.6551	1.5369	2.3341
0.486	0.7691	0.8954	0.2513	0.1228	0.4129	0.747	1.1987
0.1477	1.5283	1.1354	0.3952	0.3512	0.6872	0.1424	1.5837
0.2177	0.0518	0.5876	3.0889	0.6748	1.5122	2.7234	1.0239
0.426	0.4677	0.4178	0.0779	0.3117	0.2439	0.9268	1.4961
0.4217	0.2046	0.7463	1.1835	0.7487	0.3924	0.7155	0.2567
0.3457	3.0295	3.9134	1.5872	0.153	0.0823	0.093	0.0334
2.2149	2.0235	2.8777	0.8053	1.1182	2.6647	0.4352	2.752
0.5692	0.0729	1.6198	1.0587	0.5553	0.3259	1.5947	0.7673
1.4484	0.3672	0.8861	0.3626	1.6314	0.1951	0.294	0.3164
0.5318	1.5938	0.8175	0.2846	0.3372	0.8907	2.666	2.2807
0.9693	0.3052	2.0097	0.712	1.7475	0.3979	0.3665	0.9861

TCGA-BA-51	TCGA-CV-7(	TCGA-CV-A(	TCGA-CV-72	TCGA-CV-69	TCGA-CV-69	TCGA-CN-4730-01A-01I
0.9203	2.8061	7.0122	3.9897	4.6466	0.1528	0.1957
2.235	1.6156	9.291	6.1363	1.9983	7.6957	4.7071
0.8891	1.2064	2.8585	1.5066	1.3853	1.6665	1.0458
0.9189	0.8016	2.5507	0.5135	1.3887	3.7254	0.7209
1.5665	0.4073	1.3646	0.2575	0.8159	1.158	0.4855
2.3413	0.5627	7.9665	1.2604	1.5852	1.6205	2.6164
0.896	0.463	0.2754	0.5989	0.8753	1.0053	0.7581
1.2312	0.6307	3.2978	0.5528	0.7582	2.427	1.1776
0.0659	0.2771	1.6201	0.378	0.7682	3.4451	1.4276
0.3853	0.5512	1.2798	0.6119	0.9104	4.4184	0.0915
1.315	4.3751	18.3544	1.8208	1.7009	3.5887	0.7024
0.1763	0.7089	3.4641	0.4536	0.7658	0.803	0.2628
0.3406	0.7159	0.1603	0.4632	1.6681	0.5005	0.2552
0.3043	0.9481	1.7232	0.1546	0.076	8.8913	1.7409
1.1418	0.8246	0.1139	0.8672	0.7817	1.8969	0.7471
2.0653	1.8257	46.2413	1.2015	3.3227	1.344	0.3768
2.5618	3.478	15.7409	17.4103	7.2368	4.4918	0.8795
0.407	0.2559	5.9525	1.1379	3.5621	0.7736	0.8858
0.7181	0.9083	1.4821	1.9491	1.4415	4.6283	0.8588
2.9392	0.5262	0.8557	0.428	1.5827	0.7912	0.557
9.4468	5.9772	6.0879	5.1805	11.6554	9.3954	7.5055
8.4068	2.1779	11.1111	5.2962	7.7334	2.3701	1.3561
2.9976	1.1274	1.9384	1.0085	2.0604	3.2993	1.8093
0.9746	1.4373	1.474	0.6005	2.1845	2.0059	0.4166
1.7008	1.2391	0.6673	1.1355	3.9154	0.6704	0.7512
0.4753	1.7753	2.482	3.4641	2.0666	3.7693	1.6459
0.6534	0.4978	7.3539	0.489	0.4494	1.3323	1.337
7.073	10.647	1.9287	4.9241	8.6805	2.9334	4.8111
0.1806	2.4258	0.062	1.742	1.198	2.6348	0.3233
2.7926	2.1383	2.1184	3.1285	2.4574	1.694	1.0538
0.3576	0.2669	1.8246	0.4088	0.8278	2.058	0.7003
0	0.0204	0	0.165	0.1756	0.0358	0.0363
0.8748	0.1187	7.4913	0.4535	0.2556	1.5876	1.7414
1.2955	0.5705	2.8808	0.4718	1.2285	2.9375	1.0552
1.0469	1.6668	0.4786	0.938	1.7861	1.0165	1.5826
1.9486	1.5826	8.1924	1.4437	1.6996	4.5654	4.6908
7.6877	4.6231	9.0911	1.5141	10.3916	9.6185	9.0499
0.1108	0.1413	0	0.0635	0.3549	0.062	0.157
0.1557	0.8981	0.3339	0.5796	1.2709	4.9039	1.0489
2.5691	0.5994	4.7943	1.6434	2.3948	1.2442	1.1237
0.406	1.3974	0.7198	0.2293	0.4482	2.0573	0.3826
0.4258	0.6788	2.639	1.0584	1.8652	3.3074	3.4708
0.3952	0.3215	1.1292	0.2428	0.4246	2.0641	0.2058
0.0572	0.2405	0.4248	0.0596	0.2143	0.9019	0.1032
6.7506	4.7833	2.8698	5.3331	4.7288	8.269	3.1555
0.0229	0.5102	10.9328	1.1793	2.197	1.3424	4.7955
0.5839	0.3398	6.7753	0.7969	0.6363	3.5573	0.6175
0.4598	0.1375	1.2014	0.103	0.8221	3.2953	0.2444
0.2656	0.0041	2.0758	1.3046	0.0413	1.2477	0.8335
14.8414	13.5397	3.7402	12.9486	18.823	12.8993	14.9003

0.4357	0.8335	0.8189	0	0.4985	3.5133	1.3794
1.3969	0.6898	0.9694	0.9908	0.958	1.2562	0.26
1.176	2.2016	1.5542	1.8726	1.7238	4.7433	1.6053
8.3742	5.1144	26.5094	4.285	4.3123	6.6199	1.308
0.9876	2.233	5.9354	1.261	4.4382	1.1048	1.7055
0.7309	1.0783	0.5515	0.938	0.1997	1.1746	0.3093
0.391	0.2255	3.5422	0.298	0.6854	0.8375	0.2241
0.2904	0.195	2.4074	0.1533	0.3848	1.1756	0.26
2.0182	0.1628	2.2121	2.171	1.1822	3.5093	0.7238
0.1758	0.4837	6.8709	1.586	0.8521	7.0433	0.1466
0.3957	0.1246	0	0.195	0.1211	1.7974	0.9182
1.0813	1.1011	0.5724	1.3631	4.0034	1.2825	1.4847
0.3328	0.1883	0.9603	0.3693	0.4668	3.0026	0.1826
0.0415	0.5287	2.418	0.7723	0.3795	0.2319	0.0588
0.1438	0.3491	0.981	0.6646	0.9826	1.9213	1.266
6.0455	1.7962	23.0286	3.0127	1.8552	12.6038	3.5449
0.0225	5.3528	65.566	0.0644	0	0.0314	0.1273
0	0	0	0	0.0239	6.4543	0
0.5198	0.4671	3.7392	0.7652	0.4325	1.5659	0.4756
0.1154	0	1.1776	0.1653	0.198	0.0403	0.4497
0.8435	0.9884	1.3091	0.4515	1.0524	1.9576	0.3746
0.348	0.0793	4.736	0.0712	0	1.5987	1.1979
1.3435	0.5248	1.8422	0.3069	0.5268	1.1826	1.3696
0.4127	1.0479	1.2186	0.9858	3.1134	2.0092	0.8017
0.4529	0.361	8.0968	1.0638	0.4895	1.2139	0.9809
0.4734	1.3869	15.3272	0.9984	0.3435	10.953	1.5714
3.7771	0.1255	2.7541	0.1128	0.072	0.8803	1.0709
0.032	0.1956	0.0311	0.5123	0.0855	2.5515	0.363
0.5724	1.355	4.1145	0.9322	0.3573	2.1587	0.5287
2.7683	0.4128	5.0729	1.1132	0.7748	1.9495	0.9599
0.0228	0	0	0.0327	0.0522	8.2308	0.3558
0.719	0.5423	0.1882	0.6698	2.0505	0.1856	0.145
2.2595	1.3536	0.3994	0.5887	1.0342	1.3978	0.3882
0.7023	0.4447	0.3276	1.0512	1.1407	0.3386	0.4316
0.8384	1.1737	1.9483	1.5239	1.7783	2.8595	0.2319
0.5888	1.5295	5.3298	1.5495	0.6505	9.9659	1.9645
6.3783	0.0648	0.7899	3.9009	1.3946	0.6817	0.2304
0	0	0	0	0.064	0	4.7998
0.8294	0.1356	2.6051	0.6705	0.8761	1.0706	1.025
2.4981	0.4179	6.2515	1.3481	1.0058	1.7897	1.8362
0.6511	0.6436	5.596	0.0933	0.7749	6.5011	0.4246
1.5174	0.5692	2.7768	0.4264	1.2597	1.0193	0.8014
2.0148	1.0955	3.3957	1.4002	0.6961	1.226	2.1815
2.8243	1.0222	4.2745	1.1596	1.9565	3.1381	1.0388
0.2981	0.5044	0.4931	0.6258	0.8018	7.1733	0.5782
16.4178	10.6805	16.6584	9.8173	9.5875	11.822	8.9082
1.2137	0.387	0.236	0.8697	0.3472	1.1881	0.043
1.0543	0.1202	0.8188	0.0576	0.207	1.9634	2.5888
6.4852	3.6329	27.9719	4.0086	9.236	6.1948	8.124
2.3152	1.0085	8.4684	1.3831	5.1553	4.5625	3.4556
1.3735	2.5853	15.0586	6.2734	1.9468	2.8004	0.7286

1.173	0.7447	0.1211	0.2529	0.2851	0.8274	0.103
2.5552	0.6685	8.2653	0.785	1.3675	7.2745	1.761
1.344	0.6342	0.8502	0.6626	0.7956	1.3231	1.072
3.1313	0.3443	1.5746	1.1219	1.0502	7.4363	0.5357
4.3553	1.2049	6.1733	2.7245	2.5765	4.9681	4.1584
1.3505	0.7374	3.669	0.7424	1.0161	1.2935	1.7571
16.0242	3.2251	22.3968	9.2366	16.1946	21.1821	27.6815
0.2191	0.0349	0.767	0	0.1003	0.9806	0.5903
0.2233	0	0	0.0492	0	0.1921	0.0243
0.4573	0.9592	0.3345	0.8994	0.4728	2.2762	0.8492
0.2508	2.1995	0.1219	1.6177	0.5023	2.0607	2.9781
4.1265	1.4441	1.272	0.8654	11.4014	0.1407	0.1427
1.1126	0.4354	3.245	0.7393	0.3472	2.3903	1.4482
0	0.1844	0	0.0829	0.1985	0.3234	0
0.2651	0.8982	2.8352	0.7599	1.6685	7.3681	0.6107
3.993	0.1326	0.3235	0.298	2.8556	0.1163	0.2358
0.2713	0.1545	1.9594	0.3611	0.3548	0.9891	0.0962
0.4143	1.2199	0.9761	0.9779	0.9481	2.4467	0.9465
0.3025	0.0689	1.2602	0	0.2967	1.8127	1.0413
0.1073	0.3705	3.9975	0.0769	0.5319	3.2249	0.1521
1.8326	0.1088	0.3222	0.0908	0.1562	2.1332	1.6651
0.1279	1.742	0.226	0.5331	0.7449	0.5526	0.2142
0.3558	0.1036	0.0821	0.2491	0.8886	0.9064	0.0345
2.0226	0.9542	10.554	0.9909	0.921	1.9913	1.3897
0.3352	0.3718	0.6801	0.188	0.467	1.8954	1.0744
1.9637	0.1423	5.3367	0.0959	0.3064	2.9644	0.0633
0.1268	0.1705	0.2104	1.7252	0.905	0.0435	0.2909
0.2994	0.1836	0.4925	0.7921	0.7905	1.2237	0.7182
3.243	0.3293	0.1205	1.8946	1.7963	3.0329	26.3245
1.0301	0.7152	15.4096	1.1429	2.7757	9.363	2.5202
2.204	0.9989	8.4839	2.0203	1.3058	0.923	0.8406
2.408	0.6009	3.449	3.3267	5.2575	2.1667	3.0446
2.9932	0.3967	8.3736	0.3104	0.1898	2.916	0.503
1.5813	1.6807	1.7081	1.6619	5.79	1.5232	0.8966
0.7348	0.3905	2.9187	0.4411	0.0862	2.8807	0.8102
0.2096	0.0279	4.3138	0.5508	0.04	0.1466	0.3962
0	0.1556	0.0632	0.233	1.6374	0.2274	0.5993
0.169	1.8183	1.4783	1.1804	1.2567	3.7212	0.3593
2.5742	1.0441	7.9202	4.5332	1.1499	2.6781	2.3822
2.0539	2.3317	6.7465	4.9203	10.946	4.6904	2.5005
0.6129	0.2753	1.2626	0.6936	1.2635	1.2107	0.3517
0.2255	0.2143	0.2698	0.2424	1.3	0.3274	0.252
37.4992	10.7093	87.5371	9.5003	27.0202	12.9173	22.1309
1.3337	0.3057	1.0697	3.2434	0.4388	4.9189	1.223
0.5126	0.2711	0.8267	0.2031	0.3189	1.1561	1.4465
0.1219	0.4582	0.2371	0.5243	0.897	0.682	0.7285
0.7218	1.8227	9.5271	3.2015	0.4702	2.5565	2.8605
1.4332	1.2972	3.0865	0.5525	1.0466	2.0896	0.343
1.6376	0.399	4.7414	1.0351	1.1292	1.175	1.4294
0.5128	1.1329	0.5887	0.306	1.0734	1.6055	0.1737
0.6687	0.6092	0.4179	1.3691	1.2025	1.4695	1.3543

4.1515	1.5122	9.0936	1.328	5.1429	0.602	2.2341
2.8354	0.9859	1.4293	0.6187	0.7477	2.4801	0.2316
2.908	0.6303	5.3807	1.6517	0	1.04	1.4153
0	1.7431	9.6785	1.0721	1.1194	2.4945	1.1421
4.664	0.1073	11.6577	3.3395	0.0973	5.477	0.2058
0.6832	0.2904	0.1328	0.2937	0.5211	1.1463	1.3558
0.096	0.3062	3.3611	0.4129	0.3663	0.6715	0.0908
0.0111	0.097	0.1506	0.0396	0.1773	0.147	0.3608
0.6396	0.1275	1.6701	1.1047	0.6897	10.1508	0.1656
2.5384	1.6688	4.5457	2.1918	3.5573	11.1047	0.9863
1.994	1.5091	0.6834	2.2792	1.9352	0.764	2.58
2.3333	2.8244	1.7358	1.2077	1.973	6.5801	6.4656
1.5723	1.0141	0.2918	1.1881	0.2617	0.5497	0.3039
10.0349	3.22	3.1606	9.4884	4.239	11.706	16.1332
0.3416	0.9802	0.0664	0.2448	0.3127	1.8628	0.8232
0.2561	0.068	0.1245	0.3058	0.4396	1.5816	1.5429
2.2248	1.2086	0.9293	0.2126	0.4526	1.2214	0.3972
6.1706	2.9154	8.0189	0.6245	0.8904	5.5657	0.91
2.0381	0.7518	3.2945	0.5154	0.7316	0.7153	0.6232
1.3803	1.9052	2.2171	1.39	1.6904	1.6484	1.0012
0.3597	0	0	0	3.018	0	0.3399
0.9023	0.5203	0.0373	5.1722	1.6255	1.6106	0.381
0.1992	0.4765	5.9079	0.1785	0.285	2.403	0.1765
0.2791	0.3615	1.4585	0.325	0.2395	0.7318	0.0989
0.5751	0.0219	0.0524	0.571	0.1209	1.1675	2.5785
2.244	0.363	0	0.233	0.5954	0.1819	0.1153
0.8735	0.5501	1.8032	0.4173	1.2586	1.9754	0.4892
3.328	3.5043	46.8009	2.1518	3.5779	16.8615	2.677
0.8125	1.0232	2.6684	1.5656	0.9532	4.6496	1.2389
4.1159	3.6148	9.9614	2.8044	4.0982	7.4316	1.3716
1.8787	3.4643	2.6228	0.6538	0.7297	2.2086	0.1669
0.5845	0.7083	6.9102	1.4074	0.3746	4.4793	1.4252
0.8389	0.0227	2.5988	0	0.9111	0.8749	0.383
0.6563	1.6522	0.2687	1.3862	1.5811	5.8931	0.4407
4.2962	0.5354	10.65	1.4604	1.007	8.226	2.134
0.2735	0.1661	0.5317	0.6719	0.2086	1.5661	0.2769
1.0096	1.7228	0.2271	0.5381	1.4608	0.2159	0.1774
7.1462	0.7784	8.1557	1.304	2.2855	2.762	1.636
0.2999	0.4627	2.6147	0.4159	0.4649	0.6358	0.2331
1.6754	0.3535	8.6069	0.2825	0.3947	2.2279	0.7334
0.3004	0.1127	2.3021	0.2026	0.1213	1.7297	0.2004
1.9066	0.2096	9.7791	0.5182	0.3009	1.5629	0.8854
3.4868	1.6991	10.9421	8.374	13.9432	15.7601	5.1393
0.843	0.4283	0.036	0.0797	2.6925	0.013	0.1051
6.7838	1.7595	16.1086	3.1001	3.0309	5.4735	3.7131
2.6436	1.0874	1.5949	0.8178	0.8876	1.9263	1.7
9.9924	0.9469	34.3743	2.2532	1.6791	12.2629	2.0802
1.6283	0.5691	0.3471	0.924	0.8253	0.8914	0.5577
0.055	0.4819	0	0.6301	3.4588	0.1921	0.5454
1.3749	2.384	2.0603	1.6274	4.5288	2.3103	1.0037
0.3444	0	5.442	0.084	0.0671	1.0143	1.3918

1.9892	2.0581	1.9396	1.0343	1.3831	1.8872	4.4918
3.3245	0.0371	1.4917	0.633	0.266	0.8127	0.9557
4.0256	0.2415	15.1497	8.9021	0	3.7226	2.6693
1.2542	1.0133	1.6642	1.4088	1.086	3.0573	0.9971
0.0408	0.13	0.317	0.5842	1.7724	0.057	0.2311
0.1553	0.6269	2.3031	0.1878	0.811	1.636	0.4129
0.0245	0.0312	0	0.0561	0.0672	0.0616	0.0416
1.7866	1.5297	7.0121	1.8587	0.6512	1.0548	6.313
0.2565	0.1363	4.1152	0.5515	0.0979	0.8072	0.6364
0.9189	0.5635	0.733	0.8779	0	0.5601	0.4342
2.1232	3.1135	44.6834	1.1553	2.1307	6.0809	0.9979
0	0	0.6605	0	0.0777	5.1299	9.6787
1.1793	1.7342	1.4471	0.8533	4.9262	10.8394	1.4283
0	0.0699	0	0	0.3009	3.6161	0.3728
0.1853	2.4053	1.0293	0.8345	0.848	4.5895	0.2627
1.1507	0.4843	1.0624	0.0692	0.1499	1.0702	1.2858
0.3988	0.4945	0	0.9842	0.6591	0.8054	1.0677
0.006	1.8817	10.403	2.533	0.0548	0.2849	1.6987
0.0385	0.3991	0.1123	0.3864	0.9696	4.3355	2.921
1.8322	1.1468	3.3514	0.7197	0.4659	2.0117	0.9235
0.6157	0.4948	0.7007	0.3874	0.3437	1.5329	0.7522
1.0633	0.797	2.299	1.3078	1.8342	2.1416	1.9574
0.7255	0.2699	0.815	0.8087	0.3505	1.3639	0.3999
0.8919	0.5987	15.2883	0.5046	0.2149	1.838	0.8984
1.2057	0.6201	0.1815	0.524	0.7299	1.8601	0.2647
0.4675	0.1608	1.0338	0.1708	0.2098	2.5637	1.3385
1.4176	1.0416	1.4621	0.8657	0.8745	2.3098	1.8174
1.8248	3.4765	1.9526	2.3273	3.9791	1.9499	5.6617
23.8133	1.7101	141.061	6.6616	13.5834	12.3997	29.6519
0.805	0.2831	1.404	2.0019	0.7315	2.7313	0.9901
3.1326	0.365	5.2511	0.0373	0.1548	0.4075	14.4138
1.4326	0.4894	3.2432	0.8066	1.5456	1.7744	0.4352
1.5764	0.311	14.6878	0.7605	0.7858	7.5186	8.1641
2.8837	1.5131	12.0303	2.7988	3.7174	9.877	1.7076
1.5759	1.0388	2.8285	1.4116	1.3178	5.9753	1.9547
12.4675	7.7329	10.228	7.0492	26.2654	8.7648	5.4474
7.0523	0.9233	4.5395	3.6831	2.5541	0.6243	3.8484
0.0188	0	0.0285	0.012	0.5175	2.2458	0.0327
4.2044	0.8812	2.9745	1.9944	0.8357	1.1869	1.1752
1.1133	0.2207	1.4275	0.4571	0.4132	1.237	0.5289
3.7181	1.2295	20.5904	3.3945	1.2921	1.5983	11.3614
1.444	3.3364	3.2552	2.1094	6.7688	12.3785	10.0074
0.7833	0.3426	0.7279	1.3909	0.8091	1.2505	0.7075
0.8987	0.6079	2.0652	1.6003	1.2466	1.1425	0.6563
0.631	7.3257	0.8368	2.3698	5.7096	0.2715	0.0413
0.4267	0.0156	21.6689	7.9084	0.0674	0.0137	0.9248
0.3477	0.507	0.2135	0.4361	0.5079	0.3743	1.3427
0.6752	0.39	6.3795	0.6779	0.9068	2.0975	0.6219
1.1117	0.1266	2.9489	0.4097	0.8541	3.5421	0.0338
0.2241	1.1395	1.6606	0.2309	0.5074	4.0555	4.4754
0.2751	0.0219	0.2942	0.0591	0.1574	1.5772	0.663

0.5991	1.517	0	1.0101	2.4195	0.4928	0.5495
0.5614	1.5329	5.5082	0.9988	0.5759	7.7693	1.7381
0.9731	0.2635	1.3745	0.7499	1.9959	0.2182	1.0802
0.3079	0.2805	5.9014	0.4413	1.1073	0.123	0.4988
0.5632	0.0641	0.8603	0.6341	1.4729	0.3937	0.5132
4.2981	4.4831	28.4888	5.7356	5.3063	16.1683	5.8395
0.621	0.1523	5.6036	0.1597	0.6195	3.696	0.158
0.4641	0.4838	5.1024	0.4605	1.0622	0.699	0.4302
1.4776	0.5957	0.5944	0.9736	0.894	0.6887	1.3242
0.3778	0.2644	0.43	0.5547	0.0105	0.4639	0.0784
0.2837	0.3104	3.4291	0.4624	0.1401	0.988	0.3076
2.4564	2.782	9.0043	5.4249	4.1778	9.798	2.645
3.2144	1.2842	5.9964	2.4298	3.1075	4.1837	3.7084
0.3271	0.7451	3.15	0.96	0.6773	1.6337	0.2871
3.9679	7.7317	7.8955	4.1568	3.5048	3.3745	8.4537
1.001	0.3594	0.4559	1.3441	0.5366	0.5675	0.3579
0.321	0.391	4.4573	0.5676	1.7145	1.8411	0.7453
0.0654	0.1738	0	0.3749	1.746	0.1524	0.1236
0.0672	0.3571	0	0.995	1.4864	0.8769	0.5397
1.7576	0.9307	0.5313	1.7267	2.7857	0.3908	0.4314
0.1301	1.3481	0	1.3517	2.3073	1.9555	0.6916
5.6174	3.1867	8.644	4.5768	6.1123	3.1806	3.3424
8.4187	0.3823	21.083	2.329	0.1829	0.8941	9.2529
12.9615	0.1227	21.2607	1.8019	0.3188	0.3844	2.7021
0.2671	0.0355	0.8512	0.0425	0.0679	0.2179	0.021
0.7186	0.6353	2.9975	0.3557	0.7774	1.7173	0.9631
0.4483	1.0324	1.8402	1.7846	2.9638	2.3566	0.6237
0.5539	0.1962	4.1802	0.2845	0.5861	0.7967	0.4277
0.9905	1.2059	6.3381	1.9616	2.8026	1.0577	0.2042
6.5972	0.9913	19.8115	2.5725	2.4733	4.1196	3.6044
10.2984	11.7791	57.9025	7.3903	15.1141	6.1778	10.1103
2.4184	1.0077	3.7898	1.5079	3.2654	0.8627	0.6975
0.2507	0.4885	5.4161	0.998	1.4024	1.2464	1.9349
0.0878	0.513	0.1138	0.4193	0.1339	0.1227	0.3318
0.5972	0.189	0.2732	0.6922	1.6781	0.7675	0.3735
0.6311	0.2516	3.8043	0.7688	0.325	0.1544	0.4474
1.1361	0.5534	1.2579	0.3166	0.8666	0.353	0.1566
0.6558	0.738	4.1253	1.6032	0.8828	2.751	0.0547
1.2262	0.2987	16.9881	0.3173	0.3898	1.6909	0.4829
0.2223	0.2363	13.1852	0.2655	0.6784	0.4664	0.4728
1.0509	0.4134	4.9358	0.4107	0.6559	2.5001	1.006
1.6415	0.81	1.2159	0.7142	0.9392	2.6165	0.6995
0.5585	0.6092	5.144	0.8847	0.8745	2.0963	0.2917
0.9406	1.5177	9.5636	1.4455	0.6743	4.1204	0.8676
1.2441	0.3386	10.3839	1.3046	0.3472	0.7213	0.6022
1.1882	0.1973	0.4332	0.4257	2.6059	0.6923	0.7018
0.5151	0.5563	1.0015	0.4762	1.9392	1.4869	0.2826
2.3633	1.0453	2.6979	0.9615	1.326	2.0255	1.1456
1.5721	0.4592	0.7817	1.0577	0.8239	2.3577	1.8628
0.4336	0.079	0.8913	0.3018	1.1909	1.0222	0.3688
0.987	1.6611	11.0887	0.6287	1.004	1.1502	0.2332

0.9219	0.9853	0.0985	0.4646	1.8549	0.1842	0.2585
0.9717	1.0457	0.529	1.1767	1.3787	1.4403	1.4601
0.3514	0.3566	3.8825	0.6067	0.329	1.2621	0.6794
1.0679	0.4224	1.6705	0.6214	0.9371	0.3817	0.5463
0.4748	0.0935	2.0517	0.4369	0.6037	0.4427	0.1994
5.6655	0.8943	16.6873	1.6078	1.5405	1.3335	0.9542
0.262	0.4178	1.019	1.1266	0.3998	0.4886	1.362
0.6211	1.2688	2.8306	0.8902	0.9773	4.6956	1.0731
1.7434	0.5162	15.2539	0.8923	3.4198	1.1144	0.4237
1.1464	0.6092	9.3621	1.4239	1.2244	0.9619	0.9751
0.9956	0.5596	5.3857	2.1769	1.1734	0.8597	1.6587
0.6359	0.1954	40.7153	0.022	0.3332	11.2712	6.1259
1.25	0.8665	0.4227	1.151	1.6169	2.1111	0.916
1.966	1.7021	10.4117	1.5989	3.0553	2.4658	1.0129
0.6214	0.0612	0	0.033	0.0702	1.5126	0.3263
0.674	0.4605	1.5445	1.5179	1.1018	0.8415	0.3071
0.6887	0.2244	0.4521	0.371	0.4746	1.5641	0.7465
0.5405	0.7899	0.7006	1.0328	3.1438	0.8188	0.4469
1.8874	0.6357	5.7891	0.381	0.73	1.078	0.3392
0.8827	0.8485	2.8017	0.4465	0.4456	1.1255	2.4659
0.6271	0	5.3998	0.0642	0.3075	2.756	0.0635
0.4904	0.4023	0.456	0.6417	1.041	1.3865	0.1159
1.5298	0.4361	2.5609	0.4937	0.7188	1.4875	0.9479
0.4557	0.3229	1.8706	0.4354	0.1159	1.7347	1.1844
0.7301	0.4074	3.8327	0.5754	2.172	0.3573	1.2937
0.7268	0.4925	22.7521	0.8593	0.499	4.4718	0.8242
1.2892	0.9672	1.2533	0.7608	2.2562	1.7232	0.2688
0.8604	0.9631	4.2357	1.7053	1.6758	4.4285	1.7387
0.6946	0.2517	1.2482	0.2262	0.3853	1.707	0.6415
5.8033	7.2932	0.9499	11.3934	13.6538	2.3807	2.4135
0.8379	0.4693	3.0822	0.6815	0.8292	0.57	1.4446
11.9291	0.426	3.1591	0.8732	1.7858	7.2793	1.7426
1.511	0.4482	15.5778	1.3093	1.2063	1.3758	1.345
0.6637	1.0356	0.4393	0.4047	0.5817	1.4019	0.4804
2.8496	0.6758	2.3353	2.0841	1.024	5.7054	2.604
0.4957	0.4799	0.9524	0.7697	0.4322	0.4374	0.7362
0.6849	0.1437	0.4556	0.762	0.4949	1.9154	0.1405
0.4122	0.1095	3.0277	0.9517	2.2535	1.6331	1.266
0.7328	0.4746	10.4635	0.2297	0.4193	2.0494	0.2922
0.1641	0.8287	4.0426	0	0.8765	5.4704	0.1551
2.4423	0.5908	1.6048	0.6276	0.8095	2.4495	0.1194
2.0021	2.2753	2.3557	0.515	1.4568	2.3259	0.7277
12.9437	1.0779	72.0159	2.2608	1.0764	18.9494	15.1958
0.5356	0.3728	0.2054	0.3135	0.2244	1.5612	0.4705
2.4567	1.5843	14.4914	5.5384	8.9704	3.7828	5.4
9.2919	4.0128	1.9984	12.0541	3.5041	1.6425	0.5055
1.0811	1.1019	3.6182	1.0413	1.5007	0.3222	0.402
11.5432	0.2614	17.5137	0.4532	0.5227	4.7908	2.0174
0.8475	0.2654	4.414	0.4772	0.3464	1.1851	0.236
1.7789	0.3566	3.1171	0.4732	0.841	1.4448	1.2458
2.3777	2.553	4.5918	6.1894	5.1824	6.9213	2.1554

2.1954	0.385	3.415	0.5349	1.2058	0.921	2.1163
0.3261	0.0866	14.266	0.2337	0.5597	0.152	0.5778
0.8996	0.6274	1.7491	1.5309	1.2223	2.1227	1.873
4.6008	1.455	3.9886	2.1119	2.7209	3.5066	0.7834
0.5299	0.5036	2.5164	0.7156	2.4021	0.9262	0.6934
2.0187	0.7869	3.8711	1.2399	1.688	0.6979	0.3538
5.1992	10.212	6.1464	9.7759	27.1602	30.1279	13.9544
0.3928	0.4273	0.4942	1.0995	1.0471	0.5364	0.1769
3.0466	2.9489	0.4419	2.6608	3.2594	3.0738	3.1871
5.1245	3.8571	5.4738	1.7966	0.8053	6.4277	3.8037
0.7992	1.2357	0.2889	1.0072	1.4981	3.6389	0.2733
4.0626	0.698	9.7205	1.1528	0.5359	2.5059	0.7506
0.4837	0.0846	1.1699	0.0169	0.054	0.2227	0.0502
0.3397	0.2235	0.1529	0.2891	2.5773	0.1976	0.105
0.7889	1.0062	45.2524	4.4095	5.7776	1.1584	3.0196
2.6667	0.5339	0.0977	0.408	0.2395	1.1357	0.0593
2.1039	0.5355	1.9823	0.4054	0.553	3.0244	1.5205
0.4177	0.4214	1.2027	0.4698	0.5525	0.8625	0.8794
1.4617	4.3069	0.1903	8.9121	8.3627	4.2247	4.2921
0.4571	0.4958	0.5529	0.3428	0.3041	1.2933	0.3089
0.0517	6.3411	1.3955	9.0041	0.4604	1.4598	1.4067
0.5954	0.2483	0.5788	0.361	2.6727	0.4163	0.4545
0.0229	0.0366	1.7398	0	0.0525	2.727	13.1069
0.0293	0.8314	0.0456	0.0168	0.0268	0.4015	0.191
0.4573	0.1646	1.5201	0.4862	0.4726	0.6394	0.3764
1.025	0.906	2.4665	0.9453	1.0683	3.0084	1.1365
0.0474	0.0058	0.3122	0.0314	0.2672	8.2258	2.6279
0.3275	0.1088	0	0	0.0312	0.2481	0.5998
2.2454	0.4036	5.5429	0.6467	0.6045	2.0778	0.2809
0.9089	0.5954	1.2467	1.0799	0.5521	2.3061	1.0916
0.9361	1.3739	0.5489	0.4046	1.1901	8.9965	1.0742
0.6128	1.2394	1.1737	1.0486	0.5756	1.036	0.7261
0.6984	0.8466	1.6266	0.49	1.6148	2.0853	0.33
4.8812	0.1621	8.6011	0.9837	0.1164	0.6399	14.5239
2.8273	1.4176	0.1554	0.7302	3.6125	0.2235	0.17
0.6994	0.1439	4.2552	0.4527	0.4647	0.7887	0.8316
0.3019	1.333	0.6097	0.8488	1.9932	0.3573	0.1646
0.31	0.2648	0.0753	1.3487	8.4374	0.2864	0.3139
1.9814	0.6194	0.6988	2.6587	3.0675	1.3989	0.5232
2.7933	0.1922	4.8257	0.7056	0.4139	0.843	0.4701
7.5444	1.3597	11.4291	1.4479	4.8043	1.1742	0.3162
0.2929	0.0584	2.2068	0.5247	1.5082	0.9215	0.1817
0.8833	0.4694	0.3053	1.1815	1.2578	0.3843	1.0573
