# Supplementary material for: The Risk Correlation between N7-Methylguanosine Modification-Related lncRNAs and Survival Prognosis of Oral Squamous Cell Carcinoma Based on Comprehensive Bioinformatics Analysis
Source: Appl Bionics Biomech. 2022 Aug 24;2022:1666792. doi: 10.1155/2022/1666792 (PMC9433249; doi:10.1155/2022/1666792)
Supplement: Supplementary Materials — File m7G-lncRNAs_exp.xls shows the expression matrix of 399 m7G-related lncRNAs. Rows represent m7G-related lncRNA names, and columns represent samples. File co-exp_rel.xls shows the coexpression relationship of m7G-related lncRNAs and m7G-realated mRNAs. The first column represents m7G-realated mRNAs, the second column represents m7G-realated lncRNAs, the third column represents coexpression correlation coefficients, and the fourth column represents the P value of the correlation test. File risk.xls presents univariate Cox regression analysis for 16 significant m7G-related prognostic lncRNAs. The first column represents samples, the second column represents the survival time of patients, the third column represents their survival status, and columns 4 to 19 represent m7G-related prognostic lncRNAs. File risk.xls presents the risk scores of nine m7G-related prognostic lncRNAs that constitute the prognostic model. The first column represents samples, the second column represents the survival time of patients, the third column represents their survival status, columns 4 to 12 represent m7G-related prognostic lncRNAs, and columns 13 and 14 represent the risk score and risk grouping for each patient. File coexp_network.xls shows the coexpression relationship between the m7G-related prognostic lncRNAs and mRNAs. The first column represents prognostic m7G-realated mRNAs, the second column represents prognostic m7G-realated lncRNAs, and the third column represents the correlation type. [file 1666792.f1.zip › risk.pdf]

id	futime	fustat	HHLA3	AC010894.1	AL513550.1	LINC02541	AL035446.1
TCGA-CV-71	5.9342466	1	7.5772	1.0049	1.1871	2.4611	0.6338
TCGA-CR-73	3.9452055	0	1.2812	0.2403	0.8968	1.6533	0.091
TCGA-IQ-76	1.2082192	0	3.8298	0.4962	0.1857	1.3356	1.1069
TCGA-CQ-62	3.9123288	0	2.3432	0.3053	0.5191	2.1181	0.0899
TCGA-CV-A6	0.5315068	1	15.7409	2.4074	3.7392	5.3298	1.5746
TCGA-CV-74	4.7068493	1	2.3232	0.3412	0.5344	2.3267	0.2009
TCGA-CQ-53	0.8684932	1	3.0364	3.6244	0.8925	5.2633	1.9528
TCGA-CV-69	1.709589	1	3.5581	1.0104	1.5724	3.3669	2.4611
TCGA-CV-71	0.7506849	1	9.5558	1.3675	1.1395	4.0279	1.89
TCGA-CR-73	3.4109589	0	7.9556	0.3467	0.852	1.9919	0.3061
TCGA-CV-70	1.0547945	1	1.227	0.7826	0.6876	4.1311	0.5092
TCGA-CQ-70	2.6465753	0	4.5598	0.4824	0.9602	1.7812	0.1363
TCGA-CV-72	0.4136986	1	17.4103	0.1533	0.7652	1.5495	1.1219
TCGA-CR-73	3.2630137	0	2.0706	0.4597	0.3126	1.374	0.2923
TCGA-CV-69	11.731507	0	7.2368	0.3848	0.4325	0.6505	1.0502
TCGA-CR-73	2.4356164	0	5.1032	0.0821	0.4249	0.5859	0.3623
TCGA-CV-70	1.5671233	1	4.133	1.4336	0.3324	2.3346	0.4382
TCGA-CV-72	1.5342466	1	1.6618	0.4844	0.575	1.7117	1.1635
TCGA-CQ-62	0.3534247	1	8.8182	0.5241	0.9235	1.0559	0.9564
TCGA-CV-74	13.041096	1	3.623	0.1511	0.4729	1.3611	0.0334
TCGA-BA-55	0.6630137	0	2.7765	0.0891	0.3856	1.3966	0.3462
TCGA-CQ-53	4.3643836	0	1.7817	0.1495	0.2126	0.7449	0
TCGA-CV-74	4.5780822	1	2.7736	0.2839	0.7763	2.1829	1.6969
TCGA-CV-75	2.539726	1	1.4279	0.5827	0.3448	2.0292	0.2352
TCGA-CV-74	7.0410959	1	9.0412	0.1656	0.3968	4.5313	1.097
TCGA-CV-74	0.2931507	1	3.22	1.031	0.7901	0.3994	4.2862
TCGA-CV-70	9.2630137	0	2.8484	0.6768	0.5679	3.1338	1.127
TCGA-CQ-62	2.6986301	1	9.7016	0.6333	0.9162	2.6331	0.85
TCGA-CV-72	3.9972603	1	3.4565	0.5708	0.8558	4.3408	0.4536
TCGA-CV-69	0.3945205	1	3.3835	0.4512	0.6086	0.6266	2.8251
TCGA-CV-74	8.3808219	1	3.4832	0.5847	0.6025	2.6665	1.6521
TCGA-CV-74	0.0383562	1	7.9186	2.733	0.9647	2.8516	0.1304
TCGA-CN-47	2.7205479	0	3.7689	0.5663	1.0503	4.6229	5.0001
TCGA-CV-72	0.9890411	1	4.7192	0.1901	0.7052	3.1883	2.8259
TCGA-CN-47	1.0739726	0	1.9041	0.222	0.1373	0.9511	0.9018
TCGA-CV-59	1.4931507	1	8.6654	0.5506	0.6263	1.3238	0.2723
TCGA-CQ-53	3.8328767	0	2.5237	0.8116	0.3942	0.8615	0.0512
TCGA-CQ-53	0.2438356	1	4.7194	0.1681	0.5542	0.4962	1.9789
TCGA-CV-70	0.6657534	1	4.1052	0.209	0.2226	1.4037	1.1484
TCGA-CQ-53	4.0164384	0	2.0067	0.4327	1.1482	0.8622	0.1274
TCGA-CV-74	7.4438356	1	1.5684	0.13	0.3444	0.7832	0
TCGA-CN-69	2.0136986	0	4.5325	1.0566	0.4212	4.9215	0.2455
TCGA-CQ-62	1.1041096	1	3.9803	2.3218	1.9463	9.1674	2.0849
TCGA-BA-51	2.3178082	0	2.5618	0.2904	0.5198	0.5888	3.1313
TCGA-CV-71	10.906849	0	1.9579	0.0927	0.2795	1.1164	0.7779
TCGA-CX-70	0.030137	1	2.6258	1.4512	3.2211	4.846	5.5172
TCGA-BA-55	4.4821918	0	2.6372	0.1446	0.3704	0.5077	3.4294
TCGA-CV-74	12.821918	1	1.3021	0.179	0.8485	2.4175	2.0022
TCGA-CR-64	3.2931507	1	2.0105	0.2721	0.1322	1.1231	0.0343
TCGA-CR-73	2.9863014	1	2.1641	0.5644	1.7072	3.869	0.9611

TCGA-DQ-75	1.169863	1	15.6269	0.3803	0.1798	2.5631	0.4741
TCGA-CV-69	0.9150685	1	3.238	0.3106	0.2437	0.5158	0
TCGA-CV-69	2.3616438	1	4.4918	1.1756	1.5659	9.9659	7.4363
TCGA-CV-70	14.389041	0	3.478	0.195	0.4671	1.5295	0.3443
TCGA-CR-64	0.969863	0	3.5809	0.4578	0.9609	1.2483	0.6809
TCGA-HL-75	2.8958904	0	3.3324	0.5116	0.6642	2.6418	0.4517
TCGA-CR-73	3.9178082	0	4.2002	0.0792	0.9794	1.7273	0.1399
TCGA-CR-73	2.6630137	0	2.8799	0.1408	0.6527	0.7538	0.2486
TCGA-CR-73	1.660274	1	3.5848	0.8674	0.5698	1.9625	1.2925
TCGA-CV-74	0.5972603	1	3.7364	0.1741	1.7463	3.9192	2.1867
TCGA-CN-60	1.5890411	1	4.4912	0.8114	1.0641	4.1563	0.6948
TCGA-CV-74	0.8054795	1	12.5632	0.8958	0.7323	3.7153	2.2851
TCGA-CV-74	2.090411	1	6.8481	0.5747	0.379	5.4333	1.4548
TCGA-CN-47	3.1534247	0	2.4117	0.4927	0.6745	1.2467	0.5801
TCGA-H7-77	1.1150685	0	2.3724	0.1104	0.2786	0.4643	0.065
TCGA-CN-47	2.2383562	0	0.8795	0.26	0.4756	1.9645	0.5357
TCGA-CQ-62	1.2493151	1	3.7986	2.5593	3.6718	15.5383	3.5881
TCGA-CR-73	2.8383562	0	2.8795	0.6619	0.4554	1.0069	2.0739
TCGA-CR-73	0.7643836	1	3.6335	0.2337	0.9933	0.8601	0.8942
TCGA-CR-73	2.5479452	0	1.699	0.1527	0.2241	0.8491	0.674

AC007114.1	AC068831.5	AC005332.6	HEIH	riskScore	risk
0.5686	0.66	2.1133	1.3867	1.508644	high
0.2497	0.2842	2.3697	0.6976	0.2970793	low
0.5291	1.1527	3.2974	1.0517	0.9419831	high
0.2291	0.6551	1.719	0.9786	0.1749236	low
2.9489	1.7491	3.9886	3.8711	38.742971	high
0.1773	1.3806	2.5357	0.6437	0.6715244	low
1.1329	0.5649	2.6078	0.9472	8.6204277	high
1.3212	1.0252	3.4637	2.1615	1.2227916	high
1.0038	2.7336	5.0235	2.2974	24.56715	high
1.1797	0.5739	3.1811	0.6919	0.9966242	high
0.2889	0.8333	1.6076	1.1433	0.1659533	low
0.9522	0.5323	2.6157	2.114	0.197504	low
0.4097	1.5309	2.1119	1.2399	7.7190838	high
0.1528	1.0484	1.7627	0.5738	0.4509403	low
0.8541	1.2223	2.7209	1.688	0.8632695	high
0.1385	1.6599	1.9158	0.908	0.9742688	high
0.2005	0.5577	1.9329	1.0902	0.798448	high
0.5838	1.8887	1.6876	1.6943	0.3652038	low
0.3721	0.6747	3.0288	1.0078	3.5766397	high
0.2551	0.6947	1.9192	0.7469	0.2945711	low
0.3425	0.2949	0.9712	1.015	0.0772276	low
0.1709	0.33	2.7195	0.9983	0.1972194	low
0.2042	0.241	3.4152	1.2994	0.5374322	low
0.8736	2.0209	2.9823	1.9874	0.3050074	low
0.086	1.3708	1.5027	0.7362	0.5662433	low
0.4351	0.869	3.8295	0.8026	14.71556	high
0.2913	1.6359	2.4097	0.6174	1.263297	high
0.658	1.1181	2.0259	1.0661	1.7768482	high
1.1564	1.0497	7.1911	1.7913	1.5693865	high
0.277	0.9052	3.2017	1.2799	2.0827638	high
0.1215	0.5735	1.5853	0.4525	0.6349311	low
0.5084	1.4264	5.6544	1.0787	134.24447	high
0.2206	1.5187	2.4232	1.0015	3.0076292	high
1.1934	2.9137	2.9028	0.7932	2.9318596	high
0.2422	0.4491	3.2532	0.6203	0.472345	low
0.2631	0.4455	2.4429	1.2305	1.0958197	high
0.2861	0.613	2.179	1.0095	0.5071578	low
0.65	0.4122	2.4552	0.8334	0.6998561	low
0.3378	1.2814	3.2313	0.8258	1.0544484	high
0.7306	0.5969	3.4667	1.0598	0.8726862	high
0.2508	0.6148	2.5228	0.4851	0.3354658	low
2.0366	0.2557	4.9498	1.4224	0.1934952	low
1.7376	2.7501	3.2225	1.3992	6.2874504	high
1.1117	0.8996	4.6008	2.0187	0.9503734	high
0.1325	0.469	1.9747	0.8748	0.1934931	low
1.3507	2.5206	3.8255	1.426	103.78354	high
0.0322	0.9118	0.7582	0.8694	0.5493956	low
0.248	0.3292	4.0583	0.4979	1.1124671	high
0.2625	0.9294	2.9864	1.2341	0.2832985	low
0.356	0.4078	2.6388	0.841	0.7903199	low

0.3603	0.4937	3.7007	1.0714	3.0084139	high
0.4965	0.5713	2.5325	1.4564	0.2060284	low
3.5421	2.1227	3.5066	0.6979	1.4708059	high
0.1266	0.6274	1.455	0.7869	0.2463552	low
0.4131	0.7977	2.2372	1.39	0.6024145	low
0.6777	0.1882	3.9372	0.8724	0.5243561	low
0.3155	0.4856	2.379	1.3123	0.3023879	low
0.2377	0.1942	3.4169	1.0789	0.4640583	low
0.2816	1.346	2.2198	0.8065	1.72841	high
0.1608	0.8895	3.0309	0.6458	2.342743	high
1.2646	0.3165	2.9025	1.017	0.3384659	low
1.1065	2.5993	3.5439	1.4662	14.122345	high
1.6123	2.6778	2.6445	0.9453	0.7459974	low
0.6948	0.0863	4.0485	1.5323	0.4069694	low
0.2102	0.3721	1.7935	1.0011	0.1513324	low
0.0338	1.873	0.7834	0.3538	0.4434684	low
0.6698	1.6168	2.8305	2.3391	3.273176	high
0.3106	0.7854	1.4386	0.4106	1.0255124	high
0.2428	0.573	4.0855	1.081	1.8976189	high
0.2181	0.6317	1.6724	1.26	0.132335	low
