# Supplementary material for: The Risk Correlation between N7-Methylguanosine Modification-Related lncRNAs and Survival Prognosis of Oral Squamous Cell Carcinoma Based on Comprehensive Bioinformatics Analysis
Source: Appl Bionics Biomech. 2022 Aug 24;2022:1666792. doi: 10.1155/2022/1666792 (PMC9433249; doi:10.1155/2022/1666792)
Supplement: Supplementary Materials — File m7G-lncRNAs_exp.xls shows the expression matrix of 399 m7G-related lncRNAs. Rows represent m7G-related lncRNA names, and columns represent samples. File co-exp_rel.xls shows the coexpression relationship of m7G-related lncRNAs and m7G-realated mRNAs. The first column represents m7G-realated mRNAs, the second column represents m7G-realated lncRNAs, the third column represents coexpression correlation coefficients, and the fourth column represents the P value of the correlation test. File risk.xls presents univariate Cox regression analysis for 16 significant m7G-related prognostic lncRNAs. The first column represents samples, the second column represents the survival time of patients, the third column represents their survival status, and columns 4 to 19 represent m7G-related prognostic lncRNAs. File risk.xls presents the risk scores of nine m7G-related prognostic lncRNAs that constitute the prognostic model. The first column represents samples, the second column represents the survival time of patients, the third column represents their survival status, columns 4 to 12 represent m7G-related prognostic lncRNAs, and columns 13 and 14 represent the risk score and risk grouping for each patient. File coexp_network.xls shows the coexpression relationship between the m7G-related prognostic lncRNAs and mRNAs. The first column represents prognostic m7G-realated mRNAs, the second column represents prognostic m7G-realated lncRNAs, and the third column represents the correlation type. [file 1666792.f1.zip › uniSigExp.pdf]

id	futime	fustat	TMEM99	HHLA3	AC010894.1	AL513550.1	LINC02541
TCGA-CV-71	5.9342466	1	3.0517	7.5772	1.0049	1.1871	2.4611
TCGA-CR-73	3.9452055	0	2.9375	1.2812	0.2403	0.8968	1.6533
TCGA-IQ-76	1.2082192	0	2.1694	3.8298	0.4962	0.1857	1.3356
TCGA-CQ-62	3.9123288	0	2.3959	2.3432	0.3053	0.5191	2.1181
TCGA-CV-A6	0.5315068	1	9.291	15.7409	2.4074	3.7392	5.3298
TCGA-CV-74	4.7068493	1	1.5736	2.3232	0.3412	0.5344	2.3267
TCGA-CQ-53	0.8684932	1	7.0828	3.0364	3.6244	0.8925	5.2633
TCGA-CV-69	1.709589	1	5.3316	3.5581	1.0104	1.5724	3.3669
TCGA-CV-71	0.7506849	1	6.4422	9.5558	1.3675	1.1395	4.0279
TCGA-CR-73	3.4109589	0	4.1565	7.9556	0.3467	0.852	1.9919
TCGA-CV-70	1.0547945	1	3.6051	1.227	0.7826	0.6876	4.1311
TCGA-CQ-70	2.6465753	0	2.1639	4.5598	0.4824	0.9602	1.7812
TCGA-CV-72	0.4136986	1	6.1363	17.4103	0.1533	0.7652	1.5495
TCGA-CR-73	3.2630137	0	3.8837	2.0706	0.4597	0.3126	1.374
TCGA-CV-69	11.731507	0	1.9983	7.2368	0.3848	0.4325	0.6505
TCGA-CR-73	2.4356164	0	1.4245	5.1032	0.0821	0.4249	0.5859
TCGA-CV-70	1.5671233	1	3.3447	4.133	1.4336	0.3324	2.3346
TCGA-CV-72	1.5342466	1	2.95	1.6618	0.4844	0.575	1.7117
TCGA-CQ-62	0.3534247	1	5.2463	8.8182	0.5241	0.9235	1.0559
TCGA-CV-74	13.041096	1	1.3116	3.623	0.1511	0.4729	1.3611
TCGA-BA-55	0.6630137	0	2.4374	2.7765	0.0891	0.3856	1.3966
TCGA-CQ-53	4.3643836	0	2.1865	1.7817	0.1495	0.2126	0.7449
TCGA-CV-74	4.5780822	1	1.8083	2.7736	0.2839	0.7763	2.1829
TCGA-CV-75	2.539726	1	5.8594	1.4279	0.5827	0.3448	2.0292
TCGA-CV-74	7.0410959	1	5.3062	9.0412	0.1656	0.3968	4.5313
TCGA-CV-74	0.2931507	1	4.8177	3.22	1.031	0.7901	0.3994
TCGA-CV-70	9.2630137	0	2.0471	2.8484	0.6768	0.5679	3.1338
TCGA-CQ-62	2.6986301	1	3.7305	9.7016	0.6333	0.9162	2.6331
TCGA-CV-72	3.9972603	1	4.3749	3.4565	0.5708	0.8558	4.3408
TCGA-CV-69	0.3945205	1	2.9021	3.3835	0.4512	0.6086	0.6266
TCGA-CV-74	8.3808219	1	3.6515	3.4832	0.5847	0.6025	2.6665
TCGA-CV-74	0.0383562	1	11.5021	7.9186	2.733	0.9647	2.8516
TCGA-CN-47	2.7205479	0	6.6401	3.7689	0.5663	1.0503	4.6229
TCGA-CV-72	0.9890411	1	2.9475	4.7192	0.1901	0.7052	3.1883
TCGA-CN-47	1.0739726	0	5.7538	1.9041	0.222	0.1373	0.9511
TCGA-CV-59	1.4931507	1	3.9878	8.6654	0.5506	0.6263	1.3238
TCGA-CQ-53	3.8328767	0	2.9924	2.5237	0.8116	0.3942	0.8615
TCGA-CQ-53	0.2438356	1	2.7552	4.7194	0.1681	0.5542	0.4962
TCGA-CV-70	0.6657534	1	2.4526	4.1052	0.209	0.2226	1.4037
TCGA-CQ-53	4.0164384	0	1.7482	2.0067	0.4327	1.1482	0.8622
TCGA-CV-74	7.4438356	1	1.8687	1.5684	0.13	0.3444	0.7832
TCGA-CN-69	2.0136986	0	3.6648	4.5325	1.0566	0.4212	4.9215
TCGA-CQ-62	1.1041096	1	19.1488	3.9803	2.3218	1.9463	9.1674
TCGA-BA-51	2.3178082	0	2.235	2.5618	0.2904	0.5198	0.5888
TCGA-CV-71	10.906849	0	2.8796	1.9579	0.0927	0.2795	1.1164
TCGA-CX-70	0.030137	1	8.0807	2.6258	1.4512	3.2211	4.846
TCGA-BA-55	4.4821918	0	2.2357	2.6372	0.1446	0.3704	0.5077
TCGA-CV-74	12.821918	1	2.387	1.3021	0.179	0.8485	2.4175
TCGA-CR-64	3.2931507	1	1.1714	2.0105	0.2721	0.1322	1.1231
TCGA-CR-73	2.9863014	1	2.7096	2.1641	0.5644	1.7072	3.869

TCGA-DQ-75	1.169863	1	5.3404	15.6269	0.3803	0.1798	2.5631
TCGA-CV-69	0.9150685	1	2.1288	3.238	0.3106	0.2437	0.5158
TCGA-CV-69	2.3616438	1	7.6957	4.4918	1.1756	1.5659	9.9659
TCGA-CV-70	14.389041	0	1.6156	3.478	0.195	0.4671	1.5295
TCGA-CR-64	0.969863	0	1.8437	3.5809	0.4578	0.9609	1.2483
TCGA-HL-75	2.8958904	0	2.1717	3.3324	0.5116	0.6642	2.6418
TCGA-CR-73	3.9178082	0	1.5566	4.2002	0.0792	0.9794	1.7273
TCGA-CR-73	2.6630137	0	1.6138	2.8799	0.1408	0.6527	0.7538
TCGA-CR-73	1.660274	1	2.2233	3.5848	0.8674	0.5698	1.9625
TCGA-CV-74	0.5972603	1	1.6274	3.7364	0.1741	1.7463	3.9192
TCGA-CN-60	1.5890411	1	3.9239	4.4912	0.8114	1.0641	4.1563
TCGA-CV-74	0.8054795	1	6.1709	12.5632	0.8958	0.7323	3.7153
TCGA-CV-74	2.090411	1	6.1045	6.8481	0.5747	0.379	5.4333
TCGA-CN-47	3.1534247	0	2.2934	2.4117	0.4927	0.6745	1.2467
TCGA-H7-77	1.1150685	0	1.2822	2.3724	0.1104	0.2786	0.4643
TCGA-CN-47	2.2383562	0	4.7071	0.8795	0.26	0.4756	1.9645
TCGA-CQ-62	1.2493151	1	1.6075	3.7986	2.5593	3.6718	15.5383
TCGA-CR-73	2.8383562	0	2.6576	2.8795	0.6619	0.4554	1.0069
TCGA-CR-73	0.7643836	1	2.1943	3.6335	0.2337	0.9933	0.8601
TCGA-CR-73	2.5479452	0	2.0301	1.699	0.1527	0.2241	0.8491

AL035446.1	MAPKAPK5-A	CASC9	FLJ20021	AC007114.1	AC010326.3	PCCA-DT	AP001505.1
0.6338	2.7897	5.2384	3.6343	0.5686	1.9874	5.3842	1.8147
0.091	1.8901	1.754	2.6425	0.2497	2.3868	1.7523	2.9452
1.1069	2.0766	2.2327	2.3009	0.5291	1.3539	3.0982	3.6786
0.0899	1.7336	4.5865	2.5596	0.2291	1.6389	2.0451	0.7352
1.5746	6.1733	11.6577	46.8009	2.9489	6.3381	2.3353	14.4914
0.2009	1.4899	2.867	3.248	0.1773	1.3401	1.4807	1.7664
1.9528	5.965	4.2626	7.3406	1.1329	1.8456	3.4546	1.9969
2.4611	2.9923	10.784	4.5513	1.3212	6.732	4.0795	2.8942
1.89	3.4852	7.342	7.2551	1.0038	2.3467	1.7176	4.6169
0.3061	2.1737	4.2373	2.6508	1.1797	1.9608	4.3802	2.4419
0.5092	2.3831	0.0048	3.3162	0.2889	1.5288	1.8087	9.5587
0.1363	2.3652	0.0045	11.1955	0.9522	2.3642	2.6085	4.5644
1.1219	2.7245	3.3395	2.1518	0.4097	1.9616	2.0841	5.5384
0.2923	1.6168	0.0043	3.836	0.1528	0.78	0.6162	2.0588
1.0502	2.5765	0.0973	3.5779	0.8541	2.8026	1.024	8.9704
0.3623	2.1226	5.2707	2.3058	0.1385	1.4743	1.0035	5.0751
0.4382	2.0095	1.0922	4.3551	0.2005	2.2737	2.7184	4.9785
1.1635	4.7754	4.4803	4.003	0.5838	1.9634	0.9703	4.374
0.9564	2.4623	0.0121	7.0944	0.3721	1.5644	1.7765	2.4609
0.0334	1.3911	0.0131	3.022	0.2551	1.2462	0.9385	2.7628
0.3462	1.0763	0	4.8902	0.3425	0.3149	0.6109	1.0297
0	1.6845	4.0328	5.9967	0.1709	1.3743	1.8316	2.2684
1.6969	3.9294	1.1182	6.7229	0.2042	2.007	1.9935	3.6282
0.2352	4.5616	0.3395	2.4111	0.8736	2.0009	0.6862	1.6236
1.097	2.4538	0.0096	7.1918	0.086	1.7808	2.0097	6.8425
4.2862	3.9905	3.0902	7.5908	0.4351	2.6065	5.427	7.9513
1.127	2.1537	1.2231	3.4857	0.2913	2.4834	2.9421	2.1302
0.85	2.5437	2.2537	3.6427	0.658	0.7163	1.5124	3.3395
0.4536	4.226	2.5192	5.3467	1.1564	0.9079	4.2322	3.1954
2.8251	2.2371	5.8255	4.2369	0.277	1.7398	3.3178	7.2593
1.6521	2.3196	3.4271	2.9209	0.1215	1.4926	1.7791	2.6396
0.1304	1.8025	9.006	7.0126	0.5084	1.9582	8.8412	3.5016
5.0001	5.1713	2.6403	12.5676	0.2206	1.0286	3.7623	2.2582
2.8259	3.0485	2.8594	6.7864	1.1934	1.68	3.3807	3.4619
0.9018	2.1728	0.4115	2.2034	0.2422	1.5172	3.3608	3.0472
0.2723	3.3117	0.0102	3.5461	0.2631	2.3613	3.4695	18.1362
0.0512	1.6029	2.4947	3.5368	0.2861	1.2635	1.7583	2.7741
1.9789	2.0372	2.9942	6.5437	0.65	0.7041	1.9638	1.6863
1.1484	2.2907	3.363	7.6437	0.3378	2.2165	2.1559	5.1588
0.1274	3.0053	1.6793	3.4327	0.7306	1.0197	1.8196	2.7352
0	0.975	3.0936	2.8586	0.2508	0.8315	1.0157	1.0061
0.2455	2.9975	6.532	10.3334	2.0366	1.4414	3.6091	2.4101
2.0849	6.6443	10.6261	8.0099	1.7376	2.5501	14.4373	5.0101
3.1313	4.3553	4.664	3.328	1.1117	0.9905	2.8496	2.4567
0.7779	0.8863	1.7618	1.9956	0.1325	0.6829	1.0626	1.6747
5.5172	6.1847	12.0291	6.7151	1.3507	4.0845	3.7652	4.1494
3.4294	2.4008	0	2.4895	0.0322	2.1906	1.7664	1.828
2.0022	1.9922	1.6175	3.1622	0.248	1.617	1.785	1.2931
0.0343	1.3127	3.0307	1.9486	0.2625	0.8931	0.9283	1.5795
0.9611	2.584	2.7132	2.674	0.356	2.1136	3.6645	3.3488

0.4741	3.5121	2.7729	8.7446	0.3603	1.0543	3.5587	8.6051
0	1.2179	4.556	2.8552	0.4965	0.9572	3.2488	0.6041
7.4363	4.9681	5.477	16.8615	3.5421	1.0577	5.7054	3.7828
0.3443	1.2049	0.1073	3.5043	0.1266	1.2059	0.6758	1.5843
0.6809	2.6407	5.9065	2.5133	0.4131	2.8106	5.5873	0.9138
0.4517	2.7054	2.1631	2.5903	0.6777	1.6576	1.3793	6.8828
0.1399	1.8437	7.01	3.9035	0.3155	0.4355	0.722	1.6688
0.2486	1.3768	0.4729	2.3699	0.2377	1.2439	0.9353	2.1608
1.2925	1.2781	0.7222	1.4549	0.2816	1.1817	2.1926	2.4966
2.1867	1.8293	4.2224	3.8009	0.1608	2.3707	0.5962	4.3673
0.6948	2.5255	0.7007	5.2789	1.2646	1.1299	1.2881	1.4209
2.2851	3.2673	3.1777	2.7618	1.1065	1.7356	2.814	4.5656
1.4548	3.6815	4.6916	4.1324	1.6123	1.5801	2.2949	2.3872
0.5801	2.8584	3.1638	3.3503	0.6948	1.0505	1.8346	2.161
0.065	1.0783	2.1906	1.602	0.2102	0.6068	1.5729	1.0962
0.5357	4.1584	0.2058	2.677	0.0338	0.2042	2.604	5.4
3.5881	6.9109	17.6931	4.6492	0.6698	3.4297	5.2147	3.8455
2.0739	3.2922	5.5603	8.6921	0.3106	0.6792	1.8258	4.2029
0.8942	2.0548	3.0139	2.0904	0.2428	1.0096	0.6676	2.919
0.674	1.4492	0.0088	3.8647	0.2181	1.3489	1.0878	3.2392

AC068831.5	AC005332.6	HEIH
0.66	2.1133	1.3867
0.2842	2.3697	0.6976
1.1527	3.2974	1.0517
0.6551	1.719	0.9786
1.7491	3.9886	3.8711
1.3806	2.5357	0.6437
0.5649	2.6078	0.9472
1.0252	3.4637	2.1615
2.7336	5.0235	2.2974
0.5739	3.1811	0.6919
0.8333	1.6076	1.1433
0.5323	2.6157	2.114
1.5309	2.1119	1.2399
1.0484	1.7627	0.5738
1.2223	2.7209	1.688
1.6599	1.9158	0.908
0.5577	1.9329	1.0902
1.8887	1.6876	1.6943
0.6747	3.0288	1.0078
0.6947	1.9192	0.7469
0.2949	0.9712	1.015
0.33	2.7195	0.9983
0.241	3.4152	1.2994
2.0209	2.9823	1.9874
1.3708	1.5027	0.7362
0.869	3.8295	0.8026
1.6359	2.4097	0.6174
1.1181	2.0259	1.0661
1.0497	7.1911	1.7913
0.9052	3.2017	1.2799
0.5735	1.5853	0.4525
1.4264	5.6544	1.0787
1.5187	2.4232	1.0015
2.9137	2.9028	0.7932
0.4491	3.2532	0.6203
0.4455	2.4429	1.2305
0.613	2.179	1.0095
0.4122	2.4552	0.8334
1.2814	3.2313	0.8258
0.5969	3.4667	1.0598
0.6148	2.5228	0.4851
0.2557	4.9498	1.4224
2.7501	3.2225	1.3992
0.8996	4.6008	2.0187
0.469	1.9747	0.8748
2.5206	3.8255	1.426
0.9118	0.7582	0.8694
0.3292	4.0583	0.4979
0.9294	2.9864	1.2341
0.4078	2.6388	0.841

0.4937	3.7007	1.0714
0.5713	2.5325	1.4564
2.1227	3.5066	0.6979
0.6274	1.455	0.7869
0.7977	2.2372	1.39
0.1882	3.9372	0.8724
0.4856	2.379	1.3123
0.1942	3.4169	1.0789
1.346	2.2198	0.8065
0.8895	3.0309	0.6458
0.3165	2.9025	1.017
2.5993	3.5439	1.4662
2.6778	2.6445	0.9453
0.0863	4.0485	1.5323
0.3721	1.7935	1.0011
1.873	0.7834	0.3538
1.6168	2.8305	2.3391
0.7854	1.4386	0.4106
0.573	4.0855	1.081
0.6317	1.6724	1.26
